# Drivers of stunting reduction in Ethiopia: a country case study

**DOI:** 10.1093/ajcn/nqaa163

**Published:** 2020-08-25

**Authors:** Hana Tasic, Nadia Akseer, Seifu H Gebreyesus, Anushka Ataullahjan, Samanpreet Brar, Erica Confreda, Kaitlin Conway, Bilal S Endris, Muhammad Islam, Emily Keats, Afrah Mohammedsanni, Jannah Wigle, Zulfiqar A Bhutta

**Affiliations:** Centre for Global Child Health, Hospital for Sick Children, Toronto, Ontario, Canada; Centre for Global Child Health, Hospital for Sick Children, Toronto, Ontario, Canada; Dalla Lana School of Public Health, University of Toronto, Toronto, Ontario, Canada; Department of Nutrition and Dietetics, School of Public Health, Addis Ababa University, Addis Ababa, Ethiopia; Centre for Global Child Health, Hospital for Sick Children, Toronto, Ontario, Canada; Centre for Global Child Health, Hospital for Sick Children, Toronto, Ontario, Canada; Centre for Global Child Health, Hospital for Sick Children, Toronto, Ontario, Canada; Centre for Global Child Health, Hospital for Sick Children, Toronto, Ontario, Canada; Department of Nutrition and Dietetics, School of Public Health, Addis Ababa University, Addis Ababa, Ethiopia; Centre for Global Child Health, Hospital for Sick Children, Toronto, Ontario, Canada; Centre for Global Child Health, Hospital for Sick Children, Toronto, Ontario, Canada; Department of Nutrition and Dietetics, School of Public Health, Addis Ababa University, Addis Ababa, Ethiopia; Centre for Global Child Health, Hospital for Sick Children, Toronto, Ontario, Canada; Centre for Global Child Health, Hospital for Sick Children, Toronto, Ontario, Canada; Dalla Lana School of Public Health, University of Toronto, Toronto, Ontario, Canada; Center of Excellence in Women and Child Health, The Aga Khan University, Karachi, Pakistan

**Keywords:** stunting, linear growth, children, nutrition, exemplar, Ethiopia, East Africa, mixed methods

## Abstract

**Background:**

Chronic undernutrition in children continues to be a global public health concern. Ethiopia has documented a significant decline in the prevalence of childhood stunting, a measure of chronic undernutrition, over the last 20 y.

**Objectives:**

The aim of this research was to conduct a systematic assessment of the determinants that have driven child stunting reduction in Ethiopia from 2000 to 2016, focused on the national, community, household, and individual level.

**Methods:**

This study employed both quantitative and qualitative methods. Specifically, a systematic literature review, retrospective quantitative data analysis using Demographic and Health Surveys from 2000–2016, qualitative data collection and analysis, and analyses of key nutrition-specific and -sensitive policies and programs were undertaken.

**Results:**

National stunting prevalence improved from 51% in 2000 to 32% in 2016. Regional variations exist, as do pro-rich, pro-urban, and pro-educated inequalities. Child height-for-age *z* score (HAZ) decomposition explained >100% of predicted change in mean HAZ between 2000 and 2016, with key factors including increases in total consumable crop yield (32% of change), increased number of health workers (28%), reduction in open defecation (13%), parental education (10%), maternal nutrition (5%), economic improvement (4%), and reduced diarrhea incidence (4%). Policies and programs that were key to stunting decline focused on promoting rural agriculture to improve food security; decentralization of the health system, incorporating health extension workers to improve rural access to health services and reduce open defecation; multisectoral poverty reduction strategies; and a commitment to improving girls’ education. Interviews with national and regional stakeholders and mothers in communities presented improvements in health service access, women and girls’ education, improved agricultural production, and improved sanitation and child care practices as drivers of stunting reduction.

**Conclusions:**

Ethiopia's stunting decline was driven by both nutrition-specific and -sensitive sectors, with particular focus on the agriculture sector, health care access, sanitation, and education.

## Introduction

Chronic malnutrition in early childhood leads to stunting, which can affect the mental and physical development of the child, and can result in the intergenerational transfer of malnutrition along with poor birth outcomes in the next generation (1, 2). Stunting is a marker of inadequacy of the environment in which a child was born and raised, and it is associated with learning difficulties and barriers to community participation (3, 4). Because of this, stunting prevalence and severity is a good indicator to use for population assessment (4) and can be used to monitor the progress of children in a population over time. In 2018 there were 149 million stunted children globally, which represents nearly 22% of all children <5 y old (5).

**FIGURE 1:**
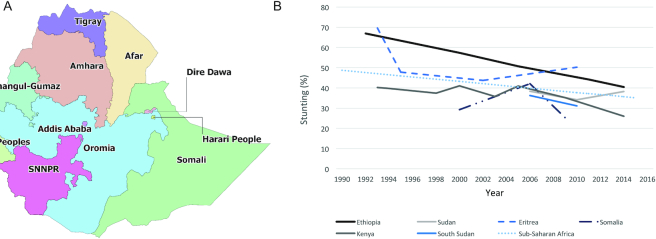
Map of Ethiopia and regional under-5 stunting prevalence. (A) Ethiopia map. (B) The prevalence of under-5 stunting in Ethiopia and its neighboring countries in the East Africa region, 1990–2015. SNNPR, Southern Nations, Nationalities, and Peoples’ Region.

Ethiopia is a landlocked country located in Eastern Africa with a population of >108 million (**Figure 1**A). A predominantly agricultural country, >80% of Ethiopians live in rural areas (6, 7). Stunting prevalence in Ethiopia has been declining steadily since the early 1990s (Figure 1B). Compared with neighboring countries, Ethiopia's stunting decline was the steadiest and demonstrated the largest decline, although prevalence was not the lowest in the region. Ethiopia has consistently had a higher stunting prevalence than the regional average; however, the gap has been narrowing over time.

Ethiopia has a diverse terrain spanning plateaus, mountain ranges, and plains, with the highlands and lowlands divided by the Great East African Rift Valley (8). The highlands of Ethiopia are well-suited for settlement because they are dense in moist forests and wetlands. In these areas, there is opportunity for agricultural growth; however, the majority is rain-fed and thus dependent upon good and consistent weather (9). Ethiopia's lowlands, however, are warm and dry, resulting in a lack of biodiversity compared with other regions of the country (6). Arid or semi-arid areas cover ∼40% of the country where agricultural production can be low, which can lead to poor food availability and nutrition security (9).

Ethiopia regularly experiences climate shocks, which can dramatically affect malnutrition levels. In 2011–2012, a severe drought in the Horn of Africa peninsula affected 4.5 million people, and specifically the Afar, Somali, and Oromia regions of Ethiopia (10). The country is also highly susceptible to earthquakes, volcanic eruptions, and flooding due to the geologically active Great Rift Valley, which can result in food insecurity and water shortages, particularly among pastoral and agro-pastoral communities (8, 10, 11).

Ethiopia is composed of 14 major ethnic groups, with the Oromo and Amhara groups in the majority at 34% and 27%, respectively (8). Consequently, Oromia is the most populous administrative region, housing 37% of the population, followed by Amhara (23%) and the Southern Nations, Nationalities, and Peoples’ Region (SNNPR) (20%) (12, 13).

In 1990, the fertility rate in Ethiopia was 7.2 children/mother, which has since dropped to 4.2 in 2016 (14). The adolescent fertility rate has also plummeted from 118 births/1000 women aged 15–19 y in 1990 to 65 in 2016 (14). In 2000, 56% of Ethiopians lived on <$1.25/d, making it a country with one of the highest poverty rates in the world. By 2011, this figure had dropped to 31%, representing a remarkable decade of poverty reduction (15). Gross domestic product per capita in Ethiopia has risen from $652 in 1990 to $1730 in 2017, whereas the poverty headcount dropped from 67% in 1995 to 27% in 2015 (**Supplemental Appendix 1**). Literacy rates among all adults and adult females have been rising, reaching 39% and 29%, respectively, in 2007. The Gender Development Index, which rose from 0.74 in 2000 to 0.85 in 2017, indicates that the disparities between men and women have been decreasing over time (Supplemental Appendix 1). Access to improved water sources has risen from 25% in 2000 to 64% in 2016 and the practice of open defecation has fallen from 80% to 27% over the same time period (Supplemental Appendix 1).

Much literature exists on the reasons behind the stunting decline in Ethiopia, and findings point to several key drivers: parental education (16–33); health care interventions, such as delivery by a skilled birth attendant (SBA) (17, 34–36), antenatal care (16, 17, 26, 34, 37–44), and postnatal care (45, 46); improvements in water, sanitation, and hygiene (WASH), including access to improved water sources (21, 32, 37, 39, 40, 45, 47–58), reduced distance to fetch water (59), access to improved sanitation (17, 20, 21, 23, 27, 29, 39, 40, 45, 51, 60–63), and reductions in open defecation (19, 27); and child feeding indicators (19, 30, 37, 38, 60, 64–70) (**Panel 1**, **Supplemental Appendix 2**) (16–98, 99–118, 119–142). Although we found a wealth of national and subnational studies exploring factors related to stunting in Ethiopia, the overwhelming majority were cross-sectional in design which precludes causality.

Panel 1 Systematic literature review of stunting determinants

Our literature review identified several basic determinants of child stunting in Ethiopia including ethnicity, economic development, governance, regional variation, climate and food shocks, wealth index, women's empowerment, and parental education. Ethnicity was found not to be associated with stunting risk (35), although religion was identified by 4 studies as associated with stunting; however, they found conflicting evidence based on which religion conferred greater risk (22, 71–73). One study suggests that economic development in Ethiopia was associated with improved nutritional status and stunting reductions (20), whereas another suggests that good governance was positively associated with stunting decline (21). Various studies have identified regional differences in stunting and some found that the northernmost regions Tigray, Amhara, and Afar were generally associated with increased stunting prevalence (16, 20, 21, 23, 74–78). Climate change, namely rainfall, drought, and temperature change, does predict some of the variation in child stunting in Ethiopia (18, 76), whereas the limited evidence of the impact of food shocks on stunting outcomes tends to show a lack of association (78, 79, 80). Increased wealth was found to be positively associated with HAZ and thus negatively associated with stunting in a large number of studies (16, 17, 19–21, 23–27, 29, 31, 34, 37, 38, 47, 60, 66, 77, 81, 82, 83, 96), whereas some found no association (30, 48, 64, 84, 85). Women's empowerment was difficult to quantify, and studies examining this variable suggested that the impact of proxies such as households being headed by women (23, 25, 30, 32, 47), women's autonomy (85), and mothers’ employment (26, 37, 48, 86) had conflicting or no association with HAZ in Ethiopia. Parental education has been shown to be positively associated with HAZ and negatively associated with stunting in the vast majority of both national (16–21) and subnational studies (22–27, 29–32, 96, 103), although some studies found no association (22, 34, 38, 47, 48, 59, 64, 82, 86).

Nutrition-specific and -sensitive programs identified in the literature were the Community-based Nutrition Program (CBNP), Productive Safety Net Program (PSNP), and the Targeted Supplementary Food Program (TSF). Two studies on the CBNP found that it was associated with increase in length-for-age *z* score and decrease in stunting (87, 88). The PSNP, however, was not found to have had an impact on stunting outcomes, although perhaps follow-up was not long enough to show these impacts (89–91). An evaluation of the TSF also showed no changes in child stunting outcomes, although there were differences between the intervention and control groups at baseline that render this finding uncertain (92).

Among the underlying causes of stunting, the literature suggests that immunization, skilled birth attendance, antenatal care, postnatal care, vitamin A supplementation, health care access and utilization, household environment, feeding practices, and food security were contributing factors. The majority of studies in our review found that childhood immunizations have a protective effect against stunting outcomes (28, 39, 40, 50, 93–95), although some found no association (23, 38, 45, 51, 97, 98). Health care interventions such as delivery by an SBA (17, 34–36), antenatal care (16, 17, 26, 34, 37–44), and postnatal care (45, 46) have been found to be associated with reduced stunting outcomes in most studies, although some found no association (16, 19, 22, 27, 30, 35, 42, 45, 49, 60, 64, 71, 86). Vitamin A supplementation is a promising initiative shown to be associated with a decreased risk of stunting, although variation in findings exists (40, 60, 61, 67, 99–102). Studies examining access, distance or time to a health facility typically found that these were not strongly related to stunting (17, 32, 33, 41, 52, 83, 100, 101, 104). There is substantial evidence in the literature that rural residence confers a higher likelihood of stunting than urban residence (18, 20, 22, 25, 28, 31, 37, 39, 48, 53, 72, 77, 80, 85, 105–107), although some studies found no association (16, 21, 23, 26, 27, 32, 34, 37, 46, 60, 64, 68, 109), whereas 1 found that rural-living children were protected (24). Improvements in WASH indicators such as access to improved water sources (21, 32, 37, 39, 40, 45, 47–58), distance to fetch water (59), access to improved sanitation (17, 20, 21, 23, 27, 29, 39, 40, 45, 51, 60–63), and open defecation (19, 27) have been generally found to be positively associated with stunting outcomes although some did find nonsignificant results (16, 17, 19, 20, 22, 23, 26, 27, 29, 30, 33, 34, 36–38, 45, 47, 49, 52, 60–62, 66, 75, 82, 93, 94, 101, 104, 108, 109, 119–126, 128). Hygiene was difficult to quantify, and this indicator was not found to be associated with stunting outcomes (19, 66, 75). Household crowding was examined in a large number of studies, of which the majority found no association with stunting, although some did find that smaller family size was associated with lower stunting (20, 23, 30, 32, 40, 41, 50, 51, 55, 63, 74, 80, 93, 102, 105, 106, 124–126, 128–132). A variety of breastfeeding practices were measured, and evidence of the association between breastfeeding and child growth in Ethiopia is mixed, although several studies did note a positive impact (19, 29, 36, 38, 40, 43–45, 49, 50, 52, 55, 56, 60, 63, 64, 69, 71, 75, 77, 79, 83, 85, 95–97, 101, 102, 105, 110, 111, 120, 124, 125, 127, 128, 132–143). Nearly all of the studies that explored the relation between stunting outcomes and complementary feeding found that it is significantly associated with child growth (58, 64, 100, 112, 119, 133, 134, 136, 139, 141). A number of studies examined the impact of food security on growth and most found that household food insecurity was negatively associated with HAZ, or positively associated with stunting (35, 47, 52, 75, 80, 113, 114, 131–134).

Immediate causes of stunting decline were identified as dietary intake, disease, maternal characteristics (age, height, BMI, and parity), and child characteristics (age, gender, birth weight, birth order, and number of children in the household). Studies highlighted the significance of improved dietary intake, dietary diversity, and consumption of multiple micronutrients, especially for children in early infancy (19, 30, 69, 70, 37, 38, 60, 64–68). Two studies examined the effect of disease on stunting and found no association (19, 85), although studies that looked at anemia found a dose–response relation between anemia severity and stunting risk (23, 27, 37). Diarrhea was also found to be an important risk factor for stunting outcomes (19, 38, 115), as was fever (27, 38, 78, 115); however, most studies treated ARI as an outcome with stunting as a significant predictor, not the other way around, which is an area for future research (60, 67, 69, 116). Literature showed that, generally, maternal age was not associated with stunting risk (20–23, 31, 34, 37, 85), but that mothers of shorter height (17, 19, 20, 31, 34, 37, 38, 47, 66, 85) or those with lower BMI were found to be more likely to have stunted children (19, 22, 23, 27, 28, 31, 37, 47, 60, 64), whereas increasing interpregnancy interval and fewer births are associated with a reduction in stunting outcomes (21, 23, 26, 31, 32, 37, 38, 96). With regard to child characteristics, studies emphasize that as child age increases, so do stunting outcomes (19, 20, 22–27, 29–32, 38, 47, 60, 64, 67, 69, 84, 86, 117, 118). In addition, under-5 boys were found to be more likely to be stunted than girls ( 19–23, 25, 27–29, 31, 37, 38, 47, 60, 64, 67, 69, 77, 84, 85, 118), whereas low-birth-weight children were at an increased risk of stunting outcomes (19, 22, 27, 37, 38, 59, 60, 64, 67, 85). A significant association was not found between birth order and stunting outcomes (16, 19, 22, 25–27, 34) but the majority of studies that looked at the number of children in households found that as the number of children under-5 in a household rises, stunting risk does as well (20, 21, 23, 47, 64, 85).

The aim of this research was to conduct a systematic assessment of the determinants that have driven stunting reduction in Ethiopia from 2000 to 2016, specifically on a national, community, household, and individual level. The 3 objectives included *1*) to quantitatively examine determinants of stunting reduction in Ethiopia and to decompose long-term stunting change into relative contributions from key drivers; *2*) to explore national- and community-level perspectives on the nutrition evolution (focused on progress in stunting) in Ethiopia and the major contributing factors behind it; and *3*) to generate a systematic landscape of the major stunting-relevant policies and programs in Ethiopia, with a focus on both nutrition-specific and -sensitive initiatives.

## Methods

### Study design

**FIGURE 2:**
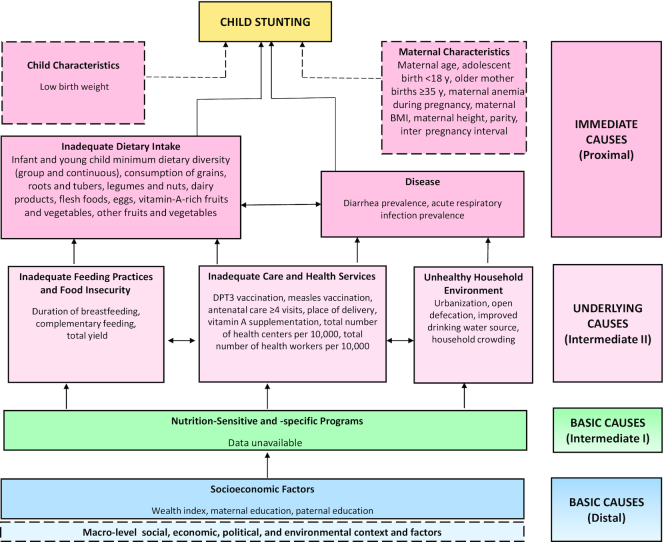
Conceptual framework showing distal, intermediate, and proximal determinants of stunting in Ethiopia. Framework reflects only indicators that were measurable and available for quantitative analysis. Skilled birth attendance was omitted because estimates were not available for the first year of study, and were only available for the last 2 y studied (2011 and 2016). Flesh foods refers to meat, fish, poultry, and liver/organ meats. Data on early initiation of breastfeeding were also unavailable and were omitted. Other variables included in analyses were child age in months, child sex, child weight, and child height. DPT3, 3 doses of Diphtheria-tetanus-pertussis vaccine.

This study involved 4 methods of inquiry including a systematic literature review, retrospective quantitative data analysis, qualitative data collection and analysis, and policy and program analyses. The time period covered was from 2000 to 2016, at three 5-y intervals (2000–2005, 2005–2011, 2011–2016). A conceptual framework was adapted from the 1995 UNICEF Nutrition Framework and the 2008 Lancet Nutrition framework, and informs the quantitative and qualitative data analyses. This framework identifies the key distal-, intermediate-, and proximal-level factors that potentially contributed to Ethiopia's stunting decline (**Figure 2**). Ethics approval for the study, inclusive of primary data collection, was obtained through the University of Addis Ababa's research ethics process. Ethics approval for the broader stunting case study was also obtained through the Research Ethics Board at the Hospital for Sick Children (SickKids), in Toronto, Canada.

### Systematic literature review

The systematic literature review resulted in 10,789 total articles being found, and consisted of a search for peer-reviewed literature, as well as gray literature, published between 1990 and 2019. Search terms used included “stunting” or “linear growth” or “linear growth stunting” or “HAZ” or “height” or “height-for-age” or “LAZ” or “length” or “length-for-age” or “undernutrition” or “malnutrition” or “nutr*” AND “child*” or “infan*” AND “Ethiopia*.” The initial database search found 10,789 records, and after duplicates were removed and records were screened, a total of 150 were included in our analysis, of which 5 were systematic reviews, 124 were quantitative analyses, and 21 were gray literature. More detailed information on the search terms, as well as the literature review flow diagram, can be found in Supplemental Appendix 2.

### Quantitative methods

#### Data sources

The sources of data used for the quantitative analyses were 4 Demographic and Health Surveys (DHSs) from 2000, 2005, 2011, and 2016. **Table 1** shows the sample size breakdown of under-5 children with available anthropometry data for these 4 surveys.

**TABLE 1:** Sample size by survey based on the index child with valid anthropometric data

	Year of Demographic and Health Survey			
Age group	2000	2005	2011	2016
<5 y	5975	2600	6561	6184
<36 mo	4623	1980	4998	4800
6–23 mo	2470	1113	2615	2620
≥24 mo	2697	1164	2978	2640
<6 mo	808	323	968	924

#### Outcomes and covariables

The main study outcomes included child height-for-age *z* score (HAZ) and stunting prevalence (HAZ < −2SD), as they were estimated from the WHO child growth standards (144). In line with Figure 2, covariables were selected as they were available in DHSs (individual/household variables) and the Agricultural Sample Survey conducted by Ethiopia's Central Statistical Agency (ecological variables at district level). We grouped potential stunting determinants hierarchically as distal, intermediate, and proximal factors that align with “basic causes,” “underlying causes,” and “immediate causes” in Figure 2.

#### Statistical analysis

Child HAZ kernel density plots were estimated for all survey years in order to examine population shifts in growth faltering over time. “Victora curves” or child HAZ-against-age plots were calculated using smoothed local polynomial regressions to enable examination of the growth faltering process from birth to 60 mo (145). We used piecewise linear splines to estimate the slopes and inflection points of the Victora curve growth trajectories (146). Equity analysis was conducted using standardized and well-established methods in order to study stunting prevalence disaggregated by wealth quintile, maternal education, area of residence (urban compared with rural), and child gender (147, 148). Wealth quintiles were derived using principal components analysis of household asset data. We also calculated the slope index of inequality (SII) and concentration index (CIX) in order to measure absolute and relative socioeconomic inequalities, respectively (147, 148). To assess the relative change (decline) in stunting prevalence of Ethiopia's districts, we calculated compound annual growth rates (CAGRs).

A series of stepwise linear regression models and hierarchical modeling were used to conduct 2 sets of multivariable analyses between HAZ and distal-, intermediate- and proximal-level variables as suggested in the literature (149). The DHS 2000–2016 rounds were assembled into a panel data set to conduct difference-in-difference (DID) analysis with time × covariable interaction terms to understand if a change in a proposed predictor of HAZ led to a change in HAZ over the studied time period (150). Regression-based Oaxaca–Blinder decomposition methods were used to decompose the change in mean child HAZ between 2000 and 2016 into the covariables that drove this change. Full methods details are included in **Supplemental Appendix 3** and the methods article in this series (Akseer et al.). All analyses were conducted with Stata version 14.0 (StataCorp LLC) and accounted for survey design and weighting.

### Policy and program review

Key nutrition-specific and nutrition-sensitive policies and programs in Ethiopia were incorporated into a timeline using an iterative approach. Literature was identified through a systematic approach in order to suggest a timeline which was then shared with expert stakeholders for them to corroborate and provide insight. Any additional policy/program documents suggested by stakeholders were added to the second iteration of the timeline, and the process was repeated until consensus was reached. Further, rating was done by stakeholders and research team members to evaluate the relative importance of the policies/programs to the observed reduction in stunting.

### Qualitative methods

Qualitative research was conducted in 2019 using in-depth interviews with key informants at the national and community levels, and focus group discussions (FGDs) with mothers in communities. In-depth interviews were conducted with national stakeholders, including nutrition experts, representatives from multi- and bilateral international donor organizations, representatives of related ministries, and state agencies. Interviews with both national and community informants asked respondents to describe key nutrition-specific and -sensitive events in Ethiopia. National informant interviews also asked about successful factors and barriers to implementation. Community informant interviews with regional stakeholders, including teachers and health staff, explored how contextual factors such as changes in social and economic situations, access to key resources, and community-level changes to dietary intake affected child nutrition. FGDs with mothers who gave birth to children during 3 time periods (1987–1991; 1995–1999; 2011–2015) sought women's experiences, and perspectives regarding pregnancy, breastfeeding, child nutrition, and perceived drivers of change and general trends in children's nutrition. A total of 12 FGDs were conducted—with 3 age groups each in a rural and an urban area, in 4 districts of the SNNPR and Somali regions. Data generated in interviews and FGDs were analyzed using the UNICEF Nutrition Framework, the Lancet Nutrition framework, and the adapted framework for country case studies (Figure 2). These frameworks guided the qualitative analysis and interpretation of key determinants, contextual factors, and barriers and facilitators to nutrition-specific and -sensitive events. Responses from national- and community-level stakeholders were analyzed separately. Thematic analysis was used to explore key themes that emerged regarding stunting determinants. Full methods for qualitative data collection and analysis are available in **Supplemental Appendix 4**.

## Results

### Descriptive analyses

#### HAZ kernel density plots and Victora curves

**FIGURE 3:**
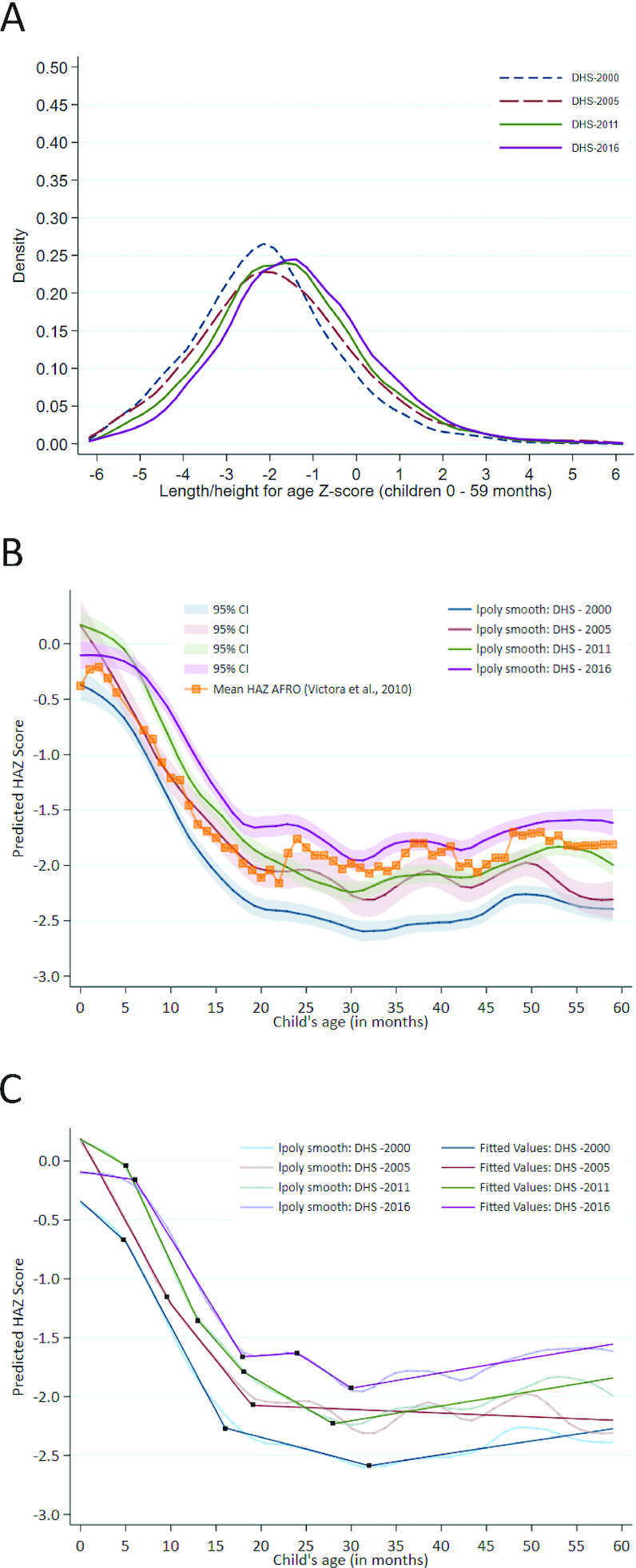
Distribution of HAZ scores and growth faltering among children under 5 y old. (A) Kernel density plot for HAZ distribution in children <5 y, DHS 2000, 2005, 2011, and 2016. (B) Victora curve using data from the 2000, 2005, 2011, and 2016 surveys among children <5 y, with 95% CIs (145). (C) Victora curve using data from the 2000, 2005, 2011, and 2016 surveys among children <5 y, with linear splines. DHS, Demographic and Health Survey; HAZ, height-for-age *z* score.

**Figure 3**A displays the HAZ kernel density plot with the HAZ distribution for children <5 y of age. The HAZ distribution from 2000 flattened out and underwent a parallel rightward shift to 2005. In comparing the 2000 and 2005 curves, the 2005 curve has a heavier right tail, indicating that more Ethiopian children are reaching a higher HAZ. By 2011, the curve shifted rightward somewhat and reached a higher peak. This indicates that the entire population of under-5 children had seen nutritional gains. This trend persisted into 2016 where the curve shifted further rightward and reached a slightly higher peak. The 2016 curve shows that by this year, more children were reaching the improved mean HAZ, which was also closer to that of the international reference population; however, the curve did flatten out, thus indicating that inequality remained present and widened slightly.

Figure 3B shows predicted child HAZ from smoothed local polynomial regressions plotted against child age for Ethiopian children for the 4 studied years, as well as an overlay of the growth faltering trends calculated from other countries in Africa. These curves allow for the examination of the growth faltering process from birth to 5 y of age.

Improvements in Ethiopian children's HAZ trajectory can be observed over the 16-y study period. The 2000 curve intercepts at the lowest point of all the years, suggesting that genetics and maternal nutrition throughout pregnancy may have been important markers of the child's size at birth. There was a decline in predicted HAZ in the first 6 mo of age, which may suggest that breastfeeding practices were inadequate, because this is the time in a child's development that exclusive breastfeeding is recommended to occur. A sharp decline can be observed during the growth faltering period between 6 and 24 mo of age. After 6 mo, food is being introduced to the child, and thus diet quality becomes a factor in their growth. The 2000 curve drops the lowest of all the curves during the growth faltering period, suggesting that children were consuming a poor diet and experienced unmet nutritional needs.

In 2016, the Victora curve intercepts just below the international reference population, which suggests that there had been improvements to maternal health or nutrition since 2000. The 2016 curve is markedly flatter in the first 6-mo period, indicating that infants were being breastfed and maintaining their growth during the first 6 mo of life. There remains a steep growth faltering period during which predicted HAZ declines sharply, although this decline does not reach 2011 levels. During the 16-y study period, it appears that improvements occurred in maternal health and nutrition, breastfeeding practices, and childhood nutrition.

The Victora curves were analyzed using piecewise linear regression to create linear splines which were fitted to the polynomial curves, with knots corresponding to major changes in the slope of the curve. These points suggest at which months in a child's development growth changes significantly. In 2000, the sharpest decline occurred between 5 and 16 mo of age at a rate of −0.14 SD/mo (Figure 3C; detailed coefficient estimates in **Supplemental Appendix 5**). By 2016, the period of greatest growth faltering was between 6 and 18 mo of age, at a slightly more moderate rate of −0.13 SD/mo. Despite fluctuations at 18 and 24 mo, the predicted HAZ in 2016 did not ever fall to stunted levels. Although the trajectories over time were similar, the absolute levels of HAZ were around half an SD higher in 2016 than in 2000.

#### Equity analysis

**FIGURE 4:**
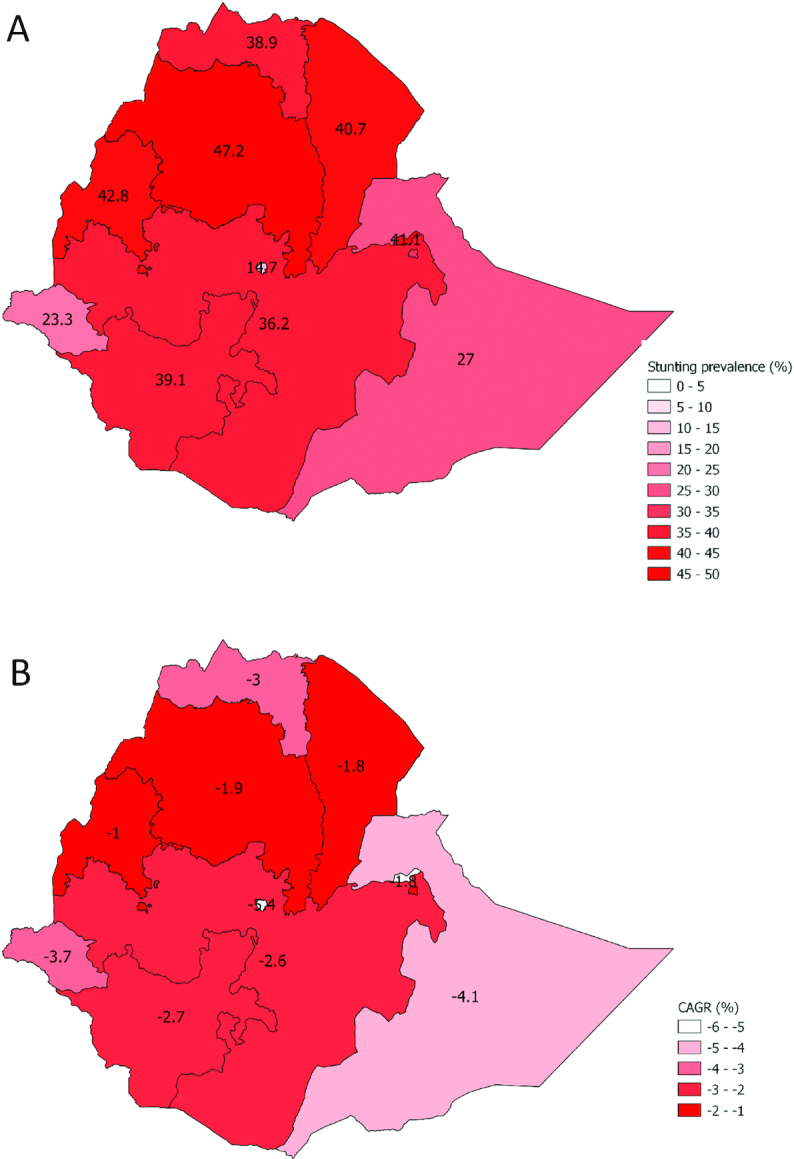
Subnational stunting estimates and rates of change in Ethiopia. (A) Subnational stunting estimates for children under-5 in Ethiopia, 2016. (B) Subnational stunting rates of change for children under-5 in Ethiopia. CAGR, compound annual growth rate; SNNPR, Southern Nations, Nationalities, and Peoples’ Region.

Stunting prevalence in Ethiopia declined from 51% in 2000 to 32% in 2016, although declines were not uniform and geographic disparities were large. Stunting prevalence was highest in Amhara and lowest in Addis Ababa in all 4 y studied (**Figure 4**A). Declines in stunting prevalence were observed in all but 1 region, and the CAGR was lowest for Addis Ababa, followed by Somali and Gambela, indicating that stunting declined most in these regions (Figure 4B). By 2016, these were the 3 regions with the lowest stunting prevalence at 15%, 27%, and 23%, respectively. Stunting prevalence in Dire Dawa increased over the studied time period, and the next highest CAGR was in Benishangul-Gumaz, which had the most modest declines in stunting. Regional stunting prevalence for other years can be found in **Supplemental Appendix 5**.

**FIGURE 5:**
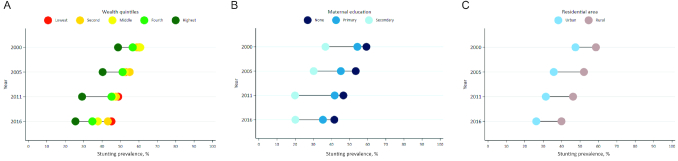
Stunting prevalence disaggregation by contextual factors. (A) Stunting prevalence disaggregated by wealth quintiles, 2000–2016. (B) Stunting prevalence disaggregated by maternal education, 2000–2016. (C) Stunting prevalence disaggregated by residential area, 2000–2016.

Stunting decline occurred across all wealth quintiles from 2000 to 2016; however, the gaps between rich and poor nearly doubled over this time period, because the wealthy experienced lower stunting prevalence and larger declines over time (**Figure 5**A). The SII and CIX plots show that both absolute and relative socioeconomic inequalities rose in Ethiopia (see **Supplemental Appendix 5**). Disaggregating by maternal education shows that mothers with higher levels of education had lower levels of stunting prevalence; however, the greatest stunting declines between 2000 and 2016 occurred among mothers with no or primary level of education (Figure 5B). Stunting prevalence was reduced for children living in both urban and rural areas. Slightly greater reductions occurred for children in urban areas, which also consistently experienced significantly lower stunting prevalence rates (Figure 5C). **Supplemental Appendix 5** graphs disaggregation by child sex, and shows that boys consistently had a slightly higher stunting prevalence than girls, and that disparities between boys and girls widened over time.

### Multivariable analyses

**FIGURE 6:**
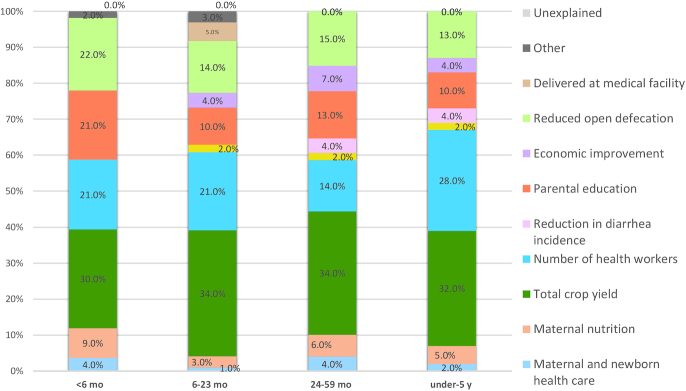
Decomposing predicted changes in HAZ (i.e., percentage contribution of determinant domains) from 2000 to 2016. Parental education breakdown: children 6–23 mo: maternal: 3.3%, paternal: 3.8%; children 24–59 mo: maternal: 7.5%, paternal: 0%; children under-5: maternal: 4.6%, paternal: 3.6%. For the 6- to 23-mo age category, delivery in medical facility is included in maternal and newborn health care (6.7%). Other category includes child age, gender, and region for all age groups in addition to maternal age (0.5%) for the 6- to 23-mo age group. HAZ, height-for-age *z* score.

Decomposition analysis reveals that the top 4 explanatory factors for stunting decline were the same across all 3 age groups (**Figure 6**). See **Supplemental Appendix 5** for supporting analyses. The top explanatory factor was increased total agricultural yield (between 32% and 34%), followed by increased number of health workers (14%–28%). Reductions in open defecation (13%–15%) and parental education (10%–13%) were the third and fourth explanatory factors for all age groups. Economic improvement also explained HAZ increases for all age groups, whereas reduction in diarrhea incidence contributed to only the ≥2 y and under-5 age groups. Maternal and newborn health care was most impactful for the 6- to 23-mo age group (6%), because it included a measure of delivery in a health facility. Maternal nutrition and fertility were also impactful for all age groups, and they explained between 2% and 6% of HAZ change.

The DID analyses (**Supplemental Appendix 5**) showed that delivery in a medical facility, number of health workers, and maternal education each contributed to HAZ change between 2000 and 2016.

### Policy and program review

**FIGURE 7:**
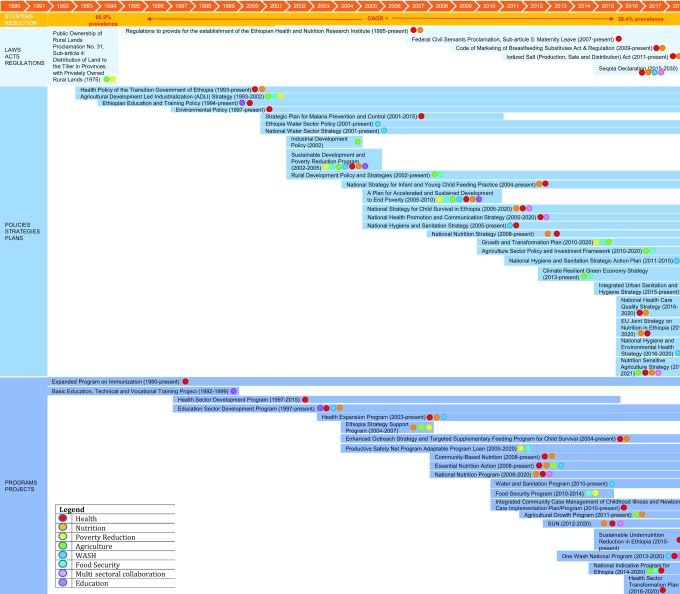
Overview of laws, policies, programs, and enablers 2000–2018 in Ethiopia. CAGR, compound annual growth rate; SUN, Scaling Up Nutrition Movement; WASH, water, sanitation, and hygiene.

**Figure 7** and **Panel 2** show a timeline of Ethiopia's key nutrition-specific and -sensitive programs and policies (151–153, 154–178). Ethiopia has introduced broad national development plans, as well as focused policies and programs that worked to address malnutrition and improvements in a child's environment more generally. Significant investments in agriculture-related policies and programs led to the strengthening of ties between agriculture and industry, particularly in rural areas, as well as improvements to the agricultural labor force and proper land use. Health system–strengthening programs bolstered by the 1993 Health Policy of the Transition Government led to the expansion of primary care, increased vaccine coverage, and the introduction of community health extension workers (HEWs). Nutrition-sensitive programs and policies related to poverty reduction, the WASH sector, and expansion of primary education, particularly for girls, have also been significant in explaining stunting change. **Supplemental Appendix 6** provides expanded information on each initiative.

Panel 2 Program and policy review

After Emperor Selassie's overthrow in 1974 after pervasive socioeconomic issues and famine in 1973, the Provisional Military Administrative Council introduced Proclamation 31 in 1975. This proclamation allowed any individual willing to cultivate land to be given land not exceeding 10 hectares so as to promote equality and economic development (154, 155). Other agriculture-related policies include the Agriculture Development Led Industrialization strategy (1993–2002) which represents Ethiopia's overarching strategic framework for development. It focuses on strengthening ties between agriculture and industry in rural areas so that incomes for rural families may rise and the nation can achieve food self-sufficiency (156, 157). The Industrial Development Policy followed in 2002 and continued to work toward agriculture-led industrialization, export-led development, and labor-intensive industries (157, 158). Also in 2002, the Rural Development Policy was introduced to minimize the need for foreign aid by developing Ethiopia's market economy through initiatives such as strengthening the agricultural labor force, proper use of land, and directing agricultural development (157, 158).

The Sustainable Development and Poverty Reduction Program (SDPRP) was a 3-y program beginning in 2002 with an aim to reduce poverty in Ethiopia. It aims to achieve this through numerous initiatives such as a focus on agriculture, strengthening private-sector growth, increased exports, particularly of agricultural products, and investment in education (159). Its successor, A Plan for Accelerated and Sustained Development to End Poverty (PASDEP), aimed to work toward achievement of the Millennium Development Goals. PASDEP ended in 2010 and its scope was wide, and affected agriculture, health care, water, and child nutrition (160, 161). After PASDEP, the Growth and Transformation Plan (2010–2020) shares the goals of its predecessors in improving economic growth and ending poverty. This plan aims to quickly and equitably drive economic growth by focusing on agriculture, expanding infrastructure, empowering women, and ensuring good governance (162, 163).

Ethiopia introduced many health and nutrition-related policies and programs over the study period. The Expanded Program on Immunization began in 1980 and continues into the present. It aims to accomplish 90% coverage of all vaccines nationally by 2020. In 1993, after the end of the Derg government, the Health Policy of the Transition Government of Ethiopia was created with a focus on women and children, the rural population, the poor, minorities, and victims of disaster (179, 164–166). The Health Sector Development Program (HSDP) was introduced years later in 1997 and provided comprehensive, integrated, and cost-effective primary care (151, 169, 169). The National Strategy for Child Survival in Ethiopia was introduced in 2005 and will continue until 2020 with the goal to achieve universal high-quality health coverage for mothers and newborns in communities and health facilities (167, 168). This strategy utilizes the Health Extension Workers (HEWs) to reach communities. The HEP emanated from the HSDP in 2003 and introduced HEWs to improve equitable access to health care in rural areas, despite resource limitations. HEWs focus on 4 major components: family health, disease prevention and control, hygiene and environmental sanitation, and health education and communication (152, 170–173). The Enhanced Outreach Strategy and Targeted Supplementary Feeding Program (2004) and the National Nutritional Policy and Strategy (2008) both address child nutrition, and continue into the present (153, 166, 174, 175).

Reductions in open defecation were an important factor in stunting decline in Ethiopia, with numerous policies addressing this important issue. Ethiopia's Health Policy of the Transition Government, adopted in 1993, had a focus on sanitation and open defecation (176). The HSDP, adopted in 1997, emphasized environmental health and communicable disease prevention, ensuring a safe environment (151, 169). The HEP had a clear focus on reducing open defecation and teaching about latrines; in fact, 5 of the 16 domains were related to safe environment (152, 170–173). Poverty reduction programs including SDPRP and PASDEP both had a focus on WASH, and the latter specifically promoted the use of latrines through the HEW program (160, 161). The National Hygiene and Sanitation Strategy (2005) also emanated from the HSDP, and itself produced the National Hygiene and Sanitation Strategic Action Plan (2011) (177, 178).

### Qualitative inquiry results

Across stakeholders, our qualitative analyses showed that health systems strengthening, improved access to health—and specifically reproductive, maternal, newborn, and child health—services, agriculture improvements and food security, breastfeeding promotion/improved knowledge of complementary feeding, disease prevention and management, social safety nets and poverty reduction programs, better access to education, and reductions in open defecation were named key drivers of child stunting reduction in Ethiopia. Full qualitative results can be found in **Panel 3** and **Supplemental Appendix 7**.

Panel 3 Qualitative inquiry results

**National expert stakeholders**

Interviews were conducted with 11 national key informants from various ministries in Ethiopia, and NGOs. Basic drivers of stunting decline as reported by national informants include increased urbanization, poverty reduction, improvements in education and women's empowerment, remittances and labor migration, and the decentralization and democratization process. This process helped regional states to improve agricultural productivity, infrastructure, and health services via a bottom-up process.

Nutrition-specific and -sensitive policies and programs discussed include programs related to community health, nutrition, poverty reduction, and health sector development. Barriers and facilitators of program and policy implementation were brought up, and key informants named the National Health Policy of 1993 as the primary guide for subsequent programs. Several key informants identified the HEP as the main driver of stunting decline in Ethiopia.

Underlying causes of stunting reduction discussed by national stakeholders include improvements to the household environment, food security and feeding practices, and access to health services. Improved sanitation and hygiene, specifically increased access to latrines, and major declines in open defecation were linked to the HEP:

“again thanks to the HEP a lot has been done regarding toilet utilization, especially in South and Tigray region there are open defecation free woredas.” (Ministry of Health representative)

Among the immediate causes of stunting decline are improvements in dietary intake, maternal characteristics such as fertility rate, and reductions in childhood illness such as malaria, vaccine-preventable diseases, and diarrhea.

**Regional stakeholders**

Regional stakeholders, including teachers and health staff (HEWs; maternal, newborn, and child health care workers; district health surveillance focal persons; and senior health center staff), were interviewed in depth across 2 regions and 4 districts. Basic drivers of stunting decline brought up by regional stakeholders include peace and security, improvements to education and women's empowerment, and poverty reduction.

A variety of nutrition-specific and -sensitive programs were discussed and implemented in community settings including the HEP, Mercy Corps, one WASH, ENGINE, PSNP, and the Pastoralist Community Development Program. The HEP was emphasized for resulting in substantial health and nutrition improvements.

Among the underlying causes of stunting reduction, interviewees discussed access to health services, WASH, and food security. The observed improvements in food security named by the regional stakeholders were related to increased agricultural production, as well as improved access to markets, availability of roads, and access to media.

Immediate causes of stunting reduction mentioned include dietary intake, infant and young child feeding, recommendations for optional breastfeeding and complementary feeding, increased contraceptive utilization, and declines in childhood communicable diseases including diarrhea, parasitic infection, typhus, and skin and eye infections. Key informants perceived limited access to animal source foods, but improved diversity of children's diets over time:

“Previously the dietary intake of people was poor. […] Children need different kinds of foods such as vegetables, fruits, rice, pasta, fish, and milk. Nowadays people consume various diets that is rich in many nutrients.” (Community informant, Somali region)

**Mothers in communities**

FGDs with mothers of children under-5 born during 3 time periods (1987–1991, 1995–1999, 2011–2015) were conducted in order to identify the factors that were salient in these time periods of substantial stunting decline. Poverty was mentioned as a concern by mothers of children in all 3 age groups in the Somali region; however, it was not brought up by mothers in SNNPR. Improvements in both boys’ and girls’ education, as well as improved infrastructure such as roads or housing, were brought up by mothers in both regions in the later years as distal drivers of stunting decline:

“Our view of girl education is not the same now and then. We used to think that a girl that goes out early to learn was a bad girl. […] Now we educate both genders equally.” (Mother FGD Aware, Somali, 1995–1999)

Neither mothers in the Somali region nor those in SNNPR brought up any nutrition-specific and -sensitive policies, likely because they were not prompted on these and they may not have been familiar with specific government initiatives. Mothers in Somali were concerned about drought and water shortage in all years, and the price of food in later years. Mothers in both regions brought up access to a safe water supply as concerning as well. Improved hygiene and sanitation, and health service access were mentioned by mothers in both regions in the latest group, whereas mothers in SNNPR in the latest group also spoke about improved crop production.

Immediate causes of stunting decline spoken about by mothers in both the Somali region and SNNPR in the later years include improved child vaccination, and a decreased concern about childhood illness because health services were readily available. Mothers in the SNNPR region also brought up improved access to fruits and vegetables and improved child care in later years.

## Discussion

### Summary

Our mixed-methods case study suggests a variety of contributors to under-5 stunting decline, including targeted nutrition-sensitive and -specific policies and programs, that led to improvements in underlying and immediate causes of child malnutrition. Key national investments in agriculture, sanitation, health, education, and poverty reduction have been essential to achieving these gains.

Numerous agriculture-focused policies allowed individuals to cultivate land, improve crop output, increase economic opportunities, and reduce food insecurity, particularly in rural areas. Health sector development policies combined with the proliferation of facilities and health workers in communities led to increased access to health services, improved knowledge through nutritional advice, and reductions in open defecation. This availability of health knowledge, combined with improved women's empowerment through increased girls’ schooling, allowed mothers to make more informed decisions around family planning and child feeding practices. Multisectoral poverty reduction programs contributed to economic improvement and affected agriculture, health care, water and sanitation, women's empowerment, and child nutrition.

Triangulating evidence from our qualitative analyses, quantitative analyses, policy review, and systematic literature review showed that the aforementioned themes recur as major contributors to stunting decline in Ethiopia. Underlying this was the government's push toward decentralization and democratization, with a focus on the country's large rural population to provide equitable services and reach development goals.

### Study strengths and limitations

Our study has 5 key strengths. First, to our knowledge, it is the first systematic mixed-methods analysis of the major determinants of stunting in Ethiopia, using a multitude of research methods in order to draw a holistic picture of the country's stunting situation. Second, we used a comprehensive set of variables in order to explore a range of determinants at all levels of the conceptual framework. Third, our quantitative analyses incorporated a hierarchical modeling approach such that we were able to build appropriate modeling pathways between potential stunting determinants that adjusted for confounders and let us examine mediators. Fourth, our qualitative analyses captured diverse and multilevel perspectives, with 12 FGDs with mothers and interviews with both community and national stakeholders. This allowed for varied opinions on the tangible changes in socioeconomic status, local infrastructure, behavior, health, and nutrition over time. Finally, our study was the first to systematically compile all nutrition-relevant published and unpublished literature and policy analysis on Ethiopia to date.

Our study also had several limitations. First, our qualitative data collection, transcription, and analysis were conducted primarily in the local language, which may have contributed to points being lost in translation. Interviews and FGDs could have been affected by recall bias, especially for mothers of children born in the earliest time period (1987–1991). Second, our study uses Oaxaca–Blinder decomposition analysis and, as such, previously cited limitations of this method apply (17, 180, 181). Third, although we adjusted for confounding variables in our quantitative analyses, residual confounding may remain from unmeasured confounders or poorly estimated variables. Fourth, mapping of financial data pertaining to nutrition-specific and -sensitive initiatives in Ethiopia may be incomplete, because data were obtained from stakeholders and published literature only, and were scarce. Lastly, measures of certain variables such as food insecurity, intrauterine growth, and dietary intake for children under-5 were lacking, requiring the use of proxy variables where appropriate and available.

### Existing evidence

This study of Ethiopia's stunting decline reveals a multidimensional story incorporating contextual factors in conjunction with targeted policies and programs that worked to improve the underlying and immediate causes of child malnutrition.

Stunting in Ethiopia declined by 18.5% points, with mean HAZ rising significantly between 2000 and 2016. Improvements in health, nutrition, and stunting were achieved in spite of ongoing ethnic clashes, conflicts with neighboring countries, the Ethiopian–Eritrean war, and climate shocks including major droughts in 2002–2003 and 2011 leading to a high level of food insecurity. Advances were achieved through reactive implementation of poverty alleviation strategies, including social protection and resettlement programs. In addition, agriculture-related policies and programs contributed to improved crop yields, reducing household food insecurity and bolstering economic improvements, especially among Ethiopia's rural population.

Our study identified several main drivers of stunting reduction, namely, improved agricultural production, health system strengthening and reforms, reductions in open defecation, poverty reduction, and improvements in education, all of which would not have been possible without strong government support and buy-in. Agriculture was a major contributing factor to improved child HAZ in Ethiopia, and has been a driving force behind the increased economic and social stability in the country. Beginning with the Public Ownership of Rural Lands Proclamation in 1975, and continuing with the Agricultural Development Led Industrialization Strategy (1993–2002), the Industrial Development Policy (2002), and the Rural Development Policy and Strategies (2002–present), the Ethiopian government has invested heavily in the agricultural sector. These investments appear to have paid off, because in 2018, the agricultural sector employed 60% of all employed women and 72% of employed men (182). The production of staple crops has risen steadily since 1990 across all regions, as has production of fruits, vegetables, and animal-source products (183, 184). Increased crop yield was the top explanatory factor in our decomposition analyses, and improved diet diversity and availability of fruits and vegetables were noted by both stakeholders and mothers. Although our literature review did not allow for definitive conclusions, there was evidence that HAZ change in Ethiopia could be explained in part by climate and food shocks (76, 78, 80), and that environmental disruptions are associated with lower HAZ.

Ethiopia's health system has undergone a number of reforms which resulted in marked improvements in health and reductions in stunting among children under-5. Health services were decentralized, allowing local governments to place emphasis on health service availability in rural communities and expand primary care provision. Policies and programs including the Expanded Plan on Immunization, the Health Policy of the Transition Government of Ethiopia, the Health Sector Development Program, and the Health Extension Program (HEP) served to spread the provision of community-based health services and expand preventative health interventions including immunization, WASH initiatives, and advice on nutrition and infant feeding practices (151, 164–166, 169–173, 176, 185). Our quantitative analysis showed that the total number of health workers and facilities increased, as did deliveries at a medical facility. Our decomposition analysis demonstrated that the increases in maternal and newborn health care and in the number of health workers positively contributed to increases in HAZ. This finding is corroborated by our qualitative research, wherein respondents consistently spoke about expansion of health facilities and increased coverage of basic health services as contributors to stunting decline. In addition, the literature indicates that skilled birth attendance and antenatal care are both negatively associated with stunting outcomes in Ethiopia.

Open defecation was identified by our decomposition analysis as the third most important factor in explaining HAZ change in children under-5, and reductions in diarrhea also contributed to this change. These improvements were likely linked to community-led sanitation programs that fell under the umbrella of the HEP. National and regional stakeholders identified these programs as the reason for increased construction of pit latrines and community engagement in healthy WASH practices taught by HEWs. Additional programs, including the National Hygiene and Sanitation Strategy and Action Plan and the Community-led Total Sanitation Program, aided in open defecation reduction and, ultimately, open defecation–free communities (186, 187). Interviews with mothers in Ethiopian communities corroborated these achievements, because mothers of children born in later years discussed improved hygiene and sanitation. Despite these achievements, safe water access remains an outstanding issue for many Ethiopians—a finding that was noted by interview respondents and highlighted by our quantitative data.

With support from the literature on the role of improved wealth on increased HAZ (16, 17, 19–29, 31, 34, 37, 47, 60, 77, 81, 82), our study found that poverty reduction and economic growth were key drivers of stunting reduction in Ethiopia. Ethiopia's economy grew significantly over the last 2 decades, and the country aims to reach lower middle income status by 2025 (188). National experts, community stakeholders, and mothers expressed in interviews that improvements in agricultural yield, employment opportunities, and government support programs drove these economic gains and were related to improvements in stunting. A national priority in Ethiopia, poverty reduction programs introduced by the government include the Sustainable Development and Poverty Reduction Program, the Plan for Accelerated and Sustained Development to End Poverty, and the Growth and Transformation Plan. Our decomposition analyses show that improvements in household wealth explained a portion of HAZ change for all age groups studied. However, equity analyses revealed that improvements did not occur uniformly, because stunting prevalence reduction favored the wealthy across all time periods, and both relative and absolute inequalities increased over time.

Finally, improvements in education have been a driver of stunting decline. The percentage of literate women rose between 2000 and 2016, as did the percentage of women with secondary or higher education. Our equity analyses provide some pathways by which child HAZ was associated with maternal education, namely that compared with mothers with no education, those with some education had increased rates of care seeking for diarrhea and acute respiratory infection (ARI), improved vaccination rates, greater piped water access, and reduced open defecation. Decomposing HAZ change in Ethiopia over the study period, we found that parental education accounted for a portion of the change observed. The literature supports our findings, because both maternal and paternal education have been found to be positively associated with HAZ (16–21, 23–26, 28, 30, 32, 103). National and regional stakeholders, as well as mothers in SNNPR and Somali, corroborated the improvements to education, particularly of girls and women, and the accompanying advances in child care and nutritional knowledge. Programs that have helped achieve these gains include the Ethiopian Education and Training Policy (189) and the Education Sector Development Program (190) which seek to ensure universal primary education, improved access, and, more recently, quality of education.

### Remaining challenges and future research

Although stunting prevalence at the national level has declined substantially, regional variations remain. Certain regions, especially those in the north such as Amhara, continue to have a very high stunting prevalence. Dire Dawa in particular increased stunting prevalence over time; this may be due to persistent droughts, land shortages and degradation, shortage of water for irrigation, lack of political will for enhancing agricultural practices (191–193), lower levels of education and higher fertility (192), and generally poor economic indicators relative to other regions (192). Government must prioritize the extension of effective programs to Dire Dawa and other regions with high stunting to accelerate stunting reduction at the national level. Children from poorer households, in rural areas, and who have uneducated mothers continue to have disproportionately higher stunting prevalence. Future nutrition strategies should take on more targeted approaches that focus on these disadvantaged groups.

Child underweight, overweight, and wasting prevalence also declined over our study period, although not uniformly. Regional variations existed among these nutritional indicators as well, with Afar and Benishangul-Gumaz having a child underweight prevalence of 35% in 2016, whereas in Addis Ababa, only 5% of children were underweight. In 2016, Somali was the region with the highest wasting prevalence, at 23%, whereas overweight was highest in Addis Ababa at 7%. Whereas maternal short stature remained <5% over our study period, maternal anemia was persistently high, particularly in the Somali region at a staggering 60%. Maternal underweight also varied by region and was highest in Afar in 2016 at 38%, and lowest in Addis Ababa at 13%. These remain challenges and areas for future research as Ethiopia continues to work toward improving maternal and child nutrition.

### Conclusion

Through political will, leadership, and prioritization of nutrition, this study of stunting decline in Ethiopia shows a multifactorial story of change. Nutrition-specific and -sensitive approaches focused on the agricultural sector to increase household food security and decrease poverty, the health sector to increase service utilization and health care access, and multisectoral efforts to improve access to sanitation and reduction in open defecation.

## Supplementary Material

nqaa163_Supplemental_Files

## Notes

Funded by a grant to the Centre for Global Child Health from Gates Ventures. The funder had no role in the design, implementation, analysis, or interpretation of the data.

Supplemental Appendices 1–7 are available from the “Supplementary data” link in the online posting of the article and from the same link in the online table of contents at https://academic.oup.com/ajcn/.

Data described in the article, code book, and analytic code will be made available upon request pending application and approval.

AA to JW authors organized alphabetically.

Published in a supplement to the *American Journal of Clinica lNutrition*. The Guest Editor for this supplement was Mark Manary, and has no disclosures. Publication costs for this supplement were defrayed in part by the payment of page charges by Gates Ventures. The opinions expressed in this publication are those of the authors and are not attributable to the sponsors or the publisher, Editor, or Editorial Board of the*American Journal of Clinical Nutrition*. The Supplement Coordinator for the supplement publication was Nadia Akseer, Gates Ventures/Hospital for Sick Children, Toronto, Canada. Supplement Coordinator disclosure: no conflicts to disclose. The Stunting Exemplars research Principal Investigator was Zulfiqar A Bhutta, Hospital for Sick Children, Toronto, Canada. Principal Investigator disclosure: no conflicts to disclose.

Abbreviations used: ARI, acute respiratory infection; CAGR, compound annual growth rate; CBNP, Community-based Nutrition Program; CIX, concentration index; DHS, Demographic and Health Survey; DID, difference-in-difference; FGD, focus group discussion; HAZ, height-for-age *z* score; HEP, Health Extension Program; HEW, health extension worker; HSDP, Health Sector Development Program; PASDEP, A Plan for Accelerated and Sustained Development to End Poverty; PSNP, Productive Safety Net Program; SBA, skilled birth attendant; SDPRP, The Sustainable Development and Poverty Reduction Program; SII, slope index of inequality; SNNPR, Southern Nations, Nationalities, and Peoples’ Region; TSF, Targeted Supplementary Food Program; WASH, water, sanitation, and hygiene.
