# Supplementary material for: Drivers of stunting reduction in Ethiopia: a country case study
Source: Am J Clin Nutr. 2020 Aug 25;112(Suppl 2):875S–893S. doi: 10.1093/ajcn/nqaa163 (PMC7487434; doi:10.1093/ajcn/nqaa163)
Supplement: nqaa163_Supplemental_Files [file nqaa163_supplemental_files.zip › Ethiopia Stunting Online Supplementary Appendices May182020.docx]

Drivers of stunting reduction in Ethiopia: A country case study

Authors: Hana Tasic^,^ Dr. Nadia Akseer, Dr. Seifu Hagos Gebreyesus, Dr. Anushka Ataullahjan, Samanpreet Brar, Erica Confreda, Kaitlin Conway, Dr. Bilal Shikur Endris, Muhammad Islam, Dr. Emily Keats, Afrah Mohammedsanni, Jannah Wigle, Zulfiqar A Bhutta

**Online Supplementary Material**

**List of Appendices**

[**Supplementary Appendix 1:** Descriptive Analysis of Contextual Factors 4](#_Toc41034139)

[**Supplementary Appendix Figure 1A:** Trends in GDP per capita, poverty and urbanization, 1990, 2017 4](#_Toc41034140)

[**Supplementary Appendix Figure 1B:** Trends in key contextual indicators, 1990, 2017 4](#_Toc41034141)

[**Supplementary Appendix 2:** Systematic Literature Review 5](#_Toc41034142)

[**Supplementary Appendix Figure 2:** Literature review flow diagram 6](#_Toc41034143)

[**Supplementary Appendix 3:** Multivariable Analyses Methods 37](#_Toc41034144)

[**Supplementary Appendix 4:** Qualitative Data Collection and Analyses Methods 39](#_Toc41034145)

[**Supplementary Appendix Table 1**: Inclusion Criteria 40](#_Toc41034146)

[**Supplementary Appendix Figure 3:** Regional map of Ethiopia displaying study sites for subnational key informant interviews and focus group discussions. 41](#_Toc41034147)

[**Supplementary Appendix 5:** Quantitative Results 43](#_Toc41034148)

[**Supplementary Appendix Figure 4A:** Spline analysis of inflection points of change in the slope of HAZ, 2000 43](#_Toc41034149)

[**Supplementary Appendix Figure 4B:** Spline analysis of inflection points of change in the slope of HAZ, 2005 43](#_Toc41034150)

[**Supplementary Appendix Figure 4C:** Spline analysis of inflection points of change in the slope of HAZ, 2011 44](#_Toc41034151)

[**Supplementary Appendix Figure 4D:** Spline analysis of inflection points of change in the slope of HAZ, 2016 44](#_Toc41034152)

[**Supplementary Appendix Figure 5A:** 2000 stunting prevalence by region 45](#_Toc41034153)

[**Supplementary Appendix Figure 5B:** 2005 stunting prevalence by region 45](#_Toc41034154)

[**Supplementary Appendix Figure 5C:** 2011 stunting prevalence by region 46](#_Toc41034155)

[**Supplementary Appendix Figure 6A:** Change in absolute SII by year in Ethiopia 46](#_Toc41034156)

[**Supplementary Appendix Figure 6B:** Change in relative CIX by year in Ethiopia 47](#_Toc41034157)

[**Supplementary Appendix Figure 7:** Stunting prevalence disaggregated by child sex, 2000-2016 48](#_Toc41034158)

[**Supplementary Appendix Table 2:** Descriptive trends in stunting determinants in 2001-2016 in children <5 years 48](#_Toc41034159)

[**Supplementary Appendix Table 3:** Decomposition analysis for children among 6-23 months from 2000-2016 54](#_Toc41034160)

[**Supplementary Appendix Figure 8:** Decomposing predicted changes in HAZ among children 6-23 months (i.e. relative ranking of product coefficients for determinant domains) from 2000-2016 55](#_Toc41034161)

[**Supplementary Appendix Table 4:** Decomposition analysis for children 24-59 months from 2000-2016 55](#_Toc41034162)

[**Supplementary Appendix Figure 9:** Decomposing predicted changes in HAZ among children 24-59 months (i.e. relative ranking of product coefficients for determinant domains) from 2000-2016 56](#_Toc41034163)

[**Supplementary Appendix Table 5:** Decomposition analysis for under-5 children from 2000-2016 56](#_Toc41034164)

[**Supplementary Appendix Figure 10:** Decomposing predicted changes in HAZ among children under-5 (i.e. relative ranking of product coefficients for determinant domains) from 2000-2016 57](#_Toc41034165)

[**Supplementary Appendix Table 6:** Difference-in-differences multivariable regression for children under-5 years from 2000 – 2016 58](#_Toc41034166)

[**Supplementary Appendix Table 7:** Difference-in-differences multivariable regression for children 24-59 months from 2000 – 2016 69](#_Toc41034167)

[**Supplementary Appendix Table 8:** Difference-in-differences multivariable regression for children 6-23 months from 2000-2016 75](#_Toc41034168)

[**Supplementary Appendix 6:** Programs and Policies 86](#_Toc41034169)

[**Supplementary Appendix Table 9:** Detailed timeline of nutrition-specific and –sensitive laws, policies and programs in Ethiopia 86](#_Toc41034170)

[**Supplementary Appendix 7:** Qualitative Results 102](#_Toc41034171)

[**Supplementary Appendix Table 10**: Description of National Key Informants 102](#_Toc41034172)

[**Supplementary Appendix Table 11:** Summary of Regional Stakeholders 119](#_Toc41034173)

[**Supplementary Appendix Table 12:** Summary and comparison of contextual, underlying and immediate factors elicited across mothers of children born in three time periods, Ethiopia 130](#_Toc41034174)

[**Supplementary Appendix References** 141](#_Toc41034175)

# **Supplementary Appendix 1:** Descriptive Analysis of Contextual Factors

## **Supplementary Appendix Figure 1A:** Trends in GDP per capita, poverty and urbanization, 1990, 2017

## **Supplementary Appendix Figure 1B:** Trends in key contextual indicators, 1990, 2017

# **Supplementary Appendix 2:** Systematic Literature Review

**Methods**

A systematic search of published peer-reviewed and grey literature related in Ethiopia was undertaken in order to synthesize information on contextual factors, national and subnational interventions, policies, strategies, programs, and initiatives that may have theoretically contributed to reductions in child stunting in Ethiopia over time. Three broad categories of search terms were used: stunting, child, and Ethiopia. Keywords representing these terms were combined with Boolean operators, adapted with appropriate syntax, and executed in multiple databases. An example of a search syntax is provided below:

1. Stunting: "stunting" or "linear growth" or "linear growth stunting" or "HAZ" or "height" or "height-for-age" or "LAZ" or "length" or "length-for-age" or "undernutrition" or "malnutrition" or "nutr*"

2. Child: “child*” or “infan*”

3. “Ethiopia*"

4. 1 AND 2 AND 3

The search for indexed literature was conducted in 15 online databases: MEDLINE, Embase, AMED, CAB Abstracts, CINAHL, Cochrane CENTRAL, Campbell Collaboration, EPPI Centre Trials Register (TRoPHI), 3ie, JOLIS, African Journals Online, WHOLIS, LILACS, Scopus, and Web of Science. Additional searches for grey literature were conducted using Google, a hand search of reference lists of relevant reviews, and direct searching organizational websites, including: National, regional and headquarter websites for UNICEF, WHO, UNDP, WFP, FAO, World Bank Group Open Knowledge Repository, African Development Bank, Nutritional International, Global Alliance for Improved Nutrition, International Food Policy Research Institute, Growth Through Nutrition, Concern Worldwide, Alive and Thrive, Ethiopian Public Health Institute and the Government of Ethiopia including the Ministry of Health and the Ministry of Agriculture.

The exported set of records were de-duplicated and screened for relevance. Records were included if they met all of the following inclusion criteria:

i) included an under-5 population in Ethiopia;

ii) published between January 1, 1990- June 12, 2019;

iii) examined one or more of the determinants of chronic undernutrition (e.g. determinants, risk factors, policies, programs, interventions, or initiatives); and

iv) examined effects on child growth or a reduction in stunting

Initial database searches returned 10,789 records, which was reduced to 4,485 after de-deduplication. Applying the screening criteria to titles and abstracts left 147 records, which was then reduced to 102 upon full-text review. All included studies were categorized for the purposes of thematic exploration and mapping. Three broad categories of studies were identified: i) quantitative analysis of child health or nutrition outcomes; ii) qualitative studies with quantitative analysis; and iii) grey literature reports from non-governmental and multilateral organizations. See **Appendix Figure 2** for a flow diagram outlining the components and progression of the review. Relevant literature was iteratively synthesized and summarized to inform our research questions and to contrast our findings with existing evidence.

## **Supplementary Appendix Figure 2:** Literature review flow diagram


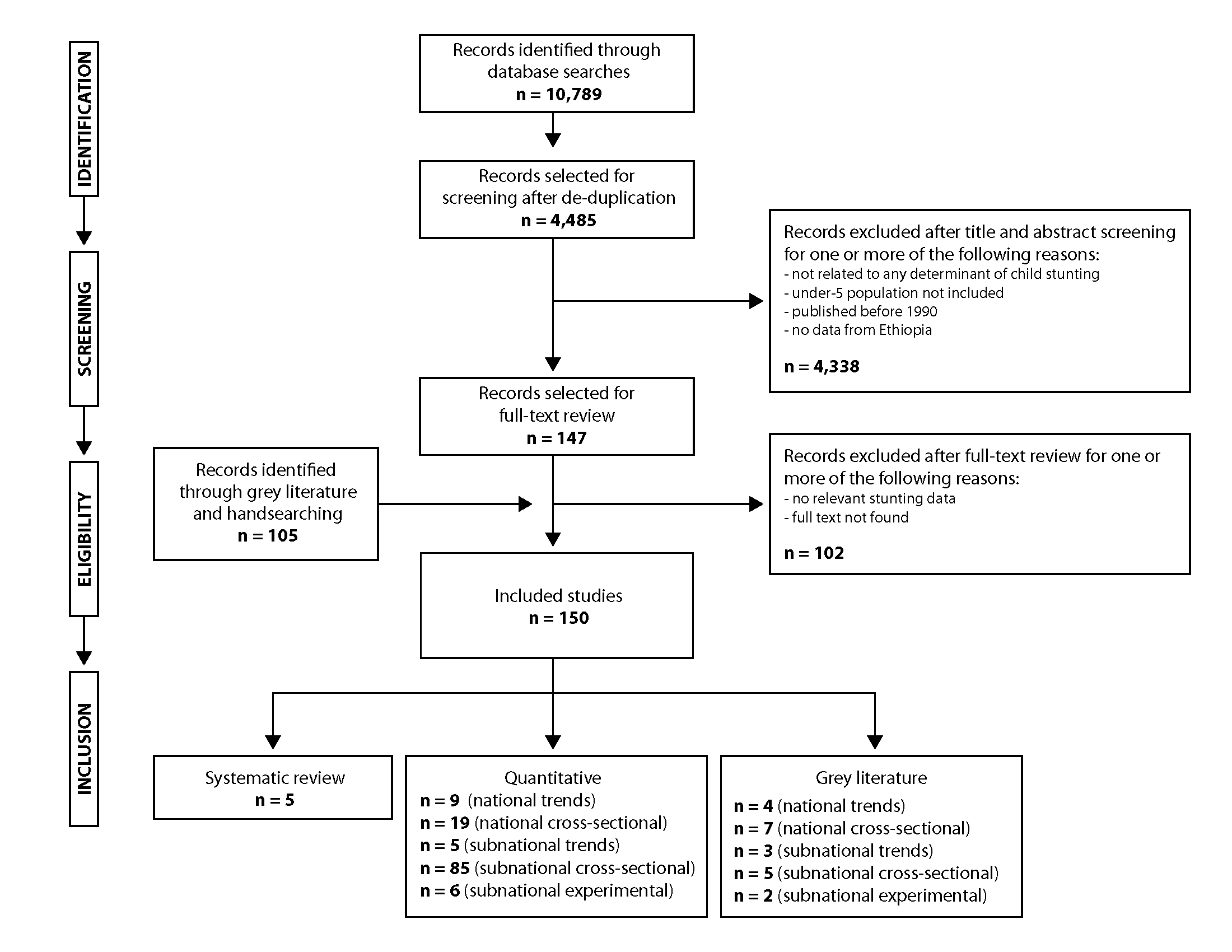


**Full Literature Review**

Stunting reduction in Ethiopia was researched by a multitude of teams, and we found many published papers that reported on factors impacting child growth. We present these findings in relation to an adapted version of UNICEF’s conceptual framework for malnutrition. Specifically, poverty, frequent climate shocks, low levels of maternal education, rampant open defecation, and lack of access to basic healthcare have contributed to chronic malnutrition among children under-5 in Ethiopia. Decentralization has allowed for greater access to healthcare and education, particularly among Ethiopia’s majority rural population. The proliferation of community health workers facilitated the spread of pit latrines and sharp decline in open defecation rates. Ethiopian children’s nutrition has also been improved by increased coordinated and multi-sectoral nutrition-specific and nutrition-sensitive policies and programs. In this literature review, we aimed to summarize data from studies that relate to the causes of chronic malnutrition in Ethiopia.

**Basic Causes (Distal)**

**Macro-level Factors**

**Social**

*Religion & Ethnicity*

Only a small handful of studies examined the association between Ethiopia’s religious groups and childhood stunting. For example, one recent cross-sectional study assessed community-level factors such as religion to see whether it can influence stunting risk. In this study, under-5 children from Catholic families were 41% more likely to be stunted and Muslim families were 33% more likely to be stunted compared to children from Protestant families (AOR=1.41, 95%CI: 1.01-1.97; AOR=1.33, 95%CI: 1.06-1.58, respectively) [1]. A non-significant association was found among children under-5 in Orthodox families (AOR=0.94, 95%CI: 0.72-1.23) [1]. Three other subnational-studies support the association between religion and stunting. One of these subnational cross-sectional studies followed children 6-23 months of Orthodox Ethiopian mothers and found that HAZ of children of non-fasting mothers (mean HAZ = -1.24 (SE = 1.34)) was significantly higher than children of the fasting sub-group (mean HAZ = -1.60 (SE = 1.35)) during the lent fasting period (p = 0.003) [2]. Lastly, a subnational study conducted in Ethiopia’s Metekele Zone of Benishangul-Gumuz region followed children 6-59 months and found that the odds of stunting among children of Muslim and Protestant families were 1.6 times and 4 times higher compared to children of Orthodox mothers (AOR: 1.61, 95% CI: 1.02-2.55; AOR: 4.27, 95% CI: 1.19-15.27, respectively) [3]. The same authors failed to find an association between stunting and Catholic children under-5 or those following traditional beliefs. One subnational study failed to find an association between under-5 stunting and Islam or other religions (AOR=1.05, 95%CI: 0.54-2.04) [4].

Only one study, a 2017 sub-national cross-sectional study by Hagos et al, examined the association between ethnicity and stunting in Ethiopia. For both stunting (Silte OR = 1.25; Bayesian credible interval [BCI] (1.00–1.54), Mareko OR = 1.35; Bayesian credible interval [BCI] (0.88–1.95), others OR = 0.97 Bayesian credible interval [BCI] (0.59–1.45) and severe stunting outcomes (Silte OR = 1.25; Bayesian credible interval [BCI] (1.00–1.54), Mareko OR = 1.35; Bayesian credible interval [BCI] (0.88–1.95), others OR = 0.97 Bayesian credible interval [BCI] (0.59–1.45), it was found that ethnicity was not significant [5].

**Key Takeaway**: Despite the limited number of studies, religion appears to play a differential role in stunting risk among children living in Ethiopia, with Muslim populations more often at a greater risk for stunting compared to other religions. Only one study examined the association between ethnicity and under-five stunting risk in Ethiopia, and although ethnicity was not found to be a significant predictor of stunting, further research needs to be done at a subnational or community level to support this finding.

**Economic**

One study included in this review explored the relationship between macro-economic factors and stunting outcomes. In a 2016 national trend study by Biadgilign et al, economic development was shown to be associated with improved nutritional status, and in particular to substantial reductions in stunting [β=-0.0016, SE=0.00013,p<0.0001]. It was also found that there was a negative correlation between stunting of children under-5 years and real per capita income (r =-0.1207, p<0.0001) [6]. Biadgilign and colleagues posit that improved per capita income in Ethiopia could enhance expenditure for food and basic health services, leading to improvements in child nutrition [6].

**Key Takeaway**: The overall results of these studies suggest that improved economic development is associated with a reduction in stunting at a national-level in Ethiopia

**Political**

Only one study, a 2019 national trend study by Biadgilign et al, examined the relationship between governance and stunting outcomes. Using indicators drawn from the Worldwide Governance Indicators (WGI), predictors such as government effectiveness, regulatory quality, and control of corruption were measured in standard deviation units. This ranged from -2.5 to 2.5 z-scores, or through percentiles from 0 to 100, where higher scores suggest better outcomes in the country. It was found that government effectiveness (AOR = 20.7; p = 0.046), regulatory quality (AOR = 0.0077; p = 0.026) and control of corruption (AOR = 0.0019; p = 0.000) were all positively associated with a reduction in stunting prevalence among children 6-59 months in Ethiopia [7].

**Key Takeaway**: Though it is only one study, and further evidence is required, the result of this study could reflect Ethiopia’s progress towards stunting reduction through good governance, combined with a society that is able to exercise its rights and participate in the country’s efforts.

**Environmental**

***Stunting and HAZ by Region***

Several studies have examined the impact of regional differences on child height-for-age outcomes. According to a 2015 systematic review by Birhanu et al, among all regions, Amhara, Tigray and Somali were most frequently reported to have the highest prevalence of child malnutrition, including stunting [8]. A 2019 national trends study by Biadgilign et al evaluated the significance of living in a particular region on stunting outcomes. Compared to the region of Tigray, it was found that only the region of Amhara had a 14% increased odds of stunting among children 6-59 months (OR= 1.14, R.SE =0.08, p=0.054). Other regions such as Oromiya (OR= 0.79, R. Sta. err = 0.05, p= 0.000), Somali (OR= 0.592, R. Sta. err = 0.48, p= 0.000), Gambela (OR= 0.519, R. Sta. err = 0.048, p= 0.000), Harari (OR= 0.601, R. Sta. err = 0.054, p= 0.000), Addis Ababa (OR= 0.6710, R. Sta. err = 0.076, p= 0.000), and Dire Dawa (OR= 0.624, R. Sta. err = 0.61, p= 0.000) were all significantly associated with a ~20%-40% reduction in stunting odds. This study also found that living in Afar, Benishangul-Gumuz, and SNNP was not significantly associated with stunting outcomes [7]. Similarly, in an earlier national trends study by Biadgilign et al from 2016, Somali region [β= -0.4384, SE = 0.0979, p = 0.000], Gambela region [β= -0.6451, SE = 0.1007, p = 0.000], Harari region [β= -0.5553, SE = 0.1066, p = 0.000], Addis Ababa [β= -0.4840 , SE = 0.1256, p=0.000], and Dire Dawa [β= -0.5315, SE = 0.1081, p = 0.000] were found to be significantly associated with reduced HAZ compared to Tigray region, while Afar, Amhara, Benishangul-Gumuz, and SNNPR were not [6].

In a 2014 national trend study by Headey et al, regions shown to be positive predictors of HAZ relative to Oromiya included: Somali (b=0.32, p<0.01), Gambela (b=0.47, p<0.01), Harari (b=0.20, p<0.01), and Dire Dawa (b=0.20, p<0.01). Regions negatively associated with HAZ scores relative to Oromiya were: Amhara (b= -0.24, p<0.01), SNNP (b= -0.17, p<0.01), and Tigray  (b= -0.26, p<0.01). Similarly, compared to Oromiya, regions shown to positively predict increases in stunting prevalence included Amhara (b=0.06, p<0.01), SNNP (b=0.03, p<0.05), and Tigray (b=0.07, p<0.01). On the other hand, Somali (b= -0.08, p<0.01), Gambela (b= -0.11, p<0.01), and Dire Dawa (b= -0.05, p<0.05) was significantly associated with an increase in stunting prevalence relative to Oromiya. Finally, regions shown to be a positive predictor of severe stunting prevalence included: Afar (b=0.065, p<0.01), Amhara  (b=0.055, p<0.01), SNNP  (b=0.060, p<0.01), and Tigray  (b=0.036, p<0.01), with Gambela  (b=-0.038, p<0.05) being the only region found to be negatively associated with severe stunting prevalence relative to Oromiya [9]. A further 7 national cross-sectional studies and 1 sub-national cross-sectional study [10] examined the significance of living in a particular region with child growth outcomes, all with differing results [1,11–16]. In particular, one of the national cross-sectional studies from 2016 by Demewoz et al found that the spatial variation of stunting in the northern parts of Ethiopia had statistically significant hot-spots for stunting, including Benishangul-Gumuz, Amhara, Tigray and Afar regions [11].

***Stunting Change by Region***

A 2014 national trends study by Hagos et al found that higher prevalence of stunting can be seen in the highlands and midlands regions of the country, compared to the lowland regions [17]. According to a 2019 national trends study by Hirvonen et al, stunting rates have decreased in all regions of the country, with the exception of Dire Dawa. This study also found that of the four most populated highland regions, stunting reduction had occurred most rapidly in Tigray (-2.9 % per annum) and slowest in Amhara (-1.8 % per annum) [18]. This finding is supported by an earlier national trend study from 2017 by Wirth et al that found that between 2000 and 2005, the 6.9% decrease in stunting nationally could be attributed to reductions in stunting in the regions of Oromiya, SNNPR, Tigray, and to a lesser extent Afar and Addis Ababa. From 2005-2011, the authors find that the national decline in stunting prevalence of 6.8% was mainly driven by Amhara, SNNP, Somali and Oromiya, and to a smaller degree by Addis Ababa, Harari and Gambela [19].

**Key Takeaway**: These studies highlight that regional differences in child stunting outcomes do exist in Ethiopia. Furthermore, similarities can also be observed between groups of regions. For example, Tigray, Amhara, and Afar are all regions located in the northern parts of Ethiopia and are generally associated with increased HAZ scores and stunting prevalence, whereas regions further towards the south such as Oromiya, Somali, and SNNPR have mostly been associated with a decrease in stunting prevalence, and an overall trend towards improved HAZ scores. Future studies on geographical and environmental diversity within each region may provide more insights in to explaining these regional variations.

***Climate and Food Shocks***

Despite Ethiopia’s history of environmental shocks such as recurrent drought and it’s links in the literature to issues such as food security [20], only a small handful of studies included in this review evaluated environmental factors in association with child growth outcomes. Among these, a 2014 national trends study by Hagos et al regarding the impact of climate change on HAZ found a positive association between increasing rainfall during growing season and stunting among Ethiopia’s midland zones (b=0.495, SE=0.16, p<0.05). However, the authors also indicate that extreme forms of rainfall leads to a higher prevalence of moderate stunting based on these models. In addition, a negative association was also found between increasing temperature (^○^C) and a reduction in HAZ within lowland zones (b= -0.55, SE=0.26, p<0.05). Lastly, the same study found that stunting was not associated with the quadratic term for rainfall (r2 = 0.060, se = 0.14) or per capita crop availability (r2 = -0.53, se=0.000), and that severe stunting was not associated with temperature (r2 = -0.24, se = 0.11), the quadratic term for rainfall (r2 = 0.52, se = 0.12), or per capita crop availability (r2 = 0.13, se=0.85) [17].

**Key Takeaway:** Overall, these studies support the notion that climate change through rainfall, drought, and temperature change partially predict the variation in child stunting in Ethiopia. Based on this, it is recommended that small-scale interventions focusing on improving agricultural sustainability and crises prevention could alleviate the impact of food shocks caused by natural disasters.

In terms of crises in food availability, a 2017 national trends study by Wirth et al found that the 2002 and 2011 food crises experienced in Ethiopia were not associated with an increase in stunting prevalence in any of the three regions most affected by these crises (i.e. Afar, Oromiya, and Somali). This study further noted that from 2000 to 2005, the prevalence of stunting decreased in these regions, although the number of cases in the Somali region increased [19]. Related to overall food availability, a national trends study by Headey et al also noted that from 2000-2011, Ethiopia saw a rapid increase in agricultural productivity.

Along the same vein as food crises, a 2019 national cross-sectional study by Tesfay et al used mixed-effect regression to examine the association between climatic regions and nutrition outcomes (HAZ) with and without the influence of shocks to food availability. Without shocks, tropic-warm/semiarid (r= -0.920, SE=0.55, p<0.1) and tropic-cool/humid climates (r= -0.977, SE=0.57, p<0.1) were negatively correlated with child HAZ scores. With shocks, only tropic-cool/humid climates (r= -1.013, SE=0.57, p<0.1) were negatively correlated with HAZ. The authors attribute this area variation to be part of the regional variation on nutrition outcomes that exists in Ethiopia. Compared to the Amhara region, a boy being in Oromiya has better nutrition than a boy in Amhara. Tesfay and colleagues conclude that a child who grows up in the northern part of Ethiopia such as Tigray – a region commonly associated with war and drought-prone areas – is less nourished than children who grow up in other regions. This reflects the negative correlations seen between more arid or humid climates and child HAZ scores.

Lastly, a 2015 sub-national cross-sectional study by Motbainor et al examined the impact of agro-ecology on HAZ. Agro-ecological zones were based on the four major cereal systems (sorghum-maize/lowland; wheat-teff/single rain season mid-land; wheat-teff/double rain season mid-land; barley/highland) in the Amhara region. The study found that compared to areas without an agricultural system in place, agro-ecological systems were not significantly associated with under-five HAZ (b=0.010, SE: 0.039 , 95% CI: - 0.065, 0.086) [21]. The authors’ takeaway is that this non-significant relationship has less to do with climate and food shocks, and more to do with the fact that it is common practice in the Amhara region to sell cereal and agricultural products rather than feeding these nutritious foods to children.

**Key Takeaway**: The limited evidence of the impact of food shocks on stunting outcomes generally shows a lack of association, however further research is required. In particular, it could be useful to understand how the impacts of climate change are shaping the resiliency preparedness of those in areas prone to food shocks, and among staple crop-producing communities.

**Socioeconomic Factors**

**Wealth Index**

Three national trend studies found household wealth, measured using asset indices, to be positively associated with under-5 HAZ [9,22,23]. The first used DHS data from 2000, 2005 and 2011 and found that at the national level, among children under 6 months, between 6-23 months and between 24-59 months, as household wealth increases, HAZ also increases (b=0.17, p<0.001, b=0.28, p<0.001, b=0.20, p<0.001, respectively). The second used the same rounds of DHS data and in addition to the positive association between household wealth and asset index (b=0.081, SE=0.01, p<0.01), also found asset index to account for 7.3% out of the 22% predicted change in under-5 HAZ from 2000 to 2011 [23]. The third study used the 2000 and 2011 DHS rounds. It found for children under-5, asset index was positively associated with HAZ (b=0.10, p<0.01) and negatively associated with stunting (b=-0.03, p<0.01) and severe stunting (b=-0.025, p<0.01) [9]. An increase in asset index was associated with an increase in HAZ and a decrease in the risk of stunting and severe stunting. Lastly, their decomposition analysis for change in stunting from 2000 to 2011 found that 9.3% of the explained change in stunting prevalence was attributed to asset index out of the overall explained change of 15.3% [9].

Three additional national trend studies analyzed the association between wealth and stunting [18,24,25]. The first used 2005 and 2011 DHS data and found that children 0-23 months in the top 60% of the wealth distribution were slightly less likely to be stunted compared to children in the bottom 40% (β= -0.039, SE= 0.013, p<0.01) [24]. The second study examined inequalities with regards to the prevalence of under-5 stunting between 2000 and 2014. The authors found that the prevalence of stunting differs between wealth quintiles (top 60% and bottom 40%) [25]. When comparing wealth quintiles, the largest difference was between the wealthiest quintile as compared to the others (poorest, poor, middle, richer) with the difference increasing over the time period of 2000-2014 [26]. A decomposition of wealth inequalities in stunting showed that only the wealth index contributed to inequalities from 2000-2014 [26]. Lastly a study using 2000-2016 DHS data found wealth to be strongly correlated with the height of children under-5 and the reduction of stunting prevalence from 2000 to 2016 was fastest in the top wealth quintiles, averaging more than 3% per year in contrast to less than 2% at the lowest quintiles [18]. In 2016, 46% of children under-5 residing in the poorest households were categorized as stunted compared to 26% in the richest households [18].

A national cross-sectional study using pooled DHS data from 2000-2011 found that children under-5 in the richest wealth index quintile [β= -0.2693, SE = 0.0745, p<0.001] had a lower risk of stunting as compared to those belonging in the poorest wealth index quintile. However, there were no significant differences among those belonging in all other wealth quintiles as opposed to the poorest wealth quintile (richer β=0.0867, SE= 0.0514, p= 0.092, middle β =-0.0250, SE = 0.0485, p= 0.607, poorer β =-0.0277, SE = 0.0471, p = 0.556) [6]. A second cross-sectional national study by Biadgilign using pooled DHS data from 2000-2016 found that children aged 6-59 months in the middle, richer and richest wealth quintiles had a reduced risk of stunting compared to children in the poorest quintile (middle OR= 0.904, R. Sta. err= 0.040, p=0.023, richer OR= 0.842, R. Sta. err= 0.037, p= 0.000, richest OR= 0.746, R. Sta err= 0.041, p= 0.000). There was no significant difference between those belonging to the poorer quintile compared to the poorest quintile (OR = 0.952, R. Sta. err=0.042, p=0.267) [7].

Nearly all national cross-sectional studies were in agreement regarding the negative impact of lower household wealth on chronic child malnutrition. Increased household wealth was found to be negatively associated with stunting from national cross-sectional studies using various rounds of the DHS (e.g. 2000, 2011, 2014, 2016) [1,11,12,14–16,27–30]. However, three national cross-studies that used data from the DHS 2005 and an additional one that used data from the Ethiopian National Micronutrient Survey and Africa Soil Information Service Soil Map found no association between household wealth and HAZ in under-5 children [31–34]. Furthermore a regional longitudinal study conducted in Butajira in the Gurage Zone of SNNPR followed a cohort of children between 0-12 months over a year and did not find any association between the poverty index and HAZ [35].

A national cross-sectional study using 2011 DHS data found the odds of stunting generally decreased as wealth increased among children under-5 (poorest AOR = 1.70 (1.38, 2.10), P<0.001, poor AOR = 1.89 (1.51, 2.35), P<0.001, middle AOR = 1.59 (1.28, 1.99), P<0.001, richer AOR = 1.45 (1.17, 1.80), P<0.001 as compared to richest) [36]. Another study using 2011 DHS data found a similar association but it was only significant for children 25-59 months (poorest: OR=1.70, 95% CI: 1.22-2.38, poorer: OR= OR=1.70, 95% CI: 1.22-2.38, middle: OR=1.57, 95% CI: 1.12-2.20, richer: OR=1.56, 95% CI: 1.11-2.17 as compared to richest) [37]. Six national cross-sectional studies were in agreement that households belonging to the poorest wealth quintile were the ones at the greatest risk of stunting [11–14,16,38]. Specifically, one study using the 2004 Welfare Monitoring Survey (WMS) compared households with medium socioeconomic status with those of low socio-economic status (n=8827) (Gurmu 2013) [13]. They found that the odds of stunting among children from low socio-economic status increased by at least 42% (β=1.42, p<0.001). Children who belonged to households of high socioeconomic status had a 28% reduced risk of stunting (β=0.72, p<0.001) [13]. Lastly, a study using 2016 DHS data decomposed the factors associated with the 18.2% difference in the prevalence of stunting between the richest and poorest socioeconomic groups [38]. They found that 61.1% of the inequality was due to the social determinants of stunting, largely caregivers’ education status (33.3%), followed by region (state) of residence (11.1%), and birth size (5.6%) [38]. Poor socioeconomic status and overall family income in Ethiopia were also identified as main risk factors for under-5 stunting in two systematic reviews, however the direction and strength of these associations were not stated [8,39]. Lastly, in one sub-national cross-sectional study conducted among both highland and low-land people in Ofla district, southern Tigray, annual household income was found to be significantly tied to stunting outcomes among those in the highland (AOR 2.052, CI:1.31-3.22, p<0.01), but was insignificant for those living in the lowland [40].

*Assets*

One sub-national cross-sectional study used the types of roofing on a house and household asset ownership (i.e. cows, barns, and umbrellas) as markers for socioeconomic status. It found that stunted children between the ages of 6-23 months living in rural communities in SNNPR less often had roofs of corrugated iron (3 vs. 14, p=0.01) and fewer lived in a household that possessed a separate building for their animals or for their kitchen (3 vs. 15, p=0.006) [41]. In this study, roofs of corrugated iron and possession of key assets were associated with higher socioeconomic status, thus the results of the study demonstrate lower socio-economic status is associated with stunting in rural communities in SNNPR. In support of this finding, a systematic review identified among its included studies that household ownerships of cows in rural areas to be associated with a reduction in stunting prevalence [42]. However, a national cross-sectional study pooling 2005 and 2011 DHS data found having livestock was not associated with risk of stunting (β= 0.001, SE= 0.022; Urban β= -0.008, SE= 0.027) [24], nor did a sub-national cross-sectional study conducted in northern Tigray [43].

**Key takeaway**: With some slight variation in the vast amount of research that has been done on the association between wealth and/or assets and child growth outcomes, it can be said that in general, increases in household wealth and assets are associated with increases in under-5 HAZ and decreased probabilities of under-5 stunting in Ethiopia.

*Parental Occupation*

Two studies looked at indicators such as parental occupation and housing structure that are directly related to household wealth. Firstly, a national cross-sectional study using pooled DHS data from 2000, 2005, and 2011 examined the association between parental occupation and under-5 stunting. They found no association between the risk of stunting and engaging in paid work or the provision of an agricultural service as compared to not working (Respondent: working paid: β = 0.0384, SE= 0.1381, p= 0.781 or agricultural service: β = 0.0172 SE = 0.1366, p=0.900 and Partner: working paid: β = 0.0737, SE = 0.0424, p= 0.082 or agricultural service: β = 0.0667, SE = 0.0420, p = 0.112) [6]. One additional national cross-sectional study using pooled DHS data from 2000-2016 also found that type of parental occupation was not associated with stunting (Respondent: paid working OR= 1.055, R. Sta. err=0.042, p=0.175 or Agricultural OR= 1.043, R. Sta. err= 0.041, p= 0.279 and Partner: paid working OR=1.102, R. Sta. err= 0.084, p= 0.201 and agricultural OR= 1.131, R. Sta. err= 0.080, p=0.082)) [6].

**Key takeaway**: Based on the limited number of studies included in this review that examined the relationship between parental occupation and stunting outcomes in children under-5, no association was found. However, further evidence is required in order to support this conclusion.

**Women’s Empowerment**

A number of studies examined factors linked to female empowerment as predictors of child stunting. Firstly, a sub-national study conducted in Butajira in the Gurage Zone of SNNPR followed a cohort of children between 0-12 months over a year and did not find any association between being in a polygamous marriage, female autonomy, social support, or khat and/or alcohol use of the mother with stunting among children at 6 months or age and 12 months of age [35]. The age of the household head was not associated with moderate stunting in a study using 2011 DHS data, but was associated with severe stunting [28]. However, the sex of the household head (male) AOR = 1.18 & CI: (1.01 – 1.38) was associated with stunting in the 2011 DHS [11], while a sub-national cross-sectional study conducted in the North Wollo Zone of Amhara region found a female-headed household was positively associated with stunting [ AOR 2.17 (1.05, 4.50), p=0.009]. In another study examining data from the 2005 DHS, the age of a female household head increased HAZ outcomes [31]. Notably, two national cross-sectional studies found no improvement in child HAZ or reduction in stunting risk with female household heads [27,44]. Employment status of the mother (Unemployed: OR = 0.95, 95% CI: 0.84, 1.08, Employed not for cash: OR = 1.01, 95% CI: 0.88, 1.15) and the sex of the household head (OR = 1.11, 95% CI: 0.97, 1.28) was also not associated with child HAZ using data from the 2000 DHS. The authors hypothesized that unemployment among mothers may not increase the risk of malnutrition as the time that is allocated towards earning income may be at the expense of time spent in feeding and caring for children [16]. There is further support for this finding in a study using data from the 2005 DHS, where the authors found malnutrition was higher in those children whose mothers were working at the time of interview or twelve months before the interview period [32]. An additional national cross-sectional study found that children of working mothers were more likely to be stunted [29], while a sub-national cross-sectional study from Amhara region found no association between maternal occupation and stunting [45]. The study by Tesfaye [32] also found that children living with both parents were less stunted than other children. However, another study found that household structure (two parents) and sex of household were not associated with stunting using the 2004 Welfare Monitoring Survey (WMS) [13].

**Key Takeaway**: The impact of women’s empowerment on child growth outcomes continues to be difficult to quantify, as proxy variables are often an imperfect representation of the occurrence of empowerment. Of the studies included in this review, those that examined the impact of female headed households and female autonomy as proxies of empowerment found that there was a no association between these variables and stunting or HAZ. Of the handful of studies that examined the impact of female employment on stunting outcomes, working mothers had children more likely to be stunted than non-working mothers.

**Parental Education**

Maternal education has been found to reduce the risk of chronic malnutrition in a number of national studies in Ethiopia. A national trend study using DHS surveys between 2000-2011 found that maternal education was positively associated with HAZ and negatively associated with stunting (b=0.02, p<0.05, b=-0.01, p<0.01) but was not associated with severe stunting (b=-0.003) among under-5 children [9]. Paternal education was also positively associated with HAZ and negatively associated with both stunting and severe stunting among under-5 children (b=0.02, p<0.01, b=-0.01, p<0.01, b=-0.003, p<0.05) and accounted for 3.3% of the overall explained change in the prevalence of stunting, which was 15.3% [9]. A second study by the same authors found that maternal and paternal education (b=0.02, SE: 0.006, p<0.01, b=0.02, SE: 0.005, p<0.01, respectively) were positively associated with child HAZ [23]. In the 1998 National Nutrition Survey, the prevalence of stunting was more than double among children under-5 born to mothers with no formal education compared to those whose mothers completed grades 9-12 [20]. A national trend study using 2000-2016 DHS data found partner’s and respondent’s education at all levels to be associated with a reduced risk of stunting (partner's education level: primary OR= 0.924, R. Sta. err = 0.031, p= 0.020, secondary OR= 0.711, R. Sta. err = 0.045, p= 0.000), higher OR= 0.622, R. Sta. err = 0.567, p= 0.000 and highest educational level of the respondents: primary OR= 0.918, R. Sta. err= 0.035, p=0.027, Secondary OR= 0.595, R. Sta. err= 0.050, p= 0.000, Higher OR= 0.447, R. Sta. err= 0.077, p= 0.000) [7]. A study that aimed to examine risk factors of under-5 stunting using the 2000-2011 DHS and the WHO conceptual framework to guide analyses also found that higher levels of maternal education increased child HAZ among children between 24-59 months (No education: b=-0.406 and primary: b=-0.345 as opposed to secondary, p=0.001) [22]. Moreover, they examined factors that may be related to female empowerment and found mothers not using contraception and their partner’s occupation (none: b=0.68, agriculture: 0.10 as compared to unskilled service/sales/prof (ref), p=0.015) among children 6-23 months (b=-0.02, p=0.014) and older age of the head of the household among children 6-23 months and 24-50 months (b=0.23, p=0.005, b=0.01, p<0.001, respectively) decreased child HAZ [22]. Lastly, a national cross-sectional study using 2005 DHS data found no association between maternal education and HAZ outcomes [34], while another national cross-sectional study using 2011 DHS data found that the odds of stunting were not associated with maternal education among children 12-24 months or older than 24 months (none: OR= 4.41, 95% CI: 0.78-24.87 and OR=3.42, 95% CI: 0.90-12.98), primary: 4.01, 95% CI: 0.72-22.48 and OR=3.66, 95% CI: 0.96-13.88, secondary: OR=1.41, 95% CI: 0.21-9.56 and OR=1.88, 95% CI: 0.37-9.59 as compared to higher level of education) [37]. This was further supported by two sub-national cross-sectional studies that found maternal education was not associated with stunting [43,45]. Like mother’s education, studies have also shown the importance of paternal education. A national study using pooled DHS data (2000, 2005, 2011) found that higher levels of education of the respondent’s (namely mothers) partners was associated with a decreased risk of stunting (primary: β = -0.0589, SE = 0.0374, p = 0.115, secondary: β= -0.4333, SE = 0.0642, p < 0.001, higher than secondary: β= -0.7743, SE = 0.1147, p = 0.000 as compared to no education) [6].

A number of cross-sectional national studies had consistent findings regarding the positive effects of increasing levels of parental education with child HAZ [31,44], with only one study by Tesfaye et al finding no association, though they did find that an educated partner (at least primary education) contributed to better child nourishment [32]. One study using the 2000 DHS found that maternal education had a minimal impact of merely 1.4% on reducing the risk of child malnourishment. The authors hypothesized that this is a reflection of the low levels of female education in Ethiopia, particularly in rural areas [27]. This study also found that in the DHS 2000, father’s education did not reduce the risk of stunting [27]. Five studies found that maternal and paternal education at the secondary level or higher reduced the risk of stunting [11–13,16,46]. A study by Gebru found only maternal education at these levels to reduce the risk of stunting [1]. Moreover, one study found that children of non-educated mothers were nearly 3 times (OR=2.78, 95% CI: 1.66, 4.64) more likely to be moderately stunted and 5 times (OR= 4.89, 95% CI: 2.27,10.53) more likely to be severely stunted as compared to children whose mothers attended secondary or a higher level of education [28]. One cross-sectional study found parental education to not be associated with stunting [24]. A study by Gebru found paternal education to not be associated with stunting and a study by Mohammed et. al found maternal education to not be associated [1,38]. A cross-sectional study using 2016 DHS data found children of mothers with primary level or no level of education to be at increased risk for stunting [14]. Two cross-sectional studies found a child’s risk of stunting to decrease with increasing levels of mother’s education [30,36].

**Key Takeaway**: Of the literature included in this review that examined the association between parental education and child growth outcomes, a vast majority indicated that in Ethiopia, parental education, and especially maternal education, is positively associated with HAZ and negatively associated with stunting for children under-5 years.

**Nutrition-specific and Sensitive Programs**

**Community-based Nutrition Program**

The Community-based Nutrition Program (CBNP) began in 2008 in Amhara, Oromiya, SNNPR, and Tigray regions, and focuses on improving the nutritional status of women and children under-two years through cost effective, community-based interventions. According to a 2017 randomized control trial by Kang et al which tested the effectiveness of the Community-based Nutrition Program among 6-12 month olds in 2 districts of the Oromiya region, those in the intervention areas had a greater increase in length-for-age (LAZ) scores [difference (diff): 0.021 z score/month, 95% CI: 0.008, 0.034] compared to children aged 6-24 months in the control areas. Furthermore, after 12 months, those in the intervention areas also showed an 8.1% (*P* = 0.02) lower stunting prevalence compared to those in the control areas [47]. A 2012 impact assessment of the CBNP among children under-3 in the regions of Amhara, Oromiya, Tigray and SNNPR by White et al also found significant improvements in HAZ scores (HAZ of 0.26 z-score units: -1.731 to 1.473; p<0.05) between baseline and midline surveys, as well as significant differences in stunting prevalence rates from 50.5% down to 40.6% (9.9%; p<0.001). The authors concluded that the CBN program led to a substantial decrease in stunting among children under-3 years in the intervention areas, which was likely attributed in some ways to a high ratio of Volunteer Community Health Workers (VCHWs) in the study area; regular contact between mothers and VCHWs as well as Health Extension Workers (HEWs); effective counselling given to mothers on appropriate feeding and caring practices; and finally referral of sick children to health facilities and supplementary feeding where necessary [48].

**Productive Safety Net Program**

Several studies have conducted evaluations of the Productive Safety Net Program (PSNP), which is a social protection initiative that provides income supplements to participating families. An assessment of PSNP by Berhane et al in 2014 found that diet quality remained poor among PSNP participants, and that there was no evidence the children of those households receiving the PSNP income supplement have improvements in the consumption of foods such as pulses, oils, fruit, vegetables, dairy or animal source proteins. There was also no evidence shown that PSNP participation led to improvements in HAZ or stunting outcomes, even when disaggregated by survey round, age and sex [49]. Using Ethiopia data from the Young Lives study, Porter et al also conducted an impact evaluation of PSNP in 2016. Results of this evaluation indicated that siblings within a household receiving PSNP support showed an improvement in HAZ at age 5 when compared to index children from 3 years prior, before PSNP was rolled out. However, the difference in mean HAZ between those who had received PSNP and those who had not within the same year (post-treatment) were not found to be significantly different [50]. Lastly, an evaluation of the impact of PSNP on children 6-24 months by Gebrehiw et al in 2018 found that PSNP showed no impact on HAZ, though the authors add the caveat that the two years allotted for measurement of effect may not be long enough to show significant improvements in stunting outcomes [51].

**Targeted Supplementary Food Program**

The Targeted Supplementary Food (TSF) program is a supplementary feeding program for under-5 children and pregnant or lactating women who screen positive for acute malnutrition in selected regions in Ethiopia [52]. A 2009 outcome evaluation of the TFS program was conducted by Skau et al at the World Food Programme in eight rural districts of four regions (Amhara, Afar, Tigrey and Somali) from 2008-2009. The control and intervention groups included acutely malnourished children 6-59 months of age who were not critically ill. The study found that the mean HAZ steadily declined for both intervention and control groups in four follow ups after program implementation had concluded (1st follow up p=0.001, 2nd follow up p=0.001, 3rd follow up p<0.001 and 4th follow up p<0.001), and that the rate of growth in the intervention group lagged behind that of the control group. Though these results may support the lack of effect of TSF on improving HAZ outcomes, the authors caveat that change in HAZ was dependent on nutritional status at baseline in this study, and that the intervention group had a higher prevalence of severe acute malnutrition than the control group, which may account for some differences in HAZ changes at end line [52]. Given these more nuanced findings, further research is required to understand the impact of the TSF program in the for regions where it was implemented.

**Underlying Causes**

**Care and Health Services**

**Immunization**

Immunization is an important part of maintaining overall child health and lowering the risk of illness. When children experience repeated bouts of illness, their bodies can become depleted of essential nutrients, which may in turn impact growth outcomes. In terms of the impact of immunization on HAZ and stunting outcomes in Ethiopia, existing literature is somewhat mixed. A 2018 systematic review of chronic malnutrition among children under-5 in the country by Abate et al found that four studies reported optimal health practice (including immunization) by mothers or their children to be associated with reduced stunting outcomes [53]. Another national cross-sectional study by Megabiaw et al in 2013 found that children who had not received measles vaccinations had a 1.6 times [CI = 1.43, 1.75), p=0.000] and 1.4 times [CI = (1.26,1.5), p=0.000] higher risk of becoming moderately and severely stunted, respectively [28]. Two additional sub-national cross-sectional studies conducted in Ethiopia between 2007-2017 further support the association between receiving immunizations and reductions in stunting, while an additional three studies found evidence of increased stunting odds among under-5 children who had not been immunized [54–58]. Conversely, a national trends study by Suri et al in 2014 found through bivariate analysis of 2000, 2005 and 2011 DHS data that for Ethiopia children under-5, DPT vaccines were not correlated with stunting or HAZ [37]. Along with this, a national cross-sectional study by Demewoz et al in 2016 found that incomplete immunizations was also not significantly associated with stunting in children under-5 (AOR =1.01 & CI: (0.76–1.34) [11]. An additional 4 sub-national cross-sectional studies conducted between 1999-2016 also found childhood vaccinations to have no significant impact on stunting outcomes [59–62].

**Key Takeaway:** Overall, the majority of studies in this review that examined the association between immunization and stunting provide supporting evidence to suggest a protective effect of childhood immunization on stunting outcomes, though more research may be required to understand the nuances and pathways of this relationship.

**Skilled Birth Attendance**

A skilled birth attendant such as a doctor, nurse, midwife or community health worker facilitating in the delivery of a newborn is important to ensure both the safety of the mother and the child, and to engage both with the health care system should there be any complications and/or for post-natal follow up care. According to a national trends study conducted in 2019 by Buisman et al, delivery by a skilled birth attendant was negatively associated with stunting outcomes for children <24 months in Ethiopia (β= -0.079, SE= 0.025, p<0.01) [24]. Similarly, in a national trends study by Headey et al in 2017 using DHS data from 2000, 2005, and 2011, it was found that being born in a medical facility was positively associated with HAZ (b=0.18, SE: 0.05, p<0.01) [23]. The importance of skilled delivery attendance in reducing moderate stunting outcomes was further supported by 2 additional sub-national cross-sectional studies [5,63]. In contrast, another national trends study by Headey et al in 2014 found that medical delivery was not significantly associated with HAZ (b=0.07), stunting (b=-0.01) or severe stunting (b=-0.021) among Ethiopian children under-5 [9]. This lack of association with stunting and/or severe stunting was further supported by two national cross-sectional studies [1,14], and 4 sub-national cross-sectional studies [5,40,45,62].

**Key Takeaway**: Overall, at a national-level, skilled birth attendance is shown to have a negative association with stunting, particularly among more recent studies. Some variation in this finding does exist at the sub-national level, and could be an area for future study.

**Antenatal Care**

In total, 24 studies explored the potential significance of women receiving antenatal care (ANC) for stunting outcomes in Ethiopia. A national trends study by Buisman et al in 2019 found that among mothers of children 6-23 months, having 4 or more ANC visits was significantly associated with a reduction in stunting outcomes compared to mothers with ≤ 3 ANC visits (β= -0.056, SE = 0.019, p<0.01) [24]. Similarly, in a 2017 national trends study by Headey et al, 4+ ANC visits was positively associated with HAZ (b=0.16, SE: 0.04) [23]. This supports an earlier national trends study by Headey et al from 2014, where 4+ ANC visits was significantly associated with increases in HAZ (b=0.22, p<0.01), reduction in stunting outcomes (b=-0.07, p<0.01) and declines in severe stunting outcomes (b=-0.044, p<0.01). All of this contributed to 2.7% of the change seen in stunting from 2000-2011, based on decomposition analysis [9]. Along with these studies, a 2014 national trend study by Suri et al found that fewer antenatal care visits was a risk factor for stunting increase [37]. A 2018 systematic review by Abate et al also reported that optimal care for mothers and their children, including antenatal care, was shown to be significant factor for stunting outcomes in four of their included studies [53]. In addition to these aforementioned studies, 2 national cross-sectional [16,29] and 5 sub-national cross-sectional studies [56,64–67] examining factors associated with stunting among children 0-59 months came to similar conclusions on the significance of increased ANC visits on HAZ and stunting (HAZ < -2 SD) outcome reductions.

In contrast to these findings, the aforementioned national trends study by Buisman et al found that for children <24 months, mothers having 1-3 ANC visits was not significantly associated with stunting in Ethiopia (β= 0.007, SE= 0.015), potentially pointing to the importance of receiving the recommended four visits, as this was found to be significant in the same study [24]. A 2017 national trends study by Woodruff et al also found no significant association between HAZ and ANC for children <6 months [22], nor did 3 national cross-sectional studies [1,15,34]. Lastly, 5 sub-national cross-sectional studies found that 0-3 maternal ANC visits was not associated with stunting among children 6-59 months across Somali and Southern zones in Ethiopia (59, 61, 68, 74, 101). Similarly, 2 sub-national cross-sectional studies looking at the same age group around the Tigray region failed to find a significant association between the number of ANC visits and stunting reduction (63, 88).

**Key Takeaway**: Overall, the majority of these studies demonstrate that ANC, and particularly ANC 4+, have a positive impact on reducing stunting outcomes among children under-5 in Ethiopia. This impact may be attributed in some way to the importance ANC visits can have for providing pregnant women with information on maternal and infant nutrition and early prevention and treatment of infections, among other topics.

**Postnatal Care**

Only four of the studies included in this literature review evaluated the significance of post-natal care for stunting outcomes. Of these, a 2012 national cross-sectional study by Rajkumar et al found that for children under-5, a post-natal check-up was not significantly associated with stunting for all children b = –0.14; t-ratio = –0.68, girls b = –0.14, t-ratio = –0.54; and boys b = –0.14 t-ratio = –0.45, nor was a checkup in the first two months after birth for all children (b = 0.14, t-ratio = 0.64); girls (b = –0.07, t-ratio = –0.24); or boys (b= 0.20; t-ratio = 0.57) [31]. Lack of post-natal care was also found to be insignificant for stunting outcomes in a 2016 cross sectional study of agro-pastoralists in northeast Ethiopia by Liben et al (AOR= 1.26 (0.63, 2.49) [65]. However, lack of post-natal care was found to be significantly associated with a 60%-65% increase in stunting odds between 2 cross-sectional studies – one conducted in the Somali region by Shine et al in 2017 [AOR= 1.59 (1.07, 2.37) p<0.05] [62], and one conducted in Kemba in 2015 by Eskezyiaw et al [AOR: 1.64; 95% CI (1.05–2.55), p=0.029] [68].

**Key Takeaway**: Conflicting evidence across a small number of cross-sectional studies suggest further evaluation on the impact of postnatal care on stunting risk.

**Vitamin A Supplementation**

The association between stunting or HAZ and Vitamin A supplementation in Ethiopia was examined by 8 studies included in this literature review. Among these, a 2019 national cross-sectional study by Mohammed et al using DHS data from 2016 found that among children 6-23 months, receiving a vitamin A supplement in the last 6 months was significantly associated with stunting reduction through increasing HAZ scores [Adjusted β = 0.16 (0.03, 0.29), p = 0.020)] [15]. Along with this, three sub-national cross-sectional studies published between 2014-2017 found that not receiving vitamin A supplementation was associated with increased stunting among children 6-59 months [69–71]. One of these studies conducted in the Hawassa Zuria woreda zone of southern Ethiopia found an ~8 times increase in stunting odds among children 6-59 months who did not receive vitamin A supplementation (COR= 7.93 (4.00-15.77), p<0.05) [69]. Only one subnational study assessing the impact of community-based nutrition programs in South East Amhara found a significant association with vitamin A supplementation and increased stunting odds [AOR = 1.774 at 95% CI (1.066, 2.951)] [56].

In contrast, a national cross-sectional study conducted by Mohammed et al in 2017 found using DHS data from 2011 that for children 6-24 months, receiving vitamin A in the past 6 months was not significantly associated with stunting [β=0·05 (−0·08, 0·17), p= 0·493)] [72]. Along with this, a 2008 national cross-sectional study by Semba et al found that among 12-59 month olds, there was no significant difference in stunting outcomes between those who did or did not receive vitamin A capsules in the previous 6 months [73]. Similar findings were reported by Getaneh et al in a 2016 sub-national cross-sectional study conducted in Hawassa Town, SNNPR [AOR= 2.558 (0.923, 7.090)] [74].

**Key Takeaway**: The effect of vitamin A supplementation on stunting risk remains promising considering the large stunting odds found among those who did not receive the supplementation in many of the aforementioned studies. That being said, given the variation in findings, future assessments on additional subnational regions and zones could provide a more conclusive picture on whether vitamin A supplementation is beneficial to reducing stunting risk.

**Health Care Access & Utilization**

A handful of the studies included in this review examined indicators related to health care access and utilization, and their impacts on HAZ and stunting. For example, in a 2016 sub-national cross-sectional study by Headey and Hvonen among children under-5 in Amhara, Oromia, Somali, SNNPR and Tigray regions, having a health worker visit was not found to be significantly associated with HAZ (b=0.080, SE=0.078) [75]. Similarly, a 2016 sub-national cross-sectional study by Getaneh et al in Hawassa Town, SNNPR found that health and care support were not significantly associated with stunting among orphans and vulnerable children aged 6-59 months [AOR= 1.00 (0.91 ,1.10)] [74]. Poor health access was also not significantly associated with stunting among children 6-59 months in a 2017 sub-national cross-sectional study done by Amare et al in Amhara region [AOR = 1.38 (0.99, 1.91)] [71], nor was distance to a health facility in another national cross-sectional study conducted by Christiaensen et al in 2001 (<=5km) (spline) r2 = -0.001, t = 0.08; (>5km) (spline) r2 = 0.003, t = 0.30 [44]. In terms of utilization of health services, a 2016 national cross-sectional study by Sohnesen et al using data from the 2014 Mini DHS found that health care utilization was not significantly associated with stunting (least square coefficient = -0.02, SE= 0.09) [46], nor was seeking health care for children in a sub-national cross-sectional study conducted by Jemal et al in Gambella Town in 2016 (after 24 h AOR = 0.94 (0.30, 2.96) [76].

Only three studies found significant associations between health care and stunting and/or HAZ in Ethiopia. Specifically, a 2017 national trends study by Headey et al found that health care accounted for 3.3% of the changed in HAZ between 2000-2011 [23]. A 2017 systematic review of from 2000-2015 by Wirth et al found that access to health care is the only factor associated with stunting, where stunted children on average live further away from health clinics compared to their non-stunted counterparts [42]. Supporting this is a 2013 sub-national cross-sectional study in the Afar region by Fentaw et al who found that both distance to a health centre (t= -5.366, P=0.000) and distance to a health institution (t = -5.366, p=0.000) are negatively associated with HAZ [64].

Key Takeaway: Among these studies, the majority found a non-significant relationship between healthcare access and stunting outcomes. However, these measurements are often assessed through units of time or distance (in km) to a health facility, and often did not provide great detail on the health services provided or sought. More research focused on the quality and delivery of health services could serve to better represent the association between health care utilization and stunting risk.

**Household Environment**

**Urban versus rural residence**

A total of 33 studies included in this review looked at the relationship between rural versus urban locality and changes in HAZ or stunting outcomes in Ethiopia. As an indicator of the large number of studies that examine the impact of place of residence on child growth outcomes, a 2018 systematic review of stunting risk factors by Abate et al found that urban versus rural residence was the most commonly reported socio-demographic variable among their included studies [53]. According to a 2019 national trends study by Hirvonen et al, in 2016, stunting was more prevalent in rural areas in Ethiopia compared to urban areas, at 39.9% versus 26%, respectively. People in urban areas have also seen a steeper reduction in stunting prevalence compared to the populations of rural areas, at 3.6% per year versus 2.4% per year, respectively, between 2000-2016 [18]. A 2016 national trends study conducted by Biadgilign et al also found rural residence was significantly associated with stunting among children 0-59 months [β= 0.2769, SE = 0.0849, p = 0.001] [6], as did a national trends study conducted in 2001 by Getahun et al [20]. Living rurally was also seen to be significantly associated with increased stunting in 6 national cross-sectional studies [1,13,28,29,32,36], 1 sub-national trends study [35]; and 6 sub-national cross-sectional studies [3,21,77–80]. Related to urbanization, a 2015 sub-national cross-sectional study by Motbainor et al found that when a child became an urban resident, their HAZ score increased by an average of 0.162 units compared to their rural counterparts (b=0.162, SE: 0.068, 95% CI: 0.029, .294, p<0.01) [21]. Only 1 national cross-sectional study, conducted in 2016 by Tadesse et al, found children in rural areas were 12% less likely to be stunted compared to their urban counterparts (p=0.02) [12].

Conversely, a 2014 national trends study by Headey et al found that urban living was not significantly associated with HAZ (b=-0.06, p<0.10), stunting (b=0.00) or severe stunting (b=0.021) [9], which was also supported by a 2019 national trends study by Buisman et al [β= -0.008, SE= 0.027] [24]. A total of 8 national cross-sectional [11,14–16,29,34,44,81] and 1 sub-national cross-sectional studies [68,82] did not find a significant association between place of residence (urban versus rural) and stunting or HAZ. Lastly, a 2019 national trends study by Biadgilign et al did not find a significant association between urbanization and child undernutrition in the country [7].

**Key Takeaway**: Overall, there is substantial evidence in the literature to support the association that under-5 children living in rural areas are more likely to be stunted compared to under-5 children living in urban areas. However, slight differences in defining which areas constitute as urban or rural may change the magnitude of the associations found across these studies. Nevertheless, the differential effect on stunting reduction seen between urban and rural areas may serve as a proxy for the impact of more proximal and immediate factors such as healthcare access, quality of treatment, and community-level disparities among urban and rural zones.

**Access to improved water sources**

A total of 44 studies examined the relationship between access to improved water and HAZ or stunting in Ethiopia. Included in these is a 2019 national trends study by Biadgilign et al that found an improved source of drinking water to be significantly associated with stunting based on DHS data from 2000-2016 [improved source of drinking water (OR= 0.905, R. Sta. err= 0.042, p= 0.031)] [7]. Similarly, a 2018 systematic review by Abate et al found five included studies reporting the state of water, sanitation and hygiene as a predictor of chronic malnutrition for Ethiopia [53]. In total, 11 sub-national cross-sectional [54,56,87,59,62,76,77,83–86] and 4 national cross-sectional studies [27,29,32,44] which examined the association of stunting among children 6-59 months found that safe and protected (i.e. piped) water sources are significantly associated with reduced stunting odds and overall improved child growth outcomes. In addition to this, one sub-national cross-sectional study conducted in the Ofla district of Tigray found that lack of water treatment increased the odds of stunting by 2.77 (CI: 1.63-4.71), p<0.001) for lowland populations in the area, but was insignificant for stunting outcomes for the highland populations [40]. Several other studies were unable to find an association between water source and stunting. Among these is a 2019 study by Buisman et al that found that for children <24 months, no significant associations existed between drinking surface water and increased stunting outcomes [β= -0.004, SE= 0.015] [24]. In terms of HAZ, a 2017 study by Headey et al found piped drinking water was not significantly associated with HAZ [b=-0.006, SE: 0.05] [23]. These results support a previous study by Headey et al which found piped water to be insignificant for HAZ [b=0.09, p<0.10], stunting [b=-0.03, p<0.10], or severe stunting [b=-0.014] [9]. Lastly, a 2016 study by Biadgilign et al that found improved drinking water was not significantly associated with stunting [6]. Along with this, 9 national cross-sectional [1,11,14,16,27,29,30,38,46]; 13 sub-national cross-sectional [10,41,91–93,55,62,63,70,75,88–90]; and 1 sub-national trends study [94] found water source and/or safety to be insignificant to child growth outcomes.

**Key Takeaway**: Water plays a pivotal role across interventions in a number of areas such as maternal and child nutrition, health care, sanitation, and hygiene. Though the large number of studies on the association between water source and safety and child growth outcomes at all levels (i.e. national, sub-national, community) in Ethiopia indicate mixed results, the importance of access to clean water cannot be understated, and future studies may be look to add more nuance to the understanding this relationship.

**Distance to fetch water**

A total of 8 studies examined the relationship between the distance or time involved in fetching water and stunting outcomes among children under-5 in Ethiopia. Of these, 2 sub-national cross-sectional studies by Dake et al in 2019 [15–30 min’ walk COR= 0.7 (0.5–0.9), >30 min’ walk COR= 0.5 (0.2–1.1)] and Yalew et al in 2014 [indicator not provided] found distance to be insignificant to stunting outcomes [95,96]. Of the remaining sub-national cross-sectional studies, 4 studies also found time to fetch water to be insignificant [10,40,82,97,98], while one study , conducted among irrigation and non-irrigation user households in Tigray, found time was significantly associated with stunting for children 6-59 months in both groups (among irrigation users: 31-59 minutes [AOR 1.854 (1.058, 3.249)]* and > or equal to 1 hour [AOR 2.549(1.458, 4.455)]; among non-irrigation users: greater than or equal to 1 hour [AOR 2.452 (1.446, 4.157)) [43] .

**Key Takeaway**: Based on this limited number of studies, the distance and time involved in fetching water are not significantly associated with under-5 stunting outcomes.

**Access to improved sanitation**

Similar to accessing improved water sources, access to improved sanitation was widely examined in the literature included in this review. A total of 34 studies looked at the relationship between sanitation and HAZ or stunting outcomes. This included a 2019 national trends study by Biadgilign et al which used data from the 2000, 2005, 2011 and 2016 DHS to show improved or modern sanitation was significantly associated with stunting outcomes for children 6-59 months [OR= 0.825, R. Sta. err= 0.041, p= 0.000)] [7]. This supports earlier findings from a 2016 study by Biadgilign et al which also found improved/modern sanitation to be significantly associated with stunting outcomes [β= -0.1867, SE = 0.0557, p = 0.001] [6]. A third national trends study by Headey et al in 2017 found that not having a toilet is linked to 7.7% of HAZ change in a decomposition analysis of 2000, 2005 and 2011 DHS data [23]. A 2018 systematic review and meta-analysis by Abate et al also found that WASH was reported in 5 studies as being a predictor of stunting reduction [53]. An additional 3 national cross-sectional studies reported similar findings related to the significance of improved sanitation on declining stunting outcomes [11,14,15,30]. Finally, 1 sub-national trends study [94] and 5 sub-national cross-sectional studies [56,59,62,70,99] also support this direction of association.

Conversely, 3 national trends studies did not find a significant association between improved sanitation and stunting outcomes. Included among these is a 2019 national trends study by Buisman et al which failed to find a relationship between stunting and use of any sanitation other than the practice of open defecation for children <24 months [β= -0.021, SE= 0.014] [24]. Additionally, a 2014 national trends study by Suri et al a 2017 found that among children under-5, improved latrines was not significantly associated with HAZ scores [37]. Another national trends study by Woodruff et al also found that HAZ was not significantly associated with sanitation based on latrine type (i.e. pit latrine versus toilets) [22]. Further supporting this lack of association were 5 national cross-sectional studies conducted between 2002 and 2019 [15,16,27,31,46]; a sub-national trends study conducted in 2017 by Dearden et al [94]; and 9 sub-national cross-sectional studies [10,41,57,71,75,76,88,92,100].

Key Takeaway: Though there were a large number of studies included in this review that tested the relationship between sanitation and HAZ or stunting outcomes, the majority of these found a significant association between improved sanitation and improvements in child growth outcomes. Disaggregating this general indicator into more explicit forms of sanitation could allow for greater ease of knowledge translation and a move towards more effective community-level interventions.

**Open defecation**

In contrast to access to improved sanitation, open defecation was only examined in relation to child growth outcomes in 3 studies included in this review. Among these, a 2017 national trends study by Woodruff et al found that for children 24-59 months, the prevalence of households practicing open defecation was a significant predictor of low HAZ scores (<=10%: b=-0.132, 11-26%: b=-0.234, 27-43%: b=-0.055, 44-63%: b=-0.082, 64-81%: b=-0.234, 82-92%: b=-0.242, 93-99%: b=-0.184 when compared to 100% (ref), p=0.028) [22]. Similarly, a 2019 national cross-sectional study using 2016 DHS data by Dessie et al found that for children 6-59 months, open defecation was significantly associated with increased stunting odds [AOR =1. 32 (1.06, 1.58)] [14]. In contrast, a 2014 national trends study by Headey et al found open defecation at the village-level to be insignificant to HAZ (b=0.00), stunting (b=-0.01), and severe stunting (b=0.005) [9].

Key Takeaway: The small number of studies in this review that examined the impact of open defecation on child growth outcomes suggest that there is a significant increase in stunting risk among households who practice open defecation, yet generalizing the results from these three studies may not be appropriate. Further research is needed to support these findings.

**Hygiene**

Similarly to open defecation, the impact of hygiene on child growth outcomes has been much less explored, perhaps due to difficulties in quantifying this factor. Overall, only 3 studies included hygiene in their analyses. Among these, a 2017 national trends study by Woodruff et al found that for children under-6 months of age, HAZ was not significantly associated with hygiene [22]. Similarly, in 2013, a sub-national cross-sectional study by Ngure et al found stunting not to be significantly associated with a hygiene index of 0-8 in SNNPR and Tigray regions [10]. This was further supported by a 2009 study by Gibson et al who also found hygiene was not associated with stunting in SNNPR [41].

**Key Takeaway**: Likely due to difficulties and imprecision in quantifying hygiene practices, this indicator has been tested by only a very small number of studies in this review. Of these, all found hygiene practices were not significant to child growth outcomes, though further research is needed in this area.

**Household crowding**

Household crowding can lead to competition for key resources such as food, as well as the spread of illness due to lack of space and unsanitary conditions. This in turn can have knock on effects for child growth outcomes, which helps to explain why 29 studies included in this review examined the relationship between family size and stunting or HAZ. Among these, smaller family size was found to be significantly associated with improved HAZ and/or less stunting outcomes in 12 subnational cross-sectional studies [8,44,101,102,56,59,64,69,78,79,97,99]. In one of these a subnational studies, conducted among children aged 24-59 months in the Dembia district of northwestern Ethiopia, the odds of stunting was 40% [AOR =1.4, 95 % CI: 1.1, 1.92), p<0.05] higher among children with a family size of more than four compared to children with less than or equal to four members [99]. Similar studies have also found evidence of increased family size and stunting risk within the southern regions of Oromiya [78,102].

In contrast, a 2016 national trends study by Biadgilign et al found that the number of members living in a household was not significantly associated with stunting among children 0-59 months (4-6 β =-0.0217, SE = 0.0582, p= 0.709; >7 members β = -0.0192, SE = 0.0652, p= 0.768) [6]. This finding is also supported in 2 national cross-sectional studies which assessed the association between stunting among under-5 children and households with 5 or more members [11,31]. An additional 11 sub-national cross-sectional studies also failed to find a significant relationship between household crowding and stunting outcomes [21,54,104,55,56,59,83,95,96,100,103]. Of these, 4 studies failed to find a significant relationship between family size (large vs. small; 4 or less vs 5-7+) and stunting among children aged 6-59 months [54,55,95,100]. These particular sub-national studies were conducted primarily in the South and Southwest regions of Ethiopia (i.e. Oromiya and Somali). Two additional studies found a non-significant association between family size and HAZ [21,103], while three other studies had similar non-significant results but focused on the northern regions of Ethiopia (i.e. Amhara and Tigray) [59,83,96].

**Key Takeaway**: In the large number of studies in this review that examined the impact of household crowding on child growth outcomes, the majority found no significant association between family size and stunting or HAZ. Given that a large number of these studies were cross-sectional and sub-national in nature, future research may focus on the longitudinal relationship between household crowding and child growth at the national-level for further insight.

**Feeding Practices and Food Security**

**Breastfeeding Trends**

*Ever breastfed, currently breastfed & frequency of breastfeeding*

Studies on the impact of breastfeeding for stunting outcomes among children under-5 in Ethiopia are numerous, yet the results are mixed. In terms of the impact of ever being breastfeed, a 2017 national trend study by Woodruff et al found that for children aged 6-23 months, having ever been breastfed was not significantly associated with HAZ, though values were not provided to support this conclusion [22]. Woodruff’s overall finding is supported, however, by 3 sub-national cross-sectional studies [10,105,106], but it is refuted by 1 national cross-sectional study [30] and 2 sub-national cross sectional studies [10,96]. In terms of the importance of currently being breastfed, 1 national cross-sectional study found not currently being breastfed was negatively associated with LAZ (length-for-age) [Adjusted β = -0.29 (-0.47, -0.11), p=0.003)], while another found that currently being breastfed was negatively associated with HAZ ( b = - 1.32, p < 0.001) [15,34]. While another sub-national cross sectional study did not find an association between currently being breastfed and stunting [ AOR = 0.75 (0.44,1.28)] One sub-national cross-sectional study found higher frequency of breast feeding to be associated with an increase in HAZ [(β = 0.019 & CI (0.006, 0.033), p value = 0.01)] [107], while a frequency of breast feeding less than 8 times within 24 hours was not found to be associated with stunting for children 0-23 months in another sub-national cross-sectional study (COR = 0.75; 95% CI: 0.46 -1.21) [108].

*Initiation of Breastfeeding*

In terms of initiation of breastfeeding, the aforementioned 2017 national trend study by Woodruff et al found that for children 6-23 months, child feeding, including initiation of breastfeeding, was not significantly associated with HAZ [22]. This lack of significance was further supported by a national cross-sectional study of 2005 DHS data that found early initiation of breastfeeding for children 0-24 months not to be associated with HAZ. Similarly, 1 sub-national trends study found no significance for both a delay of initiation of 1 hour after birth among those 6 months of age at the time of study [AOR 1.13 (0.79,1.63)] and those 12 months of age at the time of the study [AOR 0.85 (0.62,1.17)] [35]. A lack of significance for initiation of breastfeeding was also supported in 5 sub-national cross-sectional studies [10,62,71,103,109]. Conversely, 3 sub-national cross-sectional studies did find that late initiation of breastfeeding (i.e. more than an hour after birth) was associated with an increased odds of stunting for children under 59 months of age [54,83,110].

*Colostrum & pre-lacteal feeding*

Regarding the feeding of colostrum (the first form of milk produced by mothers after delivery which is rich in antibodies for the newborn), a 2016 systematic review of stunting literature on Ethiopia by Wirth et al found that not giving newborns colostrum was associated with stunting in several of the included studies [42]. This finding is further supported by 5 sub-national cross-sectional studies [2,60,69,111,112]. Conversely, 1 sub-national trend study [35] and 4 sub-national cross sectional studies [56,99,109] did not find colostrum feeding to be associated with stunting outcomes, however, indicating that the existing literature is divided on the impact of this practice in relation to child growth.

Related to this first form of feeding, pre-lacteal feeding, or the feeding of newborns something other than breastmilk after birth (e.g. water) was also found to have mixed effects on stunting in the existing literature on Ethiopia. A 2014 national trends study by Suri et al, which compared DHS data from 2000, 2005 and 2011, found that for children <6 months, giving food other than breastmilk within the first three days of life was not significantly associated with stunting OR=1.71 (0.96-3.05) [37]. This was further supported by a 2010 sub-national trends study by Medhin et al for children at both 6 months [AOR 1.21 (95% CI: 0.54,2.72)] and 12 months [AOR 0.99 (95% CI: 0.50, 1.95)] [35], and by 4 sub-national cross sectional studies [35,66,96,108]. However, 6 studies, including 1 sub-national trends study (AOR = 1.83; 95 % CI: 1.28, 2.61) [113] and 5 sub-national cross-sectional studies did find pre-lacteal feeding to be significantly associated with stunting for children under 59 months [95,98,100,112–114].

*Exclusive breastfeeding*

According to a 2019 national trends study by Hirvonen et al, in Ethiopia, exclusive breastfeeding rates improved between 2000 and 2016. In 2000, almost all children were breastfed in their first two months of life, though rates began to decline at the three month mark, when only about 40% of children were exclusively breastfed. By 2016, a decline in the rate of exclusive breastfeeding was seen at the four month mark, which indicates that children are being exclusively breastfed for longer compared to 2000 [18]. Despite this finding, however, like many of the other breastfeeding indicators, existing literature is also mixed on the significance of exclusive breastfeeding for HAZ and stunting outcomes in Ethiopia. A 2017 national trend study by Woodruff et al found that for children <6 months, HAZ was not significantly associated with child feeding, including exclusive breastfeeding [22]. Along with this finding, a national cross-sectional study by Disha et al that used 2005 DHS data found that exclusive breastfeeding under 6 months was negatively associated with HAZ (b= -0.80, p < 0.01) [34]. Along with this, 2010 sub-national trends study by Medhin et al found that non-exclusive breastfeeding at both 6 and 12 months of age was not significantly associated with stunting [35], and sub-nationally, 7 cross-sectional studies also found that exclusive breastfeeding was not significantly associated with stunting [56,67,71,83,115,116]. In support of an association between exclusive breast feeding and child growth outcomes, 5 cross-sectional studies did find a significant association between exclusive breastfeeding and stunting [56,63,84,103,109].

*Breastfeeding duration*

In terms of breastfeeding duration, a 2014 national trend study using DHS data from 2000-2011 by Suri et al found that for children 12-24 months and older than 24 months, still breastfeeding increased the child’s odds of stunting [(12-24: OR=2.48 (CI: 1.51-4.08) and >24: OR=1.90 (CI: 1.49-2.43), respectively]. For children 6-12 months, however, still breastfeeding was not significantly associated with stunting OR=3.26 (CI: 0.92-11.61) [37]. A 2016 systematic review of the WHO Stunting Framework by Wirth et al, which used Ethiopia as a case study, also found that breastfeeding past 12 months of age was associated with an observed increase in stunting prevalence. Along with this, one national cross-sectional study found increased duration of breastfeeding to be positively associated with stunting [28]; and another found that continued breastfeeding in children 12-15 months was negatively associated with HAZ (b= -1.66, p < 0.01) [34]. In contrast, another national cross-sectional study found increased duration of breastfeeding to be positively associated with HAZ [117], and one sub-national study found breastfeeding duration to not be associated with HAZ [78]. 4 sub-national studies found increased breastfeeding duration to be negatively associated with stunting [63,76,93,111,118], while 3 found increased breastfeeding duration to be positively associated with stunting [58,112,119], and one found no association [40].

**Key Takeaway:** Due to wide variation in the type and method of measurement for breastfeeding data, results of the association between breastfeeding and child growth in this literature review are largely mixed. While the positive impact of breastfeeding on child health and nutrition has been noted in several studies, further research on the impact of particular breastfeeding practices is required in order to make more definitive statements regarding their impacts on growth outcomes.

**Complementary Feeding**

Complementary feeding, which is usually initiated at 6 months of age, is important for diversifying children’s diets and giving them access to important nutrients for growth. Much of the existing literature on Ethiopia has examined the impact of complementary feeding on HAZ and/or stunting outcomes for children under-5. For example, a 2017 systematic review by Abdulahi et al concluded from the combined results of 10 cross-sectional studies that consumption of cereal-based complementary food was negatively associated with stunting [39]. 4 sub-national cross-sectional studies found that children who started complementary feeding before or after 6 months of age had an increased odds of stunting compared to those who started at 6 months [86,106,108,114], while one sub-national study only found early initiation of complementary feeding to be positively associated with stunting [105]. Another sub-national cross-sectional study found children who started complementary feeding at or after 12 months of age were at increased odds of stunting compared to those who started at 4-6 months [112].

One subnational study found children who consumed complementary food less than 3 times a day were at an increased odds of stunting compared to children receiving complementary foods at least 3 times a day ([AOR=3.01; 95%CI (1.48, 5.58), p=0.01). Children from families that used cereal-based complementary foods had statistically higher HAZ compared to those that did not according to another sub-national study [92]. The odds of stunting among orphans and vulnerable children (OVC) whose first food was porridge were found to be at an increased odds of stunting compared to OVC whose first complementary food was milk in one subnational cross-sectional study [74]. Only one study, a national cross-sectional examination of DHS data from 2005, found that introduction of solid, semisolid or soft food for infants 6-8 months was not associated with HAZ outcomes (b= 0.43) [34]

**Key Takeaway**: Of the studies included in this review that examined the relationship between complementary feeding and stunting or HAZ outcomes, nearly all found this type of child feeding to be significant for child growth. In particular, the timeliness of initiation and type of complementary food provided to children seems to have notable implications for either lower or raising the risk of stunting, and is an area of future research that could have significant policy implications.

**Food Security**

Given the history of famine and the impact of climate shocks such as drought and flooding on agricultural production in Ethiopia, a number of studies within the existing literature have examined the impact of food security on HAZ and stunting outcomes. Among these, one 1995 national cross-sectional study by Pelletier et al found that increases in cultivated land (used as a proxy for food security) led to statistically significant decreases in stunting prevalence for children ages 2-5 in ten regions (Arsi, Bale, North Omo, South Omo, West Gojam, North Gonder, South Gonder, East Shewa, West Shewa, Tigray) and increases in stunting prevalence in two regions (Kefa and Borena) out of a total of 22 regions. Additionally, for children 2-5 years old, stunting prevalence was found to decrease significantly as land cultivation increased among those growing cereals, cereals & roots, and cereals & cattle (respective chi-squared probabilities: 0.0000, 0.0000, 0.085) [120].

4 sub-national cross-sectional studies found household food insecurity to be associated with an increased odds of stunting [5,76,105,121] and 2 sub-national cross-sectional studies found food insecurity to be negatively associated with HAZ [10,104]. 3 sub-national cross-sectional studies [21,103,106] and another national cross-sectional study did not find significant associations [27]. Despite Ethiopia’s variable topography, only one study, by Hagos et al, examined the association between altitude and stunting outcomes, which was found to be insignificant, for both moderate stunting [OR = 1.00 Bayesian credible interval [BCI] (1.00–1.00)], and severe stunting [OR = 0.99 Bayesian credible interval [BCI] (0.99–1.00)] [5].

**Key Takeaway**: Of the studies that examined the association between food security and child growth outcomes, the vast majority found that household food insecurity was negatively associated with HAZ and positively associated with the risk of stunting. Interestingly, a small number of studies also examined land cultivation or altitude in relation to food security and found the associations with stunting to be negative in the case of increasing land cultivation and insignificant in the case of altitude.

**Immediate Causes**

**Dietary Intake**

Child dietary habits are directly related to chronic malnutrition. In terms of dietary diversity and frequency of meals, a 2017 national longitudinal study by Woodruff et al examined risk factors for under-5 stunting using DHS data from 2000-2011 and the WHO conceptual framework. The authors found that among children 6-23 months, dietary frequency, dietary adequacy, eating vitamin A rich foods, and consumption of iodized salt was not associated with HAZ [22]. Conversely, dietary diversity with a higher number of food groups consumed was found to decrease the odds of stunting by 27% among children between 6-12 months in a 2011 national cross-sectional study (OR=0.73, 95% CI: 0.56-0.95) [37]. Another national cross-sectional study conducted using DHS data from 2005 also found that dietary diversity was positively associated with HAZ (b = 0.23, p < 0.001), as was minimum acceptable diet among children ages 6-23 months (b = 0.43, p < 0.05); minimum diet diversity for children 6-23 months, minimum meal frequency for children 6-23 months, and consumption of iron rich/iron-fortified food for children 6-23 months were all found to be insignificant for HAZ outcomes in this study, however [34,122]. Two additional cross-sectional studies found that increased median dietary daily intake [41], meal frequency, dietary diversity, and vitamin A supplementation were significantly associated with reduced length-for-age stunting [15]. However, meal frequency, iron, hemoglobin levels, iodine in salt, and vitamin A supplementation were not significantly associated with height-for-age stunting in 4 other studies [29,31,72,123]. Another subnational longitudinal study which followed children under-5 and their respective caretakers between February 2014 and May 2015 in 9 districts in the regions of Oromiya and SNNP found that low dietary diversity (AOR = 3.10, 95% CI: 1.47-6.51, p<0.001) and medium child dietary diversity (AOR= 1.89 95% CI: 1.06-3.35, p<0.001) increased the risk of stunting [113]. To highlight the importance of maintaining an adequate dietary intake on reducing stunting risk, a national survey using the 2016 DHS found that children taking multiple micronutrients between the ages of 0-24 months was associated with a 49% decreased odds of being stunted (0-24 months: [OR= 0.51, 95%CI: 0.27-0.93]; 25-59 months: [OR= 1.43, 95%CI: 0.99-2.09]) [29]. In a 2016 study by Fentahun et al., findings suggest that not feeding children specials foods while they were ill further increased the risk of stunting (AOR= 1.78, 95% CI: 1.16-2.74, p<0.001) [113].

With milk and dairy-based sustenance serving as an important part of a child’s development, milk consumption in Ethiopia was found to be highly dependent on cow ownership [124]. A study examined the effects of cow ownership on child growth using the DHS 2000 and the Agricultural Growth Program Survey of 2011, which included the regions of Amhara, Oromiya, SNNP, and Tigray. They found that households owning cows improved child HAZ and reduced the risk of stunting among children between the ages of 12-18 months and 12-24 months (HAZ: b=0.471, SE: 0.153, p<0.01, Stunting: b=-0.133, SE: 0.036, p<0.01, HAZ: b= 0.324, SE: .117, p<0.01, Stunting: b=-0.10, SE: 0.03, p<0.01, respectively). However, among children between the ages of 0-12 and 18-60 months, these relationships were not significant [124]. Additionally, while controlling for the effects of asset ownership, agricultural income, agro-ecological characteristics, and zone (woreda), households owning cows was not associated with HAZ among children 12-24 months [124].

**Key Takeaway**: Overall, these studies highlight the importance of improved dietary intake, dietary diversity, and consumption of multiple micronutrients, particularly among children at the early stages of infancy. Combining the impact of adequate dietary recommendations and exclusive breastfeeding could further improve the effect of stunting reduction as children develop.

**Disease**

Only a handful of studies examined the effects of disease on the risk of stunting. For example, a national longitudinal study examined risk factors for under-5 stunting using the 2000-2011 DHS and the WHO conceptual framework to guide analyses. They found that among children under-6 months, child morbidity was not associated with HAZ [22]. Additionally, a subnational study conducted in Butajira in the Gurage Zone of SNNPR followed a cohort of children between 0-12 months over a year and did not find any association between severe illness in the first 2 months of life and child stunting at 6 or 12 months of age [35].

**Key Takeaway:** Based on the very small number of studies included in this literature review, no significant associations between stunting and HAZ was found among diseases in general. Follow-up studies are needed to validate these non-significant findings.

**Anemia**

A national study used the 2011 DHS to assess the effects of anemia on stunting and found that children with mild anemia were at a 43% higher risk of stunting (AOR = 1.43, 95% CI: 1.24, 1.64), and that the risk of stunting increased with the severity of the anemia. Those who had moderate anemia were at a 76% higher risk of stunting and those with severe anemia were at a 3 times greater risk of stunting as compared to non-anemic children (1.76, 95% CI: 1.52, 2.03, AOR= 3.23, 95% CI: 2.35, 4.43, respectively) [11]. Furthermore, one other cross-sectional study which assessed the association between maternal anemia status and child undernutrition found that having a mothers with anemia is a risk factor for under-5 stunting. A cross-sectional study using 2016 DHS data found that children between 0-24 months had significantly increased odds (59%) of being stunted among mothers aged 35 years or over compared to mothers between the age of 25-34 years (25-34 years: OR= 1.12, 95%CI: 0.91-1.4; 35 ≥ years: OR= 1.59, 95%CI: 1.17-2.16) [29]. From this, anemia was found to significantly increase the likelihood of stunting among children aged 25-59 months (0-24 mos: OR= 1.13, 95%CI: 0.93-1.36; 25-59 mos: OR= 1.42, 95%CI: 1.15-1.76) [29]. In addition, a recent cross-sectional study using the 2016 DHS found that children of anemic mothers were 1.18 times more likely to be stunted compared to children of mothers without anemia (AOR= 1.18, 95%CI: 1.06-1.32) [14]. In contrast, another national cross-sectional study found that maternal anemia was not associated with stunting among children between the ages of 0-24 months (OR= 1.14, 95%CI: 0.94-1.40) and 25-59 months (OR= 1.25, 95%CI: 0.82-1.91) [29].

**Key Takeaway:** The majority of these studies demonstrate a dose-response relationship between anemia severity and stunting risk (i.e. higher anemia severity results in increased stunting risk). Interventions should be directed towards regions with the highest prevalence of maternal anemia in order to improve maternal nutrition and limit the risk of stunting.

**Diarrhea**

A national longitudinal study using DHS 2000-2011 found that children between 24-59 months who had diarrhea in the past 2 weeks had lower HAZ (b=0.27, p<0.001) as compared to those without diarrhea [22]. This was supported by another study that used the 2011 DHS and found that children having diarrhea in the last 2 weeks increased the odds of stunting among children over the age of 24 months (OR=1.54, 95% CI: 1.12-2.10) [37]. Additionally, a study conducted in Dubluk and Elka in the Oromiya region in 1990 found that low HAZ was correlated with increased diarrheal disease incidence (r = -0.21, P=0.01) in Elka (n=231) but not in Dubluk (n=197) [125]. This may be attributed to differences in sample sizes or zonal differences between the two areas with Elka being an agricultural area, while Dubluk is a semi-nomadic pastoralist area [125].

Key Takeaway: Diarrhea is an important risk factor for increased stunting outcomes, based on the studies included in this literature review. Longitudinal research or historical data on diarrheal incidence beyond a 2 week period could further inform future interventions.

**Acute Respiratory Infection**

A recent cross-sectional study used the 2016 DHS to explore the determinants of acute respiratory infection (ARI) among children under-5, showing that moderately stunted children were at a 24% increased odds of developing ARI compared to normal children (AOR= 1.24, 95% CI: 1.01 – 1.54) [126]. In addition, a subnational longitudinal study followed children under-5 and their respective caretakers between February 2014 and May 2015 in 9 districts in the regions of Oromiya and SNNP. Stunting was found to be a predictor of having any child morbidity or an acute respiratory infection (AOR = 2.55, 95% CI: 1.11–5.89, p<0.001 and AOR = 3.23, 95% CI: 1.39–7.52, p<0.001, respectively) [113]. One cross-sectional study found that acute respiratory infection, diarrhoea, and general morbidity were not found to increase the risk of stunting (AOR= 1.28, 95% CI: 0.99–1.83, AOR=0.79, 95% CI: 0.41–1.51, AOR=1.05, 95% CI: 0.76–1.44, respectively) [113], and two additional cross-sectional studies failed to find an association between history of infection and stunting (HAZ and LAZ) [15,72].

**Key Takeaway:** An assessment of the study results show that most studies treat ARI as an outcome, with under-5 stunting acting as a significant predictor and/or risk factor. The lack of studies looking at the reverse relationship (i.e. ARI as the main predictor and stunting as the primary outcome) begs further investigation on whether this association is linear, or comorbid with other disease determinants.

**Fever**

A national longitudinal study using 2000-2011 DHS data found that children between 24-59 months who had a fever in the last two weeks had lower HAZ (b=0.15, p=0.006) [19]. Additionally, a national cross-sectional study using only the 2011 DHS found children having a fever in the last 2 weeks was associated with stunting among children over the age of 24 months (AOR 0.76 CI: 0.61-0.96) [37]. This was supported by another cross-sectional study using the same survey, where children under-5 that did not have fever 2 weeks prior to the survey had between ~10%-12% (OR=0.909, p=0.0099; OR= 0.8800, p= 0.45, respectively) reduced stunting odds as compared to those with fever [14]. Lastly, a study conducted in the Oromiya region in 1990 failed to find an association between risk of fever with height-for-age in Elka and Dubluk [125].

**Key Takeaway:** These studies demonstrate that fever still poses a slight risk towards increased stunting outcomes among under-5 children. Combined with other ailments, this determinant may escalate as a serious risk factor over the course of a child’s development. Frequent monitoring, reporting, and in-person assessments of fevers could help public health workers track areas in need of treatment.

**Maternal Characteristics**

**Maternal Age**

A substantial amount of the literature included in this review looked at the association between maternal age and stunting outcomes for children under-5 within Ethiopia. A national trend study using DHS 2000-2016 data found that having a mother aged between 40-44 and 45-49 was significantly associated with reduced odds of stunting among children under-5 (40-44 years: OR= 0.768, SE= 0.75, p=0.007, 45-49 years: OR= 0.680, SE= 0.078, p= 0.001) [7]. In a national cross-sectional study by Gebreegziabher et al, it was found that among mothers whose first birth was between 20-29 years, there was a significant reduction in stunting odds (OR= 0.87, 95%CI: 0.76-0.99) for children 25-59 months, yet this association was insignificant when observed among children under 24 months (OR= 0.90, 95%CI: 0.77-1.06) [29]. A further six national studies and one subnational study failed to demonstrate a significant relationship between maternal age and stunting risk [1,6,7,11,24,35,36]. Included in these was a 2016 national trend study by Biadgilign et al that used 2000-2011 DHS data and failed to find an association between women’s age and stunting among under-5 children for a wide range of maternal age groups (age 20-24 [β = 0.0459, SE = 0.0849, p=0.589], age 25-29 [β = -0.0089, SE = 0.866, p=0.919], age 30-34 [β = 0.0064, SE = 0.0918, p=0.945], age 35-39 [β = -0.0718, SE = 0.0953, p=0.451], age 40-44 [β = -0.1297, SE = 0.1047, p=0.215]) [6]. Similarly, a subnational conducted in Butajira in the Gurage Zone of Ethiopia’s SNNPR followed a cohort of children between 0-12 months over a year and did not find any association between maternal or paternal age and stunting among 6 month and 12 month old infants [35].

**Key Takeaway:** The vast majority of the studies included in this literature review failed to find a relationship linking maternal age or age of mothers at first birth to stunting risk.

**Maternal Height**

Maternal height has been shown to predict child height in a number of studies. A national longitudinal study aimed to examine risk factors for under-5 stunting using the 2000-2011 DHS and the WHO conceptual framework to guide analyses. They found that maternal height was associated with child HAZ among children under 6 months, between 6-23 months and between 24-59 months (b=0.04, p<0.001, b=0.05, p<0.001, b=0.05, p<0.001, respectively) [22]. Four cross-sectional studies found significant associations between shorter mothers and increased stunting risk [27,29,36,37]. In particular, one national study found that mothers who were shorter than 145 cm had a 1.52 times increased odds of having stunted children (<145 cm AOR= 2.52, 95% CI: 1.81-3.51) [36]. This was further supported by a national cross-sectional study of 2005 DHS data that also found maternal height to be positively associated with HAZ (b = 0.04, p < 0.01) [34]. In addition, a subnational cross-sectional survey found mothers of stunted children were significantly shorter (height difference of 3.64 cm [95% CI 1.42, 5.85]) and lighter (weight difference of 2.74kg [95% CI: 0.63, 4.85]) as compared non-stunted children between the ages of 6-23 months living in rural communities in SNNP [41]. Interestingly, a national study using DHS data from 2000-2011 found that children of mothers with a height below 145 cm [β= -0.9254, SE = 0.1072, p<0.001] were at a lower risk of stunting compared to those whose mothers were taller than 145 cm [6]. However, on the contrary, a national trend study using DHS 2005-2011 data found that mothers taller than 150 cm are less likely to have stunted children (mother taller than 150 cm: β= -0.102, SE= 0.018, p<0.01) [24]. Another national study supported this relationship, showing a positive association between maternal height and under-5 child HAZ (b=0.04, SE: 0.002, p<0.01) [23]. However, these findings were nuanced by a subnational study conducted in Butajira in the Gurage Zone of Ethiopia’s SNNPR which found the association between maternal height and child stunting outcome to be insignificant [35]. In terms of the impact that maternal height has on stunting and undernutrition, Silva (2005) argues that the intergenerational effect of maternal height and stunting risk exists, but is not expected to directly influence the development of a child relative to the child’s nutritional status, for example [27].

**Key findings:** These studies found a general increase in stunting outcomes among mothers who were shorter than 145 centimeters. It is also important to highlight the significance of the inter-generational effects of maternal short stature, which should prompt action towards long-term maternal nutrition programs.

**Maternal BMI**

Most studies are in agreement regarding the influence of maternal BMI on the risk of chronic malnutrition to their children. One cross-sectional study using DHS data from 2011 found that children of underweight mothers (BMI<18.5 kg/m^2^) were at an increased risk of stunting (AOR=1.13, 95% CI: 1.00, 1.28) while children of overweight mothers (BMI>25 kg/m^2^) had a reduced risk of stunting (AOR=0.69, 95% CI: 0.52, 0.90) as compared to children of normal weight mothers (BMI: 18.5-24.9 kg/m^2^) [11]. Three other cross-sectional studies support the relationship between low maternal BMI (BMI<18.5 kg/m^2^) and increased stunting risk [1,14], showing similar trends even when using LAZ as the measure for stunting [15]. Two additional cross-sectional studies parallel these results [29,127], with one study showing a 75% reduction in stunting odds among overweight mothers with a BMI≥25 kg/m^2^ compared to those with BMI between 18.5-24.9 kg/m^2^ (BMI ≥ 25: OR= 0.25, 95%CI: 0.11-0.55, p<0.01; BMI 18.5-24.9: OR= 0.72, 95%CI: 0.49-1.05) [29]. One cross-sectional study found a positive association between maternal BMI and child HAZ among children 6-23 months and 24-59 months (b=0.05, p<0.001, b=0.05, p<0.001, respectively) [22]. Three studies refute these associations, two of which failed to find an association between maternal BMI and child stunting or HAZ outcomes [27,34], while another found that maternal BMI was not a significant predictor of stunting among women with BMI less than 18.5 kg/m^2^ (AOR= 1.09, 95%CI: 0.98-1.21) [36].

**Key Takeaway:** Overall, a majority of studies in this review support the association between underweight mothers (BMI<18.5kg/m^2^) and increased stunting risk. Suggestions for early and preventative nutritional programs targeted at underweight mothers could help to reduce the risk of chronic malnutrition among under-5 children.

**Parity/Inter-pregnancy Interval**

Increasing inter-pregnancy intervals allow women to recover fully before becoming pregnant again and consequently may lead to improvements in child growth outcomes. Three national cross-sectional studies provide evidence to support a reduction of stunting risk among increasing maternal birth intervals, particularly among children 24 months and above [16,36,37]. Among these, a national cross-sectional study using the 2000 DHS found that increases in the preceding birth interval from under 24 months, 24-25 months, and finally to 36-47 months decreased the risk of stunting (< 24 months: OR = 1.89, 95% CI: 1.58, 2.27, p = 0.001, 24-35 months: OR = 1.60, 95% CI: 1.37, 1.88, p = 0.001, 36-47 months: OR= 1.52, 95% CI: 1.28, 1.79, p = 0.001, respectively as compared to 48 months or higher) [16].

In terms of parity, or the number of births per woman, a national trend study using DHS data from 2000-2016 found that under-5 children born to women who had given birth three or more times have a 13% increased odds of being stunted (OR= 1.1333, SE=0.046, p=0.002) [7]. Using DHS 2011 survey data, another national cross-sectional study found a significant association supporting the link between women who had two or more births 2 and increased stunting risk among their children [11]. Only one cross-sectional study using 2016 DHS data found that maternal parity of 4 births or more was not associated with stunting among children 0-24 months (parity 4-6: OR= 1.01, 95%CI: 0.82-1.24; parity 7: OR= 0.76, 95%CI: 0.57-1.02), as well as children aged 25-59 months (parity 4-6: OR= 0.94, 95%CI: 0.80-1.10; parity > 7: OR= 0.92, 95% CI: 0.74-1.13) [29]. Lastly, a study using DHS 2011 found that twin births were twice (OR=1.99, 95% CI: 1.46, 2.72) more likely to lead to severe stunting compared to single births [28]. This was supported by a study that used the 1995/6-1998 Welfare Monitoring Surveys and found that on average twins were shorter by 0.42 SDs [44].

**Key Takeaway**: A large proportion of the literature provides evidence to suggest that increasing inter-pregnancy interval and fewer births per woman results in a reduction of stunting outcomes among children under-5.

**Child Characteristics**

**Age**

A number of studies support that the risk of chronic malnutrition increases with age. One national trend study using DHS data from 2000-2011 found that stunting was highest among children between 2-3 years of age (1 years: β = 1.5375, SE = 0.0507, p<0.001), 2 years: β = 1.8906, SE = 0.0511, p<0.001), 3 years: β = 1.7545, SE = 0.0501, p< 0.001), 4 years (β = 1.5485, SE = 0.0506, p<0.001) as compared to children under 1 year of age [6]. Similarly, a sub-national cross-sectional study conducted in the North Wollo Zone of Amhara region also found that age was significantly associated with stunting among children 2-5 years compared to children under age 2 ([AOR 2.49 (1.23, 5.05), p=0.001] [45]. In further support of this conclusion, a number of national cross-sectional studies using DHS data ranging from 2000-2016 found that with increasing age, children were more likely to be stunted [11,12,16,27,31,44]. Among these, several national cross-sectional studies found ~5-10 times increased stunting odds (including measures of length-for-age) among children 24-35 and 35-59 months [1,14,15,30,36,128]. Based on cross-sectional 2016 DHS data, under-5 children 12 months and above were shown to have an increased odds of stunting compared to children between 6-11 months (6-11 months: AOR= 1.27, 95%CI: 0.95-1.69; 12-23 months: AOR= 5.04, 95%CI: 3.95-6.41, p<0.001; 24-59 months: AOR= 10, 95%CI: 7.71-12.98, p<0.001, respectively) [1]. In contrast to this, however, three national trend studies found that children 2-3 years of age were not at a higher risk of stunting compared to children outside this age range (<2 and ≥3) [13,22,37]. Two subnational studies in Ethiopia’s Oromiya (Elka village) and SNNP regions support these trends [113,129]. Only two national cross-sectional studies demonstrate a negative association between child age and height-for-age [33,72]; and a third found no association between child age and HAZ outcomes [34].

**Key Takeaway**: Studies emphasize the significant relationship between increasing child age and increased stunting outcomes. Healthcare efforts towards specific child age-groups and the most vulnerable (i.e. newborns and infants) may diminish the chronic effects of stunting as children grow older.

**Gender**

Within this literature review, three national trend studies found that male children having higher stunting risk when compared to females [6,7,18]. Five national cross-sectional [11,14,34,36,127] and three sub-national cross-sectional studies [35,113,129] support this finding that male children have an increased likelihood of becoming stunted or having lower HAZ compared to females. A handful of these studies found that males are at a higher risk of severe stunting based on DHS 2011 data, in addition to male children having between 14%-20% increased odds of moderate stunting compared to females [11,14,36,127]. Using the 2004 Welfare Monitoring Survey (WMS), another study had consistent results concluding that males were at a 10% higher risk of being stunted as compared to females (n=8827) [13]. However, one subnational longitudinal study followed children under-5 and their respective caretakers between February 2014 and May 2015 in 9 districts in the regions of Oromiya and SNNP. In this study, female children were nearly 5 times more likely to be stunted as compared to their male counterparts (AOR=4.99, 95% CI: 2.62-9.53, p<0.001). The authors attributed this finding due to sex discrimination with regards to the distribution of food in developing countries [113].

Four additional cross-sectional studies parallel the increased stunting risk among male children, with these studies showing reduced stunting risk among younger under-5 females between the ages of 6-24 months [1,29,30,37]. One cross-sectional study using 2016 DHS data found that female children under-5 were ~15% less likely to be stunted compared to males (female OR= 0.84, p<0.001) [30]. In addition, female children were at a lower odds of stunting among children 6-12 months (OR= 0.61, 95% CI: 0.38-0.97) and 12-24 months (OR= 0.65, 95% CI: 0.48-0.87), but not for those below 6 months (OR= 0.77, 95% CI: 0.44-1.33) or above 24 months (OR= 0.94, 95% CI: 0.79-1.12) [37]. Lastly, significant associations were also found between under-5 gender and LAZ [15,33,72], and one national longitudinal study using DHS 2010-2011 found that males (<6-23 months) had lower HAZ compared to females [22]. Only one cross-sectional study using the DHS 2000 did not find an association between child gender and stunting [27].

**Key Takeaway:** The trend shown across the vast majority of these studies demonstrates that under-5 male children are generally more likely to be stunted compared to their female counterparts. Significant gender differences in stunting outcome may indicate underlying factors such as sex discrimination, differential food allocation, and food scarcity which help to explain a child’s nutritional status.

**Low Birthweight**

Three national cross-sectional studies found evidence to support the association between low birthweight and increased stunting risk, particularly among under-5 children born with small to average birth sizes [1,29,37]. One national cross-sectional survey using the 2016 DHS found that among children aged between 0-24 months, children with a small birth size had a 71% increased odds of being stunted compared to children with average birth weight (small bw: OR= 1.71, 95% CI: 1.37-2.12; average bw OR= 1.22, 95%CI: 1.00-1.49) [29]. Furthermore, birth size was also found to be significantly associated with length-for-age as a measure of child stunting (small adjusted β= -0.45, 95%CI: -0.62, -0.29), p<0.001; average adjusted β= -0.24, 95%CI: -0.39, -0.09, p=0.002) [15]. In contrast, a cross-sectional study by the same authors found small or average birth size was negatively associated with low mean height-for-age (small β= -0.62, 95%CI: -0.78, -0.45, p<0.001; average β= -0.26, 95%CI: -0.41, -0.10) [72]. Evidence is unclear on whether large birth sizes is associated with HAZ based on three national studies looking at under-5 children and large birth size [14,22,34]. However, two cross-sectional studies found that birth size was not found to be a risk factor of stunting among these age groups [35,37]. A subnational study conducted in Butajira in the Gurage Zone of SNNPR which followed a cohort of children between 0-12 months over a year did not find any association between low birth weight (<2500g) and stunting at 6 months (AOR=1.69, 95% CI: 0.81,3.50) [35]. Along with this, another sub-national study conducted in northern Tigray among irrigation and non-irrigation using households found only medium birth size of children 6-59 months in non-irrigation households to be significant for stunting [AOR 0.267 (0.144, .498)], while large birth size was found to be insignificant for all households [43] .

**Key Takeaway:** Children born with a low birthweight have an increased risk of stunting outcomes and have a lower mean HAZ compared to under-5 children of average or higher birthweight. A focus on both prenatal and postnatal interventions for both the mother and infant would aid in reducing low birthweight complications among newborns.

**Birth Order**

Across 3 national trend studies and 3 cross-sectional studies, only one, a national trend study using DHS data from 2000-2011 found that increasing birth order for children between 24-59 months decreased HAZ (b=-0.08, p=0.001) [22]. Using the 2004 Welfare Monitoring Survey (WMS), a subnational study examined the effects of the number of siblings on the risk of stunting among under-5 children (n=8827). They found that the presence of one sibling significantly increased the odds of stunting, by 24%, while the presence of at least two competing siblings increased the odds of stunting by 33% [13]. On the other hand, a national study using the same surveys found no association between birth order and HAZ, stunting or severe stunting among children under-5 (b=-0.01, b=0.00, b=0.004, respectively) [9]. This is further supported by a national trend study showing no association between birth order or birth intervals greater than 24 months and stunting risk (Birth order: β= 0.001, SE= 0.004, Birth interval > 24 months: β= -0.022, SE= 0.019) [24]. Lastly, three cross-sectional studies failed to find a relationship between birth order (2^nd^ to 6^th^), last birth, and stunting risk [1,14,16].

**Key Takeaway:** Birth order was not found to be significantly associated with stunting outcomes or HAZ. Immediate determinants such as current household crowding, socio-environmental status, hygiene, etc. could be attributed in explaining the increased odds for stunting among children with competing siblings found in one study. More research is required to verify these findings.

**Number of Children among Households**

Two national trend studies using DHS data from 2000-2016 found that households with more than two children under the age of five years were at a higher risk of stunting compared to households with two or fewer under-5 children [6,7]. Among seminal national cross-sectional studies using the 2000 DHS, one found that increasing the number of children under-5 was positively associated with stunting (b=0.025, SE: 0.009, p<0.01) [27]. Similarly, using DHS data from 2005, a study by Disha et al found that the number of children in a household was negatively associated with HAZ (b = - 0.32, p < 0.01) [34]. Additionally, a national study using 2011 DHS data supported this finding, showing that households with increasing number of children under-5 increased the risk of stunting [11]. However, a different national study found a 13% reduced odds in stunting among households with more than two children under-5 (>2 OR= 0.879, SE= 0.036, p= 0.002) [7]; and a subnational study conducted in Butajira in the Gurage Zone of SNNPR followed a cohort of children between 0-12 months over a year and did not find any association between number of children under-5 and stunting at 6 months and 12 months of age [35].

**Key Takeaway:** The majority of these studies show that the number of children under-5 years in a household impacts growth outcomes, including an increased risk of stunting.

# **Supplementary Appendix 3:** Multivariable Analyses Methods

Linear Multivariable Regression (Difference-in-difference Analysis)

We undertook linear multivariable regression analyses, and included all covariables and adjustment factors as fixed effects. We added interaction terms between each potential determinant and time (i.e. time*covariable interaction terms), which signify whether a change in a proposed predictor of HAZ leads to a change in HAZ over the studied time period. The four cross-sectional surveys used in this analysis were assembled into panel datasets, and difference-in-difference (DID) analyses were used. Univariate statistics were estimated using means/standard deviations and frequencies/proportions as appropriate. We used the interaction estimators in unadjusted and adjusted regression methods to estimate the DID effect. The general model specification included an interaction term between time and the various indicators. The multivariable regression models were adjusted for child age, sex and region. Effect estimates were reported with 95% confidence intervals. All statistical analyses were performed using Stata version 14.0. The complex sampling design of DHS surveys was taken into account by using the STATA's svyset function. Standard errors were estimated using the Taylor series linearization method, which incorporates sampling weight, primary sampling unit, and stratum appropriate to the DHS sample design.

To examine the association between HAZ and various indicators, we conducted a series of step-wise linear regression models. A hierarchical modelling approach using distal, intermediate and proximal level variables was executed as suggested by Victora 1997 (54) to generate the final multivariable models. Variables within each level were selected from our general conceptual framework as defined in Figure 8. Step 1 was a series of bivariate regressions to determine crude associations between indicators in our conceptual framework and HAZ outcome. Step 2 was to use all candidate variables for multivariable model building (i.e. with p-value ≤0.20) irrespective of their direction to move forward for multivariable modeling. Selected variables are entered into backward stepwise elimination modeling within their respective levels and those with p-values <0.15 are retained. At each step, the crude and adjusted associations between the indicator and HAZ was analyzed for statistical significance. Multicollinearity among adjustment variables was evaluated using variance inflation factors (VIF) where VIF>3 were considered suspect for collinearity.

Oaxaca-Blinder Decomposition

We also undertook the commonly used Oaxaca-Blinder decomposition methods [130,131] to assess determinants of nutritional change over time in Ethiopia. These methods based on individual-level data have high statistical power and have been widely used to assess nutrition determinants in low and middle income settings [22,131–133].

We analyzed individual-level data from four rounds of Ethiopia’s DHSs: 2000, 2005, 2011, 2016. Our analysis focused on the index mother-child pair from each survey round. Defined as the youngest child of the youngest mother in each household, selection of an index pair simplifies the model and interpretation, and is common practice in advanced analysis of DHS datasets. The total number of index pairs available from each survey were n=5975, n=2600, n=6561, and n= 6184 for DHS 2000, 2005, 2011, and 2016, respectively. A flow chart outlining sample size breakdown during the index pair selection process is presented in Appendix 4. Given that the dietary needs/practices and growth trajectories of children in the first 1000 days of life vary notably from children beyond 2 years of age, it has been suggested that these two cohorts be analyzed separately to unmask true effects of environmental conditions and other factors on undernutrition. We conducted analyses for the entire under-5 year child population, the 24-59 month child population, and the 6-23-month population [134]. The <6-month population had too small a change in HAZ, thus rendering the results of analysis not meaningful. Thus, only the decomposition results for the under-5, 6-23-month and 24-59-month populations are presented.

We used the continuous formulation of HAZ (as opposed to categorical stunting) as the dependent outcome to strengthen statistical power of the analyses. Linear least square regression models -accounting for survey design and weights - were used to assess associations between $\boldsymbol{y}_{\boldsymbol{i,t}}$, our outcome variable measured for a child *i* at time *t*, a vector of time-varying determinants (**X**), time-invariant child age and sex control variables (**C**), and a survey round time variable (**T**) to capture any trend effects. Collectively, with the standard error term, the model is expressed in Equation 1.

$\boldsymbol{Y}_{\boldsymbol{i,t}}=\boldsymbol{\beta}\boldsymbol{X}_{i,k}+\boldsymbol{C}_{i}+\boldsymbol{T} + \varepsilon_{i,t}$ [Equation 1]

The conceptual framework and corresponding list of covariables, their data sources, and definitions used in decomposition assessment are included in Appendix 5. Applying the conceptual framework, we used a similar hierarchical modelling approach (as described for DID analysis) whereby we examined the distal, intermediate, and proximal level determinants of HAZ.

Equation 1 was applied to derive β coefficients for determinants (DHS 2000 – DHS 2016). To explain the relative contribution of each covariable over time to HAZ change, we used the Oaxaca- Blinder decomposition under the assumption that the β coefficients are the same across the two populations and the error term has the mean zero. Using the estimated parameters from Equation 1 and the (weighted) means of explanatory variables in the two time points, we applied Equation 2 (e.g. for years 1992/93 to 2017) to obtain the predicted change in HAZ due to the change in each determinant [135].

$$\Delta\bar{Y}_{i,t}=\beta\left( \bar{X}_{2016}-\bar{X}_{2000} \right)$$

The product coefficients for individual determinants were subsequently ranked to identify the relative contribution of each factor to HAZ change. Like determinants were also grouped into broader domains for interpretation. We examined variance inflation factors (VIF) to assess multicollinearity between variables whereby a VIF > 3 was considered suspect of high inter-variable correlation. For model building, a p-value <0.20 was considered statistically important and variables with p< 0.15 were retained in the final hierarchical multivariable models. All analyses were carried out in Stata version 14.0.

# **Supplementary Appendix 4:** Qualitative Data Collection and Analyses Methods

Qualitative Inquiry Process

The qualitative component of the case study aimed to understand the drivers of stunting reduction among children in Ethiopia through exploring the perspectives of key national stakeholders in the development and implementation of relevant policies and programs, the experiences of community health workers and mothers in the community. Specific qualitative research objectives included:

1. To explore nutrition-specific and –sensitive key events (policies/strategies/programs/guidelines) in Ethiopia that may have contributed to a reduction in child stunting;
2. To identify important contextual factors that have functioned as enablers/drivers and barriers to reduction of stunting in Ethiopia; and
3. To document community-level insight and experiences on the stunting transition in Ethiopia from community/volunteer health workers and mothers of young children.

The conceptual framework by Black et al., informed the development of an adapted framework (**Figure 2**), the design of the in-depth interview and focus group discussion guides, as well as analysis and interpretation of the qualitative data. Our qualitative data collection tools were also informed by existing literature and nutrition questionnaires; for example, the International Food Policy Research Institute’s nutrition-focused qualitative data collection toolkit was consulted and relevant tools were adapted to our research objectives as appropriate. Data was analyzed using key themes including: basic causes, underlying causes, and immediate causes of reduction in stunting and malnutrition.

Qualitative Research Design

We undertook three independent research activities to inform study objectives. At the first stage, national stakeholders were interviewed to provide insight and expertise on objectives 1 and 2. This top-down approach aimed to solicit macro-level perspectives and experiences in health and nutrition in Ethiopia. All the experts were interviewed in Addis Ababa, where main administrative structures and institutions are located. To understand how individuals in the community received and implemented major nutrition-specific and –sensitive policy/program events and their experiences in the nutritional transition as a whole, we consulted childcare workers in the community (e.g. at schools, health facilities, etc.) and the mothers of these children. These latter two research activities largely informed objectives 2 and 3, but also shed light on objective 1.

*Sampling and Recruitment Strategy*

Participants were identified and selected using purposive sampling strategies [136], including snowballing sampling [137]. National stakeholders were purposively selected due to their involvement in the design, implementation, monitoring or evaluation of nutrition-specific or –sensitive policies and programs (**Appendix Table 1**). Key informants were asked to identify and refer the research team to other individuals with knowledge and expertise in the area of nutrition, policy, and stunting reduction. Participants were recruited by phone and a follow-up email was sent to request their participation in the study.

At the district level, key informants were purposively selected based on their experiences working in communities for 10-15 years, and for working in the areas of health, education and agriculture. To obtain focus group discussion (FGD) participants, a set of core inclusion characteristics were identified. These included:

1. Women who had children born between 1987-1991, 1995-1999 and 2011-2015;
2. Those able to express their opinion freely;
3. Those who were willing to participate in the interview; and
4. Those who were from different geographical locations within the district.

In the focal areas, both the mothers of children over the different periods and the key informants were identified through support from the District Administration Health Office and via Health Extension Workers (HEWs). These sampling strategies helped to ensure that a range of diverse perspectives at national and community-levels were captured.

## **Supplementary Appendix Table 1**: Inclusion Criteria

| **Type of Stakeholder** | **Inclusion Criteria** |
| --- | --- |
| National Stakeholders | - Key informants with extensive experience in and knowledge of design, implementation and evaluation of nutrition-specific and –sensitive policies and programs in Ethiopia. Examples include: national policymakers (e.g., Ministry of Health, Ministry of Education, etc.), bilateral/multilateral organization (e.g., UNICEF), international/local NGOs (e.g., Save the Children, Nutrition International, Alive and Thrive, World Vision, etc.) and academics. |
| Regional Stakeholders | - Paid/voluntary community stakeholders in the Southern Nation, Nationalities, and Peoples (SNNP) and Somali regions. Examples include: Health Extension Workers (HEWs), health surveillance personnel, Expanded Program on Immunization (EPI) implementers, maternal, newborn and child health workers, and teachers. |
| Mothers in Communities | - Mothers of children born in 1987-1991; - Mothers of children born in 1995-1999; - Mothers of children born in 2011-2015; and - Currently living in SNNP and Somali regions. |

Semi-structured with regional respondents and focus group interviews with mothers were conducted in two regions: SNNP and Somali (Figure 9). These two regions were selected based on their geographic location, ability to capture urban and rural perspectives, as well as the substantial progress made in these regions to reduce stunting among children. Within these two regions, Yeki and Bonga districts in SNNP and Aware and Harshin districts in Somali were selected as particular areas of focus based on the substantial progress in the reduction of stunting made in these districts compared to other districts. Focus communities were purposively selected, using convenience sampling. One rural and urban district was randomly selected within each focal area, and three FGDs were conducted in each. These sampling strategies helped to ensure that a range of diverse perspectives at national and community levels were captured.


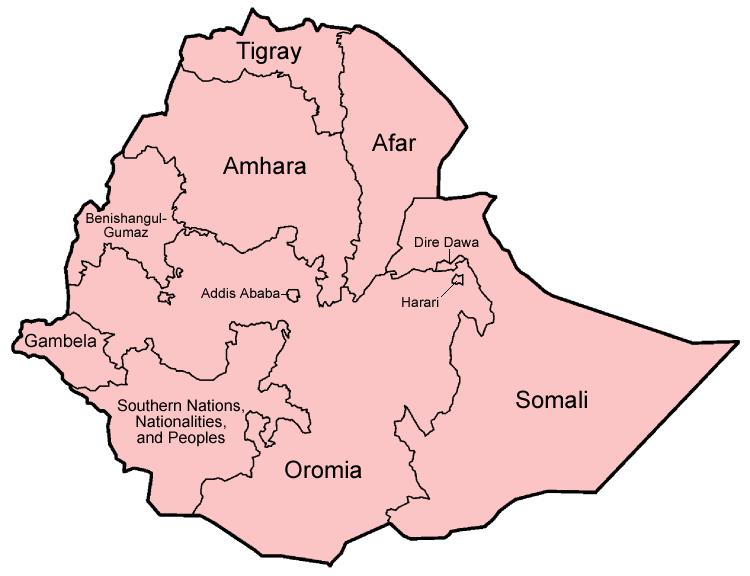


## **Supplementary Appendix Figure 3:** Regional map of Ethiopia displaying study sites for subnational key informant interviews and focus group discussions.

Research Methods

*In-Depth Interviews*

Firstly, 11 interviews were conducted with experts in Addis Ababa. These experts were officials from ministries directly or indirectly concerned by the issues of reducing malnutrition, including the Ministry of Health, Ministry of Education, Ministry of Agriculture, Ministry of Finance and Economic Development, experts from the specialized agencies of the United Nations, international NGOs and professors from Addis Ababa University. These individuals were purposively selected based on their expertise in nutrition, health and other sectors.

Secondly, 12 interviews were conducted in two districts of SNNP region (Bonga and Yeki) and two districts of Somali region (Harshin and Aware). These interviews targeted resource persons with several functions: teachers and health staff (health extension workers, maternal, newborn and child health care workers, district health surveillance focal persons, and senior health centre staff). Interviews were conducted in both urban and rural areas.

*Focus Group Discussions*

Thirdly, 12 focus groups discussions were organized, with three groups per district, across the two regions. In each region, three focus groups were held with mothers who gave birth between 1987-1991, three focus groups with mothers who gave birth between 2003-2007, and three focus groups with younger women who gave birth between 2011-2015.

*Data Analysis*

Data generated during focus group discussions, and semi-structured interviews were analyzed using the UNICEF Nutrition Framework [138], Lancet Nutrition framework [139], and the adapted framework for the country case studies (Figures 7 & 8). These conceptual frameworks guided the qualitative analysis and interpretation of key determinants and contextual factors, as well as facilitators and barriers to nutrition-specific and –sensitive events. The qualitative analysis explored distal/basic causes (e.g., GDP, education, political context), nutrition-sensitive and -specific programs, underlying causes (e.g., inadequate feeding practices, and food insecurity, inadequate care and health services and unhealthy environment), and proximal/immediate causes (e.g., maternal characteristics, inadequate dietary intake, disease, and child characteristics). Responses from national stakeholders, regional stakeholders and mothers at community-level were analyzed separately. Thematic analysis was conducted to explore key themes that emerged based on stunting determinants including socioeconomic status (e.g., living conditions), migration, hygiene and sanitation, and nutrition and eating behaviours.

All interviews were audio recorded and conducted in the local language via a total of seven trained interviews (three in SNNP and four in Somali). The interviews were then translated into English. National key informant and regional stakeholder interviews took between 1-3 hours and focus group discussion took between 2.5 and 3.5 hours.

# **Supplementary Appendix 5:** Quantitative Results

## **Supplementary Appendix Figure 4A:** Spline analysis of inflection points of change in the slope of HAZ, 2000


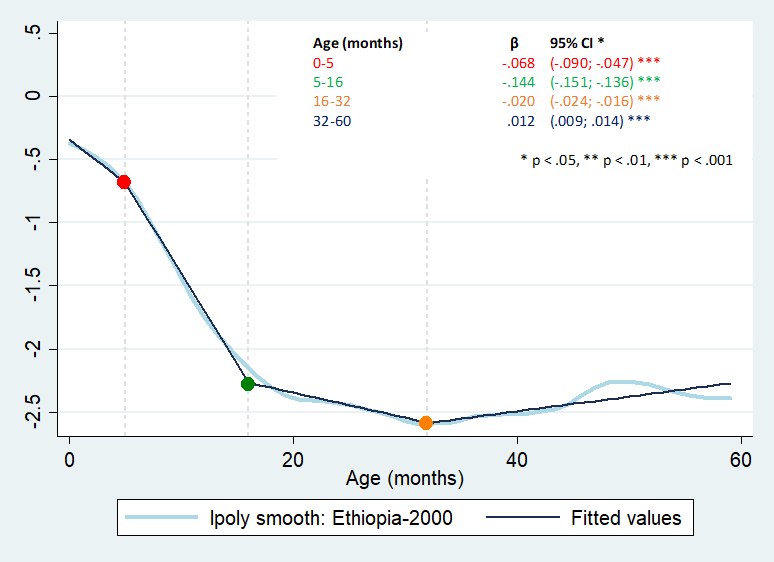


## **Supplementary Appendix Figure 4B:** Spline analysis of inflection points of change in the slope of HAZ, 2005


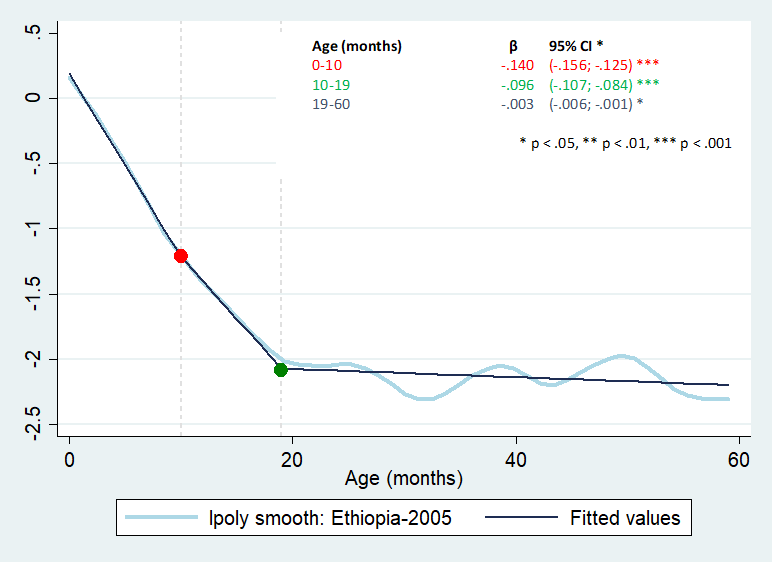


## **Supplementary Appendix Figure 4C:** Spline analysis of inflection points of change in the slope of HAZ, 2011


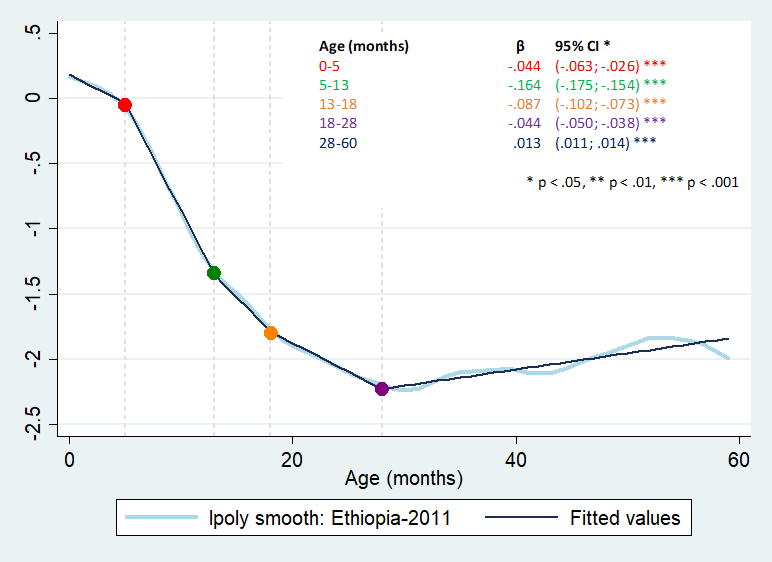


## **Supplementary Appendix Figure 4D:** Spline analysis of inflection points of change in the slope of HAZ, 2016


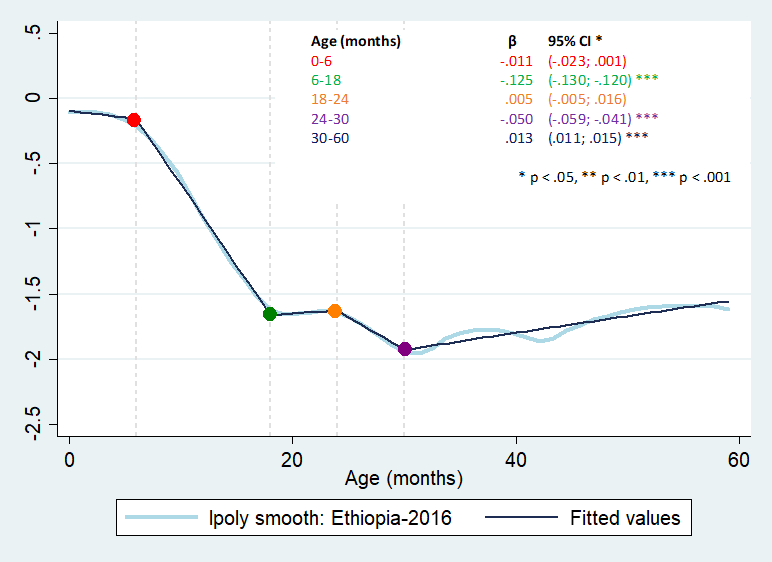


## **Supplementary Appendix Figure 5A:** 2000 stunting prevalence by region


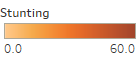

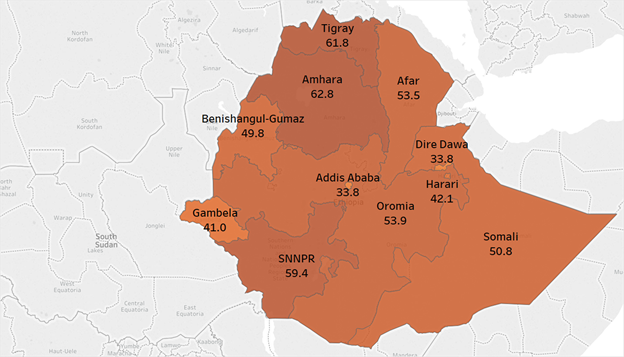


## **Supplementary Appendix Figure 5B:** 2005 stunting prevalence by region


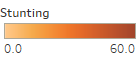

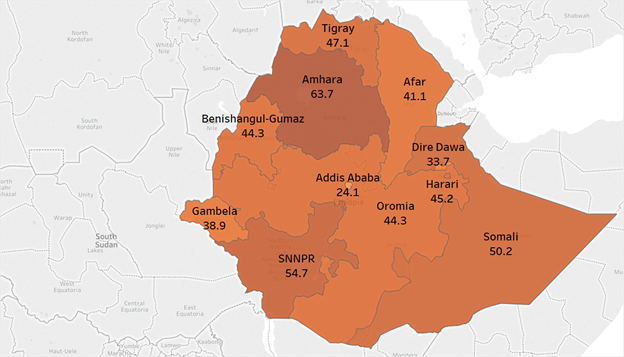


## **Supplementary Appendix Figure 5C:** 2011 stunting prevalence by region


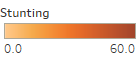

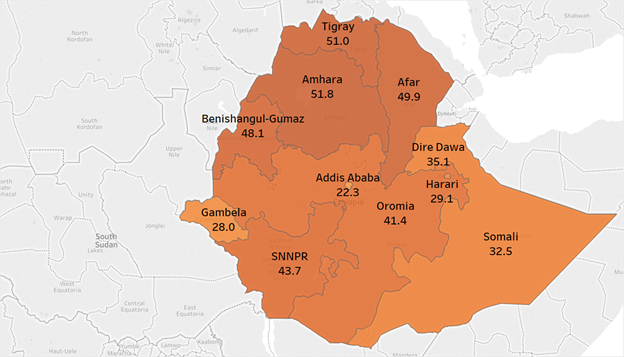


## **Supplementary Appendix Figure 6A:** Change in absolute SII by year in Ethiopia


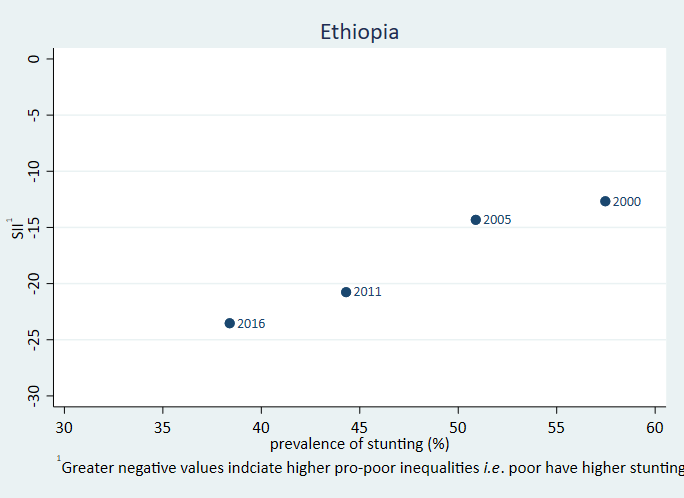


## **Supplementary Appendix Figure 6B:** Change in relative CIX by year in Ethiopia


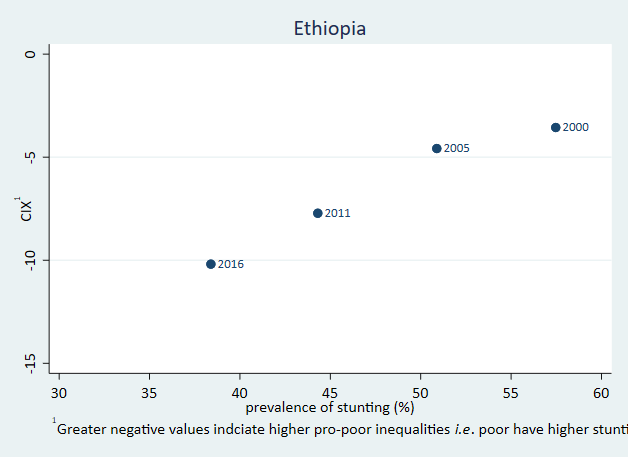


## **Supplementary Appendix Figure 7:** Stunting prevalence disaggregated by child sex, 2000-2016


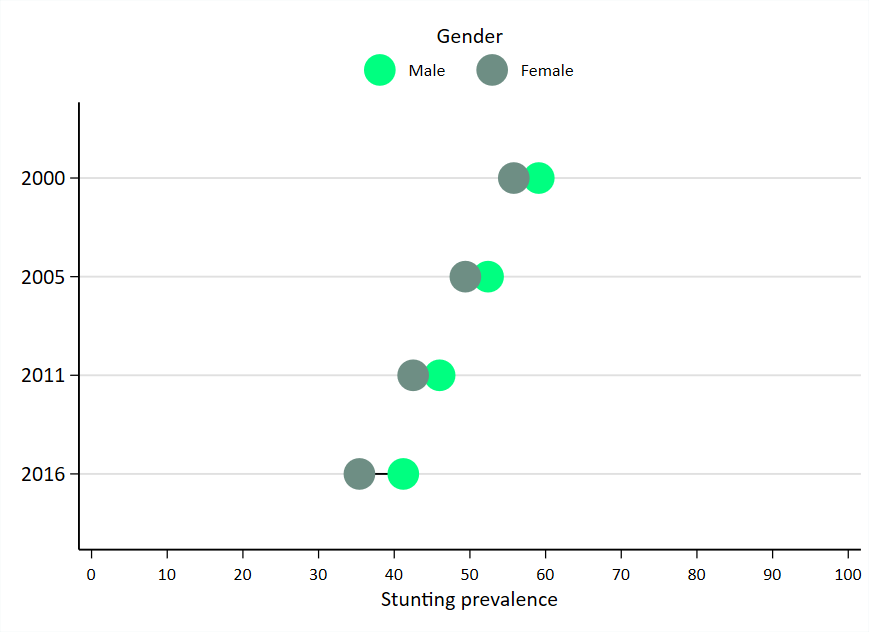


## **Supplementary Appendix Table 2:** Descriptive trends in stunting determinants in 2001-2016 in children <5 years

| **Domain/Indicator** |  |  |  | **DHS survey** |  |  |
| --- | --- | --- | --- | --- | --- | --- |
|  | **2000** | **2005** | **2011** | **2016** | **(2016 - 2000)** | |
|  | **(n = 5975)** | **(n = 2600)** | **(n = 6561)** | **(n = 6184)** | **Change** | **p-value** |
| **Outcome** | | | | | | |
| Height for age z-score | -2.14 | -1.79 | -1.57 | -1.35 | 0.79 | <0.001 |
|  |  |  |  |  |  |  |
| Stunting | 55.16 | 49.07 | 42.31 | 36.71 | -18.45 | <0.001 |
| % of children below -2sd |  |  |  |  |  |  |
|  |  |  |  |  |  |  |
| **Child Demographic** | | | | | | |
|  | | | | | | |
| Child sex (male) | 50.43 | 50.84 | 50.92 | 51.03 | 0.60 | 0.601 |
| % of males |  |  |  |  |  |  |
|  |  |  |  |  |  |  |
| Child age | 22.48 | 22.79 | 23.25 | 22.64 | 0.16 | 0.746 |
| (in months) |  |  |  |  |  |  |
|  |  |  |  |  |  |  |
| **Distal level** | | | | | | |
| **Basic causes & Income poverty** | | | | | | |
| Wealth Index (nine components using PCA) | 1.64 | 2.26 | 2.77 | 2.50 | 0.85 | <0.001 |
| (0 - 10) |  |  |  |  |  |  |
|  |  |  |  |  |  |  |
| Mother yea of education | 0.88 | 1.03 | 1.57 | 2.10 | 1.22 | <0.001 |
|  |  |  |  |  |  |  |
| Father year of education | 1.92 | 2.21 | 2.92 | 3.37 | 1.46 | <0.001 |
|  |  |  |  |  |  |  |
| **Intermediate level** | | | | | | |
| **Inadequate feeding practices and food insecurity** | | | | | | |
| Duration of breastfeeding | 18.58 | 18.35 | 16.10 | 15.41 | -3.17 | <0.001 |
| (in months) |  |  |  |  |  |  |
| Total yield | 11.15 | 11.83 | - | 18.09 | 6.94 | <0.001 |
|  |  |  |  |  |  |  |
| **Inadequate care and health services** | | | | | | |
| Live births attended by Skilled birth attendants | - | - | 12.03 | 30.26 | - | - |
| (% women) |  |  |  |  |  |  |
|  |  |  |  |  |  |  |
| Antenatal care | 10.73 | 13.22 | 19.47 | 32.07 | 21.34 | <0.001 |
| (% women with at least 4 visits) |  |  |  |  |  |  |
|  |  |  |  |  |  |  |
| Place of delivery at medical facility | 5.36 | 5.92 | 11.01 | 31.60 | 26.24 | <0.001 |
| (% women delivered at medical facility) |  |  |  |  |  |  |
|  |  |  |  |  |  |  |
| Vitamin A supplementation | 61.12 | 51.39 | 57.75 | 49.60 | -11.52 | <0.001 |
|  |  |  |  |  |  |  |
|  |  |  |  |  |  |  |
| Total number of health center | 0.17 | 0.69 | 2.22 | 2.20 | 2.03 | <0.001 |
| (per 10,000 population) |  |  |  |  |  |  |
|  |  |  |  |  |  |  |
| Total number of health workers | 1.29 | 1.92 | 4.29 | 7.37 | 6.08 | <0.001 |
| (per 10,000 population) |  |  |  |  |  |  |
|  |  |  |  |  |  |  |
| **Unhealthy household environment** | | | | | | |
| Urbanization | 11.00 | 8.92 | 14.34 | 12.50 | 1.50 | 0.492 |
| (% of urban population) |  |  |  |  |  |  |
|  |  |  |  |  |  |  |
| Open defecation | 85.52 | 65.80 | 42.34 | 35.18 | -50.34 | <0.001 |
| (% population) |  |  |  |  |  |  |
|  |  |  |  |  |  |  |
| Water source - piped | 13.40 | 17.65 | 27.48 | 28.26 | 14.87 | <0.001 |
| (% of population) |  |  |  |  |  |  |
|  |  |  |  |  |  |  |
| Number of household members | 5.90 | 6.01 | 5.85 | 5.83 | -0.07 | 0.319 |
|  |  |  |  |  |  |  |
|  |  |  |  |  |  |  |
| **Proximal level** | | | | | | |
| **Disease** | | | | | | |
| ARI infection | 37.48 | 21.08 | 22.48 | 22.31 | -15.17 | <0.001 |
| (% under-5 population within last 2 weeks) |  |  |  |  |  |  |
|  |  |  |  |  |  |  |
| Diarrhea infection | 29.00 | 22.92 | 16.42 | 14.59 | -14.42 | <0.001 |
| (% under-5 population within last 2 weeks) |  |  |  |  |  |  |
|  |  |  |  |  |  |  |
| **Child characteristics** | | | | | | |
| Low birthweight* | 7.51 | 14.65 | 8.36 | 12.87 | 5.36 | 0.042 |
| (%, index child) |  |  |  |  |  |  |
|  |  |  |  |  |  |  |
| **Maternal characteristics** | | | | | | |
| Age | 29.61 | 29.69 | 29.17 | 29.46 | -0.15 | 0.463 |
| (Mean, mothers 15-49) |  |  |  |  |  |  |
|  |  |  |  |  |  |  |
| Index births within last 5 years | 6.47 | 6.91 | 5.91 | 5.41 | -1.06 | 0.100 |
| (% mothers <18 years) |  |  |  |  |  |  |
|  |  |  |  |  |  |  |
| Index births within last 5 years | 17.92 | 17.75 | 14.12 | 15.12 | -2.80 | 0.005 |
| (% mothers >= 35 years) |  |  |  |  |  |  |
|  |  |  |  |  |  |  |
| Anemia during pregnancy | 0.00 | 26.97 | 17.54 | 28.08 | 0.00 | <0.001 |
| (% women 15-49 years) |  |  |  |  |  |  |
|  |  |  |  |  |  |  |
| BMI level | 20.05 | 20.33 | 20.38 | 20.67 | 0.62 | <0.001 |
| (Mean mothers 15-49 years) |  |  |  |  |  |  |
|  |  |  |  |  |  |  |
| Height | 156.47 | 157.05 | 156.55 | 156.98 | 0.51 | 0.009 |
| (Mean mothers 15-49 years) |  |  |  |  |  |  |
|  |  |  |  |  |  |  |
| Parity | 4.29 | 4.49 | 4.12 | 4.05 | -0.24 | 0.005 |
| (Total fertility rate) |  |  |  |  |  |  |
|  |  |  |  |  |  |  |
| Interpregnancy interval | 45.15 | 44.59 | 46.79 | 49.11 | 3.95 | <0.001 |
| (in months) |  |  |  |  |  |  |
|  |  |  |  |  |  |  |

## **Supplementary Appendix Table 3:** Decomposition analysis for children among 6-23 months from 2000-2016

| **Factors** | **Estimated coefficient** | **Mean difference (2016 - 2000)** | **Predicted change in HAZ** | **Share of predicted change in (%)** |
| --- | --- | --- | --- | --- |
| HAZ Score | - | 0.85 | 0.937 | 109.9% |
| Wealth index | 0.046 | 0.85 | 0.040 | 4.2% |
| Mother education | 0.033 | 1.31 | 0.044 | 4.7% |
| Father education | 0.038 | 1.52 | 0.057 | 6.1% |
| Duration of breastfeed (in months) | -0.073 | -0.40 | 0.029 | 3.1% |
| Complementary feeding | 0.210 | -0.01 | -0.002 | -0.2% |
| DPT vaccination | -0.195 | 0.33 | -0.063 | -6.8% |
| Antenatal care visits 4+ | 0.053 | 0.25 | 0.013 | 1.4% |
| Place of delivery | 0.155 | 0.32 | 0.049 | 5.2% |
| Health person per 10,000 pop | 0.034 | 6.11 | 0.207 | 22.1% |
| Total crop yields | 0.049 | 7.00 | 0.342 | 36.5% |
| Reduced open defecation | -0.281 | -0.50 | 0.141 | 15.1% |
| Use of dairy products | 0.190 | -0.01 | -0.001 | -0.1% |
| Use of fruits and vegetables | 0.358 | 0.00 | -0.001 | -0.1% |
| Early age pregnancy | -0.358 | -0.01 | 0.004 | 0.4% |
| Maternal BMI | 0.037 | 0.51 | 0.019 | 2.0% |
| Maternal height | 0.045 | 0.28 | 0.013 | 1.4% |
| Inter-pregnancy interval (in months) | 0.003 | 4.41 | 0.015 | 1.6% |
| Others | - | - | 0.033 | 3.5% |

## **Supplementary Appendix Figure 8:** Decomposing predicted changes in HAZ among children 6-23 months (i.e. relative ranking of product coefficients for determinant domains) from 2000-2016

## **Supplementary Appendix Table 4:** Decomposition analysis for children 24-59 months from 2000-2016

| **Factors** | **Estimated coefficient** | **Mean difference (2016 - 2000)** | **Predicted change in HAZ** | **Share of predicted change in (%)** |
| --- | --- | --- | --- | --- |
| HAZ Score | - | 0.80 | 0.67 | 84.3% |
| Wealth index | 0.05 | 0.93 | 0.05 | 7.1% |
| Mother education | 0.06 | 1.03 | 0.06 | 8.7% |
| Father education | 0.02 | 1.45 | 0.04 | 5.3% |
| Antenatal care visits 4+ | 0.17 | 0.18 | 0.03 | 4.4% |
| Total number of health workers | 0.05 | 2.02 | 0.10 | 14.8% |
| Total crop yield | 0.04 | 6.10 | 0.24 | 35.2% |
| Reduced open defecation | -0.20 | -0.51 | 0.10 | 14.9% |
| Diarrhea | -0.27 | -0.10 | 0.03 | 4.1% |
| Maternal age | 0.02 | -0.57 | -0.01 | -1.6% |
| Maternal BMI | 0.03 | 0.67 | 0.02 | 3.3% |
| Maternal height | 0.04 | 0.46 | 0.02 | 2.8% |
| Inter-pregnancy interval (in months) | 0.00 | 2.47 | 0.01 | 1.6% |
| Others | - | - | -0.01 | -0.8% |

## **Supplementary Appendix Figure 9:** Decomposing predicted changes in HAZ among children 24-59 months (i.e. relative ranking of product coefficients for determinant domains) from 2000-2016

## **Supplementary Appendix Table 5:** Decomposition analysis for under-5 children from 2000-2016

| **Factors** | **Estimated coefficient** | **Mean difference (2016 - 2000)** | **Predicted change in HAZ** | **Share of predicted change in (%)** |
| --- | --- | --- | --- | --- |
| HAZ Score | - | 0.79 | 0.953 | 120.3% |
| Wealth index | 0.049 | 0.85 | 0.042 | 4.4% |
| Mother education | 0.041 | 1.22 | 0.050 | 5.3% |
| Father education | 0.033 | 1.46 | 0.048 | 5.0% |
| Antenatal care visits 4+ | 0.078 | 0.21 | 0.017 | 1.8% |
| Number of health workers | 0.045 | 6.08 | 0.271 | 28.4% |
| Total crop yield | 0.044 | 6.94 | 0.306 | 32.1% |
| Reduced open defecation | -0.248 | -0.50 | 0.125 | 13.1% |
| Diarrhea | -0.257 | -0.14 | 0.037 | 3.9% |
| Maternal age | 0.014 | -0.15 | -0.002 | -0.2% |
| Maternal BMI | 0.048 | 0.62 | 0.030 | 3.1% |
| Maternal height | 0.043 | 0.51 | 0.022 | 2.3% |
| Inter-pregnancy interval (in months) | 0.004 | 3.95 | 0.015 | 1.6% |
| Others | - | - | -0.007 | -0.7% |

## **Supplementary Appendix Figure 10:** Decomposing predicted changes in HAZ among children under-5 (i.e. relative ranking of product coefficients for determinant domains) from 2000-2016

## **Supplementary Appendix Table 6:** Difference-in-differences multivariable regression for children under-5 years from 2000 – 2016

| **Domain/Indicator** | **Outcome = HAZ** | | | |
| --- | --- | --- | --- | --- |
|  | **(Height for age z-score for under-5 children)** | | | |
|  | **Period 2000 to 2016** | | | |
|  | **Bivariate regression coefficient** | | **Final multivariable regression coefficient*** | |
|  | b estimate (95% CI)  *p*-value | | b estimate (95% CI)  *p*-value | |
| **Distal level** | | | | |
| **Basic causes & Income poverty** | | | | |
| Wealth Index (nine components using PCA) | 0.062 (0.051; 0.072)    <0.001 | | 0.041 (0.029; 0.052)    <0.001 | |
| (0 - 10) |  |  |  |  |
|  |  |  |  |  |
| Wealth Index # Year | 0 (-0.002; 0.001)    0.53 | |  |  |
| (0 - 10) |  |  | -- | |
|  |  |  |  |  |
| Mother years of schooling | 0.074 (0.063; 0.084)    <0.001 | | 3.146 (-0.263; 6.556)    0.071 | |
|  |  |  |  |  |
|  |  |  |  |  |
| Maternal education # year | -0.002 (-0.003; 0)    0.042 | | -0.0016 (-0.0032; 0.0001)    0.073 | |
|  |  |  |  |  |
|  |  |  |  |  |
| Father years of schooling | 0.062 (0.053; 0.07)    <0.001 | | 0.028 (0.017; 0.039)    <0.001 | |
|  |  |  |  |  |
|  |  |  |  |  |
| Father education # Year | -0.0004 (-0.0017; 0.0009)    0.578 | | -- | |
|  |  |  |  |  |
|  |  |  |  |  |
| **% of residual variance explained by covariates** | | |  | **16.5%** |
| **Intermediate level** | | | | |
| **Inadequate care and health services** | | | | |
| Antenatal care | 0.329 (0.242; 0.417)    <0.001 | | 0.13 (0.038; 0.223)    0.006 | |
| (% women with at least 4 visits) |  |  |  |  |
|  |  |  |  |  |
| Antenatal care # Year | -0.013 (-0.026; 0.001)    0.061 | | -- | |
|  |  |  |  |  |
|  |  |  |  |  |
| Place of delivery | 0.509 (0.412; 0.606)    <0.001 | | 47.342 (12.863; 81.822)    0.007 | |
| (% of delivery at medical facility) |  |  |  |  |
|  |  |  |  |  |
| Place of delivery # year | -0.0329 (-0.0484; -0.0175)    <0.001 | | -0.023 (-0.041; -0.006)    0.007 | |
|  |  |  |  |  |
|  |  |  |  |  |
| total number of health care centers per 10,000 population | -0.052 (-0.124; 0.02)    0.159 | | -- | |
|  |  |  |  |  |
|  |  |  |  |  |
| total number of health care centers per 10,000 population # Year | -0.0067 (-0.0181; 0.0047)    0.247 | | -- | |
|  |  |  |  |  |
|  |  |  |  |  |
| total number of health workers per 10,000 population | 0.032 (0.02; 0.044)    <0.001 | | 5.03 (-0.326; 10.386)    0.066 | |
|  |  |  |  |  |
|  |  |  |  |  |
| total number of health workers per 10,000 population # Year | -0.0043 (-0.0071; -0.0016)    0.002 | | -0.0025 (-0.0051; 0.0002)    0.067 | |
|  |  |  |  |  |
|  |  |  |  |  |
| **Inadequate feeding practices and food security** | | | | |
| Total crop yield | 0.012 (0.001; 0.023)    0.026 | | 0.022 (0.012; 0.033)    <0.001 | |
|  |  |  |  |  |
|  |  |  |  |  |
| Total crop yield # Year | -0.0028 (-0.0051; -0.0005)    0.018 | | -- | |
|  |  |  |  |  |
| **Unhealthy household environment** | | | | |
| Urbanization | 0.4653 (0.3433; 0.5872)    <0.001 | | -- | |
| (% of urban population) |  |  |  |  |
|  |  |  |  |  |
| Urbanization # Year | -0.00162 (-0.02093; 0.01769)    0.87 | | -- | |
|  |  |  |  |  |
|  |  |  |  |  |
| Open defecation | -0.2967 (-0.3764; -0.2169)    <0.001 | | - | |
| (% population) |  |  |  |  |
|  |  |  |  |  |
| Open defecation # Year | 0.00771 (-0.00535; 0.02076)    0.247 | | -- | |
|  |  |  |  |  |
|  |  |  |  |  |
| Water source - piped | 0.286 (0.192; 0.38)    <0.001 | | -- | |
| (% of population) |  |  |  |  |
|  |  |  |  |  |
| Water source - piped # Year | -0.0091 (-0.025; 0.0068)    0.261 | | -- | |
|  |  |  |  |  |
|  |  |  |  |  |
| Household crowding | 0.02 (0.003; 0.036)    0.018 | | -- | |
|  |  |  |  |  |
|  |  |  |  |  |
| Household crowding # Year | 0.0003 (-0.0022; 0.0027)    0.833 | |  |  |
|  |  |  | -- | |
|  |  |  |  |  |
| **% of residual variance explained by covariates** | | |  | **17.4%** |
| **Proximal level** | | | | |
| **Disease** | | | | |
| Acute Respiratory incidence/ reports | -0.012 (-0.088; 0.065)    0.765 | | -- | |
| (% under-5 population within last 2 weeks) |  |  |  |  |
|  |  |  |  |  |
| Acute Respiratory incidence # Year | 0.002 (-0.01; 0.014)    0.742 | | -- | |
|  |  |  |  |  |
|  |  |  |  |  |
| Diarrhea incidence/reports | -0.1722 (-0.2532; -0.0911)    <0.001 | | -0.256 (-0.332; -0.179)    <0.001 | |
| (% under-5 population within last 2 weeks) |  |  |  |  |
|  |  |  |  |  |
| Diarrhea incidence # Year | 0.007 (-0.006; 0.02)    0.28 | | -- | |
|  |  |  |  |  |
|  |  |  |  |  |
| **Maternal characteristics** | | | | |
| Age | -0.016 (-0.021; -0.012)    <0.001 | | 0.012 (0.007; 0.016)    <0.001 | |
| (Mean, mothers 15-49) |  |  |  |  |
|  |  |  |  |  |
| Age# Year | 0.00022 (-0.00045; 0.0009)    0.517 | | -- | |
|  |  |  |  |  |
|  |  |  |  |  |
| Index births within last 5 years | -0.182 (-0.317; -0.047)    0.008 | | -- | |
| (% mothers <18 years) |  |  |  |  |
|  |  |  |  |  |
| Index birth within last 5 years # Year | -0.012 (-0.033; 0.008)    0.238 | | -- | |
|  |  |  |  |  |
|  |  |  |  |  |
| Index births within last 5 years | -0.052 (-0.137; 0.034)    0.235 | | -- | |
| (% mothers >= 35 years) |  |  |  |  |
|  |  |  |  |  |
| Index birth within last 5 years # Year | 0.003 (-0.009; 0.016)    0.605 | | -- | |
|  |  |  |  |  |
|  |  |  |  |  |
| BMI level | 0.054 (0.039; 0.068)    <0.001 | | 0.047 (0.034; 0.061)    <0.001 | |
| (Mean mothers 15-49 years) |  |  |  |  |
|  |  |  |  |  |
| BMI level # Year | 0.0001 (-0.0021; 0.0023)    0.935 | | -- | |
|  |  |  |  |  |
|  |  |  |  |  |
| Height | 0.046 (0.041; 0.051)    <0.001 | | 0.046 (0.041; 0.051)    <0.001 | |
| (Mean mothers 15-49 years) |  |  |  |  |
|  |  |  |  |  |
| Height # Year | 0.0005 (-0.0003; 0.0013)    0.24 | | -- | |
|  |  |  |  |  |
| Parity | -0.021 (-0.035; -0.008)    0.002 | | -- | |
| (Total fertility rate) |  |  |  |  |
|  |  |  |  |  |
| Parity # Year | 0.001 (-0.001; 0.003)    0.281 | | -- | |
|  |  |  |  |  |
|  |  |  |  |  |
| Interpregnancy interval | 0.005 (0.004; 0.007)    <0.001 | | 0.003 (0.002; 0.005)    <0.001 | |
| (in months) |  |  |  |  |
|  |  |  |  |  |
| Inter pregnancy intervals # Year | -0.0002 (-0.0004; 0.0001)    0.129 | | -- | |
|  |  |  |  |  |
|  |  |  |  |  |
| **% of residual variance explained by covariates** | | |  | **20.7%** |
| **Time** | | | | |
| **Time** | | | | |
| Year | 0.049 (0.042; 0.055)    <0.001 | | 0.027 (0.013; 0.04)    <0.001 | |
|  |  |  |  |  |
|  |  |  |  |  |
| * Adjusted for child age, sex, and province | |  |  |  |

## **Supplementary Appendix Table 7:** Difference-in-differences multivariable regression for children 24-59 months from 2000 – 2016

| **Domain/Indicator** | **Outcome = HAZ** | | | |
| --- | --- | --- | --- | --- |
|  | **(Height for age z-score for children 24 months and above)** | | | |
|  | **Period 2000 to 2016** | | | |
|  | **Bivariate regression coefficient** | | **Final multivariable regression coefficient*** | |
|  |  | b estimate (95% CI) |  | b estimate (95% CI) |
|  |  | *p*-value |  | *p*-value |
|  |  |  |  |  |
| **Distal level** | | | | |
| **Basic causes & Income poverty** | | | | |
| Wealth Index (nine components using PCA) |  | 0.073 (0.06; 0.085) |  | 0.041 (0.026; 0.056) |
| (0 - 10) |  | <0.001 |  | <0.001 |
|  |  |  |  |  |
| Wealth Index # Year |  | 0.0005 (-0.0015; 0.0025) |  |  |
| (0 - 10) |  | 0.62 |  | -- |
|  |  |  |  |  |
| Mother years of schooling |  | 0.085 (0.073; 0.096) |  | 0.043 (0.027; 0.059) |
|  |  | <0.001 |  | <0.001 |
|  |  |  |  |  |
| Maternal education # year |  | -0.002 (-0.004; 0) |  |  |
|  |  | 0.087 |  | -- |
|  |  |  |  |  |
| Father years of schooling |  | 0.064 (0.052; 0.075) |  | 0.0252 (0.01; 0.0404) |
|  |  | <0.001 |  | 0.001 |
|  |  |  |  |  |
| Father education # Year |  | -0.0007 (-0.0025; 0.001) |  |  |
|  |  | 0.417 |  | -- |
|  |  |  |  |  |
| **% of residual variance explained by covariates** | | |  | **7.5%** |
| **Intermediate level** | | | | |
| **Inadequate care and health services** | | | | |
| Antenatal care |  | 0.418 (0.316; 0.519) |  | 0.146 (0.031; 0.261) |
| (% women with at least 4 visits) |  | <0.001 |  | 0.013 |
|  |  |  |  |  |
| Antenatal care # Year |  | -0.021 (-0.037; -0.005) |  |  |
|  |  | 0.01 |  | -- |
|  |  |  |  |  |
| Place of delivery |  | 0.522 (0.399; 0.645) |  | 44.022 (1.567; 86.478) |
| (% of delivery at medical facility) |  | <0.001 |  | 0.042 |
|  |  |  |  |  |
| Place of delivery # year |  | -0.0336 (-0.0549; -0.0124) |  | -0.022 (-0.043; -0.001) |
|  |  | 0.002 |  | 0.042 |
|  |  |  |  |  |
| Total number of health care centers per 10,000 population |  | -0.175 (-0.253; -0.098) |  |  |
|  |  | <0.001 |  | -- |
|  |  |  |  |  |
| Total number of health care centers per 10,000 population # Year |  | -0.0014 (-0.0149; 0.0122) |  |  |
|  |  | 0.844 |  | -- |
|  |  |  |  |  |
| Total number of health workers per 10,000 population |  | 0.045 (0.031; 0.059) |  |  |
|  |  | <0.001 |  | -- |
|  |  |  |  |  |
| Total number of health workers per 10,000 population # Year |  | -0.0017 (-0.0047; 0.0013) |  |  |
|  |  | 0.269 |  | -- |
|  |  |  |  |  |
| **Inadequate feeding practices and food security** | | | | |
| Total number of yield |  | -0.022 (-0.032; -0.011) |  |  |
|  |  | <0.001 |  | -- |
|  |  |  |  |  |
| Total number of yield # Year |  | -0.0014 (-0.004; 0.0013) |  |  |
|  |  | 0.307 |  | -- |
|  |  |  |  |  |
| **Unhealthy household environment** | | | | |
| Urbanization |  | 0.637 (0.5; 0.774) |  |  |
| (% of urban population) |  | <0.001 |  | -- |
|  |  |  |  |  |
| Urbanization # Year |  | 0.004 (-0.019; 0.027) |  |  |
|  |  | 0.725 |  | -- |
|  |  |  |  |  |
| Open defecation |  | -0.315 (-0.412; -0.218) |  |  |
| (% population ) |  | <0.001 |  | - |
|  |  |  |  |  |
| Open defecation # Year |  | 0.00005 (-0.0175; 0.01759) |  |  |
|  |  | 0.996 |  | -- |
|  |  |  |  |  |
| Water source - piped |  | 0.434 (0.324; 0.544) |  | 0.13 (0.009; 0.251) |
| (% of population) |  | <0.001 |  | 0.035 |
|  |  |  |  |  |
| Water source - piped # Year |  | -0.008 (-0.027; 0.011) |  |  |
|  |  | 0.403 |  | -- |
|  |  |  |  |  |
| Household crowding |  | -0.0002 (-0.0217; 0.0214) |  |  |
|  |  | 0.987 |  | -- |
|  |  |  |  |  |
| Household crowding # Year |  | 0.0002 (-0.0031; 0.0035) |  |  |
|  |  | 0.894 |  | -- |
|  |  |  |  |  |
| **% of residual variance explained by covariates** | | |  | **8.6%** |
| **Proximal level** | | | | |
| **Disease** | | | | |
| Acute Respiratory incidence/ reports |  | -0.025 (-0.126; 0.075) |  |  |
| (% under-5 population within last 2 weeks) |  | 0.624 |  | -- |
|  |  |  |  |  |
| Acute Respiratory incidence # Year |  | 0.006 (-0.009; 0.021) |  |  |
|  |  | 0.426 |  | -- |
|  |  |  |  |  |
| Diarrhea incidence/reports |  | -0.302 (-0.4232; -0.1808) |  | -0.2842 (-0.4068; -0.1617) |
| (% under-5 population within last 2 weeks) |  | <0.001 |  | <0.001 |
|  |  |  |  |  |
| Diarrhea incidence # Year |  | 0.008 (-0.011; 0.027) |  |  |
|  |  | 0.426 |  | -- |
|  |  |  |  |  |
| **Maternal characteristics** | | | | |
| Age |  | 0.01 (0.004; 0.016) |  | 0.018 (0.011; 0.024) |
| (Mean, mothers 15-49) |  | 0.001 |  | <0.001 |
|  |  |  |  |  |
| Age# Year |  | 0.0006 (-0.0003; 0.00149) |  |  |
|  |  | 0.191 |  | -- |
|  |  |  |  |  |
| Index births within last 5 years |  | -0.052 (-0.216; 0.111) |  |  |
| (% mothers <18 years) |  | 0.531 |  | -- |
|  |  |  |  |  |
| Index birth within last 5 years # Year |  | -0.019 (-0.045; 0.007) |  |  |
|  |  | 0.155 |  | -- |
|  |  |  |  |  |
| Index births within last 5 years |  | 0.202 (0.091; 0.313) |  |  |
| (% mothers >= 35 years) |  | <0.001 |  | -- |
|  |  |  |  |  |
| Index birth within last 5 years # Year |  | 0.003 (-0.014; 0.02) |  |  |
|  |  | 0.696 |  | -- |
|  |  |  |  |  |
| BMI level |  | 0.045 (0.028; 0.061) |  | 0.035 (0.019; 0.05) |
| (Mean mothers 15-49 years) |  | <0.001 |  | <0.001 |
|  |  |  |  |  |
| BMI level # Year |  | 0.0013 (-0.0014; 0.0039) |  |  |
|  |  | 0.342 |  | -- |
|  |  |  |  |  |
| Height |  | 0.041 (0.034; 0.048) |  | 0.041 (0.034; 0.048) |
| (Mean mothers 15-49 years) |  | <0.001 |  | <0.001 |
|  |  |  |  |  |
| Height # Year |  | -0.0001 (-0.0012; 0.0009) |  |  |
|  |  | 0.795 |  | -- |
|  |  |  |  |  |
| Parity |  | -0.005 (-0.021; 0.011) |  |  |
| (Total fertility rate) |  | 0.504 |  | -- |
|  |  |  |  |  |
| Parity # Year |  | 0 (-0.002; 0.003) |  |  |
|  |  | 0.83 |  | -- |
|  |  |  |  |  |
| Interpregnancy interval |  | 0.006 (0.004; 0.008) |  | 0.005 (0.003; 0.007) |
| (in months) |  | <0.001 |  | <0.001 |
|  |  |  |  |  |
| Inter pregnancy intervals # Year |  | -0.0001 (-0.0004; 0.0002) |  |  |
|  |  | 0.466 |  | -- |
|  |  |  |  |  |
| **% of residual variance explained by covariates** | | |  | **12.6%** |
| **Time** | | | | |
| **Time** | | | | |
| Year |  | 0.048 (0.04; 0.056) |  | 0.05 (0.036; 0.065) |
|  |  | <0.001 |  | <0.001 |
|  |  |  |  |  |
| * Adjusted for child age, and sex |  |  |  |  |
|  |  |  |  |  |

## **Supplementary Appendix Table 8:** Difference-in-differences multivariable regression for children 6-23 months from 2000-2016

| **Domain/Indicator** | **Outcome = HAZ** | | | |
| --- | --- | --- | --- | --- |
|  | **(Height for age z-score among 6 to 23 months children)** | | | |
|  | **Period 2001 to 2016** | | | |
|  | **Bivariate regression coefficient** | | **Final multivariable regression coefficient*** | |
|  |  | b estimate (95% CI) |  | b estimate (95% CI) |
|  |  | *p*-value |  | *p*-value |
|  |  |  |  |  |
| **Distal level** | | | | |
| **Basic causes & Income poverty** | | | | |
| Wealth Index (six components using PCA) |  | 0.064 (0.048; 0.079) |  | 0.041 (0.024; 0.059) |
| (0 - 10) |  | <0.001 |  | <0.001 |
|  |  |  |  |  |
| Wealth Index # Year |  | 0.0005 (-0.0018; 0.0027) |  |  |
| (0 - 10) |  | 0.682 |  | -- |
|  |  |  |  |  |
| Mother years of schooling |  | 0.064 (0.05; 0.079) |  | 4.854 (0.453; 9.255) |
|  |  | <0.001 |  | 0.031 |
|  |  |  |  |  |
| Maternal education # year |  | -0.0028 (-0.0051; -0.0005) |  | -0.0024 (-0.0046; -0.0002) |
|  |  | 0.018 |  | 0.031 |
|  |  |  |  |  |
| Father years of schooling |  | 0.056 (0.043; 0.069) |  | 0.028 (0.012; 0.045) |
|  |  | <0.001 |  | 0.001 |
|  |  |  |  |  |
| Father education # Year |  | 0 (-0.001; 0.002) |  |  |
|  |  | 0.685 |  | -- |
|  |  |  |  |  |
| **% of residual variance explained by covariates** | | |  | **15.3%** |
| **Intermediate level** | | | | |
| **Inadequate feeding practices and food insecurity** | | | | |
| Duration of breastfeeding |  | -0.106 (-0.116; -0.095) |  | -0.07 (-0.116; -0.024) |
| (in months) |  | <0.001 |  | 0.003 |
|  |  |  |  |  |
| Duration of breastfeed # Year |  | -0.0011 (-0.0026; 0.0005) |  |  |
|  |  | 0.191 |  | -- |
|  |  |  |  |  |
| Complementary feeding |  | -0.249 (-0.373; -0.125) |  | 0.173 (0.03; 0.316) |
| (% children) |  | <0.001 |  | 0.017 |
|  |  |  |  |  |
| Complementary feeding # Year |  | 0.017 (-0.001; 0.035) |  |  |
|  |  | 0.071 |  | -- |
|  |  |  |  |  |
| **Inadequate care and health services** | | | | |
| DPT vaccine |  | -0.019 (-0.131; 0.092) |  |  |
| (% infants with 3 doses) |  | 0.737 |  | -- |
|  |  |  |  |  |
| DPT vaccine # Year |  | -0.009 (-0.025; 0.007) |  |  |
|  |  | 0.276 |  | -- |
|  |  |  |  |  |
| Measles vaccine |  | -0.266 (-0.377; -0.156) |  |  |
| (% infants) |  | <0.001 |  | -- |
|  |  |  |  |  |
| Measles vaccine # Year |  | -0.006 (-0.023; 0.011) |  |  |
|  |  | 0.486 |  | -- |
|  |  |  |  |  |
| Antenatal care |  | 0.333 (0.207; 0.459) |  | 0.155 (-0.009; 0.318) |
| (% women with at least 4 visits) |  | <0.001 |  | 0.064 |
|  |  |  |  |  |
| Antenatal care # Year |  | -0.015 (-0.033; 0.003) |  |  |
|  |  | 0.112 |  | -- |
|  |  |  |  |  |
| Place of delivery |  | 0.397 (0.262; 0.532) |  | 53.896 (-6.453; 114.244) |
| (% of delivery at medical facility) |  | <0.001 |  | 0.08 |
|  |  |  |  |  |
| Place of delivery # year |  | -0.047 (-0.07; -0.024) |  | -0.027 (-0.057; 0.003) |
|  |  | <0.001 |  | 0.081 |
|  |  |  |  |  |
| Vitamin A supplementation |  | -0.035 (-0.131; 0.061) |  |  |
| (% infants) |  | 0.48 |  | -- |
|  |  |  |  |  |
| Vitamin A supplementation # year |  | -0.009 (-0.023; 0.006) |  |  |
|  |  | 0.244 |  | -- |
|  |  |  |  |  |
| Total number of health care centers per 10,000 population |  | -0.019 (-0.119; 0.081) |  |  |
|  |  | 0.707 |  | -- |
|  |  |  |  |  |
| Total number of health care centers per 10,000 population # Year |  | -0.009 (-0.023; 0.006) |  |  |
|  |  | 0.251 |  | -- |
|  |  |  |  |  |
| Total number of health workers per 10,000 population |  | 0.029 (0.012; 0.045) |  | 0.04 (0.018; 0.062) |
|  |  | 0.001 |  | <0.001 |
|  |  |  |  |  |
| Total number of health workers per 10,000 population # Year |  | -0.005 (-0.008; -0.001) |  |  |
|  |  | 0.012 |  | -- |
|  |  |  |  |  |
| Total yield |  | 0.017 (0.003; 0.031) |  | 0.047 (0.029; 0.065) |
|  |  | 0.016 |  | <0.001 |
|  |  |  |  |  |
| Total yield # Year |  | -0.003 (-0.006; 0) |  |  |
|  |  | 0.025 |  | -- |
|  |  |  |  |  |
| **Unhealthy household environment** | | | | |
| Urbanization |  | 0.459 (0.302; 0.616) |  |  |
| (% of urban population) |  | <0.001 |  | -- |
|  |  |  |  |  |
| Urbanization # Year |  | -0.012 (-0.034; 0.01) |  |  |
|  |  | 0.285 |  | -- |
|  |  |  |  |  |
| Open defecation |  | -0.3371 (-0.4485; -0.2257) |  | -0.201 (-0.337; -0.065) |
| (% population ) |  | <0.001 |  | 0.004 |
|  |  |  |  |  |
| Open defecation # Year |  | 0.007 (-0.01; 0.024) |  |  |
|  |  | 0.414 |  | -- |
|  |  |  |  |  |
| Water source - piped |  | 0.2468 (0.1129; 0.3808) |  |  |
| (% of population) |  | <0.001 |  | -- |
|  |  |  |  |  |
| Water source - piped # Year |  | -0.017 (-0.037; 0.004) |  |  |
|  |  | 0.109 |  | -- |
|  |  |  |  |  |
| Household crowding |  | 0.012 (-0.011; 0.035) |  |  |
|  |  | 0.296 |  | -- |
|  |  |  |  |  |
| Household crowding # Year |  | 0.002 (-0.002; 0.005) |  |  |
|  |  | 0.352 |  | -- |
|  |  |  |  |  |
| **% of residual variance explained by covariates** | | |  | **18.9%** |
| **Proximal level** | | | | |
| **Disease** | | | | |
| Acute Respiratory incidence/ reports |  | -0.04 (-0.147; 0.067) |  |  |
| (% under-5 population within last 2 weeks) |  | 0.46 |  | -- |
|  |  |  |  |  |
| Acute Respiratory incidence # Year |  | 0.005 (-0.011; 0.02) |  |  |
|  |  | 0.537 |  | -- |
|  |  |  |  |  |
| Diarrhea incidence/reports |  | -0.113 (-0.223; -0.004) |  |  |
| (% under-5 population within last 2 weeks) |  | 0.042 |  | -- |
|  |  |  |  |  |
| Diarrhea incidence # Year |  | 0.004 (-0.013; 0.021) |  |  |
|  |  | 0.642 |  | -- |
|  |  |  |  |  |
| **Inadequate dietary intake** | | | | |
| Infant and young child minimum dietary diversity |  | 0.354 (0.174; 0.533) |  |  |
| (% children took at least 4 food gorups) |  | <0.001 |  | -- |
|  |  |  |  |  |
| Infant and young child minimum dietary diversity # Year |  | -0.002 (-0.027; 0.023) |  |  |
|  |  | 0.869 |  | -- |
|  |  |  |  |  |
| Grains, roots, and tubers |  | -0.249 (-0.377; -0.12) |  |  |
| (% children) |  | <0.001 |  | -- |
|  |  |  |  |  |
| Grains, roots, and tubers # Year |  | 0.01 (-0.009; 0.028) |  |  |
|  |  | 0.298 |  | -- |
|  |  |  |  |  |
| Legumes and nuts |  | -0.214 (-0.332; -0.095) |  |  |
| (% children) |  | <0.001 |  | -- |
|  |  |  |  |  |
| Legumes and nuts # Year |  | 0.002 (-0.015; 0.019) |  |  |
|  |  | 0.834 |  | -- |
|  |  |  |  |  |
| Dairy products |  | 0.349 (0.236; 0.461) |  | 0.133 (0.016; 0.251) |
| (% children) |  | <0.001 |  | 0.026 |
|  |  |  |  |  |
| Dairy products # Year |  | -0.011 (-0.027; 0.006) |  |  |
|  |  | 0.195 |  | -- |
|  |  |  |  |  |
| Flesh foods and Eggs |  | 0.205 (0.055; 0.356) |  | 0.128 (-0.011; 0.268) |
| (% children) |  | 0.008 |  | 0.071 |
|  |  |  |  |  |
| Flesh foods and Eggs # Year |  | -0.002 (-0.023; 0.02) |  |  |
|  |  | 0.884 |  | -- |
|  |  |  |  |  |
| Vitamin-A rich frutis and vegetables |  | 0.092 (-0.035; 0.219) |  |  |
| (% children) |  | 0.155 |  | -- |
|  |  |  |  |  |
| Vitamin-A rich frutis and vegetables # Year |  | 0.002 (-0.016; 0.02) |  |  |
|  |  | 0.846 |  | -- |
|  |  |  |  |  |
| Other fruits and vegetables |  | 0.371 (0.178; 0.565) |  | 0.36 (0.167; 0.553) |
| (% children) |  | <0.001 |  | <0.001 |
|  |  |  |  |  |
| Other fruits and vegetables # Year |  | -111.303 (-127.885; -94.721) |  |  |
|  |  | <0.001 |  | -- |
|  |  |  |  |  |
| **Maternal characteristics** | | | | |
| Age |  | -0.005 (-0.012; 0.003) |  |  |
| (Mean, mothers 15-49) |  | 0.212 |  | -- |
|  |  |  |  |  |
| Age# Year |  | -0.0002 (-0.0014; 0.0009) |  |  |
|  |  | 0.657 |  | -- |
|  |  |  |  |  |
| Index births within last 5 years |  | -0.312 (-0.508; -0.115) |  | -0.358 (-0.591; -0.125) |
| (% mothers <18 years) |  | 0.002 |  | 0.003 |
|  |  |  |  |  |
| Index birth within last 18 years # Year |  | 0.006 (-0.024; 0.035) |  |  |
|  |  | 0.705 |  | -- |
|  |  |  |  |  |
| Index births within last 5 years |  | -0.023 (-0.166; 0.12) |  |  |
| (% mothers >= 35 years) |  | 0.752 |  | -- |
|  |  |  |  |  |
| Index birth within last 5 years # Year |  | -0.007 (-0.029; 0.014) |  |  |
|  |  | 0.494 |  | -- |
|  |  |  |  |  |
| BMI level |  | 0.063 (0.043; 0.083) |  | 0.036 (0.013; 0.058) |
| (Mean mothers 15-49 years) |  | <0.001 |  | 0.002 |
|  |  |  |  |  |
| BMI level # Year |  | 0.0007 (-0.0023; 0.0038) |  |  |
|  |  | 0.631 |  | -- |
|  |  |  |  |  |
| Height |  | 0.054 (0.046; 0.062) |  | 0.051 (0.043; 0.059) |
| (Mean mothers 15-49 years) |  | <0.001 |  | <0.001 |
|  |  |  |  |  |
| Height # Year |  | 0 (-0.001; 0.002) |  |  |
|  |  | 0.695 |  | -- |
|  |  |  |  |  |
| Parity |  | -0.007 (-0.027; 0.013) |  |  |
| (Total fertility rate) |  | 0.511 |  | -- |
|  |  |  |  |  |
| Parity # Year |  | 0.001 (-0.002; 0.004) |  |  |
|  |  | 0.473 |  | -- |
|  |  |  |  |  |
| Interpregnancy interval |  | 0.004 (0.002; 0.006) |  | 0.003 (0.001; 0.005) |
| (in months) |  | <0.001 |  | 0.017 |
|  |  |  |  |  |
| Inter pregnancy intervals # Year |  | -0.0003 (-0.0006; 0.0001) |  |  |
|  |  | 0.122 |  | -- |
|  |  |  |  |  |
| **% of residual variance explained by covariates** | | |  | **23.2%** |
| **Time** | | | | |
| **Time** | | | | |
| Year |  | 0.053 (0.045; 0.061) |  | 0.011 (-0.007; 0.029) |
|  |  | <0.001 |  | 0.219 |
|  |  |  |  |  |
| * Adjusted for child age, sex, and province | | | | |

# **Supplementary Appendix 6:** Programs and Policies

## **Supplementary Appendix Table 9:** Detailed timeline of nutrition-specific and –sensitive laws, policies and programs in Ethiopia

| **ACTS/LAWS/REGULATIONS** | | |
| --- | --- | --- |
| 1. Regulations to Provide for the Establishment of the Ethiopian Health and Nutrition Research Institute (EHNRI) (1962- present) | Description | The Ethiopian Health and Nutrition Research Institute conducts research at the national level on the causes and spread of disease, as well as focusing on nutrition, traditional medicines, medical practices and modern drugs. It seeks to contribute to the advancement of health science and technology through data collection, research and study. Research duties of the Institute include research on the production of diagnostic, prophylactic and therapeutic substances; traditional drugs and practices; nutrition and food science and the prevention of diseases due to malnutrition. The Institute is given the power to study the cause, health impact and distribution of diseases as well as the production of traditional drugs. It was established with the duty to prepare a pharmacopeia of traditional drugs and enable traditional medicine practitioners to develop their profession through training in order to improve the quality of their services. Since its beginning, this institute has contributed to nutrition and public health interventions but has not significantly contributed to research activities [140,141]. |
|  | Importance | Likely important as a long-standing platform for gathering and sharing evidence for action focused on nutrition. |
|  | Theme(s) | Health |
|  |  | Nutrition |
| 1. Public Ownership of Rural Lands Proclamation No. 31, Sub-article 4: Distribution of land to the Tiller in Provinces with Privately Owned Rural Lands (1975) | Description | The Public Ownership of Rural Lands Proclamation No.31 was initiated in the wake of the ouster of Emperor Haile Selassie from power in 1974, following the failure of his government to adequately address pervasive socio-economic issues and the famine of 1973/1974. Proclamation No. 31 was announced in 1975 by the Provisional Military Administrative Council (PMAC), commonly referred to as the Derg. It posits that any individual willing to cultivate land is to be given this land so long as it does not exceed 10 hectares. The Proclamation was designed to promote equality and economic development by removing the feudal system and land tenure system from Ethiopia. However, the evaluation of its implementation found that it did not lead to significant, positive productivity or improvements in the standard of living [142,143]. |
|  | Importance | Very Important |
|  | Theme(s) | Agriculture |
|  |  | Food Security |
| 1. Federal Civil Servants Proclamation, Sub-Article 5: Maternity Leave (2007-present) | Description | The Federal Civil Servants Proclamation was created in 2007 and reformed in 2017. Based on the broader leave parameters of the 2017 reform, pregnant civil servants are entitled to paid leave for pre-natal medical examinations and paid maternity leave, in accordance with a physician’s recommendation. 30 days leave before birth and 90 days after delivery for a cumulative total of 120 days of paid leave are allowed. Paternity leave is also allowed for 10 days from the time of delivery [144,145]. |
|  | Importance | Likely not important for stunting decline |
|  | Theme(s) | Health |
| 1. Code of Marketing of Breast-milk Substitutes Act & Regulation (2009 – present) | Description | The Code of Marketing of Breast-milk Substitutes aims to protect and promote breastfeeding and ensure the proper use of breastmilk substitutes when necessary. It sets out national responsibilities of the infant food industry, health workers, national governments and concerned organizations. This code applies to all products marketed to replace breastmilk and encourages breastfeeding by restricting aggressive marketing used to sell breastmilk substitutes. The code bans advertising breastmilk substitutes to the public and to health care workers. Under this code, samples and gifts of breastmilk substitutes are not to be given to mothers or healthcare workers and information on artificial feeding products must be science-based and not idealized. The superiority of breastfeeding must also be highlighted in information about artificial feeding options. Unsuitable products such as sweetened condensed milk are not to be marketed for babies. A 2018 evaluation of the code found that it had few legal provisions, and that information gaps existed in several of the enforceable areas of the code, as well as the criteria of the monitoring mechanism [146,147]. |
|  | Importance | Not important to stunting decline |
|  | Theme(s) | Health |
|  |  | Nutrition |
| 1. Iodized salt (Production, Sale and Distribution) Act (2011-present) | Description | The Iodized Salt Act was established in 2011 and related to the importation, storage, transportation, distribution or sale of iodized salt for human consumption, in order to prevent and eradicate the public health effects of iodine deficiency in Ethiopia. Though iodization of salt began in the 1980s, progress was slow due to war and a lack of a clear enforcement strategy. This Act has helped to progressively increase the iodization of salt and included elements such as household visits, social promotion, committee creation and resource mobilization. Household iodized salt coverage increased from 15.4% in 2011 to 89% in 2016 [148–152]. |
|  | Importance | Likely not important |
|  | Theme(s) | Health |
|  |  | Nutrition |
| 1. Seqota Declaration (2015-2030) | Description | The Seqota Declaration aims to ensure universal access to nutritious food in the first 1000 days of a child’s life through the use of multi-sectoral nutrition-sensitive interventions around education, water, sanitation and social protection. Nutrition-specific interventions and the empowerment of women/girls is also supported through the Seqota Declaration. From 2016 to 2020, implementation is at a regional level around Tekeze River basin. National implementation is planned from 2020-2030. Key goals of the Declaration include: Zero stunting in children under-2 years old; 100% access to adequate food all year round; transformed smallholder productivity and income; zero post-harvest food loss; innovation in climate smart sustainable food systems; water, sanitation and hygiene; education and social protection. Multi-channel social and behavior change communication and community development will be utilized to achieve these goals [153]. |
|  | Importance | Promising recent initiative |
|  | Theme(s) | Health |
|  |  | Nutrition |
|  |  | WASH |
|  |  | Multi-sectoral Collaboration |

| **POLICIES/STRATEGIES/PLANS** | | | |
| --- | --- | --- | --- |
| 1. Health Policy of the Transition Government of Ethiopia (1993-present) | Description | The Health Policy of the Transition Government of Ethiopia was created following the end of the Derg government, who had struggled to enact their envisioned health policy. The transitional government’s health policy resulted from an assessment of the current state, extent and causes of existing and emerging health problems in the country. It focuses especially on women and children, the rural population, the poor, minorities, those affected by disaster, and those working at the forefront of economic productivity. Major components of this health policy include the decentralization and democratization of the health system; preventative and promotive components of health care; equitable standards of healthcare; promoting and strengthening inter-sectoral activities and maximally utilizing internal and external resources; strengthening international collaboration in relation to all activities contributing to health development; capacity development; capacity building; and promotion of participation of the private sector and NGOs. Priorities of this policy include information, education, support and communication related to a number of public health initiatives including: communicable diseases; malnutrition; mental health; occupational health; environmental health; and health management and infrastructure. This policy also aimed to conduct applied research and provide essential medicines, staff and equipment while concurrently acknowledging and giving attention to traditional medicine [154]. | |
|  | Importance | Very important | |
|  | Theme(s) | Health | |
|  |  | Nutrition | |
| 1. Agricultural Development Led Industrialization (ADLI) Strategy (1993-2002) | Description | The Agricultural Development Led Industrialization Strategy (ALDI) is seen as the overarching strategic framework guiding Ethiopia’s development. Its main objective focuses on strengthening ties between agriculture and industry by improving productivity among small-scale farmers and investing in industry in rural areas. The major motivation of this strategy is the presumption that strengthening of these sectors will motivate the economy in rural areas. Key components of this strategy focus on achieving rapid growth in agricultural production, raising incomes for rural families, achieving nation food self-sufficiency, and producing surpluses for sale within urban areas. This strategy utilizes a two-pronged approach which focuses on agricultural production and domestic manufacturing based on agro-processing. Agriculture and industry are expected to be expanded through this strategy for consumption domestically and via exports, while also reducing food insecurity. From this strategy came the Industrial Development Policy/Strategy (IDS), which was announced in 2002 [155,156]. |  |
|  | Importance | Very important |  |
|  | Theme(s) | Agriculture |  |
|  |  | Food Security |  |
|  |  | Poverty Reduction |  |
| 1. The Ethiopian Education and Training Policy (1994-present) | Description | The Ethiopian Education and Training Policy was launched to expand equitable access to relevant, high quality education across the country. The key objectives of this policy include providing equitable access to primary education and vocational training; restructuring the education system; changing the curriculum to increase the relevance to the community context; and overall quality improvement throughout the education system. The policy aims to improve the physical and mental potential of individuals and develop citizens who can utilize resources, respect human rights and equality and realize the full potential of all Ethiopians. This policy includes implementation strategies for formal and non-formal education, and led to the Education Sector Development Program, which began in 1997 [157]. |  |
|  | Importance | Very important |  |
|  | Theme(s) | Education |  |
| 1. Environmental Policy   (1997-present) | Description | Ethiopia’s Environmental Policy has the overall goal of improving and enhancing the health and quality of life of all Ethiopians, and promoting sustainable development through sound management and use of natural, human-made and cultural resources, as well as the overall environment. Focus is on sustaining biological diversity and renewable natural resources in order to improve the lives of the current generation while preserving the environment for future generations. The policy aims to develop, adapt and disseminate new technologies to develop under-utilized natural resources and manage the exploitation of non-renewable resources. Its guiding principles allow each community the right to a healthy environment and gives these communities the ability to make decisions to sustainably manage their resources. This also policy promotes social equity and the empowerment of women to be fully involved in decision-making and project design. It aims to integrate natural resource and environmental management across sectors with consideration and adjustment to full environmental cost pricing in the form of taxes, fees and incentives. While this policy is theoretically comprehensive, implementation has not resulted in significant environmental outcomes. This is likely due to a lack of resources, expertise and conflicting policies [158,159]. | |
|  | Importance | Likely important (as the country’s major policy focused on improving environment) but insufficient evidence | |
|  | Theme(s) | Health | |
| 1. Strategic Plan for Malaria Prevention and Control (2001- 2015) | Description | The Strategic Plan for Malaria Prevention and Control has the overall goal of reducing morbidity and mortality from malaria through expansion of the diagnosis, treatment, prevention, surveillance and control of the disease. The Plan focuses on scaling up malaria prevention and control activities alongside the Accelerated Expansion of Primary Health Care Coverage, with special attention paid to the capacity of Health Extension Workers (HEWs) in malaria prevention and control. Goals of this Strategic Plan include the widespread coverage of ACTs (artemisinin combination therapies), insecticide treated bed nets, indoor residual spraying and increased training across the health system. Activities focus on case management; selective vector control; epidemic prevention and control; information, education and communication (IEC) and behavioral impacts; and capacity building. This Plan is reformed and scaled up in 5 year intervals with increases in coverage goals at each reformation. The success of this plan has been supported by strong political commitment and robust malaria policies as well as strong financing from the government and outside donors. Challenges in the implementation of this plan have been associated with the absence of tracking and monitoring systems, high turnover of health staff, logistics, limited equipment and delays at the district level [160–165]. | |
|  | Importance | Likely important (since very effective program and contributed to reduced disease burden) but insufficient evidence to link program to stunting decline | |
|  | Theme(s) | Health | |
| 1. Ethiopian Water Sector Policy (2001- present) | Description | The Ethiopian Water Sector Policy aims to develop water resources in an equitable and sustainable way for the economic and social benefit of the country’s people. Among its goals is to mitigate disasters such as drought and flooding through efficient management of water resources. Objectives include the equitable, efficient and sustainable allocation of water and the conservation and protection of water resources. This policy identifies water as an economic and social good, commonly owned by the people of Ethiopia, each of who shall have adequate water of acceptable quality. This policy indicates that the development of water should be decentralized via multi-stakeholder participation, and especially the participation of women in water management. The policy is translated into action through the National Water Sector Strategy [166]. | |
|  | Importance | Likely not important to observed stunting decline given limited improvement in water sector and no major national programs linked to this policy | |
|  | Theme(s) | WASH | |
| 1. National Water Sector Strategy (2001-present) | Description | The National Water Sector Strategy provides an action plan for meeting the goals of the Ethiopian Water Sector Policy. This strategy implements guidelines for water supply and sanitation services, including livestock watering, and more broadly supports poverty reduction and sustainable human resources development. This strategy aims to improve living standards and socioeconomic wellbeing while improving water resources. Key objectives of this strategy include: improving people’s living standard and socio-economic well-being; allowing Ethiopians to realize food self-sufficiency and food security; extending water and sanitation coverage and improving environmental health; generating additional hydropower; utilizing water resources to achieve national development priorities; and promoting integrated water resource management [167]. | |
|  | Importance | Likely not important to observed stunting decline given limited improvement in water sector and no major national programs linked to this policy | |
|  | Theme(s) | WASH | |
|  |  | Health | |
|  |  | Food security | |
| 1. Industrial Development Policy/Strategy (IDS)   (2002) | Description | The Industrial Development Policy/Strategy (IDS) endeavors to work towards a broader goal of agriculture-led industrialization, export-led development and labor-intensive industries. It aims to ensure the competiveness of Ethiopian goods in international markets and as a result, export-oriented sectors are given preferential treatment. The government therefore plays a leading managerial role in this policy and in the implementation of domestic-foreign partnerships. IDS is linked to subsequent development plans within the country such as the Sustainable Development and Poverty Reduction Program (SDPRP) and the Plan of Action for Sustainable Development and Eradication of Poverty (PASDEP) [156,168,169]. |  |
|  | Importance | Very important |  |
|  | Theme(s) | Agriculture |  |
| 1. Sustainable Development and Poverty Reduction Program (SDPRP)   (2002-2005) | Description | The Sustainable Development and Poverty Reduction Program strives to reduce poverty and maintain macroeconomic stability for Ethiopia. This Program aimed to reduce poverty by 10% and increase the GDP by 7% by 2005. These economic improvements were expected to be achieved through: a focus on agriculture; strengthening private sector growth and development; increased exports, especially for high value agriculture products and skins, leather and textiles; investment in education to build capacity; decentralization of decision making to improve responsiveness; research on agriculture including water harvesting and small scale irrigation; and increased water resource utilization [170]. |  |
|  | Importance | Very Important |  |
|  | Theme(s) | Poverty Reduction |  |
|  |  | Food Security |  |
|  |  | Agriculture |  |
|  |  | WASH |  |
|  |  | Health |  |
|  |  | Nutrition |  |
|  |  | Education |  |
|  |  | Multi-sectoral Collaboration |  |
| 1. Rural Development Policy and Strategies (2002-present) | Description | The Rural Development Policy and Strategies works to minimization the need for foreign aid through the development of a market economy where rapid growth is assured and all Ethiopian people may benefit. To achieve this, a number of strategies are utilized including: providing basic direction to agricultural development; strengthening the agricultural labor force; proper use of land; preparing area compatible development packages; working towards market-led agriculture development; improving rural finance; promoting private sector participation in agricultural development; strengthening non-agricultural rural development activities; and managing rural development. This approach has experienced a number of challenges in its implementation including inadequate infrastructure, difficulty among farmers in acquiring new knowledge and technological backlog [156,168,169]. | |
|  | Importance | Very important | |
|  | Theme(s) | Agriculture | |
|  |  | Food Security | |
| 1. National Strategy for Infant and Young Child Feeding Practice (2004-present) | Description | The National Strategy for Infant and Young Child Feeding Practice is focused on standardizing infant and young child feeding in order to improve child health and clarify the roles of those responsible for promoting these feeding practices. This Strategy outlines targets based on child age. For children 0-6 months it promotes immediate initiation of breastfeeding; colostrum feeding; exclusive breastfeeding for the first six months; and adequate nutrition, support and care for lactating mothers. For children 6 to 24 months targets include: maintaining breastfeeding for up to two years; practicing responsive feeding; proper preparation and storage of complementary foods; adequate feeding based on a child’s age; appropriate food consistency, frequency and density; adequate nutrition provided by complementary foods; use of vitamin and mineral fortified products as needed; and continued feeding during illness. Targets are also specifically outlined for the feeding of infants and young children living with HIV/AIDs. Despite these recommendations, recent monitoring has shown that this strategy has not been fully executed, with front-line medical staff lacking updated guidelines [171,172]. | |
|  | Importance | Likely important but insufficient evidence regarding effectiveness and impact on child stunting | |
|  | Theme(s) | Nutrition | |
|  |  | Health | |
| 1. A Plan for Accelerated and Sustained Development to End Poverty (PASDEP) (2005-2010) | Description | A Plan for Accelerated and Sustained Development to End Poverty (PASDEP) aims to pave the way to achieve the Millennium Development Goals by 2015. It aims to build a foundation upon which Ethiopia is able to become a middle income country in the next 20-30 years through rapid and enduring economic development. This plan utilizes a number of strategies to provide direction in meeting its goals. Strategies include: managing the balance between economic and population growth; empowering women; accelerating growth and implementation capacity; managing risk; creating employment; and strengthen infrastructure and human resources. The scope of PASDEP is large—policies, strategies and programs drafted as part of PASDEP are associated with every industry including agriculture, healthcare, water and child nutrition. At implementation, base case and high growth scenarios were determined for GDP as well as agriculture, industry and services. Ethiopia was able to exceed the high growth scenario for GDP, agriculture and services but fell short of even the base case scenario for industry. Success was achieved through the use of small scale agriculture diversification, capacity building, investment in infrastructure and growth of non-agricultural production. Challenges throughout the process of achieving the Plan’s stated goals included climate challenges, low domestic saving, difficulty in collecting domestic revenue and high inflation [173,174]. |  |
|  | Importance | Very Important |  |
|  | Theme(s) | Poverty Reduction |  |
|  |  | Food Security |  |
|  |  | Agriculture |  |
|  |  | WASH |  |
|  |  | Health |  |
|  |  | Nutrition |  |
|  |  | Education |  |
|  |  | Multi-sectoral Collaboration |  |
| 1. National Strategy for Child Survival in Ethiopia (2005-2020) | Description | The National Strategy for Child Survival in Ethiopia aims to achieve universal high quality health coverage for mothers and newborns in communities and health facilities. It also aims to utilize community empowerment for interventions and reduce neonatal and child mortality rates nationally. Within the community, this strategy utilizes Health Extension Workers (HEWs) to identify and refer sick children to appropriate care; encourage breastfeeding; support healthy newborn care; and promote contraception for birth spacing. Facilities manage cases referred by HEWs and provide treatment, disease testing and advice for feeding. Mass media communication via radio is utilized to support these activities. Interventions under this strategy specifically target maternal and neonatal care, nutrition and disease control in an effort to reduce the under-five mortality rate to less than half of the 2013 rate by 2020. Guiding principles of this strategy include: equity and accessibility; community engagement; integration across the health system; evidence-based innovation; provision of quality service; responsiveness; evidence-based decision-making; efficient resource use; and strong monitoring and communication of best practices. The success of this strategy is a result of strong commitment, collaboration and funding across sectors, decentralization of health services, increased opportunity for women and girls and the implementation of related programs and policies. Barriers to this program include limited coverage, human resources, and lack of a robust health information system. Staff were also limited by lack of advanced training, lack of motivation and staff shortages [175,176]. | |
|  | Importance | Very important | |
|  | Theme(s) | Nutrition | |
|  |  | Health | |
|  |  | Multi-sectoral Collaboration | |
| 1. National Health Promotion and Communication Strategy (NHPCS) (2005-2020) | Description | The National Health Promotion and Communication Strategy aims to coordinate health communication and promotion within the country. This strategy guides health education and communication in order to improve knowledge and attitudes using evidence-based best practices. It aims to reduce barriers to behavior change and improve social determinants of health through strong multi-sectorial communication. It utilizes a range of communication strategies and technologies to improve health communication, including the use of mass and social media as well as dissemination of information through community groups. This strategy seeks to empower communities by improving capacity at the community level through the use of standardized guidelines as well as monitoring and evaluation [177]. | |
|  | Importance | Likely important | |
|  | Theme(s) | Health | |
|  |  | Multi-sectoral Collaboration | |
| 1. National Hygiene and Sanitation Strategy (2005-present) | Description | The National Hygiene and Sanitation Strategy aims to reduce fecal contamination and the spread of waterborne diseases. Its main goal is to prevent feces from entering the living environment and disrupting the fecal-oral route of disease. To achieve this, the strategy puts responsibility of hygiene improvement at the household-level with additional resources provided by community-level health workers. Three pillars represent the foundation of this strategy—Pillar 1 involves the creation of enabling environments through capacity creation, evaluation and supporting finance and policy; Pillar 2 includes the promotion of hygiene and sanitation through communication, community participation and social marketing; and Pillar 3 centers on the improvement of access to technology to facilitate waste management and water supply capabilities. The National Hygiene and Sanitation Strategy falls under the Health Sector Development Program, which focuses on public health interventions that are expected to have significant impact and reach [178]. | |
|  | Importance | Very important | |
|  | Theme(s) | WASH | |
|  |  | Health | |
| 1. National Nutritional Strategy (NNS) (2008-present) | Description | The National Nutritional Strategy has an overarching goal of ensuring the best nutrition possible for all Ethiopians. This strategy provides particular focus to the nutrition of vulnerable populations and aims to protect all citizens from malnutrition-related health problems, including unhealthy dietary patterns and lifestyles. Nutrition coordination across sectors working towards improvements in nutrition is an additional goal. The use of health facilities at the community level to provide education, growth monitoring, and care improvement, as well as fortification based interventions (i.e. Vitamin A supplementation and salt iodization) are key components of this strategy [152,179]. | |
|  | Importance | Likely important but late introduction so may not have impact on national stunting change | |
|  | Theme(s) | Nutrition | |
|  |  | Health | |
|  |  | Multi-sectoral Collaboration | |
| 1. Growth and Transformation Plan (GTP) (2010-2020) | Description | The Growth and Transformation Plan (GTP) has the overarching goals of improving economic growth and ending poverty. Its objectives include maintenance of 11% GDP growth, improvements in the quality of education and health, and engaging in sustainable state building and macroeconomic frameworks. Pillars of this plan include quicker and more equitable economic growth with a focus on agriculture; developing an environment that is friendly to the growth of industry; expanding infrastructure and social development quality; empowering women and youth; and ensuring good governance. Monitoring of this program is done through the use of a census, administrative and survey data currently utilized by the Welfare Monitoring System Program [180,181]. |  |
|  | Importance | Very Important |  |
|  | Theme(s) | Poverty Reduction |  |
|  |  | Food Security |  |
|  |  | Agriculture |  |
|  |  | Multi-sectoral Collaboration |  |
| 1. Agriculture Sector Policy and Investment Framework (2010-2020) | Description | The Agriculture Sector Policy and Investment Framework utilizes policy instruments in an aim to increase agricultural production and food security, resulting in the achievement of middle-income status for Ethiopia by 2020. Its objectives include improved productivity in agriculture and natural resources, improved commercialization and industrialization of agriculture, and universal food security. This initiative utilizes a number of key components to reach its objectives. For example, to improve production, it aims to increase the capacity of the least productive farmers in the country and move farmers to semi-commercial farming in order to improve food security. The management of natural resources will also be done in a sustainable way to reduce degradation [182]. | |
|  | Importance | Promising recent initiative | |
|  | Theme(s) | Agriculture | |
|  |  | Food Security | |
| 1. National Hygiene and Sanitation Strategic Action Plan (2011-2015) | Description | The National Hygiene and Sanitation Strategic Action Plan aims to achieve universal basic sanitation in Ethiopia by 2015. It includes multiple objectives to improve sanitation, hand washing, and reduce open defecation. It is intended to empower communities and increase access to facilities and services while improving emergency preparedness, regulation and human capacity for the implementation of good sanitation practices. This plan implements school and community-based interventions to educate and provide appropriate resources and hardware (e.g. improved latrines) to empowered communities [183]. | |
|  | Importance | Promising recent initiative | |
|  | Theme(s) | WASH | |
|  |  | Health | |
|  |  | Education | |
| 1. Climate Resilient Green Economy (CRGE) Strategy (2013-present) | Description | The Climate Resilient Green Economy Strategy aims to increase the per capita GDP to $1000 USD and achieve middle-income status by the year 2025, and prevent an increase in greenhouse gas emissions above the levels seen in 2013. This strategy consists of both a plan for a green economy and a plan for climate resilience. It also has specific goals around improving food security, production and income while reducing emissions, leapfrogging to energy efficient technology and the use of renewable sources of energy. Additionally, it aims to protect and re-establish forests. Specific initiatives have been selected to fast track these goals, including: exploiting Ethiopia’s vast hydro-power potential; large scale promotion of advanced rural cooking technology; efficient improvements in the livestock value chain and REDD+ [156,184]. |  |
|  | Importance | Promising recent initiative |  |
|  | Theme(s) | Agriculture |  |
|  |  | Food Security |  |
| 1. Integrated Urban Sanitation and Hygiene Strategy (2015-present) | Description | The Integrated Urban Sanitation and Hygiene Strategy was created to reduce the impact of poor sanitation on health by implementing sanitation systems, encouraging behavioral change, and strengthening regulatory and institutional capacities. Key elements include the construction of latrines at the community level as well as providing adequate sanitation in schools. These interventions are supported by communication encouraging behavior change through mobile SMS messaging and print media. Goals of this strategy include the elimination of open defecation by 2020, universal access to latrines and toilets, significant goals for waste management and healthcare waste and strengthening the capacity of the sector as a whole through the creation of a coordination body and the creation of a monitoring system and sanitation database [185]. | |
|  | Importance | Promising Recent initiative | |
|  | Theme(s) | WASH | |
|  |  | Health | |
| 1. National Health Care Quality Strategy (2016-2020) | Description | The National Health Care Quality Strategy aims to increase access to health care for all Ethiopians, as well as improve clinical care and patient safety. It utilizes interventions within facilities and within communities to improve quality of care and create awareness among patients and health care workers of best practice strategies. It aims to improve health care quality across a number of domains including effectiveness, patient safety, patient centeredness, access and equity. This strategy focused on five key areas which were expected to result in the greatest improvement to the health system: maternal and child health; nutrition; communicable diseases; chronic diseases; and clinical and surgical services [186]. | |
|  | Importance | Promising recent initiative | |
|  | Theme(s) | Health | |
|  |  | Nutrition | |
| 1. EU Joint Strategy on Nutrition in Ethiopia (2016-2020) | Description | The EU Joint Strategy on Nutrition in Ethiopia aims to contribute to the National Nutrition Program and the Seqota Declaration. This Joint Strategy has objectives focused on achieving a common understanding of development challenges, filling gaps, determining guiding principles with EU partners, improving quality of policy discussions, making current financing more effective and enhancing the leverage of EU support. Because of the European Commission’s commitment to reduce stunting in 7 million children globally by 2025, nutrition has been chosen as the theme through which to collaborate with member states. Notable components of this strategy include increasing capacity; designing common monitoring and evaluation systems; empowering women; addressing foodborne and waterborne diseases; internal cooperation; and community focused approaches [187]. | |
|  | Importance | Promising recent initiative | |
|  | Theme(s) | Nutrition | |
|  |  | Health | |
| 1. National Hygiene and Environmental Health Strategy (2016-2020) | Description | The National Hygiene and Environmental Health Strategy was designed to support the Health Sector Transformation Plan and is implemented with a vision to prevent disease and promote health, safety and wellbeing. The objectives of this strategy include improving access to adequate, safe and equitable sanitation and water; improving food safety; control of communicable and vector borne diseases; community empowerment; and reduction in pollution exposure. Major components include sanitation, personal hygiene, water quality, food hygiene, housing and institutional health, vector control, pollution and occupational health and safety [188]. | |
|  | Importance | Promising recent initiative | |
|  | Theme(s) | WASH | |
|  |  | Health | |
| 1. Nutrition Sensitive Agriculture Strategy (2016-2021) | Description | The Nutrition Sensitive Agriculture Strategy was created to add value to the National Nutrition Program and Seqota Declaration, and to ensure that Ethiopia’s food system has positive impacts on nutrition outcomes, with a particular focus on women and children. This strategy has a number of major objectives including leveraging nutrition into agriculture policies; strengthening organizational structures and capacity; improving year-round availability, access and consumption of nutritious foods; community resilience; empowerment of women and gender equality; and the establishment and strengthening of multi-sectoral coordination. It works towards strengthening multi-sectorial coordination between sectors and development partners [189]. | |
|  | Importance | Recent promising initiative | |
|  | Theme(s) | Agriculture | |
|  |  | Health | |
|  |  | Nutrition | |
|  |  | Multi-Sectoral Collaboration | |

| **PROGRAMS/PROJECTS** | | |
| --- | --- | --- |
| 1. Expanded Program on Immunization (EPI) (1980-present) | Description | The Expanded Program on Immunization (EPI) aims to accomplish 90% coverage nationally with all vaccines by 2020. This program utilizes community interventions to reach individuals through health facilities and through mobile units to provide access to immunization for those living at increased distance from health facilities. This program has existed since the early 1980s and initially had limited success due to high turnover of staff, poor infrastructure, limited trained personnel and lack of donor funding. Implementation of Reaching Every District (RED) and Sustainable Outreach Services (SOS) since the early 2000s has focused on providing coverage in hard to reach and below-target areas. Women of reproductive age and children under one year of age are current targets of the program. Priorities include improvement of the cold chain for vaccine transportation and storage, and introduction of a broader range of vaccines. Objectives for 2016 – 2020 include: increasing and maintaining vaccine coverage; maintaining polio free status and achieving recommended Acute Flaccid Paralysis (AFP) surveillance; eliminating measles and promoting the elimination of rubella and congenital rubella syndrome; attaining and maintaining elimination/control of vaccine preventable diseases; expanding cold chain capacity; improving healthcare worker knowledge; strengthening monitoring and evaluation; and  increase government financing for traditional and new vaccines [152,190,191]. |
|  | Importance | Very important |
|  | Theme(s) | Health |
| 1. Basic Education, Technical and Vocational Training Project (1992-1999) | Description | The Basic Education, Technical and Vocational Training Project was created in 1992 as part of the Emergency Recover and Reconstruction Program (ERRP). It focuses on improving the quality of education and cultivating specialized training. The program aims to close the gaps that exist in the quality of education and institutional availability between regions, while creating a significant number of new spaces across all levels of the education system. The initial project was due to be completed in 1996, but was extended to 1999 in order to provide more time to meet targets. Upon completion, it was relatively successful with 96% of school construction complete. Radio receivers were distributed to primary schools to provide access to educational programming and training was provided to educational media personnel [192]. |
|  | Importance | Very important |
|  | Theme(s) | Education |
| 1. Health Sector Development Program (HSDP) (1997-2015) | Description | The Health Sector Development Program has the overall goal of providing comprehensive, integrated, and cost-effective primary care, with a special focus on communicable disease prevention and control, nutrition, environmental health and hygiene, reproductive health and immunization. Major objectives of this program include: the reduction of maternal and under-five mortality; reduction of the total fertility rate; reduction of the incidence of and mortality from communicable and vector-borne diseases; and increased coverage of the Health Extension Program (HEP). Notable achievements of this program include: an increased number of health centers, posts and hospitals; increased number of health care workers and Health Extension Workers (HEWs) and frontline worker training; improved contraceptive coverage and maternal care; improvements to infectious and communicable diseases, including improvements to bed net distribution; and improvements in the number of children receiving Vitamin A supplementation and community nutrition coverage [193,194]. |
|  | Importance | Very important |
|  | Theme(s) | Health |
| 1. Education Sector Development Program (ESDP) (1997-present) | Description | The Education Sector Development Program (ESDP) derives its goals from the Ethiopian Education and Training Policy. Specific objectives of the Education Sector Development Program include increased access to and participation in education and training, ensuring equity; ensuring quality and relevance of education and training; lower education inefficiencies; prevention of HIV/AIDS and increased stakeholder participation. The major goals of ESDP include the production of good citizens, achieving universal primary education, meeting the needs of the workforce and building capacity within the education system. While early iterations of this program focused mainly on primary education, more recent amendments have expanded its reach to adult and non-formal education. Evaluations indicate that this program has been quite successful since its inception in 1997. While some regional variations continue to exist, the number of primary schools has nearly tripled and primary-level enrollment has grown from 3 to 18 million. These improvements have been focused at the primary level with work yet to be done at higher levels of education. Some of the challenges associated with this program include management challenges, regional and gender disparities and challenges related to community participation [195–197]. |
|  | Importance | Very important |
|  | Theme(s) | Education |
|  |  | Health |
|  |  | WASH |
|  |  | Nutrition |
| 1. Essential Nutrition Action (ENA) (1997-present) | Description | Essential Nutrition Action (ENA) is part of a wider program implemented by USAID across Asia and Africa. It aims to consolidate seven areas of nutrition behaviors that will provide the greatest reduction in maternal and child morbidity and mortality. This program is delivered both in communities and health facilities. Education and counselling are provided on best practice feeding for the prevention of malnutrition in children, and care associated with the prevention of malnutrition and improved maternal health are provided within health facilities. ENA seeks to improve health services and encourage behavior change in the first 1000 days of life through the use of interventions at 6 key contact points with the health system: during pregnancy; during delivery; postnatal and during family planning; during immunization; during well child and growth monitoring appointments, and during the care of a sick child. It recognizes the importance of sector integration to provide additional coverage, and leverages the role of agriculture, schools, sanitation and community nutrition in improving maternal and child health [171,198,199]. |
|  | Importance | Likely important as a preventive nutrition framework adopted by country |
|  | Theme(s) | Health |
|  |  | Nutrition |
|  |  | Agriculture |
|  |  | WASH |
|  |  | Multi-sectoral Collaboration |
| 1. Health Extension Program (HEP) (2003-present) | Description | The Health Extension Program (HEP) is a collaboration between the Ministry of Health and Ministry of Education which aims to improve equitable access to health care in rural areas, despite limited resources. In order to do this, it is focused on shifting health care resources from urban areas to rural areas where are larger proportion of the population resides. HEP utilizes high impact health services and focuses on four major components: family health; disease prevention and control; hygiene and environmental sanitation; and health education and communication. Decentralization and the provision of health coverage at the community and household-level, along with improving community knowledge are additional objectives. This program also aims to empower women and reduce maternal and child mortality while promoting an overall healthy lifestyle, with multiple new components being added addressing these elements at the community-level. Interventions are delivered within the community, health facilities and in schools. Healthy WASH practices, bed net usage and feeding are communicated by Health Extension Workers (HEWs) during home visits. Schools engage in deworming and health education. Within facilities, maternal and child care as well as nutritional and family planning advice is provided. Outreach and training for health workers and improvement in the quality of health services at the local level has also been implemented as part of this program. Evaluation of HEP has shown it has led to improvements in the health of participating communities. Use of latrines, family planning methods, maternal care, infectious diseases and immunization have also all been positively impacted by the HEP. Despite this, challenges remain including high turnover and poor salary of HEWs, poor referral system and gaps in equipment and pharmaceuticals [200–204]. |
|  | Importance | Very important |
|  | Theme(s) | Health |
|  |  | Nutrition |
|  |  | WASH |
|  |  | Multi-sectoral Collaboration |
| 1. Ethiopia Strategy Support Program (ESSP) (2004-present) | Description | The Ethiopia Strategy Support Program (ESSP) aims to reduce poverty through sustainable development and improving the policymaking process and capacity in Ethiopia. Objectives of the program aim to fill knowledge gaps and create a more integrated knowledge support system to solve complex issues associated with developing a pro-poor rural development strategy. Through this program, food policy analysis and rural policy development is supported using the Rural Economy Knowledge Support System (REKSS). Major activities of this program include engaging in collaborative research, knowledge management, knowledge dissemination and capacity building, and improving communication and institutional collaboration [205,206]. |
|  | Importance | Not sufficient evidence to conclude any impact on stunting reduction |
|  | Theme(s) | Nutrition |
|  |  | Agriculture |
|  |  | Poverty Reduction |
| 1. Enhanced Outreach Strategy and Targeted Supplementary Feeding Program (TSF) for Child Survival (2004-present) | Description | The Enhanced Outreach Strategy and Targeted Supplementary Feeding (TSF) Program for Child Survival is a biannual campaign to target immediate and underlying causes of malnutrition by providing mothers and children with preventative health care. This program utilizes a range of delivery platforms to improve immunization; prevent and control communicable and vector-borne diseases; identify malnutrition and provide deworming and vitamin A supplementation. Technology is utilized to provide education at the community-level directly to the targeted population. After 2012, the Community Health Days Program, part of the Health Extension Program (HEP), was implemented to replace the Enhanced Outreach Strategy. Community Health Days (CHDs) are implemented in four regions to provide proven health interventions to women and children. These include immunization, Vitamin A supplementation, deworming, and care for malnutrition. CHDs are held quarterly to reach women and children in communities and improve the coverage of the aforementioned health interventions [207,208]. |
|  | Importance | Very important |
|  | Theme(s) | Health |
|  |  | Nutrition |
| 1. Productive Safety Net Program Adaptable Program Loan (PSNP APL) (2005-2020) | Description | The Productive Safety Net Program Adaptable Program Loan (PSNP ADL) was created to improve resiliency and reduce the vulnerability of rural households in Ethiopia who experience chronic food insecurity. The most recent revision of this program targets 10 million people, and is delivered utilizing financial incentives and community-based programs. Households with able bodied adults are given cash transfers for 6 months of the year in exchange for participation in public works projects such as the development of infrastructure and water improvement which contribute to the creation of sustainable community assets. Those households who do not have a member who is able to work are provided with 12 months of transfers unconditionally. Both types of transfers reduce household reliance on credit unions, which contribute to a cycle of indebtedness. Within the communities, training is provided to allow households to diversify their skill set and earning potential while stimulating local markets. Monitoring and evaluation have indicated that this program has performed well in reducing food insecurity in chronically food insecure areas of Ethiopia with a number of target indicators being surpassed. A number of households reported benefits from the public works projects which included the construction of roads, classrooms, health facilities and improved water management [209–211]. |
|  | Importance | Very important |
|  | Theme(s) | Poverty Reduction |
|  |  | Food Security |
| 1. Community-based Nutrition (CBN) Program (2008-present) | Description | The Community Based Nutrition (CBN) Program aims to utilize cost effective and sustainable community-based interventions to improve the lives of mothers and their children. This program engages the community in malnutrition prevention and is an element of the umbrella Community Maternal, Neonatal and Child Health (CMNCH) Program. Activities under the CBN include growth monitoring and screening, expanded referral services, monthly community discussions, micronutrient supplementation and deworming, and creating multi-sectoral links to improve nutrition through agriculture, WASH and social programs. Evaluation activities conducted by this program indicate significant improvements in stunting in areas in which this program was well implemented [48,212,213]. |
|  | Importance | Likely important but late introduction, so may not have impact on national stunting change |
|  | Theme(s) | Health |
|  |  | Nutrition |
|  |  | Multi-sectoral Collaboration |
| 1. National Nutrition Program (2008-2020) | Description | The National Nutrition Program (NNP) aims to develop nutrition-sensitive interventions and address immediate causes of malnutrition by creating an enabling environment for evidence-based decision-making. It aims to improve maternal and child nutrition in addition to providing services for communicable and non-communicable disease-related nutrition services across the lifespan. It is implemented to strengthen initiatives that were not sufficiently addressed by the initial NNP launched in 2008 and to target emerging issues with a view to the multi-sectorial and multidimensional complexity of malnutrition. At the community level this program provides malnutrition prevention through nutrient supplementation, feeding advice and nutritional assessments. It uses a program called Engage the Media to advocate for nutrition policy both with the public and policy makers while building capacity of the media. School feeding and deworming interventions and fortification of oil and flour are additional points of contact for this program. Key programs under the NNP include: the Health Extension Program (HEP); Essential Nutrition Action (ENA); Community Based Nutrition (CBN); Therapeutic Feeding Programme (TFP); Enhanced Outreach Strategy/Extended Enhanced Outreach Strategy and Targeted Supplementary Feeding (TSF); School Feeding Program; Food Fortification; and WASH.  Monitoring for this program is done using a number of objectives associated with stunting, fruit and vegetable consumption, maternal and newborn feeding and care, micronutrient supplementation, policy creation objectives and HIV care. Major drivers of success in this program are the interest and collaboration of development partners and government as well as human capacity for nutrition. Challenges in implementing this program include inadequate resources and financial mobilization and inadequate facilities and governance structure [152,172,214,215]. |
|  | Importance | Likely important but late introduction, so may not have impact on national stunting change |
|  | Theme(s) | Nutrition |
|  |  | Health |
|  |  | Multi-sectoral Collaboration |
| 1. The Water and Sanitation Program (WSP) (2010-present) | Description | The Water and Sanitation Program (WSP) is a multi-donor partnership created by the World Bank to assist governments in providing affordable, safe and sustainable water access to their citizens. This program assisted the Ethiopian Government in creating a 5-year plan from 2011 to 2015 to support providing sanitation for Amhara, Oromia, Tigray and SNNPR regions, which account for 80% of Ethiopia’s population. Activities under WSP include hand washing and sanitation promotion, capacity building and monitoring. By 2014 improvements included 29% of individuals with access to sanitation as a result of WSP and 71% of individuals with access to sanitation as a result of other factors [216–218]. |
|  | Importance | Promising recent initiative |
|  | Theme(s) | WASH |
| 1. Food Security Program (FSP) (2010-2014) | Description | The Food Security Program (FSP) aims to substantially contribute to the reduction of food insecurity among rural households in Ethiopia and work towards achieving the Millennium Development Goals through the elimination of poverty. This program has 4 major components: the Productive Safety Net Program (PSNP) to simulate local economies and prevent asset depletion; the Household Asset Building Program to increase food security though the diversification of income producing employment; the Complementary Community Investment Program to develop community infrastructure in food insecure areas; and the Resettlement Program to provide resources for households in PSNP woredas [219]. |
|  | Importance | Promising program adopted through the Productive Safety Net Program (PSNP) |
|  | Theme(s) | Food Security |
|  |  | Poverty Reduction |
| 1. Integrated Community Case Management of Childhood Illness and New Born Care Implementation Plan/Program 2010 (2010-present) | Description | The Integrated Community Case Management of Childhood Illness and Newborn Care Implementation Plan/Program aims to strengthen the quality of maternal and child health at Primary Health Care Units (PHCUs). Objectives of this program include improving use of services and competency of health care workers; improving monitoring and accountability in primary care; ensuring ownership and evaluation of integrated community case management of childhood illness and newborn care; and improving supply chain management of resources for child health. This program is delivered through health care workers and distribution of related resources in the community, and utilizes pre-deployment training to fill gaps in health care worker knowledge and avoid interruptions in health services. Major factors for success in this program include the implementation of supportive supervision and on-the-job mentoring for staff and rapid scale-up and the focus on the health unit as a whole rather than individual health care workers. This program was not without challenges. Some challenges to implementation have included poor coverage and utilization of services, inadequate accountability, non-standardized referral mechanisms, mismanagement of medical supplies and inadequate budget implementation [220,221]. |
|  | Importance | Promising recent initiative |
|  | Theme(s) | Health |
| 1. Agricultural Growth Program (AGP) (2011-present) | Description | The Agricultural Growth Program (AGP) aims to diversity diets and improve nationwide growth through the increase and commercialization of agriculture in high potential areas. At the household-level, it aims to cultivate nutrient dense produce and animal products while supporting the participation of women and youth. It includes components of agricultural public support; agricultural research; small scale irrigation; agricultural marketing and value chain; and program management, capacity building and monitoring and evaluation. The evaluation of this program utilized a number of indicators including food yield and sale increases as well as dietary diversity [222]. |
|  | Importance | Promising recent initiative |
|  | Theme(s) | Agriculture |
|  |  | Nutrition |
| 1. Scale Up Nutrition (SUN) Movement Strategy (2012-2020) | Description | The Scale Up Nutrition (SUN) Movement, which Ethiopia joined in 2012, is an international, multi-sectoral collaboration which has agreed upon a number of malnutrition related goals to be achieved by 2025. This collaboration is motivated to end all forms of malnutrition by 2030. It has a number of global targets including a reduction in stunting, wasting and low birth weight, a reduction in anemia among reproductive age women, an increase in breastfeeding for the first 6 months and no increases in overweight, obesity and diabetes. It aims to reach these targets through implementation of proven nutrition interventions and the introduction of sustainable nutrition policy. Globally, SUN has goals to improve the overall nutrition of women and children through improved access to food healthcare, water and sanitation as well as improving feeding practices that contribute to positive nutritional outcomes. SUN has identified four strategic objectives, including: creating enabling political environments and improving the environment to increase collaboration; establishing best practice to scale up interventions; aligning actions around high quality plans with multi-stakeholder accountability; and improved resources for coordinated approaches. The evaluation of SUN has proven difficult as many elements are country-specific and have not been quantified. The way in which SUN has led to changes in stakeholder practices through increased collaboration and attention brought to the problem of malnutrition remains unclear [223–227]. |
|  | Importance | Promising recent initiative |
|  | Theme(s) | Nutrition |
|  |  | Health |
|  |  | Multi-sectoral Collaboration |
| 1. Sustainable Undernutrition Reduction in Ethiopia (SURE) (2015-present) | Description | The Sustainable Undernutrition Reduction in Ethiopia (SURE) Program focuses strongly on agriculture and was the inaugural government-led multi-sectoral nutrition program in Ethiopia. This program utilized mobile health and mass media to share its messaging while also providing education at the community-level on infant feeding and community nutrition. By 2020 this program has a goal of reducing stunting by 26% in four regions, namely Tigray, Amhara, Oromia, and SNNPR. The SURE program aspires to improve dietary diversity and complementary feeding, strengthen the system of health and agriculture overall and foster coordination across sectors to improve nutrition [228]. |
|  | Importance | Promising recent initiative |
|  | Theme(s) | Health |
|  |  | Nutrition |
|  |  | Multi-sectoral Collaboration |
| 1. One Wash National Program (OWNP) (2013-2020) | Description | The One Wash National Program (OWNP) pursues the improvement of sanitation and water supply, as well as good hygiene practices. Improved multi-sectoral collaboration and integration are major guiding principles of the program. It is built upon the pillars of good governance, efficient use of resources and capacity development to deliver WASH across all levels of the country. The program is broken down into the specific categories of rural, urban and institutional WASH along with overall capacity building and program management. Within these areas it seeks to improve hardware and infrastructure access, wastewater management, and support to hygiene practices [229]. |
|  | Importance | Promising recent initiative |
|  | Theme(s) | WASH |
|  |  | Health |
|  |  | Multi-sectoral Collaboration |
| 1. National Indicative Program for Ethiopia (2014-2020) | Description | The National Indicative Program for Ethiopia works within the broad objectives of the Growth and Transformation Plan (GTP) and the Climate Resilient and Green Economy Strategy (CRGE). It focuses on three main sectors for cooperation towards sustainable socio-economic development: sustainable agriculture and food security; improved quality of health care and overall health outcomes while addressing social determinants of health; and improvement and support for energy efficiency and the energy sector while phasing out support to the road sector [230]. |
|  | Importance | Promising recent initiative |
|  | Theme(s) | Agriculture |
|  |  | Food Security |
|  |  | Health |
|  |  | Multi-sectoral Collaboration |
| 1. Health Sector Transformation Plan (HSTP) (2016-2020) | Description | The Health Sector Transformation Plan (HSTP) was implemented to realize a long-term plan in which all Ethiopians are able to access health services when needed without significant hardship. This program is part of the Growth and Transformation Plan (GTP) and is built upon the pillars of excellence in health service delivery, quality improvement, leadership and governance, and health system capacity. This plan includes a number of targets for 2020 associated with a reduction in infectious diseases, child malnutrition and under-5 mortality. It has strategic initiatives to improve community engagement, financial management, emergency management, quality and equity, leadership capacity and regulation [231]. |
|  | Importance | Very important |
|  | Theme(s) | Health |
|  |  | Multi-sectoral Collaboration |

# **Supplementary Appendix 7:** Qualitative Results

Results from the in-depth interviews and focus group discussions are organized according to type of stakeholder, by national and community-level perspectives. Results are summarized according to key themes including basic (contextual) factors, nutrition-specific and –sensitive policies and programs, intermediate and immediate causes. Supporting evidence and quotes were selected to demonstrate a range of participants’ diverse perspectives on the determinants, and policies and programs.

**1. National Stakeholder Perspectives**

A total of 11 national key informants were interviewed in Addis Ababa (Table 15). These State and non-State actors were identified and recruited based on their substantial expertise and experience working in nutrition-specific and –sensitive sectors, institutions, and programs in Ethiopia and include multilateral, bilateral, government (national and subnational), academic/research, as well as local/international non-governmental organizations.

## **Supplementary Appendix Table 10**: Description of National Key Informants

| **Participant #** | **Organization** |
| --- | --- |
| Participant 1 | Nutrition team Leader, Federal Ministry of Health, Ethiopia |
| Participant 2 | Deputy Director, Nutrition International, Ethiopia |
| Participant 3 | WASH System strengthening specialist, World vision, Ethiopia |
| Participant 4 | Director , Alive and Thrive |
| Participant 5 | Head of Food and Nutrition, Ministry of Agriculture, Ethiopia. |
| Participant 6 | Professor of Nutrition, Addis Ababa University |
| Participant 7 | Basic Education Section ,Ministry of Education, Ethiopia. |
| Participant 8 | Advisor to the State Minster ,Ministry of Education, Ethiopia. |
| Participant 9 | Plan, Implementation, Monitoring and Evaluation Directorate Director, Ministry of Finance and Economic Development |
| Participant 10 | Nutrition Specialist, UNICEF Ethiopia. |
| Participant 11 | Child Health Specialist, Independent Consultant |

**Nutrition and Stunting Trends in Ethiopia**

National key informants acknowledged that the country has documented a significant reduction in the prevalence of stunting. However, they also stated that the stunting burden is still high considering the absolute number of stunted children in Ethiopia.

*“As we know it and as researches also show the trend is decreasing. Twenty years ago, it [stunting] was 58% but now its 38%. This mean in the past 20 years the rate is decreasing but the reduction is a very slow rate. Stunting is declining but the rate is low. I personally think it’s on a slow rate. Both Ethiopia and the SDG took commitment in making the rate of stunting zero by 2030. However, in the past 20 years we reduced stunting by 1% every year and now it’s at 38% and we are left with only 11 years for 2030.”* [Ministry of Education Representative]

Key informants elicited multiple reasons for the observed reduction of stunting in Ethiopia. These included the overall improvement in the economy, access to health services, access to education, agricultural productivity, WASH and emergency preparedness, and development programs such as the Productive Safety Net Program (PSNP).

“*The first is development activities the government set on agriculture. There are works done on agriculture and health sector. The second is governments plan for emergency prevention and preparedness in case of food insecurity. In case of food insecurity, the government contributes in quick and daily provision of food. The third is productive safety net program: This is helping those in need of help which can be by money or providing of food. This has its own contribution. The others are economic expansion, employment rate etc. This has contributed directly or indirectly for reduction of children stunting*.” [Ministry of Finance Representative]

Key informants acknowledged that reduction of stunting is not uniform across regions. There are regions demonstrating higher prevalence of stunting and there is a difference in the burden of stunting within localities in the same region. They mentioned that poor infrastructure, recurrent drought, food insecurity, religious fasting, poor health services, and illiteracy are reasons for the observed regional variation in stunting. Respondents stated that they observed regional variations in states such as Amhara and Afar regions which have high stunting burden.

*“The drop is not uniform across regions. There are regions with high prevalence and still suffering from that like Afar and Amhara. Their prevalence is 46% or 48% which is near to 50%. This means one in two kids is stunted. It is essential to see exact number but Amhara and Afar have high prevalence. When we see the two regions they are completely different in socio demographic, socio economic and agro ecological status. Amhara region is among highly productive regions except few food insecure woredas. Afar region have chronic food insecurity and highly dependent on support. Infrastructure of Afar region is weak. So the cause of stunting is completely different for these regions. In Afar it may be related to complementary food. In Amhara it is related with behavioral and religious issues.”*  [iNGO representative]

**Basic Drivers of Stunting Decline in Ethiopia**

The key informants at the national level identified multiple key factors that have potentially facilitated the observed stunting reduction in Ethiopia over the past two decades. These include urbanization, poverty reduction, women’s education and empowerment, labor migration and remittances, as well as sociopolitical contexts including peace and security, decentralization, political commitment, and change of political ideology.

*Urbanization*

Key informants indicated that the increase in urbanization is a noticeable phenomenon that continues to grow fast in Ethiopia. The urbanization seen in Ethiopia is often characterized by an increase in migration from rural areas to urban cities as well as massive constructions in the cities. The national key informants perceived that the increased urbanization was an important factor to the country’s stunting reduction and multiple pathways were elicited. The pathways included improved access to services and supplies, stimulating the economy, job creation, and improved income and human capital.

Here is an example on how urbanization has influenced stunting:

*“The importance of urbanization is that it helps people to get services nearby. It helps to manage services that are dispersed in to one area; both the health services and educational centers and in turn stimulate the economy. It will also improve the supply. If we don’t increase the supply, then it will cause problem in sharing. But the positive side outweighs the negative. In urbanization, people live together when their economy increases. This is everywhere in the world. Urbanization is one of the reflections of development.” [*Ministry of Finance Representative]

However, urbanization was also seen as a potential challenge to the stunting reduction by affecting the breastfeeding practices as mothers who work, food safety practices, and causing a reduction in crop production as farmer’s landownership is threatened by expanded urbanization.

The following examples illustrate how urbanization has influenced stunting in the country over the past two decades:

*“When there is urbanization there is increased access to health and improved literacy rate. But there is also a down side to urbanization, because it has led to increased slum areas. Breastfeeding practice has improved in our country but it is still low, it is around 50%. But breastfeeding practice is a challenge in urban areas because there are mothers who are employed. There is definitely a challenge in breastfeeding in urban area, so there is a plus and minus side to urbanization.”* [Child Health Specialist and Consultant]

*I think urbanization has effect on farmers. I don’t know the political view but it is my opinion. This is because farmers’* *life bases on their land. They eat what they produce. If they lose their land because of urbanization, it will be difficult for them to survive. The family will end up in streets and labor works. Even though there is some compensation, farmers don’t know how to use their money because we didn’t work on behavioral change till now. Farmers’ life is different from buying food and eating. Urbanization will be good if we give another land for farmers as replacement. [*Ministry of Agriculture Representative]

“… *There is a direct relation between urbanization and globalization and nutrition and it is growing faster. You can see this everywhere; there is construction and large number of populations around. What I am concerned about is who will give services in rural areas*…” [FMOH Representative]

*:.. I hear that people are migrating to urban areas. I also agree that the number of people living in urban area is increasing. I believe that if the number of people living in urban area is not proportional with the available resource it will cause a problem. Though I can’t say that this will cause stunting but in my opinion when there is urbanization knowledge will grow. People have knowledge in these areas and they will start implementing this knowledge…*” [UNICEF Representative]

*Poverty Reduction*

Across all the national key informant interviews, respondents cited that the rate of poverty in the country had declined dramatically over the past 20 years. Moreover, the key informants indicated that not only was the general decrease important but also the absolute amount of poverty reduction.

The comment below from a respondent from the Ministry of Finance and Economic Development illustrates these points:

*“I need to refer the percent not to cause of factual errors. Please wait me… There is a poverty trend … Lets’ go and see the poverty trend …. Because it’s a factual thing … When the 1^st^ poverty survey was done …. In 1995/96 the national figure was at 49.5% and in 2003 it reduced to 29.6%. Now, according to the 2008 survey it’s reduced to around 23%. This data was around here…. Let me follow the fact … you have to follow the fact … Yes, its 23.5% on the survey done in 2008. Therefore, it has decreased this way from time to time. This means the poverty line has decreased almost in half from 1995/96 to 2016. It has reduced with a good amount…” [Ministry of Finance and Economic Development Representative]*

According to the respondents, the rate of employment has improved in the country and resulted in improvements of food security in the respective households, which in turn contributed to improved nutritional status of children. Moreover, the respondents provided programs or actions that have potentially played significant roles in reducing child undernutrition in Ethiopia. The relevant programs elicited in the discussions included the Agricultural Transformation Program, the Disaster Prevention and Preparedness Program, the Productive Safety Net Program, and the government’s public investment in construction of roads and buildings.

Below are some quotes that capture this well:

*“Poverty is decreasing. National income is good. Productive safety net program like asset building is a key program for the changes we have seen including stunting reduction. This program helps households to cop up during shock/lean periods.” [INGO Representative]*

*“…The major thing that made a change is the focus given to the agriculture sector by the government. These are, the allocation of manpower in the agriculture, supply of fertilizers and use of different alternative methods for production like irrigation has increased the production of food/crops. On the other hand, the disaster prevention and preparedness commission has strengthened itself and provide food donations for areas where there is food insecurity. The 3^rd^ one is the Productive Safety Ney program. These have made their contributions. Especially they have contributed for the reduction in people affected by sever poverty and also they contributed for it not to expand.” [*Ministry of Finance Representative

*Improvements in Education & Women’s Empowerment*

For many of the respondents, there has been considerable improvement in women empowerment and girls’ education across the nation in the past 20 years. The respondents indicated that the government has taken the issue of increasing enrollment of female students as one of the main agendas in the education sector. The government’s actions to improve women’s education included arrangements to retain females in schools and affirmative actions on enrollment marks to encourage female students to join higher education. Although the overall change was considerable, respondents did not agree that the proportion of girls/females in the school population was satisfactory especially at higher education institutions such as colleges and universities.

The following extracts from the interview transcripts illustrate some of these points:

*“…The number is big but it has not yet become balanced. Currently the gender parity index is not equal. The number of dropouts is also high when we see the second cycle of primary education (5 to 8 grade level). You will see that there is huge dropout of girls. When they are enrolled in the primary education the number is relatively close but as you go higher the percent of women will reduce. For example, in the university in the 1st degree level, only 32% of them are women. But at a lower level … if you see “Amhara’ region and Dire Dawa city administration it’s around the women to men proportion is 50:50. When you go higher let’s say at a master’s level the enrollment is around 20%. Therefore, the proportion is still growing up.” [Ministry of Education representative]*

*“The gender proportionality is increasing from previous years. This is also due to government’s support and this support contributed to their increased involvement. Women are now achieving better results and competing in class as 1^st^, 2^nd^, and 3^rd^ places and this is increasing now. The proportion has increased from previous time in great number. One of the reasons for this is government support and supports from schools which have contributed their own parts. And also women competence has increased contributing to the high increasing situation from previous times.” [Basic Education advisor, Ministry for Education]*

At the community level, respondents indicated that there has been a change in perception regarding the importance of education, particularly girls’ education. This is perceived to be the result of strong programs that promote women education in the country.

*“…And when a student is absent the teacher asks the reason and if it is repeated and if he hears that she is about to get married then the teacher will link it to the legal bodies and an action will also be taken. There are such improvements and also an increase in the community awareness about women, the fact that most of the health extension workers are females, most teachers being female and attention given to girls’ education by the government. It’s improving in general.” [Ministry of Education representative]*

Another ministry of education representative further emphasized the importance of shifting community beliefs:

*“These are the things. The other thing is role model, which I told you earlier, which is one factor. When women students are equal with the male students and achieve results that are better than males, parents and families will send girls to school. Parents and families now understand the use of education. So now they are sending both girls and boys to school.” [Ministry for Education representative]*

The national experts affirm that there was a direct relationship between women empowerment and girl’s education and improvement in child nutrition. Respondents indicated that educated mothers could challenge traditional practices that are not good for child nutrition, better space births, and provide better foods and care to children.

*“Education has a direct relation. Because even if we have the resources and if there is lack of awareness on how to use them, it can cause stunting. Therefore, it’s not only about having the resources but also we need the knowledge on what pregnant women should eat, how to feed a child and what foods to eat at the adolescences age…”* [iNGO representative]

Another iNGO representative also mentioned the relationship between women empowerment and improvement in child nutrition:

*“…Yes, like I said educated women are becoming mothers. You observe this during surveys. This creates a fertile ground. Recently I visit one educated mother. Her children attend school. The family is challenging common traditional practices. They question why. The number of educated mothers increased over the past 20 years and it will increase*.” [iNGO representative]

*Remittances & Labour Migration*

Key informants discussed the common types of migrations as well as the mechanisms and strategies through which migration could positively or negatively impact stunting in the Ethiopian context. The types of migrations identified by respondents included economic/labor migration, disaster related migration, as well as in country and international migration to foreign countries.

*“If you’re asking me about migration, migrations can be due to different reason. One reason is natural disaster. People migrate to escape from such disaster. They will migrate to areas that don’t have such problems and sometimes migrate to far areas. The other one is manmade disaster. The repeated conflicts we see these days in different areas will cause people to migrate. The other one is Economic migration. When we see it as a country now the number of people migrating to the Middle East country is increasing. They are migrating to look for a better life. Therefore, whatever reason is migration has an impact on children and it might also cause stunting.” [*iNGO representative]

The labor migration to urban cities for construction work and other similar jobs does not necessarily result, as discussed by the key informants, in improved income and in turn improved household economy and nutrition. The national experts highlighted that there is a high chance of unemployment in the urban cities since the number of jobs may not be in line with the volume of migration and hence labor migration could negatively impact child nutrition.

*“In our country we might not call this migration… but due to the urbanization and increased activities like construction works in urban areas, people will migrate from rural areas to such urban areas to find a job. There will be sharing of resources and this might cause increased rate of un-employment in the urban area …. If there is high un-employment rate in urban area and if those who migrate start a family and have kids in the urban area, then this might create a problem in the children food intake.” [*Ministry of Finance Representative]

*“I can’t say much about this but those who travel outside Ethiopia, such as those who travel to Arab countries might help advance the financial status of their family by sending money, so that might have contributions because it can help improve the capacity of the family to raise a child. But I don’t think internal migration where people move from rural area to urban area has contributed for stunting reduction.”* [Child Health Specialist and Consultant]

However, the international migration to countries in the Middle East for in house work is perceived to be directly and indirectly influencing child undernutrition depending on whether the remittances sent back home are properly managed and used. In addition, the currency transfers from citizens living abroad is increasing the national foreign currency and its deposits. This would improve the country’s capital to buy consumption goods, drugs and others, expansion of infrastructure, and supplies that could directly or indirectly impact reduction in child undernutrition.

*“An increase in “HAWALA” increases the foreign currency and its deposit. An increase in the foreign currency deposit will have importance in the country’s capital to buy goods, drugs, or anything in general. Therefore, it is important to pay for things the country imports from outside or materials the country doesn’t produce since they are bought in foreign currency. It will also be used for expansion of infrastructures for example: road construction will make schools accessible and to sell what has been produced. Therefore, the foreign currency will have importance to increase these supplies. These are foreign currency we get from selling things from outside the country and foreign currency we get from ‘HWALA” is also important.”* [Ministry of Finance Representative]

*Sociopolitical Context*

For many of our respondents, the sociopolitical context has impacted stunting reduction over the past two decades. National informants mentioned that the government’s ideology and commitment as well as the decentralization and democratization process are core components within the sociopolitical context that are potentially driving the observed improvements in nutrition. However, national informants also highlighted that the recent absence of peace and security is challenging the stunting reduction.

Respondents mentioned that the government’s actions on decentralization helped regional states to improve agriculture productivity, infrastructure, and health services at the grassroots level. Key informants stressed the importance of political commitments over other factors as the main driver for regional states to perform better, as seen in the difference in undernutrition rates between regions.

*“This is from the time where the country developed decentralization into regional system. This has a positive impact because it will help in an increment of production. It will also help in development of basic infrastructures. This will indirectly help the regional economy to stimulate. It will also help to report problems as soon as possible. Therefore, it has a positive impact in such aspects.” [*Ministry of Finance Representative]

Few of our respondents mentioned a change in political ideology from the monarchial system to the socialist Derge and later regimes during the interviews. In the monarchial system, the ownership of the agricultural land was owned by few individuals and the landlords. This change in ideology, also called “Land to the tiller,” presented agricultural land reforms including ownership of land and governance system whereby the majority of the rural poor farmers owned agricultural land for their use.

The following extract from one of the interviews illustrates the relation between production and consumption within the “political ideology” dimension:

*“It was in the derge regimen where land was given for society lastly. Young peoples if they don’t get a land from their family then they will not have at all. Even if they get per capital size is small when it is given for all children. Land is a basic asset. The fundamental thing is access to basic resource which is land. If land is not available there will not be production if there is no production, there is no consumption.” [iNGO representative]*

Respondents highlighted that crop production reflected the level of peace and stability.

*“In case of political factors: I don’t know in the past 20 years since I have been working in ministry of health for the past 10 years only. It was peaceful until the past 3-5 years then conflict has been rising everywhere, which has a high impact. And due to the instability peoples are not focusing on farming even in the winter time then displacement follows. The production that people got will also be destroyed during the conflict.”* [Ministry of Health representative]

Despite the longstanding peace and security, key informants highlighted that in the past five years there has been an absence of peace and security coupled with internal displacement, which has been challenging the exemplar reduction of child undernutrition in the country. The current instability has shifted the governments’ and donors’ focus from the development of nutrition programs to emergency responses.

*“…When you have too much conflict and displaced peoples millions of birr/dollar will spent to support. So the nutrition development projects will be deprioritized. If you remember in 2016, there was high number of internally displaced people and humanitarian crisis. During this time, funding opportunity was significantly decreased for projects working on improving developmental nutrition. Because government prioritized emergency or short term interventions than long term project. Like I told you if you go to Afar with a 10 million long term project and a 3 million short term project; they prefer the short term program. It doesn’t make sense for ministry of health if you bring long term development project when there are millions people who needs emergency interventions. So these issues have effect...”* [iNGO representative]

**Nutrition-Specific and –Sensitive Policies and Programs**

National key informants identified nutrition-specific and –sensitive programs that have influenced stunting reduction in the country from 1990-2016 and further discussed programs/projects that have both nutrition -specific and -sensitive components. These include the National Nutrition Program (NNP), Sustainable Undernutrition Reduction in Ethiopia (SURE) and the Health Extension Program (HEP). Some of these projects/programs were implemented through a multi-sectoral collaboration approach.

Barriers and facilitators of program/policy implementations were also discussed during the interviews. Key informants stated that the National Health Policy of 1993 was the primary guide for the initiation of these programs/projects. Poor budgeting, inadequate human resources and capacity of professionals as well as problems related to accountability were the barriers identified for effective implementation of these programs. Key informants indicated that although the scale of implementation and effectiveness of programs differs from one program to another, all nutrition programs contribute to improvements in nutritional status in Ethiopia. Many key informants identified the HEP as the main driver to the observed stunting reduction in Ethiopia.

The nutrition-specific and –sensitive programs mentioned by the national key informants are described in detail below:

**Health Extension Program (HEP)**

The HEP is a community-based health program adopted by the government of Ethiopia in 2003 with the aim of achieving universal health coverage. The program had 16 packages and two additional packages were added later on. Key informants felt that integrating nutrition into the extension activities has led to significant improvements in nutritional status of families. In addition, key informants stated how the HEP addresses nutrition-sensitive components by preventing disease, expanding immunization services and generally making the family healthy. Key informants also mentioned the substantial contribution of the program towards hygiene and sanitation by creating awareness about latrine construction in the community. According to the key informants, the fact that health extension workers are recruited from the same community helps them work hard and makes them acceptable by the community. Key informants also identified poor work motivation as a key challenge regarding the HEP.

*“… Health extension program is working on prevention at community level. Health extension workers provide ANC service for pregnant women, provide health education on dietary intake and give iron foliate supplements. She follows if they are taking the iron foliate or not since she lives nearby. Therefore, this has a high impact. One thing I have seen in the socio-political area is the decentralization of the health extension program to reach the community and it has made a huge impact…”* [*iNGO representative*]

*“Main achievements of the program include, improvement in immunization services, which is, almost all children are immunized against polio, measles and TB and children who are immunized can easily be traced in the community, mothers use of family planning service has increased, there is improved hygiene and sanitation from the previous time and improved latrine utilization etc.”* [*iNGO representative*]

**National Nutrition Program (NNP)**

The NNP is a major nutrition program designed by the government of Ethiopia in 2008. Before the start of the NNP, the health sector was the primary sector supporting nutrition. Later on, the multi-sectoral nutrition approach was introduced involving thirteen sectors. The key informants highlighted the significant contribution of this program towards improving nutrition in Ethiopia. However, key informants also indicated that it is too early to identify the impact of the program, as it has not been fully evaluated yet.

*“…. NNP is a basic document for all nutrition specific and sensitive programs. So I think it was one great program in the country …”* [iNGO representative]

*“… This strategic document puts high-level impact indicators that can be assessed by conducting a survey. For example, currently the coverage of GMP is 46%. Timely follow up of GMP allows us to know the stunting rate and the aim is to reduce it from 38% to 26%. We don’t exactly know the rate of stunting now because we didn’t conduct a survey yet ... What I can now talk about is works done on capacity building. Integrated training modules are prepared on the strategic objectives and training has been provided by the national team for all the regions and city administrations. They also cascaded the program and trained respective health care providers. One round training was also given to health extension workers by IRT (integrated refreshment training) module, which is taken from NNP contents*.” [Ministry of Education representative]

Key informants indicated that government commitment, support from donor organizations, engagement of the private sectors, and implementation of programs such as the Health Sector Development Program (HSDP), Sustainable Undernutrition Reduction in Ethiopia (SURE), and Growth Through Nutrition (GTN) were the prominent facilitators for the program. The multi-sectoral nature of the program was identified as both a facilitator and as a challenge for its implementation. Although the platform helps to improve the need for coordination of various sectors to solve the country’s nutrition problem, key informants indicated that only the health and agriculture sectors are actively working in the implementation of the program and that the poor coordination of the multi-sectoral platform is negatively influencing the effective implementation of the program.

*“… Multi-sectoral task needs collaboration, planning, and evaluating. However, we are not doing it. There might be repetition of tasks by more than one sector because of communication gap. Nutrition interventions need collaboration; for example, if agriculture is working on home gardening, water sector has to facilitate availability of water, education has to work on awareness creation and health sector has to do cooking demonstration. This is how we will bring change…”* [Ministry of Agriculture representative]

**Sustainable Undernutrition Reduction in Ethiopia (SURE)**

SURE is a government-affiliated project with both nutrition-sensitive and -specific interventions at the community level. The main components of the program are primarily food and dietary diversification of complementary feeding promotion, teaching and awareness creation, and conducting campaigns. Multi-sectoral coordination is also one of the core program component. Key informants explained the multi-sectoral coordination component of the program by discussing how the health and agriculture extension workers make joint household visits to create awareness in the community by identifying households with pregnant mothers and children less than 2 years of age. The key informants also mentioned the contribution of the program in arranging cooking demonstrations and community dialogue.

*“… It’s a 5-year program and it has not been evaluated yet ... from the process indictor perspective there is no objective that is not being implemented. The major one was preparing training manual on (IYCN/NSA), facilitating guide, package and job aids on mothers and children’s dietary intake. The training was provided for more than 7000 health extension workers and agricultural extension workers. This is how they (the health extension workers and DAs) did the Joint household visit”* [Ministry of Health representative]

**Productive Safety Net Program (PSNP)**

The Productive Safety Net Program (PSNP) is a social safety net program that supports chronically food insecure households, responds to shocks, and promotes sustainable development. The program started in 2005 and provides conditional food or cash transfer to households that joined the program. Under the PSNP, pregnant and lactating mothers are exempted from participation in public work and are allowed a direct benefit with soft conditionality/criteria. These include antenatal care, growth monitoring and promotion (GMP), family planning, behavioral change, communication programs, nutrition services, and other maternal and child health services. According to national key informants, the PSNP helped the poorest of the community by helping households build assets and become independent to feed their family better. Key informants indicated that the number of households receiving support from the program is gradually decreasing, implying resilience of families (households) for shock.

*“… This program helps the community to save and build household asset. Asset leads to income and wealth so the family can have resilience during shock periods. They don’t have to sell their cattle and other things. When the family buys cow then they will have milk and butter. That is an asset. They either can eat it or sell it. That is how I understand the program.”* [iNGO representative]

*“… Making PSNP-4 nutrition sensitive is one achievement for me. It is contributing for stunting reduction.”* [Ministry of Agriculture representative]

**Health Sector Development Program (HSDP)**

The Health Sector Development Program (HSDP) started in 1997. The main objective of the HSDP is to provide comprehensive, integrated, and cost effective primary health care service, with a focus on communicable diseases prevention and control, nutrition, environmental health and hygiene, reproductive health, and immunization. The HEP started in the second phase of the HSDP. Key informants highlighted that various initiatives were implemented by the HSDP to achieve the Millennium Development Goals (MDGs), that attention was given to maternal and child health services, and affirmed that the HSDP has contributed towards improving health and nutrition in Ethiopia.

“…*HSDP is one of the program, which I believe have great contribution for stunting reduction…in HSDP, we have achieved immunization, there is improvement in access to health in almost all regions and there is improvement in IYCF practice at community level such as breastfeeding and so on. There was a continuous evaluation on child health sensitive programs …”* [Child Health Specialist and Consultant]

**Underlying Causes**

*Improved Household Environment: Water, Sanitation & Hygiene (WASH)*

National key informants discussed contextualized improvements in the household environment in relation to separation of human and animal houses, presence of a separate kitchen, overcrowding, and availability of a toilet. However, there were mixed opinions and views regarding improvements in the household environment over time. For instance, some informants stated that there has been increased separation of human and animal houses while others stated that animal and human cohabitation continued to be common.

“*I had the chance to see some areas, there are improvements being made on separating human and animal’s house, having another place for cooking, and making shelves for material/utensils storage and on household & personal sanitation. The health extension program is doing a good job on that*.” [Ministry of Health representative]

*“If we are talking about the households in rural area there is still a problem of crowding. I visited a lot of places in rural areas and animals and humans are still living together and the cooking places is still inside the house, it’s very difficult to say there is improvement because the farmers give you reasons like others might steal their animals if they prepared another place for the animals only. Even though it’s just my thought and it’s not research based there is still no improvement in household crowding. Even in urban areas, people don’t have toilets, they use water bottles and plastics for toilet, that is why we see urine in water bottles in the streets because the house is so small, and it’s a place for every activity as they don’t have kitchen and bed rooms.*” [iNGO representative]

According to the respondents, there is a modest improvement in the accessibility of clean water. The respondents discussed that the improvement is partly due to improved access to road and increased donation. However, the respondents highlighted that the improvement is not enough as there are many communities that continue to walk long distances to obtain clean water. The water access problem is especially concentrated in rural areas across the country compared to the urban areas. Respondents highlighted that the lack of maintenance of water sources contributes to the decreased access of safe water.

The following extracts illustrate these points:

“*I can say that there is improvement in the accessibility of clean water. But our big problem is we start the work but we don’t do it in a way that it could be sustainable. Let’s say you built water for some school and you don’t arrange maintenance. If you go back after a year, because of simple problem you find it not functioning and very dirty. So it will be back to zero. Beside the number of water facilities, we build we should work in collaboration on making it sustainable.*” [Ministry of Health representative]

“*There is improvement but it is not enough. Still there are peoples who walk one hour to get water. If they brought one jar they won’t use it for washing. They will use it only for drinking and cooking. There is problem in availing WASH facilities; this is where the donors should involve*.” [Ministry of Agriculture representative]

“*There is also an improvement in water supply as well. But there is still a problem especially in rural area, these people have to travel lots of Kilometers to fetch water, they also use contaminated river water*.” [Ministry of Health representative]

Key informants discussed sanitation and hygiene in terms of access to latrines, utilization of latrines, open defecation in free kebeles/districts, and hand washing practices. Respondents highlighted that although there is increased construction of latrines, a large gap remains regarding proper utilization. However, they mentioned that the HEP has significantly contributed to the construction of pit latrines as well as the promotion of hand washing and sanitation through community engagement.

*“Back then, those who didn’t use toilet was 70% to 80% but now it has reduced to 40%. This is achieved by collaboration of health offices environmental and sanitation department, NGO’s and they are still working on it. In addition, now, there is a WASH national program and better hand washing practice before eating and preparing food in the community creating a high impact in improvement of hygiene and sanitation. Also safe water provision is reaching around 65% in collaboration of government and partners.”* [iNGO representative]

*“Pit latrines were also built but they don’t give the service we want. They don’t have hygiene and sanitation facilities. Without available WASH facilities, it doesn’t give sense if we teach about behavioral change.”* [iNGO representative]

*“again thanks to the HEP a lot has been done regarding toilet utilization, especially on South and Tigray region there are open defection free woredas. A lot has been done regarding water sanitation through the HEP. The health extension workers provide education by going house to house to prevent water contamination by teaching them to use narrow mouth jar, to use boiled water and so on.”* [Ministry of Health representative]

*Food Security & Feeding Practices*

Key informants described food security in terms of production, crop diversification, agricultural technology, land size, population growth, rainfall/irrigation, market driven production etc. Many of the key informants indicated that there has been increased agricultural production over time. However, respondents indicated that a problem remains regarding the diversification of agricultural products.

The following extracts describe these points:

*“as per my understanding in the past 20 years, more work has been done on improving production. The main focus is to provide food for the population. Even though I can’t tell you the figure in number but the production is increasing. As we hear it from the media and from the news, the production is increasing. But after the introduction of nutrition sensitive agriculture in the past 3-4 years, the work being done is not only on production. We can’t say there will be nutrition security by producing only maize. Therefore, the agriculture sector is working with nutrition sensitive agriculture strategy, which is focused on food diversification and micronutrients. Previously production of fruits and vegetables were not given attention. But now the agriculture sector is working on this with more focus. More focus is given for production of fruit and vegetables, fish farming, animal breeding and mixed farming (production of different types of crops.”* [iNGO representative]

The key informants also indicated that there is decreased per capita yield due to actors such as the increased population size, lack of rainfall, lack of irrigation, and modern farming system.

*“Agriculture is the same as when we were children. They are still using oxen and pick axe. Back then they were working only on productivity. But productivity only can’t make difference we have to make it nutrition sensitive agriculture. Now National nutrition agriculture strategy is launched and it is one positive forward movement.”* [iNGO representative]

*Improved Access to Health Services*

Key informants discussed improvements in health services in terms of the number of health facilities built, the improved access to the health service, human resources, supplies and equipment, and quality of health services.

Across all the respondents, there are substantial improvements and a huge expansion of health facilities in the country over time. The expansion of health services was related to the massive construction of health posts and health centers across all regions in the country. Informants indicated that the observed expansion of health services and the health posts in each village has resulted in improved coverage of basic health services, counselling services, and improved practices among mothers in the community.

The following extracts elucidate the views:

*“I worked for 16 years in health system after graduation that is how far I know. There was no infrastructure when I was working at woreda level. Now it has improved and number of staff has also increased. Primary health care acceleration is very good. It was started by 300 health centers now it is more than 3000 health centers and more than 30,000 health posts. If we see the woreda I used to work, it used to have only 7 health facilities for 57 kebeles but now all kebeles have their own health post. The change is visible we can see health extension workers and midwives. Attention is given for mother, child health and child care nutrition. This shows that, nutrition services are engaging at lower level. Activities which were done through campaign like Vitamin A, CBN and CHD is now becoming routine activities. There are many midwifes so I think Iron folic acid distribution will be good. Changes in the health system brought a favorable ground for nutrition.”* [iNGO representative]

*“The health system improvement is the reflection of the country progress. There is significant improvement in coverage. In previous years’ peoples have to walk a long way to reach health facility, but now there are health centers, health posts and hospitals near to the community… There are still some regions which walk longer away to health facilities carrying patients.”* [iNGO representative]

Key informants also indicated that the massive training and deployment of health extension workers to heath posts in each village is a significant contributor to improved health service access. The deployment of health extension workers at the village level has helped to bring services such as immunization, health education, nutrition education, vaccination, antenatal care and referral services closer to the community level.

*“The decentralization of the health extension program to reach the community has a high impact. Primarily the program made services available at the “kebele” level. Whenever something happened, they will go and visit the health extension worker since she lives nearby. The major and the main one is that the health extension worker provides ANC for all pregnant mothers and when there is problem, she will find solution at the pregnancy level. She also provides health education on dietary intake. She provides iron foliate. She follows if they are taking the iron foliate or not since she is nearby. Therefore, this has a high impact. One thing I have seen in the socio-political area is the decentralization of the health extension program to reach to the community and it has made a huge impact.”* [iNGO representative]

*“...Relatively it’s good. We can see at a “district” level that there is a health center and satellite health posts. There are health extension workers there providing services like immunization, health education, nutrition education, vaccination etc. Health extension workers will refer the patients to health center when there are cases that are complicated, then health center to zonal hospital then to referral hospitals. There is a health system structure from the community to a higher level of care.”* [iNGO representative]

Although the health system expansion has contributed to the observed stunting reduction, key informants indicated that there is a need for multi-sectoral collaboration to bring about the desired level of change in stunting reduction. They have also mentioned the lack of nutrition experts at heath facilities that can deliver the required quality nutrition services.

The following extracts elucidate these points:

*“We need to assign one responsible person at each level. That responsible person should be an expert to understand the magnitude of the problem of nutrition on the population and children but we have very few experts on this area as a country. We also come from this population and it used to not bother us until we understand the situation. We had that understanding by working in this area then we started talking about the issue. The universities need to produce many experts on this area to address the issue up to “woredas” and health centers. In the health centers, you find Health officers, nurses and pharmacists but what about nutrition experts (clinical nutrition and community nutrition). In hospitals, doctors decide everything including the findings, the drugs and tell patients to eat or avoid certain foods. But if we have nutrition experts to give counseling on patients’ dietary intake, the drugs will also be effective. In some cases, the person might be cured without taking medication just by improving his/her dietary intake and building his/her immunity.”* [Ministry of Health representative]

*“The focus given for nutrition is still low. They are not addressing prevention of under nutrition and stunting. They are still doing acute malnutrition/SAM management. Because they are being evaluated based on that. This means they prefer to manage the aftermath than prevention. Even after management they don’t counsel properly to prevent other family members or the next episode.”* [iNGO representative]

In addition, key informants pointed out a growing problem regarding the low motivation of health care providers working in the health system. Moreover, the quality of health services delivered in these facilities is rated as poor.

*“Health care providers have no passion and they are demotivated. Let’s say you give training this month and if you go after a month the routine work will not be there. Even if you give them a job aid to support the counseling they don’t use it. When you ask them why they say there is high case load but it is more of motivation issue. This is a push factor for the mothers. If they don’t get a quality care they will not come for follow-up. There is also high staff turnover.”* [iNGO representative]

*“The health service is also poor; we need to work to improve the service provider’s knowledge on nutrition. So if we work on this I think we can improve a lot in the future, but I think there is still a lot remaining at this stage.”* [Child Health Specialist and Consultant]

**Immediate Causes**

*Dietary Intake*

In response to our question about improvements or changes in diets and infant and young child (IYC) feeding over the past two decades, respondents highlighted a general improvement in the “concern or care” mothers give to IYC diet and the type/quantity of diet given. For example, mothers used to give whatever was available in the house but now they understand the benefits of a “good” IYC diet and tend to be concerned and try to explore options to access the IYC diet. The observed change is attributed to improved awareness on the benefits of a “good” IYC diet, agricultural production, and the knowledge on the components of IYC. However, for many of our respondents, diet diversity, quality, and accessibility remain major concerns in the country.

*“Gruel [made of similar flour to that of porridge but thinner] used to be a universal meal for children Now it is changed. As of 2004 we started the IYCN and ENA promotion. Thin porridge, any mother can tell you about it now because it is promoted through health extension workers. There are mothers who still give gruel for their children but I am sure the number of mothers who uses thin porridge outweighs them. We don’t know how much the thin porridge enriched with essential nutrients but there is improvement…” [iNGO representative]*

*“There is some improvement. Back then it was believed eating variety foods as luxury. Now the community is started understanding the benefit of eating diversified food though there is availability problem. Mothers understand the benefit of good diet on the fetus. The community also understands the benefits of feeding children with diversified food on growth, mental development and school performance.” [Ministry of Agriculture representative]*

Getting a variety of food to feed children was a major concern for households as there is a challenge in the availability of foods throughout the seasons and the challenges families face in buying foods due to physical accessibility in markets. Moreover, the amount and quality of diets for children is perceived to be poor and is linked to the dominant cereal and grain based IYC diets and not being able to provide “nutrient rich or good” foods including animal source foods such as eggs, milk, and meats.

*“Dietary diversity score figure is always burdensome. It shows us we are not successful on it so we have to think and work more. The diet is nutrient deficient and lack animal source food, it is mainly cereal and grain. Our food has no problem on energy content. We may eat much for compensation even if there is much fiber in it. But still it is deficient in zinc, which is important for stunting prevention. Iron, we can get from contamination. Calcium has relation with growth and stunting but milk is not available now….” …” [iNGO representative]*

*“Under quantity sustainability still matters. You think a child is fed throughout a year? The availability is also an issue because there are types of foods that are not available in areas. You can’t feed it the same thing always like "Teff" or corn. But it needs to be diversified. So there need to be accessibility of diversified food. But not only diversity but also safe nutrition, accessibility and utilization of diversified food. And we need to see its availability throughout the year. So if this is not achieved it does not matter if we tell or teach a mother to prepare and feed a balanced diet. So to do all this food needs to be available. These foods need to be at least available at the market even if it’s not produced at home.”* [Ministry of Health representative]

*Maternal Characteristics*

For most of our respondents, there had been a substantial improvement in maternal health related to the family planning programs in the country. The family planning program, coupled with the exclusive breastfeeding culture in the country has contributed to the significant reduction in the country’s fertility rate. The key informants also noted a shift in the community’s perception of having many children for future earning source.

*“Based on what I heard it’s decreasing. I don’t know the exact data but previously it was around 8.5. Now the fertility rate is around 5 point something. This has a high impact to reduce malnutrition. I also hear that family planning utilization and birth interval is increasing. This has an impact on reducing malnutrition.” [iNGO representative]*

*“Back then it was believed that having many children as a good thing. Many thought once the kid grow up he will bring income. It is changing now, parents no more wants to send their children for work in early age so they are having few children. Spacing between pregnancies also improved because parents are facing the effect of having repeated pregnancies with in short period of time. … Now birth control is available in the nearby health facility and the community can get advice from health professionals” [Ministry of Agriculture representative]*

*“I think the change is in the urban population because now they are having 1 or 2 children. This might be because of economic status of people (now with the inflation and so). There is a relation between this factor and nutrition especially with spacing. If people can feed their children, they can give birth as many as they want but, if you can’t feed and give birth to 3 or 4 and let them be raised as their luck, it will have a negative impact. There are some areas where they don’t care about nutrition and they will have babies thinking they will grow up by luck. And in this area rate of family planning is average…”* [Ministry of Health representative]

*Reduction in childhood illness*

According to the national key informants, the magnitude of childhood diseases such as malaria, vaccine preventable diseases, and diarrhea have decreased impressively over time. They also mentioned that a reduction in childhood morbidities has contributed a lot for the observed stunting reduction in the country.

*“… water sector policy, malaria prevention, and the one that I have mentioned earlier regarding communicable disease management have great contribution for stunting reduction. Malaria prevalence have decreased a lot in our country and that has contribution for stunting reduction.”* [Child Health Specialist and Consultant]

Moreover, our respondents indicated there were various programs implemented in the country that had played major roles in reducing childhood morbidities and in turn undernutrition. The programs mentioned included the Malaria Prevention Program, the Integrated Management of Childhood Illnesses, the WASH Program and the Community Based Nutrition Program.

The following extracts describe a few of the programs that have shown to improve child health:

*“Integrated management of new born child illness (IMNCI) has relation with stunting. Diarrhea is the most important cause of stunting. Repeated infection will increase the risk. Beside a good dietary intake, the kid should get medical assistance during illness to be healthy and to have a normal growth. IMNCI, integrated child care management (ICCM) and community based nutrition are essential for survival and stunting reduction. Nutrition education during these times is also essential.” [ iNGO representative]*

*“The other is WASH program. The wash program aids the reduction in stunting by working to provide safe water and this will reduce the risks of acquiring parasitic infections that consumes micronutrients. “[WASH expert]*

*Child Characteristics*

Child characteristics including birth weight and intergenerational factors were not highlighted by national key informants as contributing to stunting declines among children in Ethiopia.

**Regional Stakeholder Perspectives**

In-depth interviews were conducted with 12 sub-national key informants across the two regions and four districts. Those interviewed included teachers and health staff (health extension workers, maternal, newborn and child health care workers, district health surveillance focal persons, and senior health centre staff) (Table 16).

## **Supplementary Appendix Table 11:** Summary of Regional Stakeholders

| **Participant #** | **Organization** |
| --- | --- |
| Aware participant 1 | Medical director, Health center |
| Aware participant 2 | Laboratory technician, Health center |
| Aware participant 3 | Medical director, Health center |
| Harshini participant 1 | District surveillance focal |
| Harshini participant 2 | District EPI focal |
| Harshini participant 3 | Nurse, Health center |
| Bonga participant 1 | Health extension worker |
| Bonga participant 2 | District Health extension program focal |
| Bonga participant 3 | District Program officer |
| Yeki participant 1 | Crops protection professional |
| Yeki participant 2 | Medical director, Health center |
| Yeki participant 3 | School teacher |

**Basic Drivers of Stunting Decline in Ethiopia**

*Social Political Context*

Regional key informants mentioned that the absence of peace and security was a major challenge and a barrier for people to move from one place to the other. This was a larger concern in the Somali region compared to the SNNP region. The improved security status in the last five years now allowed residents to access markets without fear and helped to reach malnourished children earlier.

“*Five years ago, things were quite different due to insecurities. People had no access to free trade and there were fear and it was difficult to reach the needy due to inaccessibility to some places. For us to reach Jigjiga it used to be a three-day trek but now it a half day trek. You can imagine the difference. Now there are many roads that connect Aware to different parts of the region and any one can move from one place to another without fear or military escort. This has resulted to reach more malnourished children who would otherwise die of it*.” [Health worker, Somali region]

*Improvements in Education & Women’s Empowerment*

Key informants discussed education in terms of increased access to school, increased number of educated people, better economic status of educated people, and a change in curriculum. The key informants indicated that there are considerable improvements in access to both elementary and high schools leading to increased number of educated people in the community. The informants also indicated that those with more education have a better economic status compared to uneducated ones.

*“Lots of things have changed! First people are now educated. Every mother or every household has at least one educated child or person. It’s very rare to find a household without one educated person today.”* [Medical Director, Somali Region]

Across respondents, there has been a remarkable growth in the proportion of female students in schools. Regional key informants indicated that the community did not encourage female education and many were not willing to send their daughters to school. This has changed dramatically and now the number of female students was comparable and even sometimes higher than male students. However, concerns were raised in terms of the number of dropouts of female students at higher-grade levels. Respondents indicated that a lack of familial support, early marriage, and long distances to schools are the main reasons for the drop out of female students at higher grades, especially from high schools. However, many of the respondents discussed the positive role of female education including better childcare, prevention of malnutrition, and promoting health.

The following extracts illustrate how rates of female education had changed:

*“Regarding female education, previously communities did not believe that girls could learn something as boys. People used to criticize girls’ education. For instance, one class there may be two female students in those days. My class in my school time from grade four to eight there were only five female students. However, now girls’ education improved. I can say that nowadays girls are better in schools than boys. The number of girls in schools is higher than boys. I believe educating girls would help the prevention of malnutrition. School children pass messages regarding health to their mothers in the home. Educated mothers would properly care their children because they know practices that lead their children’s health problems. I can say health of children is in the hands of the mothers. If mothers’ practice good behaviors, such keeping the hygiene and sanitation of their children and giving their children balanced diets children would enjoy good health.”* [Health worker, Somali region]

Although female education has increased, the following quote demonstrates concern regarding the high dropout rate of females:

*“Even though most of females are learning now there is also high rate of drop out from school. This is because women are forced to drop school when they lack someone to support economically. Numbers of women are found to attend school at elementary level but when it goes higher their number becomes reduced because of different reasons. The first one is lack of economic support. The other is distance of schools. It is difficult to go long distance and attend school especially for women.”* [Health worker, Southern region]

*Poverty Reduction*

Key informants discussed that there is a considerable improvement in the economic condition of their respective communities in terms of having better housing, trade, and employment opportunities. The change in the standard of housing from a small hut to a house made of iron and bricks was seen as a sign of economic gain for farmers in the community. The improved agriculture and business opportunity in the locality were perceived to be the key factor for the economic improvement. In addition, more employment opportunities were believed to have improved food security and nutrition in the locality.

The following extracts illustrate how living conditions have changed:

*“I think there are many changes after 15 years. For example, the farmer who used to live in a small hut is now living in a house made of tin and bricks. There were illiterate families, but now they are sending their children to school so that they will create educated children. Some farmers also send their children to private schools. Farmers are now opening “wefcho bet”. They are now having improvements. Majority of people 90-95% of people live in a tin house. there is an improvement of the farmers in this district and it can be said it is the result of the agriculture. Nevertheless, when you look around, there might be poor farmers. These are farmers who came from another area recently and living in a small hut.”* [Agriculture expert, Southern region]

*“If we also see the civil servants, the number of people now working for the government have increased and if you compare last year and this year the number have increased tremendously and we have almost 100 new persons employed. All these employed people will in one way or the other improve the food security in their respective household which will in turn improves the nutritional status of children.”* [Health professional, Somali region]

*Nutrition-Specific and –Sensitive Policies and Programs*

Key informants cited various nutrition -specific and -sensitive programs that were implemented in the community. These include the HEP, MERCY CORPS, one WASH, ENGINE, PSNP, and the Pastoralist Community Development Program (PCDP). Key informants emphasized substantial improvements in the health and nutrition of their respective communities after the start of the HEP. They mentioned that among the 18 health extension packages, 85% of the activities are on communicable diseases. Nutrition, hygiene and sanitation, and maternal and child health services were the major focuses of the HEP. They have indicated that the HEP was successful in implementing programs such as vaccination, family planning, nutrition, promotion of hygiene and sanitation, latrine construction and other maternal and child health services. Health education was mentioned as the most important strategy to achieve these goals. Key informants mentioned resistance to change by the community as a key challenge to the HEP. This resistance was rooted in the poor awareness regarding the importance of services and the misconception that health extension workers were paid directly by the community, which generated suspicions about the services provided by health extension workers.

The following extracts elaborate about the role of the HEP in improving maternal and child health services at the grass root level:

“*Before the start of the health extension program, there was huge challenge on maternal and child health in this community. Mothers bleed because of delays and also many other problems which negatively affected the women health. The health of the child too was jeopardized before the commencement of the health extension program.”* [Health worker, SNNPR]

*“Regarding nutrition, now the mothers are appropriately feeding themselves and their children. If possible, they prepare food from what is available at home. At times they sell what they produce and buy from the market what they don’t have. Previously once they drink coffee in the morning, they won’t take any other thing after that till night. Now the after a serious of health educations there are changes. At least mother eats now every 6 hours and 3 times a day. In addition, they might also diversify it from different sources”* [Health worker, SNNPR]

The Pastoralist Community Development Program (PCDP) was elicited as one of the programs that contributed to stunting reduction by building schools, animal and human health posts, and water dams for the community. Key informants also indicated the role of the ONE WASH program in terms of improving hygiene and sanitation in their communities. The reflection on the one WASH program indicates the successes in improving coverage and the challenges in terms of not reaching the entire community.

*“ …They (WASH) have a good name and a great plan but it is not up to our expectation. They dug few toilets for the governmental institutions but they have not dug toilets for the community…* They were implementing most of the WASH programs. They used to *provide hand washing facilities to the community with the demonstration of how to do. They used to teach and post pictures of how many times a mother needs to wash her hands while caring for a child. You can see their posters in this health center yourself.* [Community KI, Somali region]

**Underlying Causes**

*Access to Health Services*

Key informants indicated that there were substantial improvements in access to health services due to the massive construction of health centres and health posts. In addition, the key informants indicated there was an increase in the health workforce. A key informant mentioned that this remarkable change in access to health services is due to the country’s high-level policies and programs. However, respondents indicated that there was still an inadequate health workforce especially for nutrition services. In addition, they reported that there was a lack of ownership, training, and emphasis regarding nutrition services in the health facility.

The following extracts from health workers illustrate the increased availability of health facilities and services at the community level:

“*The availability and accessibility of health services were limited before twenty years. It was difficult for the community to access basic health services. This was due to the absence of health infrastructure and the insecurity that disrupted the availability of basic health services. Before twenty years, the woreda had one clinic and currently the district has three health centers and fifteen health posts. The number of health workers increased compared with previous years.*” [Health worker, Somali region)

“*We only had once health center in this Woreda back in the days, but now we have 3 or 4 health centers. There is also health post that has changed to health center and health center that has changed to hospital. There was no hospital in Tepi but we have one hospital there now. There are also different health posts in each kebele, which has improved access to contraceptives. Overall there is great improvement in relation to health facility*.” [Health worker, SNNPR]

*Water, Sanitation & Hygiene*

Key informants indicated an improvement in latrine coverage over time. They also mentioned that there has been a substantial reduction of open defecation. However, they indicated that there is still a gap in the number, quality, and utilization of latrines. They also highlighted the significant contribution of the HEP in improving coverage and utilization of latrines*.*

The following extracts illustrate these ideas:

*“People didn’t use toilet facilities back then. They used to defecate on open fields or in the bush. However, this has changed and people have their own toilets in their houses now. There is no one without toilet facility. They even dispose their children’s call of nature using the toilet. They are also aware of how to wash their hands after using of toilets. The toilet coverage is more than 90 % now. Except those areas with the conflict that has a gap on implementation there is good coverage of toilets.”* [Health worker, SNNPR]

“*Everyone has toilet facility in their house but the quality is poor. It took us 5 years to make them dig hole for toilet because they don’t think that toilet is important for them. And it will take a lot to make them build toilet with good quality. There are only few people who understood the benefit so they constructed the toilet very well. However, a lot is expected so that the community understands the benefit of toilet.”* [Health worker, SNNPR]

“*All of the toilets that we have managed to construct through the community is not even 15% and the toilets constructed of poor quality and there are no slabs and most of them smell bad and these resulted people not to use the toilets regularly.”[*Health worker, Somali Region]

Key informants indicated that there have been some improvements in access to safe water over time. However, they mentioned that there is still a significant gap in the provision of safe drinking water for the community as many people are still using river water without any treatment.

The following extracts illustrate these points:

*“Years back, water shortages were common. Communities used to fetch water from long distances. The people used to go a place called Bulale to fetch water. Now there is improvement in accessing water. Now there are dams, Birkas and wells that were constructed by the government and private individuals. The changes in governance system and the restoration of peace in the district have resulted in improvement of the availability of water.”* [Health worker, Somali Region]

*“In terms of access to clean water, there is no much improvement. There is no clean water. I cannot say there is no water at all. On areas where there is Action Aid project there is water investment and they are building pipe water. But if we count the water sources in a village there are only 3 or 4 improved water sources.”* [Health worker, SNNPR]

*“Most people use spring water, and some use river water. Sometimes people are affected by Giardia and other water borne diseases. There is challenge with water supply in this kebele, they are working to improve the supply but now we face challenge in having access to safe water supply.”* [School teacher, SNNPR]

*Food Security*

Key informants indicated that there have been substantial improvements in food security in their communities. The observed improvement in food security was related to the increased agricultural productions as farmers started to use better farming practices and agricultural inputs. In addition, improved access to markets, availability of roads, and access to media were identified by key informants as factors in improving food security in their locality.

*“There is no problem regarding having food in this district. People are independent in terms of that. They are now competing about what they can provide for the market. There might be a few farmers who are not able to feed themselves but majority of them are independent.”* [Agriculture Expert, SNNPR]

*“They are now using new methods of farming and start to use fertilizers and improved seeds. This helped them to be more productive and live a better life. There was no road access so that becomes obstacle to sell what they have produced. And people in the nearby didn’t buy them. However, the access is changed and they are able to sell what they produce with good amount of money now.”* [Health worker, SNNPR]

*“It has improved a lot. Back then, they didn’t get such information because they only get the information from agricultural professionals. Now, the farmer uses media like television and get different information. Now they are getting information from different directions. There are farmers who live in doubt and don’t even want to know, but when we see it generally, I say it is improved from 15 years back. The hope is that this kind of farmers will change when they see other farmers improve in economy and will want to be like them and change*.” [Agriculture expert, SNNPR]

Although there is improvement in food security, key informants indicated that the market price of food items had increased overtime and this might decrease financial access to food. Key informants also stated that due to the increase in population size, the per capita farmland size was decreasing resulting in a small land size for one person to farm. Moreover, farmers were now shifting to farming cash crops and this could result in a decreased production and availability of food.

*“The price of food is higher now compared to previous years. Previously people used to sell one goat and it was sufficient for the food of two households. However now money of one goat covers only two items of food, which is not adequate even for one household. Despite the high price of food, the accessibility of food is better now because of the modern transportation. Previously people used to transport foods from the town with their animals as a vehicle.”* [Community KI, SNNPR]

*“Back then, the farmers had wide land. Now that the generation increased, the land is getting smaller and smaller so I will tell you openly that there is a land shortage in our district now. Majority of people are farmers so the land is divided and there is only a small land for one person to farm.”* [Agriculture Expert, SNNPR]

**Immediate Causes**

*Dietary Intake*

Key informants perceived that the quality of food given to children was better years back (no period specified) compared to the present. Informants highlighted that production itself had not decreased but access to animal source foods had decreased due to costs of purchasing these items. Increased market access had also resulted in farmers selling products that would have normally been primarily for their own consumption. Despite the perceived limited access to animal source foods, key informants indicated that the diversity of children’s diets has improved over time. This was mainly attributed to the improved availability of agricultural products in their community. Moreover, key informants indicated that the expansion and improved access of health services and the education given by the health extension workers have improved the knowledge of mothers to diversify diets of children.

The following extracts illustrate the issues regarding animal source foods, market access, and diet diversity:

*“People used to consume the products of their animals such as meat, milk and butter however now people do not use animal products. People used to eat locally available foods such as maize and sorghum but now they use pasta, rice and flour. Now people sell milk and butter and in exchange, they take pasta, rice, oil and sugar. People used to cultivate farms before twenty years but the last fifteen years people abandoned cultivation of land due recurrent droughts and persistent conflict, which affected the availability of food.”* [Community key informant, Somali region]

*“.... Back then as I mentioned people used to drink milk only but now the milk they used to depend on is not even available. So naturally when something gets out from somewhere something else gets in. So, food has replaced the milk which people used to depend on back then. Previously, it was very rare to see trucks with exception of government vehicles. Now many vehicles arrive in Aware each day and mostly these tracks carry food and vegetable that are meant for business. They are bringing food, vegetables and other needs for the district. For that reasons availability of food has increased so far*.” [Community key informant, Somali region]

*“Previously the dietary intake of people was poor. People used to eat foods that did not contain enough nutrients. People used to eat the same type of foods such as meat, butter maize and sorghum. Mothers and others adults could survive this type of foods however; children did not receive nutritious diets in previous years. Children need different kinds of foods such as vegetables, fruits, rice pasta fish and milk. Nowadays people consume various diets that is rich in many nutrients. Dietary intake is better now than previous years because of the availability of many different foods. Previously people did not know vitamins. The availability of vitamins was limited to towns. Majority of the people lived in rural areas. Before twenty years health facilities did not exist in the rural areas. However, people now get vitamins and others nutrients due to easy access of all types of health services everywhere.”* [Community informant, Somali region]

*Infant and Young Child Feeding*

Key informants perceived that there had been significant improvements in breastfeeding pattern such as initiation of breastfeeding, colostrum feeding, prelacteal feeding as well as frequency and duration of breast-feeding. However, key informants also mentioned that there are problems regarding mixed feeding and delay in the initiation of complementary feeding.

*“Yes there is change. In the past when a child was born the colostrum was discarded and the baby was not allowed to breastfeed that. They were unaware about the health benefit of it. Now immediately they gave birth they will start breastfeeding. There is no colostrum discarding. On the frequency of the breastfeeding, also there is a change. Previously once they left the house they will be back after long time. But now they feed at least 10 or 12 times. They frequently breastfeed. So they have understood the health education we gave them on immediately breastfeeding and they are applying it.”*[Health worker, SNNPR]

*“The main problems related to nutrition and dietary intake of children includes mixed feeding because mothers feed their children food or milk before the age of complementary feeding in addition to breast milk. Mixed feeding causes health problem to the child.*” [Community informant, Somali region]

*Dietary Recommendations*

Key informants indicated that increased access to health care and increased availability of health workers considerably changed dietary recommendations including optimal breastfeeding, complementary feeding, WASH etc. Before the expansion of health services, the elderly from the community were the ones providing counseling regarding infant and young child feeding as well as nutrition during pregnancy.

*“Back then, there was no good functioning health system because the number of health facilities and health workers were limited. Mothers do not come to health facilities searching for information regarding nutrition of their children. They obtain information from the society especially the older people and health workers when children got sick.”* [Community informant, SNNPR]

Key informants indicated that an increase in health education combined with the advice health workers now provide to mothers positively influence the dietary intake of children.

*“We advise mothers to give care to their children. We also advise mothers to feed their children with balanced diets containing every nutrient that children require for better growth. We recommend mothers to feed their children with various foods. We also tell mothers to feed their children every type of food the child can eat. We advise mother to feed their children healthy and hygienic foods. Health workers also recommend mothers to keep the hygiene and sanitation of their environment. Health workers recommend mothers to continue giving their children the breast milk until two years in addition to the supplementary foods.”* [Community informant, SNNPR]

*“After the health professionals start educating the community widely, the education also included the negative effect of introducing other food before the appropriate age. The inappropriate feeding might lead to malnutrition. Even their gastric is not mature enough to handle the food at that age. There might be also other concomitant diseases. After we came here, we started screening for the malnourished kids using MUAC. After screening, we also started referring them, so that they will get further treatment. At health center or hospital, they will get treatments like formula milk then they will get better. And those who have vegetables garden at their backyard will appropriately feed their children. Those who take our counseling seriously are improving, their family and their children too.”*[Health worker, SNNPR]

*“There is better counseling ever since we started implementing focused antenatal care. We used to just check her weight and tell her that she needs to maintain her weight and that she shouldn’t get hungry. But now we counsel her about the type of food she needs to eat and how many times a day she needs to eat.” [Health worker, SNNPR]*

However, they also mentioned that poor families could not afford to give a balanced diet to their children even after they understood its benefits.

*“back in the days there used to be parents who did not properly utilize food because of lack of knowledge despite having access and thus their children were affected by malnutrition. And there are also parents with nothing to provide their children and who can’t do nothing about it. You can counsel and change those parents with poor knowledge because they have access the challenge is for the parents who did not have anything. There is nothing we can do for them. So these days you only see malnutrition among that family who don’t have anything. There is no one coming with malnutrition because of poor knowledge as long as they have access to food. This has changed because for example they have milk at home but instead of using it they used to sell it, same is true for egg. But now if you go to the market mothers from rural village will be in line to buy egg just like the rest of us. This indicates change, but there is nothing we can do for those parents who don’t have anything.”* [Health worker, SNNPR]

Despite the improvements in dietary recommendations, they highlight that there was still a need for health workers who provide proper nutrition counseling.

*“There is no nutrition professional. I didn’t see health extension workers teaching about this. There are health extension workers down to the community but they don’t give lessons about nutrition and what to eat. They might give lessons about other health issues but they don’t tell the farmer to eat this and that so that he can be healthy and you can have a full diet if you wat with what you have doing this and this. So I say there is a gap regarding this.”* [Agriculture expert, SNNPR]

*Maternal Factors*

Key informants discussed contraceptive use and its impact on malnutrition. Key informants from the SNNP region perceived that access and choice for family planning services have improved over time. They also indicated substantial improvement in the utilization of contraceptives and hence resulting in better child spacing. However, key informants from the Somali region indicated that there has been no improvements in contraceptive utilization due to religious beliefs.

The following extracts illustrate these points:

*“There is a lot of improvements in this regard. In the past, they used to give birth every other year. At that time they counseling was too low. They were not aware about the different types of family planning methods. Now there is one which is given every 3 month, 3 year. If she decides not to give birth, she can take the one for 3 years and if she changed her mind and wanted to give birth she can have a bay after the 3 years. If she wants to have more space, she can take the one for 5 year. They have all the choice. They use which ever method they want according to their choice. There is much improvement on this.”*[Health worker, SNNPR]

*“A mother will not give birth to a child soon after having another child. if there is spacing a child will not be affected by malnutrition. Back in the days they keep giving birth to the extent where they can’t even remember their name. but now that is no longer the case so family will not stress because of malnutrition. And that is a big advantage.”* [Health worker, SNNPR]

*“They see it as a very bad thing to use contraceptive and the few ones who use, use it in secret and they do not tell anyone else. They say we are Muslims and Allah is going to feed the children and it’s disobeying to space children for the fear of “what they would eat” and “who will feed them”. When we told them it’s not about who will feed them or what they will eat then they say Allah gave it to us it’s something that is out of our control. They also belief if they use contraceptives, they will lose fertility forever. Women who use family planning or other contraceptives to space pregnancy are very rare.”* [Community informant, Somali region]

*Disease*

Key informants indicated there had been a significant decline in childhood communicable diseases such as diarrhea, parasitic infestation, typhus, and skin and eye infection. They indicated that the achievement is made because of improved community awareness, and improved hygiene and sanitation.

*“There were lots of cases of diarrhea as an outbreak. But now there is no diarrhea at all. It was like an epidemic before.”* [Health worker, SNNPR]

*”Typhus used to be an outbreak before but now the numbers of cases are reduced. Cases of diarrhea, typhus, eye infection, scabies and skin infection are reduced in the health centers. This is a change that is brought because of community members become aware and start to keep themselves clean.”* [Health worker, SNNPR]

*“As I was saying before there were some gaps on hygiene and sanitation of children. This is related to abdominal parasites that influence on the child’s appetite and feeding practice. So there was a gap on preventing this parasitic infection through keeping children’s hygiene”* [Health worker, SNNPR]

*“When we summarize people in this are protected from different diseases because of the drinking water and toilet facility. Back then, children were affected by diarrheal disease and different other diseases, but now due to the availability drinking water and toilet facility the burden of these diseases are decreased.”* [Health worker, Somali region]

**Mothers in Communities Perspectives**

We conducted FGDs with mothers of children under-5 born during the three time periods (1987-1991, 1995-1999, 2011-2015) when the country documented substantial stunting reduction. We analyzed the contextual, underlying and immediate factors elicited by community members and compared them across the three time periods to identify the factors that were salient in the time periods. Table 17 depicts a summary comparison of the regions (Somali and SNNP), and mothers with children born in 1987-1991, 1995-1999, and 2011-2015, highlighting key contextual, underlying and immediate factors and trends over time

**Supplementary Appendix Table 12:** Summary and comparison of contextual, underlying and immediate factors elicited across mothers of children born in three time periods, Ethiopia

| **Location** | **Drivers** | **Mothers of children born in 1987-1991** | **Mothers of children born in 1995-1999** | **Mothers of children born in 2011-2015** |
| --- | --- | --- | --- | --- |
| **Somali Region** | **Distal Causes** *(For example: political context/stability/conflict, poverty reduction, education, women’s empowerment, urbanization, labour migration/remittances)* | - Poverty represented a concern - Conflict, instability and insecurity was a concern - Education was not prioritized | - Poverty represented a concern - Conflict, instability and insecurity was a concern - Education was not prioritized - Improved infrastructures such as roads and housing - Women’s income improved | - Poverty and poor living condition remained a concern - Boys and girl’s education improved - Improved infrastructures such as roads and housing - Women’s income improved |
|  | **Basic Factors** *(Nutrition-Specific & -Sensitive Policies & Programs)* | - NA | - NA | NA |
|  | **Underlying Causes** *(e.g., improved feeding practices and food security, improved care and health services, improved household environment/WASH)* | - Drought and water shortage was less of a concern - Food price was not a concern - Animal source foods were accessible - Health service was a concern - Access to safe water supply was a concern - Poor hygiene and sanitation | - Drought and water shortage was a concern - Price of food was a concern - Animal source food was less accessible - Health service was a concern - Access to safe water supply was a concern - Improved hygiene and sanitation | - Drought and water shortage was a concern - Price of food was a concern - Animal source food was less accessible - Health service access improved - Access to safe water supply was a concern - Improved hygiene and sanitation |
|  | **Immediate Causes** *(e.g., improved dietary intake (infant and young child, dietary diversity, etc.), disease, maternal characteristics (parity, interpregnancy intervals, maternal age, maternal height) and child characteristics (low birthweight)* | - Vaccination and FP service was absent or inaccessible - Childhood illnesses were a concern - Sociocultural practice and taboos on diet | - Improved child health services such as vaccination - Childhood illness of lesser a concern since health service were available nearby - Suboptimal feeding was a concern | - Improved child health services such as vaccination - Childhood illness of lesser a concern since health service were available nearby - Suboptimal feeding was a concern |
|  |  |  |  |  |
| **SNNPR** | **Distal Causes** | - Women and girls education was not a priority in families - Poor infrastructure - No role of women in decision making | - Boys and girl’s education improved - Improved infrastructures such as roads and electricity - Poor role of women in decision making | - Boys and girl’s education improved - Improved infrastructures such as roads and electricity - Improved role of women in decision making |
|  | **Basic Factors (Policies & Programs)** | - NA | - NA | - NA |
|  | **Underlying Causes** | - Child care was less of a concern/priority - Less use of agricultural supplies - Poor health service access - Access to safe water supply was a concern - Poor hygiene and sanitation | - Child care was less of a concern/priority - Improved crop production, supplies and methods - Health service access improved - Access to safe water supply was a concern - Improved hygiene and sanitation | - Improved child care - Improved crop production, supplies and methods - Health service access improved - Access to safe water supply remains a concern - Improved hygiene and sanitation |
|  | **Immediate Causes** | - Vaccination and FP service were absent or inaccessible - Childhood illnesses were a concern - Diet rich in animal source | - Improved child health services such as vaccination - Childhood illness of lesser a concern since health service were available nearby - Fruits and vegetables access improved - Child care improved - Suboptimal feeding was concern | - Improved child health services such as vaccination - Childhood illness of lesser a concern since health service were available nearby - Fruits and vegetables access improved - Child care improved - Suboptimal feeding was concern |

*N/A = Topic/issue was not discussed by mothers in FG*

**Basic Drivers of Stunting Decline in Ethiopia**

*Poverty*

FGD participants identified poverty, peace and security, and women empowerment and education as the most prominent contextual/distal factors. Mothers in the Somali region, across the different year groups, indicated that poverty remains the most salient factor. The discussants acknowledged the big role government support programs such as the PSNP had on vulnerable segments of the population. In addition, mothers in the SNNP region with children born in 1995-1999 and 2011-2015 noted some improvements in living conditions.

*“Needs of the communities are more. People are poor now and they do not have animals. I came this town when my animals died and I was dependent on the aid of the government.”* [Mother FGD Aware, Somali, 1987-1991].

*Conflict and Security*

Next to poverty, conflict and insecurity were the most commonly raised factors in both regions and across the year groups. The prevailing conflict between rebels and the government, particularly in Somali regions, directly and indirectly affects the food security situation of the communities. FGD participants indicated that the instability and conflict has influenced nutrition among children in a number of ways. For example, the conflict had obstructed availability of foods in the market, caused decreased crop production, hampered transport, and affected trade between communities.

The following four extracts from the FGD transcripts illustrate these points:

*“…Regarding food access, things have changed over the past 20 years. People were farming but as due to the insecurity and absence of peace the farming was halted…. Farmers were forced to abandoned their farms and migrated to the villages and towns where they ended up jobless …Currently families started to cultivate lands due to the peace agreement that have been reached between the government and the rebels, Thanks to Allah.”* [Mother FGD Aware, Somali, 1995-1999].

*“Now that there is no insecurity, people are free to go anywhere unlike previous times when we were handicapped. All these different types of vegetables that the lady has mentioned is a result of the peace that has resulted free movement of vehicles. Before there were a lot of fear and there were no vehicles to bring food and the vegetables that were just mentioned. Those days’ people never dreamt of getting vegetable.”* [Mother FGD Aware, Somali, 2011-2015].

*“Even though we cultivate and produce crops, we were not able to sell it since it was not safe. We were not able to buy foods for our children. We were living with fear. But now it has changed and we are living in peace. We are producing foods and selling them on market in order to buy what is not available at home. We are also feeding our children with foods from both what we produce and the market.”* [Mother FGD Yeki, SNNP, 1987-1991].

Moreover, more and more farmers were joining the military away from home and this has left a huge burden on the women to feed their kids.

*“my husband was forced to join the military which I was all alone by the time. I was in stress that I have nothing to feed my children. I was forced to move to my mother’s house so that I could feed them. I was worried and spend terrible time.”* [Mother FGD Yeki, SNNP, 1987-1991].

*Women Empowerment and Education*

The next distal factors elicited during the discussion were related to the improvements in women empowerment and education. FGD participants highlighted that the value towards education in general and girls’ education in particular had improved over time. This attitude change was due to observing the benefits of education, the change in values given to children, and the improved access to schools, infrastructures and transportation availability in the communities. Moreover, the observed empowerment of mothers benefited children in terms of providing appropriate care and nutrition to their children.

*“Previously we were just pastoralists and housewives, we use not work but currently women are working and they have their own source of income and in a position to feed their children. If you work you will be in a position to care for children but if don’t the child may not get the proper care, he/she is supposed to get”.* [Mother FGD Harshin, Somali, 1995-1999].

*“A mother who is educated feed her child with foods of better quality. This is because she is aware and wants her child status to be improved. But those mothers who are not educated feed their children whatever they got and is available at home.”* [Mother FGD Yeki, SNNP, 1987-1991].

In addition, FGD participants indicated that the role of women in the household and their earnings, and productivity has improved over the three periods (1987-1991, 1995-1999, and 2011-2015).

*“Our view of girl education is not the same now and then. We used to think that a girl that goes out early to learn was a bad girl. Our cattle’s and our farms were of more value to us than education. We did not even know what education was. Now that we have seen the results of education, and I think is too late to realize it. We have seen how others surpassed us while we wronged our female children. We sent them to do household chores such as grinding maize while boys went to learn. No we copy the good we saw others do. Now we educate both genders equally. Salaam alaykum”* [Mother FGD Aware, Somali, 1995-1999].

*“He used to do whatever he wanted with the money, but now I am working too; so we both have to discuss and decide when buying things and it is better now.”* [Mother FGD Yeki, SNNP, 1987-1991].

**Underlying Causes**

FGD participants identified environmental shocks (drought), access to health services, food insecurity, and access to safe water as the most prominent underlying factors. Drought is a major concern particularly in the Somali region while the high market price of foods remain important challenges in both the Somali and the SNNP region.

*Food Security & Feeding Practices*

The women highlighted a complex pattern of food insecurity in their communities over the time period. In particular, for communities in the Somali region, the earlier period (1987-1991) was ideal for women in terms of availability of “*good food*.” The availability of good foods such as meat, milk and butter was better before than the present. Most attributed this change in availability due to the recurrent drought the Somali region had faced in the recent years leading to the death of livestock in the communities. However, in the same communities, the availability of fruits, vegetables, and other crops had improved in the recent years because of the improved peace and stability, transportation system, and accessible markets.

*“Children had better growth in previous years because we used to feed them with grounded maize (cooked maize flour), camel’s milk, and butter. Now it is different from previous years. Because communities do not have enough animals and income for better growth of children.”*

*”* [Mother FGD Aware, Somali, 1987-1991].

*“…There is change in climate because it is much hotter than before, the number of animals has also decreased, and decreased access to nutritious food. So, the cause is climate change…”* [Mother FGD Harshin, Somali, 1995-1999].

*“The main reasons for the changes in children’s nutrition are drought, environmental changes that resulted in death of animals.”* [Mother FGD Harshin, Somali, 1987-1991]

For communities in the SNNP region and across the groups, FGD participants highlighted an overall improvement in agricultural production due to better farming practices such as increased fertilizer use. Moreover, women affirmed an improvement in the availability and physical accessibility of diverse agricultural produces. The major bottleneck for these communities was linked to the imbalance between the selling prices of their produces and buying prices of commodities that they do not produce including crops, fruits, and vegetables.

*“There is an improvement related to the farming method. I am a woman and work on back yard vegetables. Back then we were just plant the crops by scattering but now we plant them in more organized way. We plant using by the methods that the agriculture sector persons show us. So I could say there is a change.”* [Mother FGD Bonga, SNNP, 1995-1999].

*“… although you sell it like that, one kilogram “teff” is now 25 birr. Even if we sell the corn at 800 birr per quintal, we can’t even buy 50 kilograms of teff with the money. Calculate the price with 25 birr per kilogram. If I sell my corn with 600 birr, it can’t even buy me 25 kg of teff. There is price inflation; what we buy is expensive…”* [Mother FGD Yeki, SNNP, 1995-1999].

*Improved Access to Health Services*

Across all regions and groups, women reported considerable improvements in health service availability and accessibility in their communities over time. We documented distinct service utilization differences among the mothers with children born in 1987-1991, 1995-1999, and 2011-2015. The participants indicated that due to the unavailability of maternal health services, mothers with children born in 1987-1991 were delivering at home with the help of mother in laws and/or traditional birth attendants, and had poor antenatal care follow-up, immunization, and contraceptive services compared to the mothers with children born in 1995-1999 and 2011-2015.

*“Despite the financial problems, ability to access care at health facilities now is much better than back then. Back then, all rural mothers were in the bush and did not have access to health care services. Even those in the towns were not that much urbanized; their life style was almost similar to that of the rural”* [Mother FGD Harshin, Somali, 1987-1991].

*“When I was in labour [my son Mohammed], help is from Allah, I lived in a rural place and the Traditional Birth Attendant assisted me. I raised him with the culture of the rural people. I raised him up with animal products. I have never visited a healthy facility during my pregnancies. I had learnt the skills from the traditional birth attendant and she never used gloves. She (the TBA women) sits under the women and delivers the women with the help of Allah. In addition, the baby gets out, we were all familiar with the traditional birth attendant and we used to call her for every delivery*.” [Mother FGD Aware, Somali, 1995-1999].

*“My children are eight in number and all were delivered in my home except the last child, who is delivered at the health center. I was given injection that stops bleeding and given good care.”* [Mother FGD Harshin, Somali, 2011-2015].

The participants indicated that the improved health service expansion has brought more services and benefits to the communities. Women indicated that the health extension workers provided key messages regarding the importance of a diversified diet, exclusive breastfeeding, personal and environmental hygiene, backyard gardening of fruits and vegetables, as well as notable services including bed net provision, and malaria prevention education and outbreaks control.

*“Back then we used to suffer. Mothers used to die on child birth, we used to bleed. But now we get vaccination, our children are getting vaccination. We get regular checkups and vaccination during pregnancy. We used to suffer a lot. But now we are getting benefits. children that are born currently are way better than those born before*.”

[Mother FGD Bonga, SNNP, 1995-1999].

*Improved Household Environment: Water, Sanitation & Hygiene*

Mothers in both regions and across the focus groups indicated that access to safe water supply remained the most derailing experience for the communities over two decades. Although women reported some improvements in parts of the respective regions where the government was drilling ground water, there still remained a huge gap in terms of safe water reaching the majority of the population. Safe water scarcity was exacerbated especially during the dry season and left a heavy burden on the women who were responsible for this chore.

*“Water availability is very poor in Aware, although a borehole was drilled; now drinking from that borehole. The borehole water is clean compared to the birka water (water collected from rain) which is not suitable for drinking and actually we don’t drink birka water instead we use it for washing clothes and other house chores.”* . [Mother FGD Aware, Somali, 1995-1999].

*“there is no difference in water supply, we have always used river water. We used to also use spring water. Now we use rain water and people who have young children will send them to fetch water from another place…”* [Mother FGD Bonga, SNNP, 1987-1991].

With respect to hygiene and sanitation, FGD participants indicated that there had been considerable improvements in the ownership and use of toilets and a reduction of open defecation. However, some women argued that not all community members owned and utilized toilets and there was a difference in capacity to construct one’s own toilet. Therefore, these households tended to either share a toilet or continued to use open field. Women also indicated that a poor waste disposal system remained a concern in the communities.

*“People started using latrines for the last fifteen years. Now you do not observe feces in open environments which is something different from previous years. Years back open defecation was common and the feces were scattered everywhere like animal feces.”* [Mother FGD Aware, Somali, 1987-1991].

*“Very few people have toilets, people use open air, when I say open air, I mean there are at least some bushes where you can hide and that is where people help themselves. I prefer open air because the toilets smell bad. People cannot afford to dig toilets and some are forced to use open fields or share with those have toilets. A person with toilet is someone who has money and has ability to construct it and for those who cannot afford they use open air to help themselves.”* [Mother FGD Aware, Somali, 2011-2015].

**Immediate Causes**

*Dietary Intake and Feeding Behavior*

Breastfeeding was a common cultural practice in both regions and across the groups. In addition, women indicated that milk was an important core IYC diet in both regions. However, there was suboptimal breastfeeding and complementary feeding practises that were still a concern in both regions and across the groups.

Women perceived that the quality of food given to children was better years back compared to the present. This was attributed to the unavailability of animal source foods such as milk, meat, butter, and other animal products in the market. Moreover, women indicated that household farming was reduced due to the recurrent drought and as a result, the production of milk and animal products had decreased over time. For these reasons, the market price of milk and animal products was a current challenge.

Women also indicated that there is a difference in nurturing children in the past compared to the present. Mothers with children born in 1987-1991 indicated that the children used to be “*strong*” and that time was characterized by plenty of meat, milk, and butter compared to the present time where the focus is on childcare, hygiene and children follow-ups.

*“I bought up my child in a good manner. I had camel and goats. We used to eat meat and drink milk and consume butter. Now children are weaker they do not drink milk.*..” [Mother FGD Aware, Somali, 1987-1991]

*“First I want to thank God for getting us to this day. There is a big difference between now and then. Back then we wouldn’t wash the children when they were born; but now we wash them. We are also immunizing them. There is a change.”* [Mother FGD Bonga, Somali, 1995-1999]

Sociocultural issues such as giving “holy water” to the child before initiating breastfeeding continued to be practiced. Boiled water, milk, sugar, honey and butter were also culturally important foods given before initiating breastfeeding.

*“I gave my child holy water and sugar immediately after birth. I gave him cow’s milk after holy water. The next morning, I breast-fed my child.”* [Mother FGD Aware, Somali, 1987-1991].

*“I gave holy water to Safiya first and then breast milk. My breasts had plenty of milk. Tahlil (holy water) or Ashar is the Quran will help to have good understanding and tender to his parents. We did Adhan (prayer call) as well. We still do Adhan. We expect good from Allah. That’s why we give them tahlil.”* [Mother FGD Harshin, Somali, 1987-1991].

*“When my son Mohamed was born, we gave him holy water since the breasts were empty. Religiously, prayer calls should be done in the newborns’ ears but giving the holly water immediately after birth is part of our culture. I gave him boiled water with sugar and camel milk for the 1st three days because he could not get enough of the breast milk after birth.”* [Mother FGD Aware, Somali, 1995-1999].

FGD participants also elicited a number of foods that are culturally not given, children used to be forbidden to eat uncooked foods or raw fruits. some individuals continue to restrict the consumption of these foods by children.

The following are some extracts that illustrate the above points:

*“there was a fear cold and raw food to cause abdominal cramp. Because of these we didn’t feed our children these foods. But after we got the knowledge we start to feed mangos that are healthy.”* [Mother FGD Yeki, SNNP, 1987-1991].

*“mango was one of the fruits that were restricted. It was believed a child might get sick if he/she eats mango so it was restricted.”* [Mother FGD Yeki, SNNP, 1987-1991].

*“injera is not allowed for children less one years of age. Unless a child is able to pee by him or herself, it is not allowed to feed injera. It is better to give pitta, gruel and porridge.”* [Mother FGD Bonga, SNNP, 1987-1991].

The expansion of health services combined with health education provided by the extension program had contributed to the reduced misconception related to culturally “*inappropriate diets*”. Women also indicated the health education they received from the HEP has helped them start the production of fruits and vegetables in the communities.

*“Back then, we used to commonly feed kale to our children but now we are feeding them “metin” (a gruel made of different cereals). Now we breastfeed them until they are 6 months old and give them “mitin” after that.”* [Mother FGD Bonga, SNNP, 2011-2015].

*Child Characteristics and Disease*

The FGD participants indicated that the burden of childhood diseases has declined in their communities over time and this was attributed to the increased availability of health services. Mothers with children born in 1995-1999 and 2011-2015 reported that their children received vaccination services, and had improved access to care and treatment at a nearby health facility. Although children born in 1987-1991 faced similar childhood diseases, the mothers were not able to take their kids to a health facility as the health facilities were either very far or not available in their respective communities. Therefore, these mothers heavily depended on traditional healers.

Across the focus groups and regions, mothers elicited common diseases and health conditions such as diarrhoea, vomiting, skin problems, and malnutrition that affected their children. Mothers attributed this problem to the unavailability of vaccination services in their communities and poor feeding practices.

*“The children I had before the change used to get sick a lot because of malnutrition*

*and other diseases. But the children I have after change are very fine. Even if they get sick we take them to health centers, we give them medicine on time. Even the disease does not occur now.”*[Mother FGD Bonga, SNNP, 1995-1999].

*“there is a lot of improvement, our children are healthier. Children in the past used to get sick a lot but now our children are healthy thanks to God. Most people are educated now, there is health center nearby so we get different counseling. Everyone is getting education and counselling so children are getting different fruits and different types of foods…”* [Mother FGD Yeki, SNNP, 2011-2015].

*Maternal Characteristics*

Maternal characteristics including maternal age, height, fertility and inter pregnancy spacing were not discussed by community level respondents as factors that have contributed to improved nutrition.

**Conclusion**

This qualitative analysis highlighted key contextual/distal, policy/program efforts, underlying and immediate causes of stunting decline according to national and community level respondents.

At the national level, the overall improvement in economy, expansion of health services, improved access to education, improved women and girl’s education and empowerment, and improved agricultural production were the most prominent factors that have contributed to the stunting reduction in the country. In addition, the introduction of the Productive Safety Net Program as well as the Health Extension Program contributed a lot to the observed stunting reduction in the country.

At the regional level, respondents identified improvements in access to health services, education, market access to foods, and peace and stability as important causes for improved nutrition.

Mothers in communities identified reduction of poverty, peace and security, and women’s empowerment and education as the most prominent contextual/distal factors. Women also identified environmental shocks (drought), improved access to health services, and food security the most prominent underlying factors while the decline in the burden of childhood diseases and improved child care practices as the key immediate causes in stunting decline. However, mothers indicated that drought, high market prices of foods, and access to safe water remain the most important challenges in both regions.

# **Supplementary Appendix References**

1 Fantay Gebru K, Mekonnen Haileselassie W, Haftom Temesgen A, *et al.* Determinants of stunting among under-five children in Ethiopia: a multilevel mixed-effects analysis of 2016 Ethiopian demographic and health survey data. *BMC Pediatr* 2019;**19**:176. doi:10.1186/s12887-019-1545-0

2 Desalegn BB, Lambert C, Riedel S, *et al.* Feeding Practices and Undernutrition in 6-23-Month-Old Children of Orthodox Christian Mothers in Rural Tigray, Ethiopia: Longitudinal Study. *Nutrients* 2019;**11**. doi:10.3390/nu11010138

3 Tadele Wuneh M. Nutritional Status and Feeding Practice of Children 6-59 Months Old, Metekele Zone of Benishangul-Gumuz Region, Northwest Ethiopia. *Sci J Clin Med* 2018;**6**:120. doi:10.11648/j.sjcm.20170606.15

4 Bemnet A, Beyene M, Bereket F, *et al.* Micronutrient levels and nutritional status of school children living in Northwest Ethiopia. *Nutr J* 2012;**11**.http://www.nutritionj.com/content/pdf/1475-2891-11-108.pdf

5 Hagos S, Hailemariam D, WoldeHanna T, *et al.* Spatial heterogeneity and risk factors for stunting among children under age five in Ethiopia: A Bayesian geo-statistical model. *PLoS One* 2017;**12**. doi:10.1371/journal.pone.0170785

6 Biadgilign S, Shumetie A, Yesigat H. Does Economic Growth Reduce Childhood Undernutrition in Ethiopia? *PLoS One* 2016;**11**:e0160050. doi:10.1371/journal.pone.0160050

7 Biadgilign S, Ayenew HY, Shumetie A, *et al.* Good governance, public health expenditures, urbanization and child undernutrition Nexus in Ethiopia: an ecological analysis. *BMC Health Serv Res* 2019;**19**:40. doi:10.1186/s12913-018-3822-2

8 Molla Birhanu M. Systematic Reviews of Prevalence and Associated Factors of Under Five Malnutrition in Ethiopia: Finding the Evidence. *Int J Nutr Food Sci* 2015;**4**:459. doi:10.11648/j.ijnfs.20150404.17

9 Headey D. An analysis of trends and determinants of child undernutrition in Ethiopia, 2000-2011. 2014. http://ebrary.ifpri.org/utils/getfile/collection/p15738coll2/id/128896/filename/129107.pdf (accessed 8 Feb 2019).

10 Ngure FM, Humphrey JH, Menon P, *et al.* Environmental hygiene, food safety and growth in less than five year old children in zimbabwe and ethiopia. *FASEB J* 2013;**27**.http://www.fasebj.org/cgi/content/meeting_abstract/27/1_MeetingAbstracts/243.2?sid=3c9bae8a-2977-4617-92ce-fdebd9b96606 http://ovidsp.ovid.com/ovidweb.cgi?T=JS&PAGE=reference&D=emed14&NEWS=N&AN=71153096

11 Demewoz H, Muluken A, Tegegn M, *et al.* Exploring spatial variations and factors associated with childhood stunting in Ethiopia: spatial and multilevel analysis. *BMC Pediatr* 2016;**16**.http://bmcpediatr.biomedcentral.com/articles/10.1186/s12887-016-0587-9

12 Tadesse M, Getabil F. Factors Associated with Stunting of Under-Five Children in Ethiopia. *Stat Appl* 2016;**14**:145–58.

13 Gurmu E, Etana D. Household structure and children’s nutritional status in Ethiopia. *Genus* 2013;**69**:113–30.https://www.scopus.com/inward/record.uri?eid=2-s2.0-84886071880&partnerID=40&md5=cacb28552f7219f8586718714e75bee4

14 Dessie ZB, Fentie M, Abebe Z, *et al.* Maternal characteristics and nutritional status among 6-59 months of children in Ethiopia: Further analysis of demographic and health survey. *BMC Pediatr* 2019;**19**:1–10. doi:10.1186/s12887-019-1459-x

15 Hussien S, Id M, Dejenie Habtewold T, *et al.* Dietary and non-dietary determinants of linear growth status of infants and young children in Ethiopia: Hierarchical regression analysis. *PLoS One* 2019;**14**. doi:10.1371/journal.pone.0209220

16 Woldemariam G, Genebo T. Determinants of the Nutritional Status of Mothers and Children in Ethiopia. Calverton, Maryland USA: 2002. doi:10.1016/j.ijhcs.2005.06.004

17 Seifu H, Torleif L, Mariam DH, *et al.* Climate change, crop production and child under nutrition in Ethiopia; a longitudinal panel study. *BMC Public Health* 2014;**14**.http://www.biomedcentral.com/content/pdf/1471-2458-14-884.pdf

18 Hirvonen K, Headey D, Golan J, *et al.* Changes in Child Under-Nutrition in Ethiopia, 2000–16. *Oxford Handb Ethiop Econ* 2019;:398–411. doi:10.1093/oxfordhb/9780198814986.013.21

19 Wirth JP, Matji J, Woodruff BA, *et al.* Scale up of nutrition and health programs in Ethiopia and their overlap with reductions in child stunting. *Matern Child Nutr* 2017;**13**:1–17. doi:10.1111/mcn.12318

20 Getahun Z, Urga K, Ganebo T, *et al.* Review of the status of malnutrition and trends in Ethiopia. *Ethiop J Heal Dev* 2001;**15**:55–74.https://www.ajol.info/index.php/ejhd/article/view/83811

21 Motbainor A, Worku A, Kumie A. Stunting is associated with food diversity while wasting with food insecurity among underfive children in East and West Gojjam Zones of Amhara Region, Ethiopia. *PLoS One* 2015;**10**:1–14. doi:10.1371/journal.pone.0133542

22 Woodruff BA, Wirth JP, Bailes A, *et al.* Determinants of stunting reduction in Ethiopia 2000 – 2011. *Matern Child Nutr* 2017;**13**. doi:10.1111/mcn.12307

23 Headey D, Hoddinott J, Park S. Accounting for nutritional changes in six success stories: A regression-decomposition approach. *Glob Food Sec* 2017;**13**:12–20. doi:10.1016/J.GFS.2017.02.003

24 Buisman LR, Van de Poel E, O’Donnell O, *et al.* What explains the fall in child stunting in Sub-Saharan Africa? *SSM - Popul Heal* 2019;**8**:100384. doi:10.1016/j.ssmph.2019.100384

25 Ambel AA, Andrews C, Bakilana AM, *et al.* Examining changes in maternal and child health inequalities in Ethiopia. *Int J Equity Health* Published Online First: 2017. doi:10.1186/s12939-017-0648-1

26 Ambel AA, Andrews C, Bakilana AM, *et al.* Examining changes in maternal and child health inequalities in Ethiopia. *Int J Equity Health* Published Online First: 2017. doi:10.1186/s12939-017-0648-1

27 Silva P. Environmental Factors and Children’s Malnutrition in Ethiopia. 2005. http://econ.worldbank.org. (accessed 8 Feb 2019).

28 Megabiaw, Berihun M, Rahman A. Prevalence and Determinants of Chronic Malnutrition Among Under-5 Children in Ethiopia. *Int J Child Heal Nutr* 2013;**2**:230–6. doi:10.6000/1929-4247.2013.02.03.5

29 Gebreegziabher T, Regassa N. Ethiopia’s high childhood undernutrition explained: Analysis of the prevalence and key correlates based on recent nationally representative data. *Public Health Nutr* 2019;**22**:2099–109. doi:doi:10.1017/S1368980019000569

30 Takele K, Zewotir T, Ndanguza D. Understanding correlates of child stunting in Ethiopia using generalized linear mixed models. *BMC Public Health* 2019;**19**. doi:10.1186/s12889-019-6984-x

31 Rajkumar, Andrew Sunil, Christopher Gaukler and JT. Combating malnutrition in Ethiopia: an evidence-based approach for sustained results. Washington, D.C.: : Washington, D.C. : World Bank 2012.

32 Tesfaye M. Bayesian approach to identify predictors of children Nutritional status in Ethiopia. 2009.

33 Tessema M, De Groote H, Brouwer ID, *et al.* Soil zinc is associated with serum zinc but not with linear growth of children in Ethiopia. *Nutrients* 2019;**11**:1–14. doi:10.3390/nu11020221

34 Disha A, Rawat R, Subandoro A, *et al.* Infant and young child feeding (IYCF) practices in Ethiopia and Zambia and their association with child nutrition: Analysis of demographic and health survey data. *African J Food, Agric Nutr Dev* 2012;**12**:5895–914.https://www.ajol.info/index.php/ajfand/article/view/75604 (accessed 24 Jul 2019).

35 Girmay M, Hanlon C, Dewey M, *et al.* Prevalence and predictors of undernutrition among infants aged six and twelve months in Butajira, Ethiopia: the P-MaMiE Birth Cohort. *BMC Public Health* 2010;**10**.http://www.biomedcentral.com/1471-2458/10/27

36 Farah AM, Endris BS, Gebreyesus SH. Maternal undernutrition as proxy indicators of their offspring’s undernutrition: evidence from 2011 Ethiopia demographic and health survey. *BMC Nutr* 2019;**5**. doi:10.1186/s40795-019-0281-z

37 Suri D, Griffiths J, Ghosh S. Factors associated with stunting in Ethiopian children under 5: A comparison of DHS 2000, 2005 and 2011. *FASEB J* 2014;**28**.http://www.fasebj.org/content/28/1_Supplement/620.6.abstract?sid=aef64871-15d2-41f6-87db-7a281b10b5de

38 Mohammed SH, Muhammad F, Pakzad R, *et al.* Socioeconomic inequality in stunting among under-5 children in Ethiopia: A decomposition analysis. *BMC Res Notes* 2019;**12**:1–5. doi:10.1186/s13104-019-4229-9

39 Abdulahi A, Shab-Bidar S, Rezaei S, *et al.* Nutritional Status of Under Five Children in Ethiopia: A Systematic Review and Meta-Analysis. *Ethiop J Heal Sci* 2017;**27**:175. doi:10.4314/ejhs.v27i2.10

40 Kinfe M. *COMPARISON OF NUTRITIONAL STATUS OF PRE-SCHOOL CHILDREN AMONG TWO AGRO ECOLOGICALLY DIFFERENT VILLAGES: THE CASE OF OFLA DISTRICT, SOUTHERN ZONE, TIGRAY, ETHIOPIA*. 2013.

41 Gibson RS, Yewelsew A, Hambidge KM, *et al.* Inadequate feeding practices and impaired growth among children from subsistence farming households in Sidama, Southern Ethiopia. *Matern Child Nutr* 2009;**5**:260–75. doi:http://dx.doi.org/10.1111/j.1740-8709.2008.00179.x

42 Wirth JP, Rohner F, Petry N, *et al.* Assessment of the WHO Stunting Framework using Ethiopia as a case study. *Matern Child Nutr* 2016;**13**. doi:10.1111/mcn.12310

43 Habtetsion M. *NUTRITIONAL STATUS OF CHILDREN AMONG IRRIGATION USER AND NON USER HOUSEHOLDS IN HINTALO WAJIRAT WOREDA, TIGRAY, ETHIOPIA: COMPARATIVE CROSS SECTIONAL STUDY*. 2013.

44 Christiaensen LJ, Alderman H. Child Malnutrition in Ethiopia: Can Maternal Knowledge Augment The Role of Income? Child Malnutrition in Ethiopia: Can Maternal Knowledge Augment The Role of Income? The authors would like to thank. 2001. http://www.worldbank.org/afr/wps/index.htm, (accessed 10 Jul 2019).

45 Mekonnen AW. *Prevalence of Stunting and its Associated Factors among 6 – 59 Months Old Children from Mersa Town, North Wollo Zone*. 2015.

46 Sohnesen TP, Alemayehu A, Fisker P, *et al.* Small area estimation of child malnutrition in Ethiopian woredas. *PLoS One* 2017;**12**:1–17.https://openknowledge.worldbank.org/bitstream/handle/10986/23924/Small0area0est0in0Ethiopian0woredas.pdf?sequence=1&isAllowed=y

47 Kang Y, Kim S, Sisay S, *et al.* Effectiveness of a community-based nutrition programme to improve child growth in rural Ethiopia: a cluster randomized trial. *Matern Child Nutr* 2017;**13**:e12349.http://onlinelibrary.wiley.com/journal/10.1111/(ISSN)1740-8709

48 White J, Mason J. Assessing the impact on child nutrition of the Ethiopia Community-based Nutrition Program. 2012. https://www.unicef.org/evaldatabase/files/Ethiopia_2013-057_CBN_Final_Report.pdf (accessed 11 Jul 2019).

49 Berhane G, Gilligan DO, Hoddinott J, *et al.* Can Social Protection Work in Africa? The Impact of Ethiopia’s Productive Safety Net Programme. *Econ Dev Cult Change* 2014;**63**:1–26. doi:10.1086/677753

50 Porter C, Radhika G. Social protection for all ages? Impacts of Ethiopia’s Productive Safety Net Program on child nutrition. *Soc Sci Med* 2016;**159**:92–9. doi:http://dx.doi.org/10.1016/j.socscimed.2016.05.001

51 Gebrehiwot T, Castilla C. Do Safety Net Transfers Improve Diets and Reduce Undernutrition? Evidence from Rural Ethiopia. *J Dev Stud* 2018;**55**:1947–66. doi:10.1080/00220388.2018.1502881

52 Skau, J., Belachew, T., Girma, T. and Woodruff BA. Outcome Evaluation Study of the Targeted Supplementary Food (TSF) Program in Ethiopia | ALNAP. 2009. https://www.alnap.org/help-library/outcome-evaluation-study-of-the-targeted-supplementary-food-tsf-program-in-ethiopia (accessed 11 Jul 2019).

53 Abate KH, Belachew T. Chronic Malnutrition Among Under Five Children of Ethiopia May Not Be Economic . A Systematic Review and Meta-Analysis. *Ethioop Heal Sci* 2018;**29**:265–77. doi:http://dx.doi.org/10.4314/

54 Bancha B, Tsegaye D, Yoseph H, *et al.* Determinants of stunting among children aged 6-59 months at Kindo Didaye woreda, Wolaita Zone, Southern Ethiopia: unmatched case control study. *PLoS One* 2017;**12**:e0189106. doi:http://dx.doi.org/10.1371/journal.pone.0189106

55 Ma’alin A, Birhanu D, Melaku S, *et al.* Magnitude and factors associated with malnutrition in children 6-59 months of age in Shinille Woreda, Ethiopian Somali regional state: a cross-sectional study. *BMC Nutr* Published Online First: 2016. doi:10.1186/s40795-016-0079-1

56 Tariku B, Mulugeta A, Tsadik M, *et al.* Prevalence and Risk Factors of Child Malnutrition in Community Based Nutrition Program Implementing and Nonimplementing Districts from South East Amhara, Ethiopia. *Open Access Libr J* 2014;**01**:1–17. doi:10.4236/oalib.1100425

57 Gebre A, Reddy PS, Mulugeta A, *et al.* Prevalence of Malnutrition and Associated Factors among Under-Five Children in Pastoral Communities of Afar Regional State , Northeast Ethiopia : A Community-Based Cross-Sectional Study. *J Nutr Metab* 2019;**2019**.

58 Mayer A.M., Save the Children UK. The causes of malnutrition in children under 3 in the Somali Region of Ethiopia related to household caring practices Preliminary Report. 2007;:1–5.

59 Gemechu A. *Determinants of Nutritional Status of Children in Amhara Region*. 2000.

60 Yinager Workineh B, Aman Yesuf E. Predictors of poor anthropometric status among children under two years of age in Gamo Gofa Zone, Southern Ethiopia, 2015; cross-sectional study. *Epidemiol Open Access* 2015;**5**:209.http://www.omicsonline.org/open-access/predictors-of-poor-anthropometric-status-among-children-under-twoyears-of-age-in-gamo-gofa-zone-southern-ethiopia-2015-crosssectio-2161-1165-1000209.php?aid=65523

61 Genebo T, Girma W, Jemal H, *et al.* The association of children’s nutritional status to maternal education in Zigbaboto, Guragie Zone, Ethiopia. *Ethiop J Heal Dev* 1999;**13**:55–61.

62 Shine S, Tadesse F, Shiferaw Z, *et al.* Prevalence and Associated Factors of Stunting among 6-59 Months Children in Pastoral Community of Korahay Zone, Somali Regional State, Ethiopia 2016. *J Nutr Disord Ther* 2017;**7**. doi:10.4172/2161-0509.1000208

63 Teshale F, Sahilu A, Lamessa D. Factors associated with stunting among children of age 24 to 59 months in Meskan district, Gurage Zone, South Ethiopia: a case-control study. *BMC Public Heal* 2014;**14**.http://www.biomedcentral.com/content/pdf/1471-2458-14-800.pdf

64 Rabia F, Bogale A, Degnet A. Prevalence of child malnutrition in agro-pastoral households in Afar Regional State of Ethiopia. *Nutr Res Pract* 2013;**7**:122–31. doi:http://dx.doi.org/10.4162/nrp.2013.7.2.122

65 Legesse Liben M, Abuhay T, Haile Y. Determinants of Child Malnutrition among Agro Pastorals in Northeastern Ethiopia: A Cross-Sectional Study. *Heal Sci J* 2016;**10**:1–10.http://search.ebscohost.com/login.aspx?direct=true&db=cin20&AN=118362665&site=ehost-live

66 Amsalu T, Tsedeke W, Anwar S. Under-nutrition and related factors among children aged 6-59 months in Gida Ayana district, Oromiya region, West Ethiopia: a community based quantitative study. *J Nutr Food Sci* 2016;**6**:543.https://www.omicsonline.org/open-access/undernutrition-and-related-factors-among-children-aged-659-monthsin-gida-ayana-district-oromiya-region-west-ethiopia-a-communityba-2155-9600-1000543.pdf

67 Haile A, Amboma A. Children’s nutritional status and its determinants in small towns, Sebeta Hawas district, Oromia, Ethiopia. *J Food Sci Nutr* 2018;**1**:33.http://www.alliedacademies.org/journal-food-science-nutrition/

68 Eskezyiaw A, Tefera C. Predictors of chronic under nutrition (Stunting) among children aged 6-23 months in Kemba Woreda, Southern Ethiopia: a community based cross-sectional study. *J Nutr Food Sci* 2015;**5**:381.http://www.omicsonline.org/open-access/predictors-of-chronic-under-nutrition-stunting-among-children-aged-623-months-in-kemba-woreda-southern-ethiopia-a-community-based-crosssectional-study-2155-9600-1000381.php?aid=56600

69 Desalegn BB, Kinfe E, Fikre K, *et al.* Stunting and Its Associated Factors in Under Five Years Old Children: The Case of Hawassa University Technology Villages, Southern Ethiopia. *IOSR J Environ Sci Toxicol Food Technol* 2016;**10**:25–31. doi:10.9790/2402-1011022531

70 Derso T, Tariku A, Biks GA, *et al.* Stunting, wasting and associated factors among children aged 6-24 months in Dabat health and demographic surveillance system site: A community based crosssectional study in Ethiopia. *BMC Pediatr* 2017;**17**:9. doi:10.1186/s12887-017-0848-2

71 Amare T, Biks GA, Terefe D, *et al.* Stunting and its determinant factors among children aged 6-59 months in Ethiopia. *Ital J Pediatr* 2017;**43**. doi:http://dx.doi.org/10.1186/s13052-017-0433-1

72 Mohammed SH, Esmaillzadeh A. The relationships among iron supplement use, Hb concentration and linear growth in young children: Ethiopian Demographic and Health Survey. *Br J Nutr* 2017;**118**:730–6. doi:10.1017/S0007114517002677

73 Semba RD, Pee S de, Sun K, *et al.* Coverage of the national vitamin A supplementation program in Ethiopia. *J Trop Pediatr* 2008;**54**:141–4. doi:http://dx.doi.org/10.1093/tropej/fmm095

74 Getaneh B, Kulkarni U, Yemane M, *et al.* Assessment of the Nutritional Status and Associated Factors of Orphans and Vulnerable Preschool Children on Care and Support from Nongovernmental Organizations in Hawassa Town. *Glob J Med Res* Published Online First: 2016.https://pdfs.semanticscholar.org/550c/e4d746059ab36445e0b9a52530a2340d8726.pdf (accessed 9 Jul 2019).

75 Headey D, Hirvonen K. Is Exposure to Poultry Harmful to Child Nutrition? An Observational Analysis for Rural Ethiopia. *PLoS One* 2016;**11**:e0160590. doi:10.1371/journal.pone.0160590

76 Zeid J, Kalkidan H, Tolassa W. Household food insecurity and its association with nutritional status among preschool children in Gambella town, western Ethiopia. *J Nutr Food Sci* 2016;**6**:566.https://www.omicsonline.org/open-access/household-food-insecurity-and-its-association-with-nutritional-statusamong-preschool-children-in-gambella-town-western-ethiopia-2155-9600-1000566.php?aid=81931

77 Hiwot Y, Tesfaye G, Firehiwot M. Prevalence and risk factors for under nutrition among children under five at Haramaya district, eastern Ethiopia. *BMC Pediatr* 2015;**15**.http://bmcpediatr.biomedcentral.com/articles/10.1186/s12887-015-0535-0

78 Beyene TT. Predictors of Nutritional Status of Children Visiting Health Facilities in Jimma Zone, South West Ethiopia. *Int J Adv Nurs Sci Pract* 2012;**1**:1–13. doi:10.5923/j.nursing.20110101.01

79 Abebaw D. Stunting and Associated Factors among Children Aged 6-59 Months in Lasta Woreda, North East Ethiopia, 2015: A Community Based Cross Sectional Study Design. *J Fam Med* 2017;**4**. doi:10.26420/jfammed.2017.1112

80 Tesfamariam K, Yilma D. Prevalence of Stunting and its Associated Factors Among Children Under 5 Age in Holeta Town. 2017. https://www.ecronicon.com/ecnu/pdf/ECNU-12-00406.pdf (accessed 10 Jul 2019).

81 S.H. M, T.D. H, A. E. Household, maternal, and child related determinants of hemoglobin levels of Ethiopian children: Hierarchical regression analysis. *BMC Pediatr* 2019;**19**:1–10. doi:10.1186/s12887-019-1476-9 LK - http://WT3CF4ET2L.search.serialssolutions.com?sid=EMBASE&issn=14712431&id=doi:10.1186%2Fs12887-019-1476-9&atitle=Household%2C+maternal%2C+and+child+related+determinants+of+hemoglobin+levels+of+Ethiopian+children%3A+Hierarchical+regression+analysis&stitle=BMC+Pediatr.&title=BMC+Pediatrics&volume=19&issue=1&spage=&epage=&aulast=Mohammed&aufirst=Shimels+Hussien&auinit=S.H.&aufull=Mohammed+S.H.&coden=BPMEB&isbn=&pages=-&date=2019&auinit1=S&auinitm=H

82 Mengistu K, Alemu K, Destaw B. Thesis Open Access 2013 Prevalence of Malnutrition and Associated Factors Among Children Aged 6-59 Months at Hidabu Abote District, North Shewa, Oromia Regional State. *J Nutr Disord Ther* 2013;:1. doi:10.4172/2161-0509.1000T1-001

83 Mussie A, Fitiwi T, Kiday H, *et al.* Undernutrition status and associated factors in under-5 children, in Tigray, Northern Ethiopia. *Nutrition* 2015;**31**:964–70.http://www.sciencedirect.com/science/article/pii/S0899900715000817

84 Hailemariam TW. Prevalence of Underweight and its Determinant Factors of under Two Children in a Rural Area of Western Ethiopia. *Food Sci Qual Manag* 2014;**31**:59–70.www.iiste.org

85 Demissie S, Worku A. Solomon Demissie, Amare Worku. Magnitude and Factors Associated with Malnutrition in Children 6-59 Months of Age in Pastoral Community of Dollo Ado District. *Sci J Public Heal* 2013;**1**:175–83. doi:10.11648/j.sjph.20130104.12

86 Zeray A, Kibret GD, Leshargie CT. Prevalence and associated factors of undernutrition among under-five children from model and non-model households in east Gojjam zone, Northwest Ethiopia: a comparative cross-sectional study. *BMC Nutr* 2019;**5**. doi:10.1186/s40795-019-0290-y

87 Tadesse A. Nutritional Status and Associated Factors Among Pastoralist Children Aged 6-23 Months in Benna Tsemay Woreda, South Omo Zone, Southern Ethiopia. *Int J Nutr Food Sci* 2018;**7**:11. doi:10.11648/j.ijnfs.20180701.13

88 Wasihun AG, Dejene TA, Teferi M, *et al.* Risk factors for diarrhoea and malnutrition among children under the age of 5 years in the Tigray Region of Northern Ethiopia. *PLoS One* 2018;**13**:e0207743. doi:10.1371/journal.pone.0207743

89 Malako BG, Asamoah BO, Tadesse M, *et al.* Stunting and anemia among children 6–23 months old in Damot Sore district, Southern Ethiopia. *BMC Nutr* 2019;**5**:3. doi:10.1186/s40795-018-0268-1

90 Lamirot A, Tariku D, Tariku L. Prevalence of malnutrition and associated factors in children aged 6-59 months among rural dwellers of Damot Gale district, South Ethiopia: community based cross sectional study. *Int J Equity Health* 2017;**16**. doi:http://dx.doi.org/10.1186/s12939-017-0608-9

91 Canaan N, Whiting SJ, Henry CJ, *et al.* Association between maternal and child nutritional status in Hula, rural southern Ethiopia: a cross sectional study. *PLoS One* 2015;**10**:e0142301.http://journals.plos.org/plosone/article?id=10.1371/journal.pone.0142301

92 Mulugeta A, Hagos F, Kruseman G, *et al.* Child malnutrition in Tigray, northern Ethiopia. *East Afr Med J* 2010;**87**:248–54.http://www.ncbi.nlm.nih.gov/pubmed/23057267 (accessed 9 Jul 2019).

93 Kahsay A. Nutritional Status of Children (6-59 Months) from Food Secure and Food Insecure Households in Rural Communities of Saesie Tsaeda-Emba District, Tigray, North Ethiopia: Comparative Study. *Int J Nutr Food Sci* 2015;**4**:51. doi:10.11648/j.ijnfs.20150401.18

94 Dearden KA, Schott W, Crookston BT, *et al.* Children with access to improved sanitation but not improved water are at lower risk of stunting compared to children without access: a cohort study in Ethiopia, India, Peru, and Vietnam. *BMC Public Heal* 2017;**17**:110. doi:https://dx.doi.org/10.1186/s12889-017-4033-1

95 Kastro Dake S, Solomon B, Bobe TM, *et al.* Predictors of stunting among children 6-59 months of age in Sodo Zuria District, South Ethiopia: a community based cross-sectional study. *BMC Nutr* 2019;**5**. doi:10.1186/s40795-019-0287-6

96 Yalew BM. Prevalence and Factors Associated with Stunting, Underweight and Wasting: A Community Based Cross Sectional Study among Children Age 6-59 Months at Lalibela Town, Northern Ethiopia. *J Nutr Disord Ther* 2014;**4**. doi:10.4172/2161-0509.1000147

97 Berhanu G, Mekonnen S, Sisay M. Prevalence of stunting and associated factors among preschool children: A community based comparative cross sectional study in Ethiopia. *BMC Nutr* 2018;**4**:28. doi:10.1186/s40795-018-0236-9

98 Assefa KM, Alemu K, Destaw B. Prevalence of malnutrition and associated factors among children aged 6-59 months at Hidabu Abote District, North Shewa, Oromia Regional State. *Ann Nutr Metab* 2015;**67**:216–7. doi:http://dx.doi.org/10.1159/000440895

99 Amare T, Haile W, Abel F, *et al.* Nearly half of preschool children are stunted in Dembia district, Northwest Ethiopia: a community based crosssectional study. *Arch Public Heal* 2016;**74**.http://download.springer.com/static/pdf/405/art%253A10.1186%252Fs13690-016-0126-z.pdf?originUrl=http%3A%2F%2Farchpublichealth.biomedcentral.com%2Farticle%2F10.1186%2Fs13690-016-0126-z&token2=exp=1462357706~acl=%2Fstatic%2Fpdf%2F405%2Fart%25253A10.1186%252

100 Mandefro A, Mekitie W, Mohammed T, *et al.* Prevalence of undernutrition and associated factors among children aged between six to fifty nine months in Bule Hora district, South Ethiopia. *BMC Public Health* 2015;**15**.http://www.biomedcentral.com/1471-2458/15/41

101 Wondemeneh Tamiru M. Under Nutrition and Associated Factors Among Under-Five Age Children of Kunama Ethnic Groups in Tahtay Adiyabo Woreda, Tigray Regional State, Ethiopia: Community based study. *Int J Nutr Food Sci* 2015;**4**:277. doi:10.11648/j.ijnfs.20150403.15

102 Adeba A, Garoma S, Gemede HF, *et al.* Nutritional Quality and Health Benefits of Okra (Abelmoschus Esculentus): A Review. *Int J Nutr Food Sci* 2015;**4**:208=215.https://www.researchgate.net/publication/308903851 (accessed 10 Jul 2019).

103 Abate KH, Belachew T. Care and not wealth is a predictor of wasting and stunting of ‘The Coffee Kids’ of Jimma Zone, southwest Ethiopia. *Nutr Health* 2017;**23**:193–202. doi:https://dx.doi.org/10.1177/0260106017706253

104 Kalkidan Hassen A, Tefera B. Women’s autonomy and men’s involvement in child care and feeding as predictors of infant and young child anthropometric indices in coffee farming households of Jimma zone, South West of Ethiopia. *PLoS One* 2017;**12**:e0172885. doi:http://dx.doi.org/10.1371/journal.pone.0172885

105 Bealu B, Tekle E, Fissahaye A, *et al.* Household food insecurity and its association with nutritional status of children 6-59 months of age in East Badawacho District, South Ethiopia. *J Environ Public Health* 2017;**2017**:Article-6373595. doi:http://dx.doi.org/10.1155/2017/6373595

106 Fekadu Y, Mesfin A, Haile D, *et al.* Factors associated with nutritional status of infants and young children in Somali Region, Ethiopia: A cross- sectional study Global health. *BMC Public Health* 2015;**15**. doi:10.1186/s12889-015-2190-7

107 Okike I, Jabbar MA, Gugsa A, *et al.* Household and environmental factors influencing anthropometric outcomes in preschool children in a rural Ethiopian community. *Ecol Food Nutr Nutr* 2005;**44**:167–87. doi:http://dx.doi.org/10.1080/03670240590952990

108 Masresha T, Tefera B, Getahun E. Feeding patterns and stunting during early childhood in rural communities of Sidama, South Ethiopia. *Pan Afr Med J* 2013;**14**:75.http://www.panafrican-med-journal.com/content/article/14/75/full/

109 Zeweter A, Haki GD, Kaleab B. Health extension workers’ knowledge and knowledge-sharing effectiveness of optimal infant and young child feeding are associated with mothers’ knowledge and child stunting in rural Ethiopia. *Food Nutr Bull* 2016;**37**:353–63.http://fnb.sagepub.com/

110 Brhane G, Regassa N. Nutritional status of children under five years of age in Shire Indaselassie, North Ethiopia: Examining the prevalence and risk factors. *Kontakt* 2014;**16**:e161–70. doi:10.1016/j.kontakt.2014.06.003

111 Hiwot D, Ayele G, Abebaw G, *et al.* Magnitude and predictors of undernutrition among children aged six to fifty nine months in Ethiopia: a cross sectional study. *Arch Public Heal* 2017;**75**. doi:http://dx.doi.org/10.1186/s13690-017-0198-4

112 Kogi-Makau W, Taye G, Teshome B. Magnitude and Determinants of Stunting in Children Underfive Years of Age in Food Surplus Region of Ethiopia: the Case of West Gojam Zone. *Ethiop J Heal Dev* 2009;**23**:98–106.

113 Netsanet F, Tefera B, Carl L. Determinants and morbidities of multiple anthropometric deficits in southwest rural Ethiopia. *Nutrition* 2016;**32**:1243–9.http://www.sciencedirect.com/science/article/pii/S0899900716300387

114 Demilew YM, Abie DD. Undernutrition and associated factors among 24–36-month-old children in slum areas of Bahir Dar city, Ethiopia. *Int J Gen Med* 2017;**10**:79–86. doi:10.2147/IJGM.S126241

115 Hiwot E, Yewelsew A, Eskindir L, *et al.* Nutritional status and effect of maternal employment among children aged 6-59 months in Wolayta Sodo Town, Southern Ethiopia: a cross-sectional study. *Ethiop J Health Sci* 2017;**27**:155–62. doi:http://dx.doi.org/10.4314/ejhs.v27i2.8

116 Sako B, Leerlooijer JN, Lelisa A, *et al.* Exploring barriers and enablers for scaling up a community‐based grain bank intervention for improved infant and young child feeding in Ethiopia: A qualitative process evaluation. *Matern Child Nutr* 2018;**14**:1. doi:10.1111/mcn.12551

117 Tesfay GB, Abidoye B. Shocks in food availability and intra-household resources allocation: evidence on children nutrition outcomes in Ethiopia. *Agric Food Econ* 2019;**7**:1–21. doi:10.1186/s40100-019-0120-1

118 Fikadu Reta A. Predictors of nutritional status and mortality of children in Southern Ethiopia. *J Biol Agric Healthc* 2015;**5**:205–17.http://www.iiste.org/Journals/index.php/JBAH/article/view/20645/21580

119 Moges B, Feleke A, Meseret S, *et al.* Magnitude of stunting and associated factors among 6-59 months old children in Hossana Town, Southern Ethiopia. *J Clin Res Bioeth* 2015;**6**:207.http://omicsonline.org/open-access/magnitude-of-stunting-and-associated-factors-among-months-old-children-2155-9627.1000207.php?aid=37208

120 Pelletier DL, Deneke K, Kidane Y, *et al.* THE FOOD-FIRST BIAS AND NUTRITION POLICY - LESSONS FROM ETHIOPIA. *Food Policy* 1995;**20**:279–98. doi:10.1016/0306-9192(95)00026-7

121 Ali D, Saha KK, Nguyen PH, *et al.* Household Food Insecurity Is Associated with Higher Child Undernutrition in Bangladesh, Ethiopia, and Vietnam, but the Effect Is Not Mediated by Child Dietary Diversity. *J Nutr* 2013;**143**:2015–21. doi:10.3945/jn.113.175182

122 Jones AD, Ickes SB, Smith LE, *et al.* World Health Organization infant and young child feeding indicators and their associations with child anthropometry: a synthesis of recent findings. *Matern Child Nutr* 2013;**10**:1–17. doi:10.1111/mcn.12070

123 S.H. M, T.D. H, A. E. Household, maternal, and child related determinants of hemoglobin levels of Ethiopian children: Hierarchical regression analysis. *BMC Pediatr* 2019;**19**:1–10. doi:10.1186/s12887-019-1476-9 LK - http://WT3CF4ET2L.search.serialssolutions.com?sid=EMBASE&issn=14712431&id=doi:10.1186%2Fs12887-019-1476-9&atitle=Household%2C+maternal%2C+and+child+related+determinants+of+hemoglobin+levels+of+Ethiopian+children%3A+Hierarchical+regression+analysis&stitle=BMC+Pediatr.&title=BMC+Pediatrics&volume=19&issue=1&spage=&epage=&aulast=Mohammed&aufirst=Shimels+Hussien&auinit=S.H.&aufull=Mohammed+S.H.&coden=BPMEB&isbn=&pages=-&date=2019&auinit1=S&auinitm=H

124 Hoddinott J, Headey D, Dereje M. Cows, Missing Milk Markets, and Nutrition in Rural Ethiopia. *J Dev Stud* 2015;**51**:958–75. doi:10.1080/00220388.2015.1018903

125 Lindtjorn B, Alemu T, Bjorvatn B. NUTRITIONAL-STATUS AND RISK OF INFECTION AMONG ETHIOPIAN CHILDREN. *J Trop Pediatr* 1993;**39**:76–82. doi:10.1093/tropej/39.2.76

126 Amsalu ET, Akalu TY, Gelaye KA. Spatial distribution and determinants of acute respiratory infection among under-five children in Ethiopia: Ethiopian demographic Health Survey 2016. *PLoS One* 2019;**14**:1–14. doi:10.1371/journal.pone.0215572

127 Megabiaw B. Prevalence and Determinants of Chronic Malnutrition Among Under-5 Children in Ethiopia. *Int J Child Heal Nutr* Published Online First: 2013. doi:10.6000/1929-4247.2013.02.03.5

128 Zewdie Aderaw A, Ahmed Ali A, Alemayehu Worku Y, *et al.* Non random distribution of child undernutrition in Ethiopia: spatial analysis from the 2011 Ethiopia demographic and health survey. *Int J Equity Health* 2016;**15**.http://equityhealthj.biomedcentral.com/articles/10.1186/s12939-016-0480-z

129 Lindtjørn B, Alemu T. Year-to-year and seasonal variations in stunting among preschool children in Ethiopia. *J Heal Popul Nutr* 2002;**20**:326–33.https://www.scopus.com/inward/record.uri?eid=2-s2.0-17444439022&partnerID=40&md5=363d67fc52dc8ce63db0868d4bd2cf4b

130 Jann B. The Blinder–Oaxaca decomposition for linear regression models. *Stata J* 2008;**8**:453–79.

131 Headey D, Hoddinott J, Park S. Accounting for nutritional changes in six success stories: A regression- decomposition approach. *Glob Food Sec* 2017;**13**:12–20. doi:10.1016/j.gfs.2017.02.003

132 Headey DD, Hoddinott J. Understanding the Rapid Reduction of Undernutrition in Nepal. *PLoS One* 2015;**10**:e0145738. doi:10.1371/journal.pone.0145738

133 Headey D, Hoddinott J, Park S. Drivers of nutritional change in four South Asian countries: A dynamic observational analysis. *Matern Child Nutr* 2016;**12**:210–8. doi:10.1111/mcn.12274

134 Alderman H, Headey D. The timing of growth faltering has important implications for observational analyses of the underlying determinants of nutrition outcomes. *PLoS One* 2018;**13**:e0195904. doi:10.1371/journal.pone.0195904

135 Restrepo-Méndez MC, Barros AJ, Black RE, *et al.* Time trends in socio-economic inequalities in stunting prevalence: analyses of repeated national surveys. *Public Health Nutr* 2014;**18**:2097–104. doi:10.1017/S1368980014002924

136 Sandelowski M. Focus on Qualitative Methods Sample Size in Qualitative. *Res Nurs Heal* 1995;**18**:179–83. doi:10.1002/nur.4770180211

137 Green J, Browne J. *Principles of Social Research*. Maidenhead: : Open University Press 2009.

138 Victora C G, Huttly, S R, Fuchs, S C, *et al.* The role of conceptual frameworks in epidemiological analysis: a hierarchical approach. *Int J Epidemiol* 1997;**26**:224–7.

139 Black RE, Victora CG, Walker SP, *et al.* Maternal and child undernutrition and overweight in low-income and middle-income countries. *Lancet* 2013;**382**:427–51. doi:10.1016/S0140-6736(13)60937-X

140 Council of Ministers Regulations No. 4/1996 Council of Ministers Regulations to Provide For The Establishment Of The Ethiopian Health And Nutrition Research Institute. Ethiopia: 1996.

141 Ethiopian Public Health Institute Website. 2018.https://www.ephi.gov.et/index.php

142 To AP, For P, Public THE, *et al.* A Proclamarion to Provide For The Public Ownership of Rural Lands. Ethiopia: 1975.

143 Abebe M. The March 1975 ‘Land to the Tiller’ Proclamation: Dream or Reality? *Am Res J Hist Cult* 2017;**2016**:1–5. doi:10.21694/2379-2914.16002

144 Federal Civil Servants Proclamation No. 515/2007. Ethiopia: 2007.

145 Federal Civil Servants Proclamation No. ----/ 2017. Ethiopia: 2017.

146 Yifru B, Negash S, Abebe Y, *et al.* Module II : Adolescent , Maternal , Infant & Young Child Nutrition ( AMIYCN ). 2016.

147 Marketing of breast-milk substitutes: National implementation of the international code: Status report 2018. 2016. doi:10.1093/heapol/czw088

148 Yohannes A. Salt Iodization Council of Ministers Regulation. 2011.https://chilot.me/2011/11/22/salt-iodization-council-of-ministers-regulation-no-20412011/

149 Adish A, Chuko T, Abay A, *et al.* Ethiopia: breaking through with a new iodized salt law. *IDD Newsl* 2013;**41**:7–8.

150 Ethiopian National Micronutrient Survey Report. 2016.

151 Zerfu D. National salt iodization coverage towards Prevention of Iodine Deficiency Disorder in Ethiopia. 2014.

152 Central Statistical Agency of Ethiopia, ICF. Ethiopia Demographic and Health Survey 2016. Addis Ababa, Ethiopia: 2016.

153 The Federal Democratic Republic of Ethiopia. Sequota Declaration Implementation Plan (2016-2030). 2016.

154 Health Policy of the Transitional Government of Ethiopia. 1993.

155 Dube AK, Fawole WO, Govindasamy R, *et al.* Agricultural Development Led Industrialization in Ethiopia: Structural Break Analysis.

156 Agricultrual Transformation Agency. Ethiopian Agriculture and Strategies for Growth. 2017.

157 Education and Training Policy. 1994.

158 Cheever M, Graichen K, Homeier D, *et al.* Environmental Policy Review 2011 Environmental Policy Review: Key Issues in Ethiopia 2011. 2011.http://web.colby.edu/eastafricaupdate/

159 Environment Policy of Ethiopia. 1997.

160 National Five-Year Strategic Plan For Malaria Prevention & Control in Ethiopia 2006-2010. 2006.

161 National Malaria Control Program Monitoring and Evaluation Plan 2014-2020. Addis Ababa, Ethiopia: 2014. doi:0360-3016(88)90012-0 [pii]

162 Bayissa GA. Accelerate Malaria Control Program Implementation in Ethiopia ; Strengths and Weaknesses. 2016.

163 Ababa A. National Strategic Plan for Malaria Prevention Control and Elimination in Ethiopia 2011-2015. 2010.

164 Ethiopia National Malaria Indicator Survey 2015. Addis Ababa, Ethiopia: 2015.

165 President’s Malaria Initiative: Ethiopia- Malaria Operational Plan FY 2018. 2015.

166 Ethiopian Water Sector Policy. 2001.

167 Ethiopian Water Sector Strategy. 2001.

168 Industry Development Strategy of Ethiopia. 2002.

169 Gebreeyesus M. *Industrial Policy and Development in Ethiopia*. 2016. doi:10.1093/acprof

170 Federal Democratic Republic of Ethiopia M of F and ED. Ethiopia: Sustainable Development and Poverty Reduction Program. 2002.

171 National Strategy for Infant and Young Child Feeding. 2004.

172 Harris J, Frongillo EA, Nguyen PH, *et al.* Changes in the policy environment for infant and young child feeding in Vietnam, Bangladesh, and Ethiopia, and the role of targeted advocacy. *BMC Public Health* 2017;**17**. doi:10.1186/s12889-017-4343-3

173 Ministry of Finance and Economic Development (MoFED). Growth and Transformation Plan (GTP) 2010/11- 2014/15 Draft. 2010.

174 Development EM of F and E. Ethiopia : Building on Progress A Plan for Accelerated and Sustained Development to End Poverty ( PASDEP ) Volume I : Main Text. 2006;**I**.

175 National Strategy for Child Survival. Addis Ababa, Ethiopia: 2005.

176 National Strategy for Newborn and Child Survival in Ethiopia 2015/16 - 2019/20. 2015.

177 Federal Democratic Republic of Ethiopia | Ministry of Health. National Health Promotion and Communication Strategy 2016-2020. 2016. https://www.medbox.org/ethiopia/national-health-promotion-and-communication-strategy-2016-2020/preview?

178 National Hygiene and Sanitation Strategy. 2005.

179 National Nutrition Strategy. Addis Ababa, Ethiopia: 2008.

180 IMF. Growth and Transformation Plan ( 2010/11-2014/15). Addis Ababa, Ethiopia: 2010. http://www.imf.org

181 Growth and Transformation Plan II (GTP II) (2015/16-2019/20). 2016.

182 Chipeta M, Emana B, Chanyalew D. Ethiopia’s Agriculture Sector Policy and Investment Framework (2010–2020) External Mid-term Review. 2015. doi:10.1016/S1360-1385(00)01813-6

183 National Hygiene & Sanitation Strategic Action Plan for Rural, Per-Urban & Informal Settlements in Ethiopia, Part II. Addis Ababa, Ethiopia: 2011.

184 Ethiopia’s Climate Resilient Green Economy: Green Economy Strategy. 2011. http://sustainabledevelopment.un.org/index.php?page=view&type=400&nr=677&menu=865

185 Integrated Urban Sanitation and Hygiene Strategy. Addis Ababa, Ethiopia: 2017. doi:10.1109/VS-GAMES.2015.7295756

186 Ethiopian National Health Care Quality Strategy 2016-2020: Transforming The Quality of Health Care in Ethiopia. Addis Ababa, Ethiopia: 2016. doi:http://dx.doi.org/10.1111/j.1538-7836.2011.04380_4.x

187 EU + Joint Strategy on Nutrition for Ethiopia 2016-2020. 2016. https://eeas.europa.eu/sites/eeas/files/eu_joint_strategy_on_nutrition_for_ethiopia_2016-2020_1.pdf

188 Hygiene N. National Hygiene and Environmental Health Strategy (2016-2020). 2016.

189 Federal Democratic Republic of Ethiopia National Nutrition Sensitive Agriculture Strategy. Addis Ababa, Ethiopia: 2016.

190 Health EFM of. Expanded Program on Immunisation ( EPI ). 2004.

191 Ethiopian National Expanded Programme On Immunization: Comprehensive Multi-Year Plan 2016-2020. 2015.

192 Basic Education, Technical and Vocational Training Project (EducationII): Project Completion Report. 2004.

193 Health FDR of EM of. Health Sector Development Program-IV 2010/11-2014/15. 2010.

194 Health Sector Strategic Plan (HSDP-III) 2005/6-2009/10. 2005.

195 The Ethiopian Education Sector Development Program. doi:10.5771/9783845259178-34

196 Education Sector Development Program IV (2010/2011-2014/2015) Program Action Plan /PAP. Addis Ababa, Ethiopia: 2010.

197 Education Sector Development Programme V ( ESDP V ). Addis Ababa, Ethiopia: 2008.

198 Nielsen J, Guyon A, Quinn V. Scaling-up high impact nutrition interventions: Experiences with the essential nutrition actions framework. *Ann Nutr Metab* 2013;**63**:419–20. doi:http://dx.doi.org/10.1159/000354245

199 Evidence for Essential Nutrition Actions. Essential Nutrition Actions Improving Maternal-Newborn-Infant and Young Child Health and Nutriton. 2011.

200 Health Extension Progam In Ethiopia. Addis Ababa, Ethiopia: 2007.

201 Sebhatu A. The Implementation of Ethiopia’s Health Extension Program: An Overview. Addis Ababa, Ethiopia: 2008.

202 Wang H, Tesfaye R, Ramana GN V, *et al.* Ethiopia Health Extension Program: An Institutionalized Community Approach for Universal Health Coverage. 2016.

203 Damtew ZA, Chekagn CT, Moges AS. The health extension program of Ethiopia: Strengthening the Community Health System. Published Online First: 2016.http://www.hhpronline.org/articles/2016/12/17/the-health-extension-program-of-ethiopia

204 Assefa Y, Gelaw YA, Hill PS, *et al.* Community health extension program of Ethiopia, 2003-2018: Successes and challenges toward universal coverage for primary healthcare services. *Global Health* 2019;**15**. doi:10.1186/s12992-019-0470-1

205 Colman D, Mellor J. The Ethiopia Strategy Support Program (ESSP): Example of an Impact-Focused Approach. http://weekly.cnbnews.com/news/article.html?no=124000

206 Dorosh P. Ethiopia Strategy Support Program II: Bridging Knowledge and Policy. 2010.

207 Selamawit N. Enhanced outreach strategy/targeted supplementary feeding for child survival in Ethiopia (EOS/TSF). *Emerg Nutr Netw* 2011;:7–10.http://fex.ennonline.net/pdf/40.pdf

208 MOH. Integrating Enhanced Outreach Strategy into Health Extension Programme Ethiopia, A Transitional Plan. 2010.

209 Diop M, Turk C, Ringold D, *et al.* Productive Safety Net APL III Project. 2016.

210 Productive Safety Net Program Phase 4 (PSNP 4): Enhanced Social Assessment and Consultation Final Repor. 2014.

211 Berhane G, Hoddinott J, Kumar N, *et al.* The Productive Safety Net Programme in Ethiopia: Impacts on Children’s Schooling, Labour and Nutritional Status. 2017. http://www.3ieimpact.org/media/filer_public/2017/01/11/ie55_psnp_ethiopia_v1.pdf

212 Community Based Nutrition Baseline Survey Report. 2013.

213 Woldehanna T. The Policy Environment for Linking Agriculture and Nutrition in Ethiopia. 2014.

214 National Nutrition Program 2016-2020. 2016. http://www.youthpolicy.org/national/Ethiopia_2004_National_Youth_Policy.pdf

215 FMOH. National Nutrition Programme June 2013- June 2015. 2013.

216 Water and Sanitation Program- About. 2018.https://www.wsp.org/index.php/about

217 Water and Sanitation Program: End of Year Report, Fiscal Year 2014. 2013.

218 Water and Sanitation Program, Africa FY15: Highlights and Achievements. Published Online First: 2019.http://www.un.org/waterforlifedecade/africa.shtml

219 Food Security Programme 2010-2014. 2009. https://mhhisrael.files.wordpress.com/2013/09/d7a0d799d794d795d79c-d7aad795d7a6d7a8d799-d794d79cd795d795d790d799-d791d797d7a7d79cd790d795d7aa-d799d7a9d7a8d790d79c.pdf

220 National iCCM-CBNC Quality Improvement and Transition Plan July 2017-June 2019. Addis Ababa, Ethiopia: 2017.

221 Ameha A, Karim AM, Erbo A, *et al.* Effectiveness of supportive supervision on the quality of integrated Community Cases Management services in 113 districts of Ethiopia. *Ethiop Med J* 2014;**52**:73–81.

222 Ethiopian Federal Ministry of Agriculture. Agricultural Growth Program II. 2015.

223 Manning R, Espeut D, Stibbe D, *et al.* Midterm Review of the Scaling up Nutrition Movement: Final Report. 2018. https://scalingupnutrition.org/wp-content/uploads/2019/03/SUN-MTR-Final-Report-2019_external-1.pdf

224 Scaling Up Nutrition ( SUN ) Movement Strategy (2012-2015). 2015.

225 SUN Movement: Revised Road Map. 2012.

226 SUN Movement Strategy and Roadmap (2016-2020). 2016.

227 2018 SUN Country Profile: Ethiopia. 2018.

228 Sustainable Undernutrition Reduction in Ethiopia (SURE!). Published Online First: 2014.ttps://ciff.org/grant-portfolio/ethiopia-stunting-prevention

229 Ethiopia TFDR of. ONE WaSH NATIONAL PROGRAM, Program Operational Manual. 2014.

230 The Government of Ethiopia and the European Commission. National Indicative Programme for Ethiopia 2014 to 2020. 2014.

231 Federal Democratic Republic of Ethiopia | Ministry of Health. Ethiopian Health Sector Transformation Plan 2015/16 - 2019/20. *Fed Democr Repub Ethiop* 2015;**20**:25.https://www.globalfinancingfacility.org/sites/gff_new/files/Ethiopia-health-system-transformation-plan.pdf
