# Supplementary material for: Drivers of stunting reduction in Ethiopia: a country case study
Source: Am J Clin Nutr. 2020 Aug 25;112(Suppl 2):875S–893S. doi: 10.1093/ajcn/nqaa163 (PMC7487434; doi:10.1093/ajcn/nqaa163)
Supplement: nqaa163_Supplemental_Files [file nqaa163_supplemental_files.zip › Ethiopia Stunting Case Study Appendix May082020.docx]

**Appendix**

**Appendix 1: Systematic literature review of stunting determinants**

Our literature review identified several basic determinants of stunting in Ethiopia including ethnicity, economic development, governance, regional variation, climate and food shocks, wealth index, women’s empowerment, and parental education. Ethnicity was found not to be associated with stunting risk (16), though religion was identified by four studies as associated with stunting, however they found conflicting evidence on which religion conferred greater risk (17–20). One study suggests that economic development in Ethiopia was associated with improved nutritional status and stunting reductions (21), while another suggests that good governance was positively associated with stunting decline (22). Various studies have identified regional differences in stunting and found that northernmost regions Tigray, Amhara and Afar were generally associated with increased stunting prevalence, and southernmost regions Oromiya, Somali, and SNNPR were associated with decreased stunting (21–29). Climate change, namely rainfall, drought and temperature change do predict some of the variation in child stunting in Ethiopia (27,30), while the limited evidence of the impact of food shocks on stunting outcomes tends to show a lack of association (29,31,32). Increased wealth was found to be positively associated with HAZ and thus negatively associated with stunting in a large number of studies (21,22,24,26,28,33–50), while some found no association (51–55). Women’s empowerment was difficult to quantify, and studies examining this variable suggested that the impact of proxies such as households being headed by women (26,37,47,51,56), women’s autonomy (54), and mothers’ employment (39,50,55,57) had conflicting or no association with HAZ in Ethiopia. Parental education has been shown to be positively associated with HAZ and negatively associated with stunting in the vast majority of both national (21,22,24,30,33,34) and subnational studies (17,26,38,40,41,46–48,50,51,56,58) though some studies found no association (17,35,37,42,43,53,55,57,59).

Nutrition-specific and –sensitive programs identified in literature were the Community-based Nutrition Program (CBNP), Productive Safety Net Program (PSNP), and the Targeted Supplementary Food Program (TSF). Two studies on the CBNP found that it was associated with increase in LAZ and decrease in stunting (60,61). The PSNP, however, was not found to have had an impact on stunting outcomes, though perhaps follow-up was not long enough to show these impacts (62–64). An evaluation of the TSF also showed no changes in child stunting outcomes, though there were differences between the intervention and control groups at baseline that render this finding uncertain (65).

Among the underlying causes of stunting, literature suggests that immunization, skilled birth attendance, antenatal care, postnatal care, vitamin A supplementation, health care access and utilization, household environment, feeding practices and food security were contributing factors. The majority of studies in our review found that childhood immunizations have a protective effect against stunting outcomes (66–72), though some found no association (26,42,73–76). Healthcare interventions such as delivery by a skilled birth attendant (16,34,35,77), antenatal care (24,34,35,39,42,50,66,69,78–81), and postnatal care (76,82) have been found to be associated with reduced stunting outcomes in most studies, though some found no association (16–18,24,33,48,49,51,53,57,76,81,83–89). Vitamin A supplementation is a promising initiative shown to be associated with a decreased risk of stunting, though variation in findings exists (49,69,90–95). Studies that examining distance or time to a health facility typically found that these were not strongly related to stunting (34,45,56,80,92,93,96–98). There is substantial evidence in the literature that rural residence confers a higher likelihood of stunting compared to urban residence (17,19,21,28,30,39,41,47,54,55,66,72,99–103), though some studies found no association (22,24,26,35,39,48–50,53,56,82,104,105), while one found that rural living children were protected (46). Improvements in WASH indicators such as access to improved water sources (22,37,39,55,56,66,67,69,73,76,89,98,100,106–110), distance to fetch water (59), access to improved sanitation (21,22,26,34,40,48,49,66,69,73,76,111–113),and open defecation (33,48), have been generally found to be positively associated with HAZ though some did find insignificant results (17,21,24–26,33–35,37,39,40,42–44,48–51,68,70,76,77,89,93,96–98,105,111,112,114–124). Hygiene was difficult to quantify, and this indicator was not found to be associated with stunting outcomes (25,33,44). Household crowding was examined in a large number of studies, of which the majority found no association with stunting, though some did find that smaller family size was associated with lower stunting (21,23,26,51,56,67–69,73,80,95,99,101,102,107,113,120–122,124–128). A variety of breastfeeding practices were measured, and evidence of the association between breastfeeding and child growth in Ethiopia is mixed, though several studies did note a positive impact (18,25,28,31,33,38,40,42,45,49,53,54,67,69,71,74,76–79,89,93,95,98,101,107,108,116,120,121,123,124,128–143). Nearly all of the studies that explored the relationship between stunting outcomes and complementary feeding found that it is significantly associated with child growth (53,92,110,115,129,130,132,136,139,144). A number of studies examined the impact of food security on growth and most found that household food insecurity was negatively associated with HAZ, or positively associated with stunting (16,25,37,98,99,127–130,145,146).

Immediate Causes of stunting decline were identified as dietary intake, disease, maternal characteristics (age, height, BMI, and parity), and child characteristics (age, gender, birthweight, birth order, and number of children in the household). Studies highlighted the significance of improved dietary intake, dietary diversity, and consumption of multiple micronutrients, especially for children in early infancy (33,39,42,44,49,51,53,90,138,147–149). Two studies examined the effect of disease on stunting and found no association (33,54), though studies that looked at anemia found a dose response relationship between anemia severity and stunting risk (26,39,48). Diarrhea was also found to be an important risk factor for stunting outcomes (33,42,150), as was fever (29,42,48,150), however most studies treated ARI as an outcome with stunting as a significant predictor, not the other way around, which is an area for future research (49,90,138,151). Literature showed that generally, maternal age was not associated with stunting risk (17,21,22,26,35,39,41,54), but that mothers of shorter height (21,33–35,37,39,41,42,44,54) or those with lower BMI were found to be more likely to have stunted children (17,26,33,37,39,41,48,49,53,72), while increasing inter-pregnancy interval and fewer births is associated with a reduction in stunting outcomes (22,26,38,39,41,42,50,56). With regard to child characteristics, studies emphasize that as child age increases, so do stunting outcomes (17,21,26,33,37,40–42,46–53,56,57,90,138,152,153). Additionally, under-5 boys were found to be more likely to be stunted compared to girls (17,21,22,26,28,33,37,39–42,47–49,52–54,72,90,138,153), while low birthweight children were at an increased risk of stunting outcomes (17,33,39,42,48,49,53,54,59,90). A significant association was not found between birth order and stunting outcomes (17,24,33,35,47,48,50) but the majority of studies that looked at the number of children in households found that as the number of children under-5 in a household rises, stunting risk does as well (21,22,26,37,53,54).

**Appendix 2: Program and Policy Review**

After Emperor Selassie’s overthrow in 1974 following pervasive socio-economic issues and famine in 1973, the Provisional Military Administrative Council introduced Proclamation 31 in 1975. This proclamation allowed any individual willing to cultivate land to be given land not exceeding 10 hectares so as to promote equality and economic development (154,155). Other agriculture-related policies include the Agriculture Development Led Industrialization strategy (1993-2002) which represents Ethiopia’s overarching strategic framework for development. It focuses on strengthening ties between agriculture and industry in rural areas so that incomes for rural families may rise and the nation can achieve food self-sufficiency (156,157). The Industrial Development Policy followed in 2002 and continued to work toward agriculture-led industrialization, export-led development and labour-intensive industries (157–159). Also in 2002, the Rural Development Policy was introduced to minimize the need for foreign aid by developing Ethiopia’s market economy though initiatives such as strengthening the agricultural labor force, proper use of land, and directing agricultural development (157–159).

The Sustainable Development and Poverty Reduction Program was a three-year program beginning in 2002 with an aim to reduce poverty in Ethiopia. It aims to achieve this through numerous initiatives such as a focus on agriculture, strengthening private sector growth, increased exports, particularly of agricultural products, and investment in education (160). Its successor, A Plan for Accelerated and Sustained Development to End Poverty (PASDEP), aimed to work toward achievement of the Millennium Development goals. PASDEP ended in 2010 and its scope was wide, and impacted agriculture, healthcare, water and child nutrition (161,162). After PASDEP, the Growth and Transformation Plan (2010-2020) shares the goals of its predecessors in improving economic growth and ending poverty. This plan aims to quickly and equitably drive economic growth by focusing on agriculture, expanding infrastructure, empowering women, and ensuring good governance (163,164).

Ethiopia introduced many health and nutrition-related policies and programs over the study period. The Expanded Program on Immunization began in 1980 and continues into the present. It aims to accomplish 90% coverage of all vaccines nationally by 2020. In 1993, after the end of the Derg government, the Health Policy of the Transition Government of Ethiopia was created with a focus on women and children, the rural population, the poor, minorities, and victims of disaster (165–167). The Health Sector Development Program (HSDP) was introduced years later in 1997 and provided comprehensive, integrated, and cost-effective primary care (85,168). The National Strategy for Child Survival in Ethiopia was introduced in 2005 and will continue until 2020 with the goal to achieve universal high quality health coverage for mothers and newborns in communities and health facilities (169,170). This strategy utilizes the Health Extension Workers (HEWs) to reach communities. The Health Extension Program emanated from HSDP in 2003 and introduced HEWs to improve equitable access to health care in rural areas, despite resource limitations. HEWs focus on four major components: family health, disease prevention and control, hygiene and environmental sanitation, and health education and communication (86,171–174). The Enhanced Outreach Strategy and Targeted Supplementary Feeding Program (2004), and the National Nutritional Policy and Strategy (2008) both address child nutrition, and continue into the present (87,167,175,176).

Reductions in open defecation were an important factor in stunting decline in Ethiopia, with numerous policies addressing this important issue. Ethiopia’s Health Policy of the Transition Government, adopted in 1993 had a focus on sanitation and open defecation (177). The HSDP, adopted in 1997, emphasized environmental health and communicable disease prevention, ensuring safe environment (85,168). The Health Extension Program had a clear focus on reducing open defecation and teaching about latrines; in fact, 5 of the 16 domains were related to safe environment (86,171–174). Poverty reduction programs including SDPRP and PASDEP both had a focus on WASH, and the latter specifically promoted the use of latrines through the health extension worker program (161,162). The National Hygiene and Sanitation Strategy (2005) also emanated from the HSDP, and itself produced the National Hygiene and Sanitation Strategic Action Plan (2011) (178,179).

**Appendix 3: Qualitative Inquiry Results**

**National Expert Stakeholders**

Interviews were conducted with eleven national key informants from various ministries in Ethiopia, and NGOs. Basic drivers of stunting decline as reported by national informants include increased urbanization, poverty reduction, improvements in education and women’s empowerment, remittances and labour migration, and the decentralization and democratization process. This process helped regional states to improve agricultural productivity, infrastructure, and health services from a bottom-up process.

Nutrition-specific and –sensitive policies and programs discussed include programs related to community health, nutrition, poverty reduction, and health sector development. Barriers and facilitators of program and policy implementation was brought up, and key informants named the National Health Policy of 1993 as the primary guide for subsequent programs. Several key informants identified the Health Extension Program as the main driver of stunting decline in Ethiopia.

Underlying causes of stunting reduction discussed by national stakeholders include improvements to the household environment, food security and feeding practices, and access to health services. Improved sanitation and hygiene, specifically increased access to latrines, and major declines in open defecation were linked to the HEP.

*“again thanks to the HEP a lot has been done regarding toilet utilization, especially on South and Tigray region there are open defection free woredas.”*

- Ministry of Health representative

Among the immediate causes of stunting decline are improvements in dietary intake, maternal characteristics such as fertility rate, and reductions in childhood illness such as malaria, vaccine preventable diseases, and diarrhea.

**Regional Stakeholders**

Regional stakeholders, including teachers and health staff (health extension workers, maternal, newborn and child health care workers, district health surveillance focal persons, and senior health centre staff), were interviewed in-depth across two regions and four districts. Basic drivers of stunting decline brought up by regional stakeholders include peace and security, improvements to education and women’s empowerment, and poverty reduction.

A variety of nutrition-specific and –sensitive programs were discussed and implemented in community settings including the HEP, MERCY CORPS, one WASH, ENGINE, PSNP, and the Pastoralist Community Development Program (PCDP). The HEP was emphasized for resulting in substantial health and nutrition improvements.

Among the underlying causes of stunting reduction, interviewees discussed access to health services, WASH, and food security. The observed improvements in food security named by the regional stakeholders were related to increased agricultural production, as well as improved access to markets, availability of roads, and access to media.

Immediate causes of stunting reduction mentioned include dietary intake, infant and young child feeding, recommendations for optional breastfeeding and complementary feeding, increased contraceptive utilization, and declines in childhood communicable diseases including diarrhea, parasitic infection, typhus, and skin and eye infections. Key informants perceived limited access to animal source foods, though improved diversity of children’s diets over time.

*“Previously the dietary intake of people was poor. […] Children need different kinds of foods such as vegetables, fruits, rice pasta fish and milk. Nowadays people consume various diets that is rich in many nutrients.”*

- Community informant, Somali region

**Mothers in Communities**

Focused group discussions with mothers of children under-5 born during three time periods (1987-1991, 1995-1999, 2011-2015) were conducted in order to identify the factors that were salient in these time periods of substantial stunting decline. Poverty was mentioned as a concern by mothers of children in all three age groups in the Somali region, however it was not brought up by mothers in SNNPR. Improvements in both boys’ and girls’ education, as well as improved infrastructure such as roads or housing were brought up by mothers in both regions in the later years as distal drivers of stunting decline.

*“Our view of girl education is not the same now and then. We used to think that a girl that goes out early to learn was a bad girl. […] Now we educate both genders equally.”*

- Mother FGD Aware, Somali, 1995-1999

Neither mothers in the Somali region nor in SNNPR brought up any nutrition-specific and –sensitive policies, likely since they were not prompted on these and they may not have been familiar with specific government initiatives. Mothers in Somali were concerned about drought and water shortage in all years, and the price of food in later years. Mothers in both regions brought up access to safe water supply as concerning as well. Improved hygiene and sanitation, and health service access was mentioned by mothers in both regions in the latest group, while mothers in SNNPR in the latest group also spoke about improved crop production.

Immediate causes of stunting decline spoken about by mothers in both Somali region and SNNPR in the later years include improved child vaccination, and a decreased concern about childhood illness because health services were readily available. Mothers in the SNNPR region also brought up improved access to fruits and vegetables and improved child care in later years.
